# Leveraging Dual-Ligase Recruitment to Enhance Protein Degradation via a Heterotrivalent Proteolysis Targeting Chimera

**DOI:** 10.1021/jacs.4c11556

**Published:** 2024-11-28

**Authors:** Adam G. Bond, Miquel Muñoz i Ordoño, Celia M. Bisbach, Conner Craigon, Nikolai Makukhin, Elizabeth A. Caine, Manjula Nagala, Marjeta Urh, Georg E. Winter, Kristin M. Riching, Alessio Ciulli

**Affiliations:** † Centre for Targeted Protein Degradation, School of Life Sciences, University of Dundee, 1 James Lindsay Place, Dundee DD1 5JJ, U.K.; ‡ CeMM Research Center for Molecular Medicine of the Austrian Academy of Sciences Vienna 1090, Austria; § Promega Corporation, 2800 Woods Hollow Road, Madison, Wisconsin 53711, United States

## Abstract

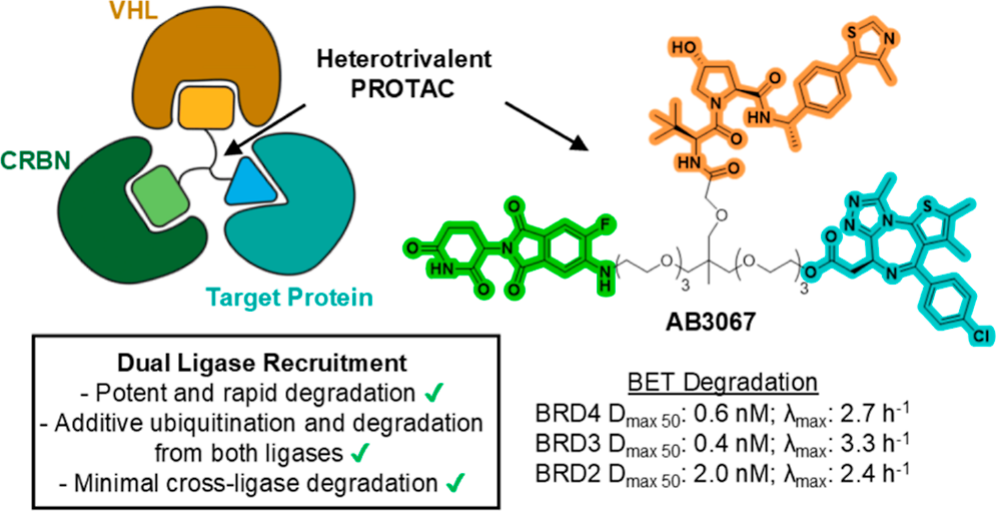

Proteolysis targeting chimera (PROTAC) degraders are typically bifunctional with one E3 ligase ligand connected to one target protein ligand via a linker. While augmented valency has been shown with trivalent PROTACs targeting two binding sites within a given target protein, or used to recruit two different targets, the possibility of recruiting two different E3 ligases within the same compound has not been demonstrated. Here we present dual-ligase recruitment as a strategy to enhance targeted protein degradation. We designed heterotrivalent PROTACs composed of CRBN, VHL and BET targeting ligands, separately tethered via a branched trifunctional linker. Structure–activity relationships of 12 analogues qualifies AB3067 as the most potent and fastest degrader of BET proteins, with minimal E3 ligase cross-degradation. Comparative kinetic analyses in wild-type and ligase single and double knockout cell lines revealed that protein ubiquitination and degradation induced by AB3067 was contributed to by both CRBN and VHL in an additive fashion. We further expand the scope of the dual-ligase approach by developing a heterotrivalent CRBN/VHL-based BromoTag degrader and a tetravalent PROTAC comprising of two BET ligand moieties. In summary, we provide proof-of-concept for dual-E3 ligase recruitment as a strategy to boost degradation fitness by recruiting two E3 ligases with a single degrader molecule. This approach could potentially delay the outset of resistance mechanisms involving loss of E3 ligase functionality.

## 1 Introduction

Proteolysis targeting chimeras (PROTACs) are bifunctional molecules that enforce proximity between a target protein and a ubiquitin E3 ligase to induce poly ubiquitination and proteasomal degradation of the target protein.^1−4^ This small-molecule modality features a catalytic, “event-driven” mode of action, which brings benefits including lower doses and more durable pharmacological effects compared to occupancy-based inhibitors that must bind a functional site on the target protein to block its function.^5^ PROTACs typically consist of a ligand for a target protein, connected by a chemical linker, to another ligand for an E3 ligase. This enables simultaneous recruitment and formation of a 1:1:1 ternary complex of a single molecule of target protein, the PROTAC and a single molecule of E3 ligase component. Most often, the recruited E3 ligases are either cereblon (CRBN) or von Hippel-Lindau (VHL).^6^ Despite the advantages and remarkable successes achieved, it can be challenging to design PROTACs that effectively perform as desired in cells or in vivo, often requiring extensive chemical optimization to achieve significant levels of degradation of the target protein.^7−9^ The target spectrum of PROTACs is broad, with over 30 PROTAC degraders for important oncogenes and other disease-driving proteins currently in clinical development.^1^ However, all PROTACs in the clinic and the vast majority of those published and patented to date recruit and depend on the activity of a single ubiquitin E3 ligase.

We and others became intrigued by the possibility that augmenting the valency of PROTACs might offer advantages by leveraging the principles and benefits of multitargeting poly pharmacology and/or avidity.^10^ In a first foray of such approach, our group developed the concept of trivalent PROTACs and exemplified this with molecules embodying two ligands that can simultaneously bind to two sites on separate domains of the same target protein (rather than two different targets). Trivalent PROTAC SIM1 connected a single VHL ligand to two ligands of a Bromo- and Extra-Terminal (BET) domain protein ligand, joined via a trifunctional linker.^11^ SIM1 effectively and durably degrades BET proteins with picomolar degradation potency, without any detectable hook effect up to micromolar concentrations, due to the combined avidity of a simultaneous *cis*-engagement of both BET bromodomains, and the cooperativity of subsequently engaging VHL in a 1:1:1 complex with the BET bromodomain protein.^11^ Subsequently, others have developed multifunctional PROTACs capable of degrading more than one target at the same time, through conjugation of two distinct ligands recruiting two different target proteins to a single E3 ligase ligand.^12^

Based on the success of SIM1, we became intrigued by the possibility of whether recruiting two different E3 ligases (e.g., CRBN and VHL) simultaneously to a given target protein would have a synergistic and potentially additive effect on target protein degradation. We reasoned that such an approach of recruiting two different E3 ligases could boost protein degradation fitness, beyond what could be attained by a PROTAC molecule dependent on a single E3 ligase, while minimizing cross-E3 degradation. Moreover, we imagined that leveraging dual-E3 ligase activity would circumvent dependency on a single E3 ligase, a known Achilles’ heel of PROTAC’s mode of action that leads to loss of E3 ligase functionality as a well-known mechanism of cellular resistance to targeted protein degradation.^13−15^ We therefore envisaged trifunctional or multifunctional molecules composed of one ligand for VHL, one ligand for CRBN, and one or more instances of target protein ligands. Such “hetero-multivalent” PROTACs would combine the ubiquitination activity from each E3 ligase, circumventing potential limitations of using two heterobivalent PROTAC molecules which would instead compete for binding to the same target protein, while also alleviating issues of having to dose two different compounds at the same time.

Here we provide proof-of-concept of this strategy with heteromultivalent molecules designed to simultaneously recruit VHL and CRBN to BET bromodomains with one ligand for each. Cellular degradation and target engagement screens validated the concept and identified a potent, proteome-wide selective and highest-performing heterotrivalent degrader, with minimal cross-E3 degradation. Real-time kinetic ternary complex, ubiquitination and degradation assays in wild-type and E3 knockout cell lines evidenced additive contribution to ubiquitination/degradation by both E3 ligases, which could not be blocked by loss of a single E3 (as for bivalent PROTACs), and instead requiring a double ligase knockout. We further exemplify our dual-ligase approach via a heterotrivalent CRBN/VHL-based BromoTag degrader and an unprecedented heterotetravalent PROTAC comprising of 1 × VHL, 1 × CRBN and 2 × BET ligand moieties.

## 2 Results and Discussion

### 2.1 Heterotrivalent PROTAC Design Rationale

**Figure 1:**
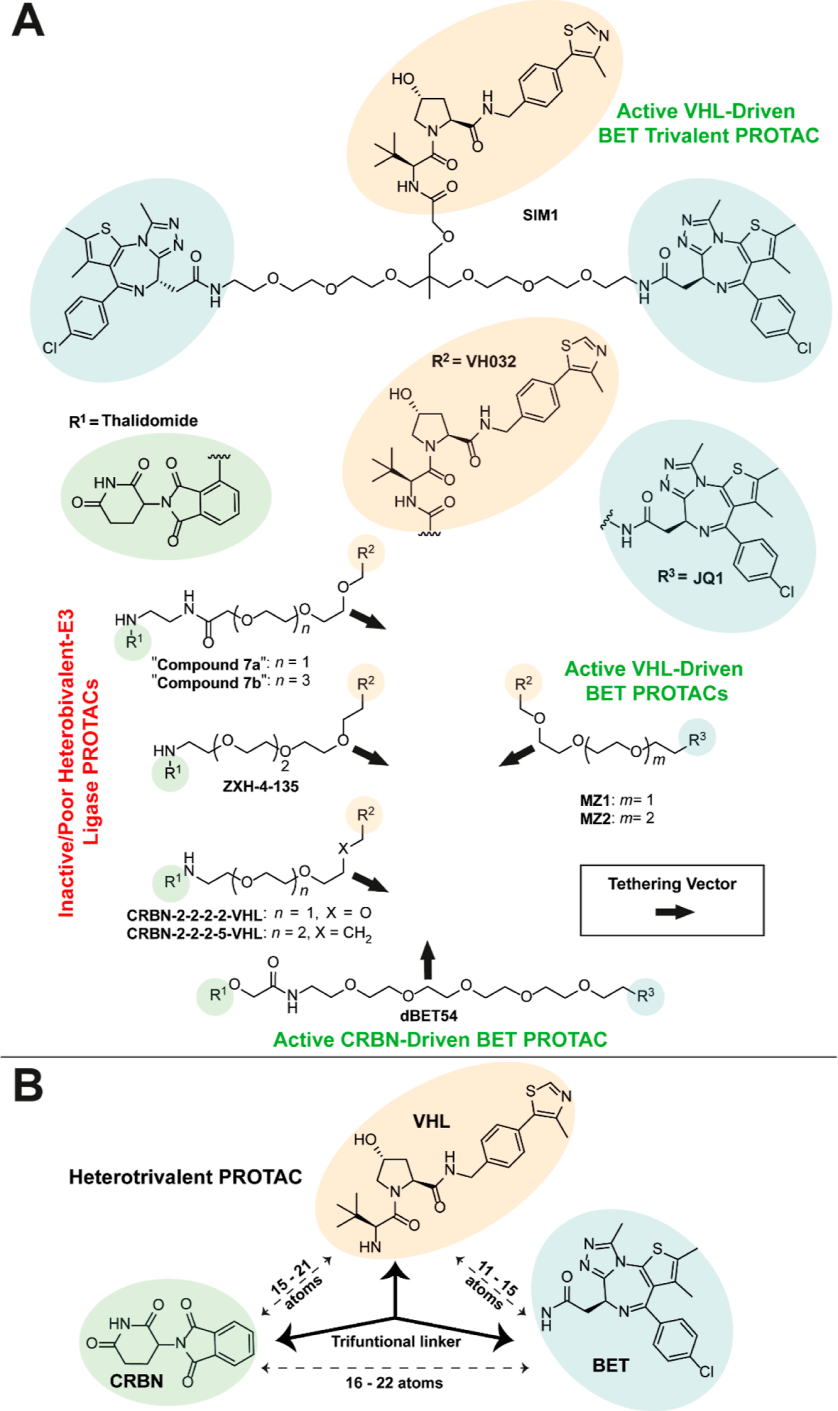
Heterotrivalent PROTAC design rationale. (*A*) Active VHL-driven BET trivalent PROTAC, **SIM1** (top), and bivalent PROTACs, **MZ1** and **MZ2** (middle right). Active CRBN-driven BET PROTAC, **dBET54** (bottom). Inactive CRBN-VHL Heterobifunctional-E3 Ligase PROTACs, **“Compounds 7a & 7b”**, **ZXH-4–135**, **CRBN-2–2–2–2-VHL** and **CRBN-2–2–2–5-VHL** (middle left). VHL ligand, VH032 (orange), BET ligand, JQ1 (blue), and CRBN ligand, thalidomide (green) are highlighted. Black arrows indicate potential vectors for linker tethering. (*B*) Simplified structure of a heterotrivalent PROTAC labeled with optimal linker lengths required between each ligand to have active VHL/CRBN driven BET degradation and to avoid cross-ligase degradation of VHL and/or CRBN.

When envisaging the heterotrivalent PROTACs, we kept several design criteria in mind. The linkage between the VHL ligand VH032, and the BET ligand JQ1 should allow for VHL-driven BET degradation; and similarly, the linkage between CRBN-binding thalidomide and JQ1 should allow for CRBN-driven BET degradation. In contrast, we intended for the linkage between VH032 and thalidomide should minimize any cross-degradation between VHL and CRBN. To link VH032 with JQ1, we used the linker lengths of active VHL recruiting trivalent, SIM1,^11^ and bivalent, MZ1^16^ and MZ2^16^ (differing by one PEG unit in the linker) BET degraders as scaffolds, which have 15, 11, and 14 atoms, respectively, between the terminal amide NH groups of VH032 and JQ1 (Figure 1A). To enable CRBN mediated degradation of BET proteins, and to allow for adequate length between JQ1 and thalidomide, we chose to use the scaffold of dBET54,^17^ an active CRBN recruiting BET degrader comprising of a 21 atom long linker between thalidomide and the amide NH of JQ1. Lastly, to best avoid E3 ligase cross-degradation, we opted to use the linker lengths of inactive/poor CRBN-VHL heterobifunctional-E3 ligase degraders, “Compounds 7a & 7b”,^18^ ZXH-4–135,^19^ CRBN-2–2–2–2-VHL,^20^ and CRBN-2–2–2–5-VHL,^20^ (15, 21, 16, 12, and 15 atoms, respectively, between the amide NH of VH032 and thalidomide) (Figure 1A).

When overlaying the structures of CRBN-2–2–2–2-VHL or CRBN-2–2–2–5-VHL with either MZ1 or MZ2, we envisaged an optimal linker composition and length between thalidomide and JQ1 (16–22 C/O atoms) that would ensure both VHL and CRBN-driven degradation of BET proteins, while helping to avoid the degradation of either ligase (Figure 1B).

### 2.2 Initial Heterotrivalent PROTACs

**Figure 2:**
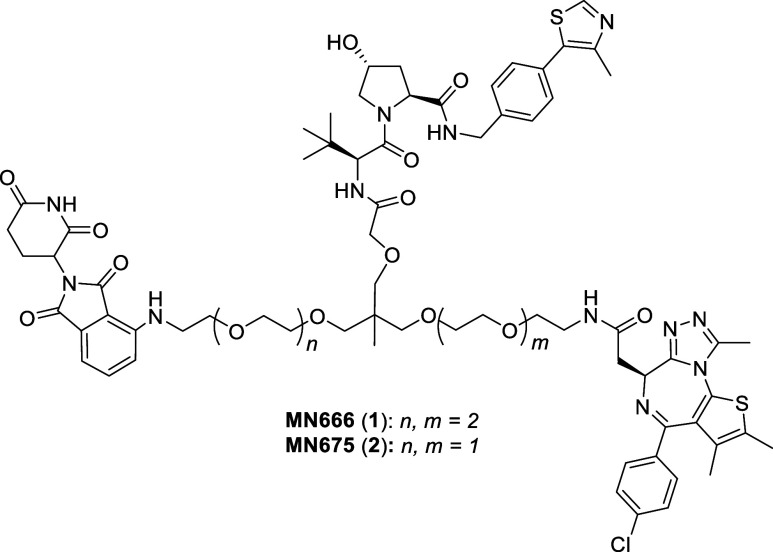
Chemical structures of first generation heterotrivalent PROTACs. Chemical structures of MN666 (1) and MN675 (2).

For proof-of-concept, we initially set out to synthesize two heterotrivalent compounds, MN666 (**1**) and MN675 (**2**) (Figure 2). The structure of **1** shares a scaffold much like that of SIM1, differing only by a JQ1 ligand being substituted with thalidomide via an aniline tether. **2** is a smaller analogue of **1**, with a PEG unit removed from both VH032-JQ1 and VH032-thalidomide sides to the linker.

**Scheme 1:**
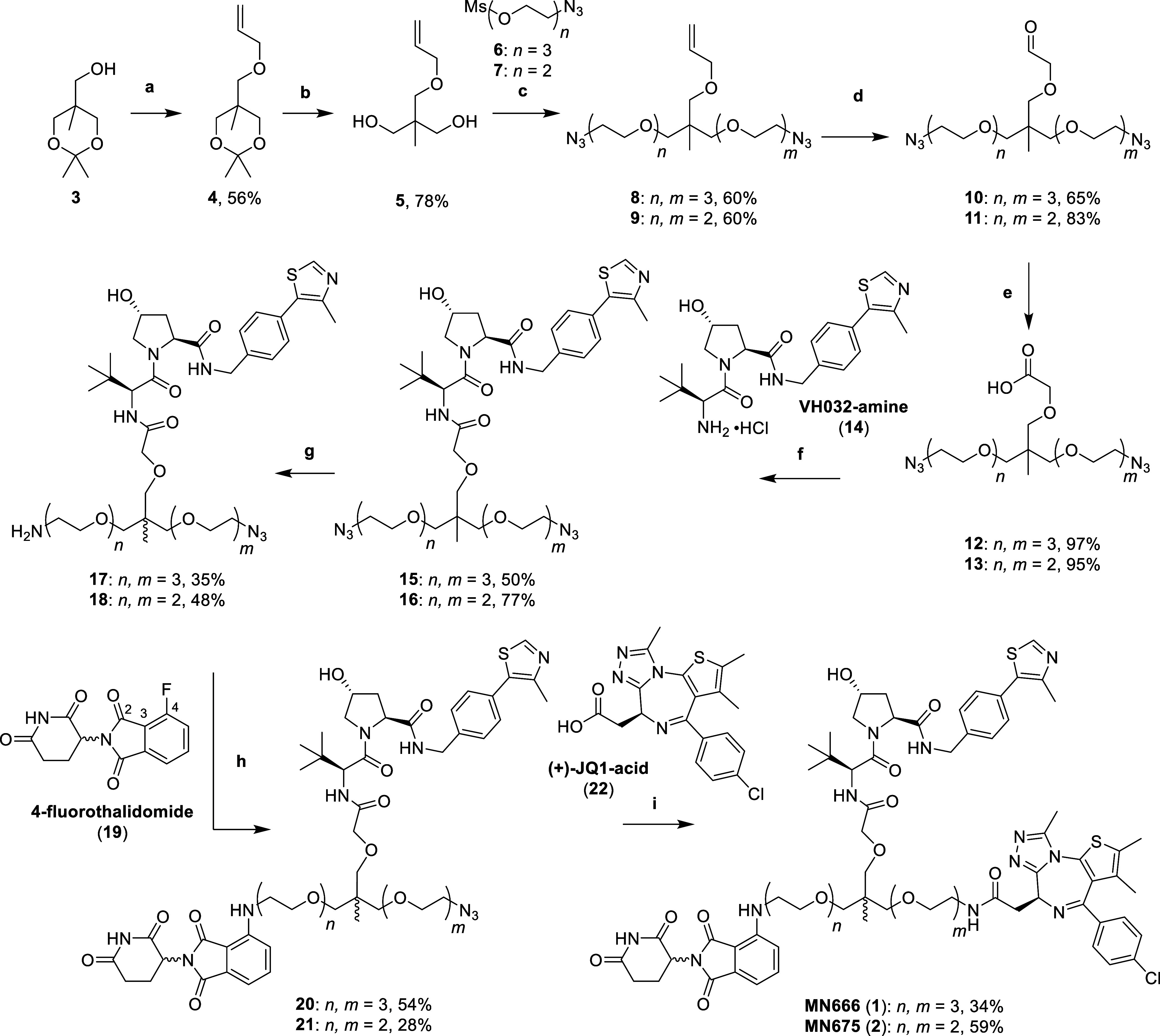
Synthesis of Initial Heterotrivalent PROTACs MN666 (**1**) and MN675 (**2**) Reaction conditions: (a) allyl bromide, KOH, TBAB, toluene, H_2_O, r.t., 16 h; (b) TFA, MeOH, H_2_O, r.t., 3 h; (c) (i) NaH, DMF, 0 °C, 30 min, (ii) **6** or **7**, DMF, 60 °C, 16 h; (d) OsO_4_, NaIO_4_, 2,6-lutidine, dioxane, H_2_O, r.t., 16 h; (e) 2-methyl-2-butene, NaH_2_PO_4_, NaClO_2_, *t*-BuOH, H_2_O, r.t., 16 h; (f) VH032-amine (**14**), HATU, DIPEA, DMF, r.t., 2 h; (g) PPh_3_, EtOAc, 1.0 M HCl (aq); (h) **19**, DIPEA, NMP, 100–120 °C, 4 h; (i) (i) H_2_, 10% Pd/C, MeOH, r.t., 16 h, (ii) (+)-JQ1-acid (**22**), HATU, DIPEA, DMF, r.t., 2 h.

Using a similar route described by Imaide et al. for the synthesis of SIM1,^11^ alcohol **3** was first alkylated with allyl bromide in a solution of potassium hydroxide and tetrabutylammonium bromide (TBAB) in toluene and water to give allyl ether **4** (Scheme 1). The acetonide of **3** was hydrolyzed with trifluoroacetic acid (TFA) in methanol and water to yield diol **5**. Next, diol **5** was deprotonated twice using sodium hydride (4 equiv) at 0 °C in dimethylformamide (DMF), before the addition of azido mesylates **6** and **7** and heating to 60 °C to yield dialkylated allyl ethers **8** and **9**, respectively. Next, alkenes **8** and **9** were oxidatively cleaved with sodium periodate, 2,6-lutidine and a catalytic amount of osmium tetroxide in dioxane and water to yield aldehydes **10** and **11**. Then, aldehydes **10** and **11** underwent a Pinnick oxidation by treating them with 2-methyl-2-butene, monobasic sodium phosphate and sodium chlorite in *tert*-butanol and water to yield carboxylic acids **12** and **13** (Scheme 1).

With the trifunctional linkers in hand, the next step was to couple acids **12** and **13** with the terminal amine of VHL ligand, VH032 (**14**, synthesized through literature procedures^21,22^) via standard amide coupling conditions with 1-[bis(dimethylamino)methylene]-1*H*-1,2,3-triazolo[4,5-*b*]pyridinium 3-oxid hexafluorophosphate (HATU) and diisopropylethylamine (DIPEA) in DMF to yield amides **15** and **16**. A Staudinger reduction was then employed to reduce a single azide of diazides **15** and **16** by slow addition with 1 eq. of triphenylphosphine in ethyl acetate and 1.0 M aqueous hydrochloric acid to yield monoamines **17** and **18**. Next, amines **17** and **18** underwent nucleophilic aromatic substitution (S_N_Ar) with CRBN ligand, 4-fluorothalidomide (**19**), by heating with DIPEA in *N*-methyl-2-pyrrolidone (NMP) between 100 and 120 °C to yield 4-substituted anilines **20** and **21**. Finally, the azides of **20** and **21** were reduced with a suspension of 10% palladium on carbon (Pd/C) in methanol, under an atmosphere of hydrogen gas. The intermediate amines were immediately coupled to the acid of BET ligand, JQ1 (**22**) using HATU and DIPEA in DMF to yield the amides of heterotrivalent PROTACs MN666 (**1**) and MN675 (**2**) (Scheme 1).

**Figure 3:**
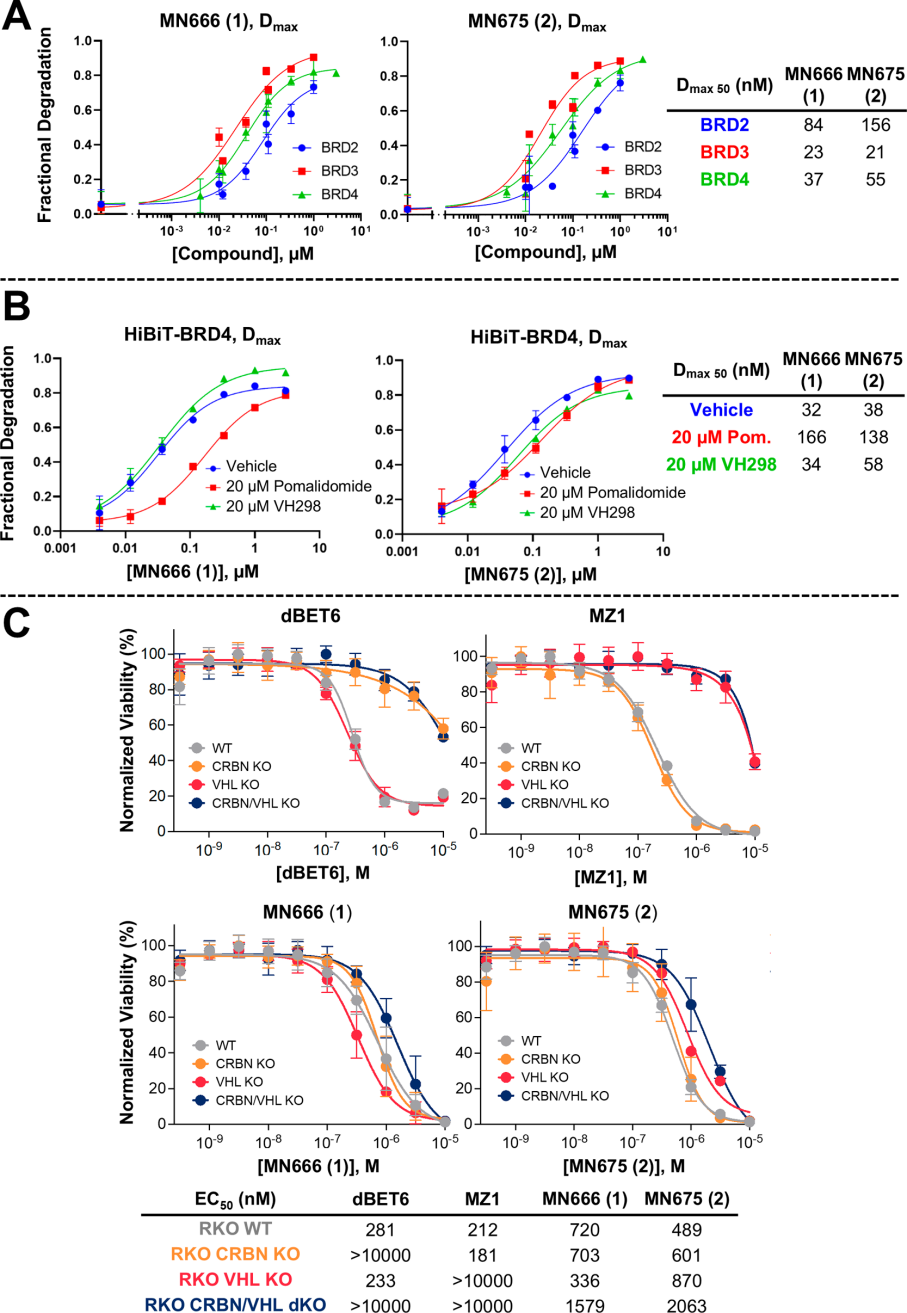
Cellular evaluation of MN666 (**1**) and MN675 (**2**). (A) (B) Degradation potency of **1** and **2** from live cell kinetic profiles in HiBiT-BRD CRISPR knock-in HEK293 cells plotted as fractional degradation at *D*_max_ versus concentration of **1** (left) and **2** (right). Cells were treated with DMSO and a threefold serial dilution of **1** or **2** over a concentration range of 4 nM to 3 μM without (*A*) or with (*B*) 20 μM of either CRBN inhibitor pomalidomide, or VHL inhibitor VH298. *D*_max 50_ is tabulated. Mean ± S.D.; *n* = 3 biological replicates (*A*) or *n* = 1 biological replicates (*B*). (*C*) Cell viability assay in BET sensitive wild-type and CRBN/VHL knockout RKO cell lines. Cell antiproliferation of MZ1 and dBET6 (top) compared to **1** and **2** (bottom) after 316 pM to 10 μM treatment in WT, CRBN KO, VHL KO or CRBN/VHL dKO RKO cell lines. Mean ± S.D.; *n* = 6 biological replicates. EC_50_ values are tabulated below and in Table S1 with 95% CI.

With our initial heterotrivalent PROTACs **1** and **2** in hand, we moved to evaluate the BET degradation profiles in cells. To this end, we performed live cell kinetic degradation assays in previously established CRISPR-edited HEK293 cell lines in which the 11-amino acid peptide, HiBiT, is appended to the N-terminus of endogenous BRD2, BRD3, and BRD4, and which stably express the 18 kDa LgBiT protein to produce NanoBiT luminescence.^23^ We treated HiBiT-tagged BRD2, BRD3 and BRD4 HEK293 cells with varying concentrations of **1** and **2** (Figure 3A, kinetic traces provided in Figure S1). Both **1** and **2** induced degradation of BRD2, BRD3 and BRD4, with *D*_max 50_s of 84 and 156 nM, respectively for BRD2; 23 and 21 nM, respectively for BRD3; and 37 and 55 nM respectively for BRD4.

To assess whether each E3 ligase ligand of **1** and **2** was able to drive degradation, we ran a similar experiment by treating HiBiT-BRD4 HEK293 cells with **1** or **2**, with or without pretreatment of either CRBN ligand pomalidomide,^24^ or VHL inhibitor VH298 (Figure 3B).^25^ When VHL binding is blocked by VH298, the degradation profiles of **1** and **2** are not drastically affected by the inability to recruit VHL when comparing to the vehicle control, with *D*_max 50_s of 34 and 58 nM, vs 32 and 38 nM, respectively (Figure 3B). In contrast, when CRBN recruitment was outcompeted by pomalidomide binding, the degradation potency dropped by 5.3-fold in the case of **1** (*D*_max 50_s of 32 vs 166 nM), and 3.6-fold in the case of **2** (*D*_max 50_s of 38 vs 138 nM). This data suggests that although both **1** and **2** can degrade BRD4 in the absence of either VHL or CRBN, there is a strong preference for CRBN mediated degradation over VHL. We further assessed the contributions of each ligase warhead of **1** and **2** by monitoring the compound’s antiproliferative effect in the BET sensitive, poorly differentiated colon carcinoma cell line, RKO, for which we had generated both CRBN and VHL single knockouts (KO), and CRBN/VHL double knockouts (dKO) (Figure 3C and Table S1).

For reference and benchmarking, we treated each RKO cell line with heterobivalent BET degraders MZ1^16^ (VHL-dependent) and dBET6^26^ (CRBN-dependent). When both VHL and CRBN are knocked out in the same cell line, neither MZ1 nor dBET6 can recruit an E3 ligase (EC_50_*s* > 10 μM), giving rise to >fourfold cell antiproliferation when compared to the wildtype cell line, consistent with the well documented greater cellular impact of BET degradation over and above BET inhibition.^26^ Interestingly, even in the absence of both CRBN and VHL, **1** and **2** exhibited a similar antiproliferation profile when compared to that of wild-type and single CRBN or VHL KO RKO cells. Strikingly, **1** and **2** gave a > fivefold increase in cell antiproliferation in the CRBN/VHL dKO cell line when compared with MZ1 and dBET6, even though they all share the same BET ligand, JQ1. This suggested that **1** and **2** are likely acting, at least in part, as potent BET inhibitors. This causes a significant reduction in the desired enhanced antiproliferative effect, as seen for MZ1 and dBET6, from degrading BET proteins in WT, VHL and/or CRBN KO cells, respectively, over and above what observed in dKO cells (Figure 3C and Table S1). It was also curious to observe more potent cytotoxicity in the case of compound **1** (but not for compound **2**) in single VHL KO cells (EC_50_ = 336 nM) compared to WT (EC_50_ = 720 nM). Albeit small (just over twofold), we do not know the cause of this difference. We speculate that in the absence of one E3 ligase (in this case, VHL), the degradation-inducing component from the remaining E3 ligase (CRBN) or the BET-inhibitory component of the compound could more substantially contribute to the observed cytotoxicity as compared to when the compound acts in the presence of both E3 ligases.

Taken together, the data shows that while **1** and **2** can induce degradation of BET proteins by engaging either ligase, the induced degradation activity was not comparably driven by each E3 ligase, and that there remained a strong nondegrading component to the compound’s cellular antiproliferative activity. We therefore sought to develop a larger and more diverse set of heterotrivalent PROTACs to expand the chemical space, and to improve on the characteristics presented by **1** and **2**.

### 2.3 Second Generation Heterotrivalent PROTACs

A first strategy to help improve our initial compounds, **1** and **2**, was to switch from an amide linkage for JQ1 conjugation chemistry to an ester. A modification which we have previously shown to be beneficial when optimizing JQ1-based BET degraders by increasing the degradation efficacy through enhancements in compound cellular permeability.^27,28^ Next, we chose to introduce diversity in the VHL binding ligand by adding a methyl group to the benzylic position of VH032, a modification that is known to enhance the binary binding affinity to VHL.^27−29^ To gain a better understanding of the effects on linker length between each ligand, we decided to synthesize analogues which varied in the number of PEG units separating either JQ1 or thalidomide to the central quaternary carbon center of the linker (Table 1). Moreover, we wanted to vary the linker attachment vector and functionality to thalidomide. In addition to the anilines tethered at the 4-C of the phthalimide, we sought to use another linkage vector at the 5-C, a tethering site which has been used successfully in other CRBN-recruiting PROTACs.^30−33^ Alongside the linkage vector at the 5-C, we wanted to introduce a fluorine atom ortho to the aniline. A fluorine at the 5/6-position of the phthalimide group of thalidomide has been shown to increase both binding affinity to CRBN and to help reduce off-target degradation of neo-substrates, Aiolos (IKZF3) and CK1α.^34^ Finally, we chose to make two compounds which would be attached to thalidomide via a piperazine at either the 4-C or 5-C of the phthalimide ring. This weakly basic functionality is commonly used to aid in solubility and has also been widely used in CRBN-recruiting degraders (Table 1).^32,33,35−37^

**Table 1:**
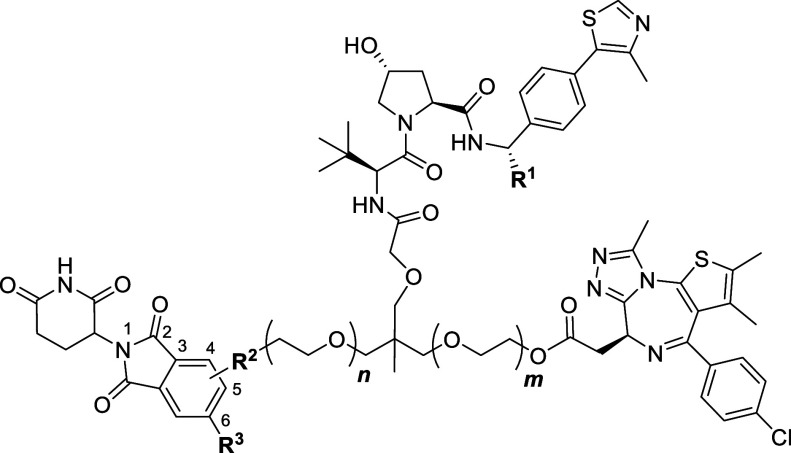

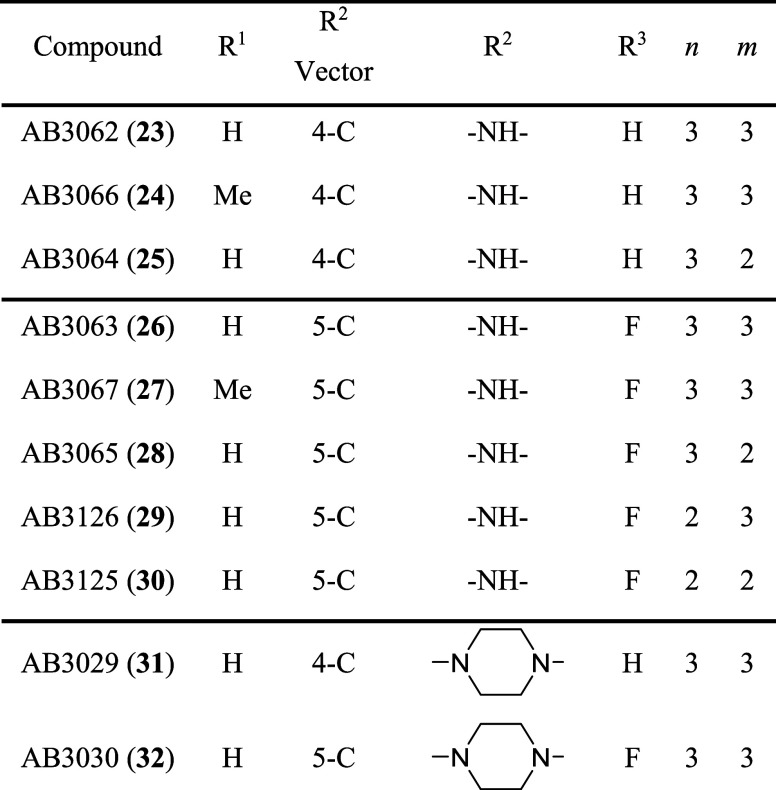
Second Generation Heterotrivalent PROTAC Library (**23–32**)

**Scheme 2:**
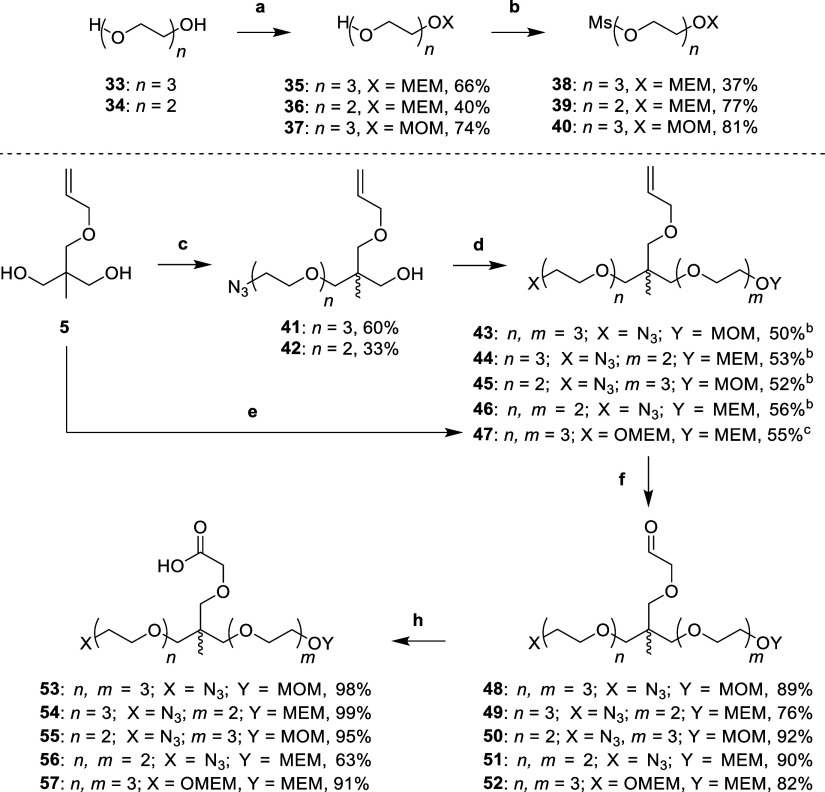
Synthesis of Trifunctional Linkers **53–57** Reaction conditions: (a) MEMCl or MOMBr, DIPEA, DCM, r.t., 16 h; (b) MsCl, DIPEA, DCM, r.t., 3 h; (c) (i) NaH, DMF, 0 °C, 30 min, (ii) **6** or **7**, DMF, 60 °C, 16 h; (d) (i) NaH, DMF, 0 °C, 30 min, (ii) **39** or **40**, DMF, 60 °C, 16 h; (e) (i) NaH, DMF, 0 °C, 30 min, (ii) **38**, DMF, 60 °C, 16 h; (f) OsO_4_, NaIO_4_, 2,6-lutidine, dioxane, H_2_O, r.t., 16 h; (h) 2-methyl-2-butene, NaH_2_PO_4_, NaClO_2_, *t*-BuOH, H_2_O, r.t., 16 h. Products **43**–**47** formed through step (f) then (g). Product **47** formed directly from step (e).

To synthesize linkers which would enable orthogonal trifunctionality, we adapted the approach seen in Scheme 1 and previously reported routes to make the linker for SIM1.^11^ The linker design consisted of the following functionalities: a carboxylic acid, to allow for facile amide coupling to VHL ligands; an amine masked as an azide, to enable future S_N_Ar attachment to thalidomide; and an alcohol protected by an acid labile acetal, to allow for both esterification to JQ1, and also for mesylation and subsequent nucleophilic substitution of piperazine substituted derivatives of thalidomide (Scheme 2).

To make linkers which would ultimately result in a primary alcohol required for later esterification to JQ1, acetal protecting groups, methoxyethoxymethyl (MEM) and methoxymethyl (MOM), were selected to mask the alcohol functionality. First, triethylene (**33**) and diethylene (**34**) glycols were treated with either methoxyethoxymethyl chloride (MEMCl) or methoxymethyl bromide (MOMBr) in DIPEA and dichloromethane (DCM) to afford mono-MEM protected alcohols **35** and **36**, and mono-MOM protected alcohol **37**, respectively. Then, alcohols **35**–**37** were treated with methanesulfonyl chloride (MsCl) with DIPEA in DCM to yield mesylates **38**–**40** (Scheme 2).

To build the trifunctional scaffold, diol **5** was carefully deprotonated using of sodium hydride (1.2 equiv) at 0 °C in DMF, before addition of azido mesylates **6** and **7** with heating at 60 °C to yield monoalkylated products **41** and **42**, respectively. This alkylation step was repeated through deprotonation of the alcohols of **41** and **42**, using sodium hydride (1.5 equiv) at 0 °C, before heating to 60 °C with acetal-protected mesylates **40** and **39** to afford dialkylated allyl ethers **43**–**46**. Diol **5** was also subjected to double deprotonation with sodium hydride (4 equiv) at 0 °C, before quenching with MEM-protected mesylate **38** (4 equiv) and heating to 60 °C to yield dialkylated allyl ether **47**. Next, the alkenes of **43**–**47** were oxidatively cleaved with sodium periodate, 2,6-lutidine and a catalytic amount of osmium tetroxide in dioxane and water to yield aldehydes **48**–**52**. Finally, aldehydes **48**–**52** underwent a Pinnick oxidation by treating them with 2-methyl-2-butene, monobasic sodium phosphate and sodium chlorite in *tert*-butanol and water to yield carboxylic acids **53**–**57** (Scheme 2).

**Scheme 3:**
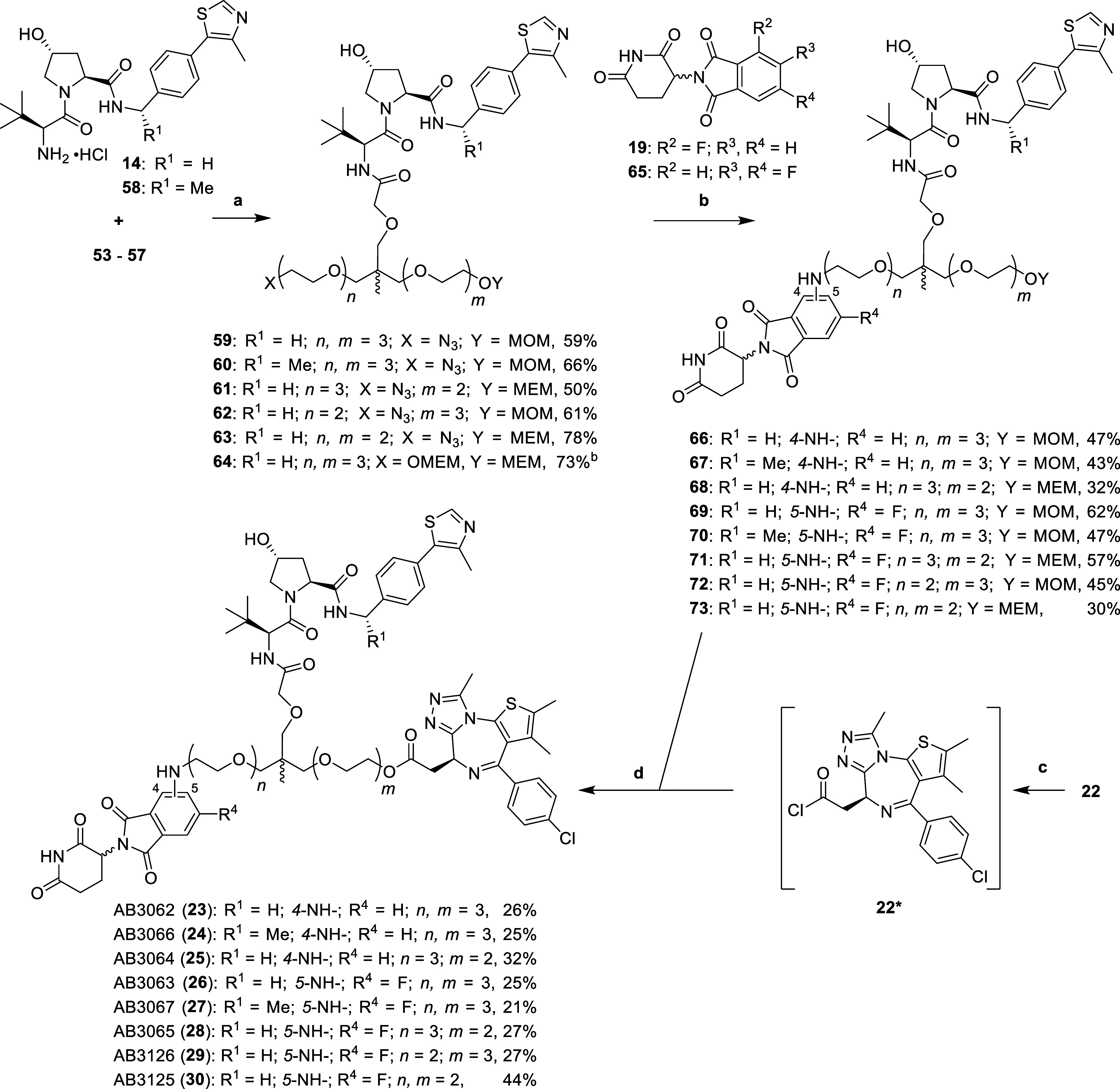
Synthesis of Aniline Tethered Heterotrivalent PROTACs **23–30** Reaction conditions: (a) HATU, DIPEA, DMF, r.t., 2 h; (b) (i) **59**–**64**, H_2_, 10% Pd/C, MeOH, r.t., 16 h, (ii) **19** or **65**, DIPEA, DMSO, 90 °C, 16 h; (c) SOCl_2_, DCM, r.t., 3 h; (d) (i) 4 N HCl in dioxane, MeOH, r.t., 3 h, (ii) 22*, DIPEA, DCM, r.t., 16 h. Product used further in Scheme 4.

The final part of the synthesis involved attachment of the trifunctional linker to the respective VHL, CRBN and BET ligands (Scheme 3).

Carboxylic acid **53** was coupled to both VH032-amine (**14**) and Me-VH032-amine (**58**, synthesized through literature procedures^38^) using HATU and DIPEA in DMF to yield amides **59** and **60**, respectively. The remaining acids **54**–**57** were coupled to VH032-amine (**14**) only using the same conditions to yield amides **61**–**64**. Next, the azides of **59**–**63** were reduced with a suspension of 10% Pd/C in methanol, under an atmosphere of hydrogen gas. The intermediate amines subsequently underwent an S_N_Ar reaction with 4-fluoro **19** and 5,6-difluoro **65** derivatives of thalidomide, by heating with DIPEA in DMSO at 90 °C to yield 4-substituted anilines **66**–**68** and 5-substituted-6-fluoro anilines **69**–**73**. Finally, MOM/MEM protecting groups of **66**–**73** were hydrolyzed with 4 N hydrochloric acid in dioxane and methanol. The subsequent primary alcohols were immediately conjugated to an intermediate acid chloride (22*), formed after treating (+)-JQ1-acid (**22**) with thionyl chloride in DCM, to afford the esters of aniline tethered heterotrivalent PROTACs **23**–**30** (Scheme 3).

**Scheme 4:**
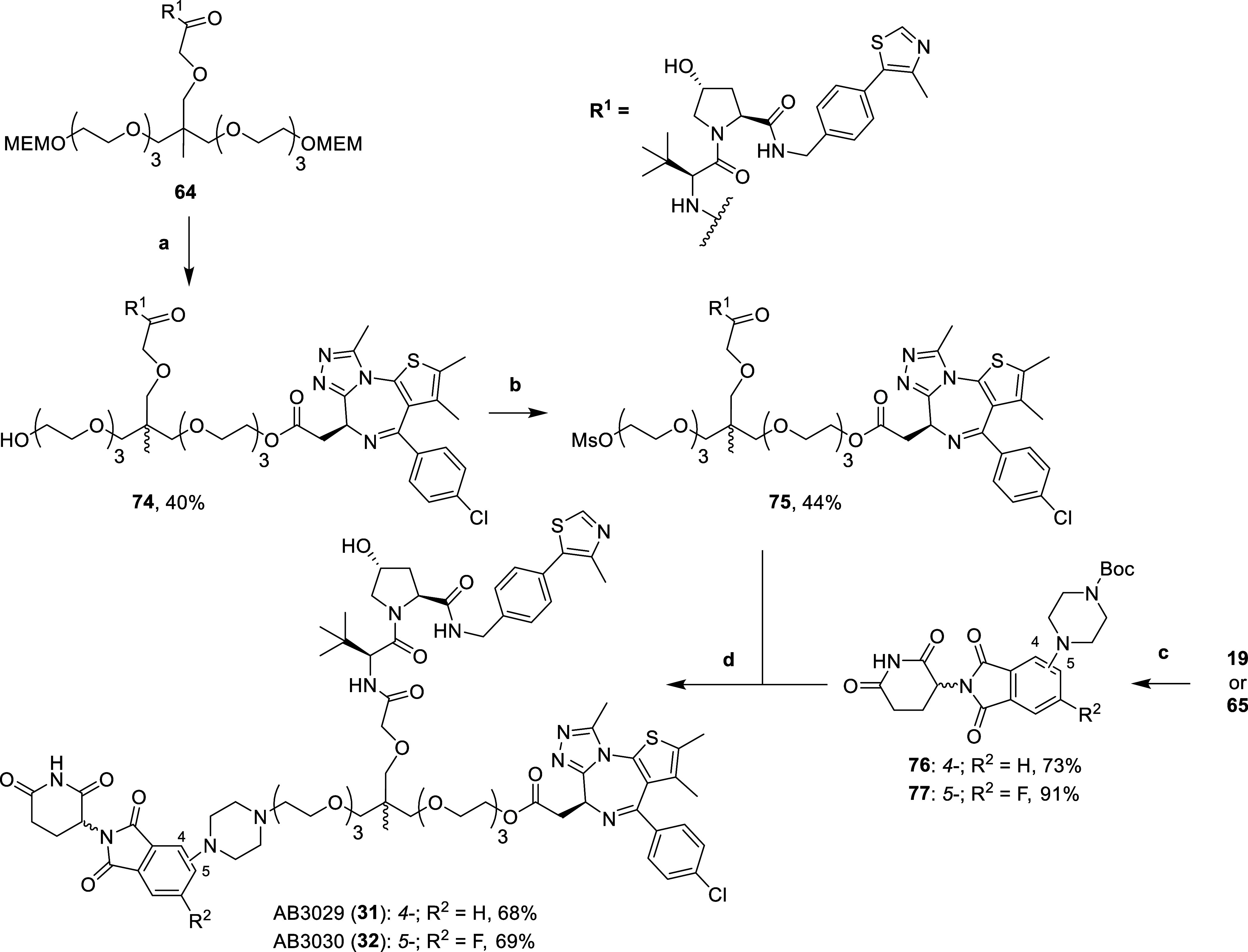
Synthesis of Piperazinyl Tethered Heterotrivalent PROTACs **31** & **32** Reaction conditions: (a) (i) 4 N HCl in dioxane, MeOH, r.t., 1 h, (ii) 22*, DIPEA, DCM, r.t., 16 h; (b) (i) MsCl, DIPEA, DCM, 0 °C, 20 min, (ii) r.t., 1 h; (c) 1-Boc-piperazine, DIPEA, DMSO, 90 °C, 16 h; (d) (i) **76** or **77**, 4 N HCl in dioxane, DCM, r.t., 16 h, (ii) **75**, DIPEA, DMF, 80 °C, 16 h.

To synthesize heterotrivalent PROTACs whose linkers are tethered via a piperazine to thalidomide, we required two masked alcohols in the linker to allow for esterification to JQ1, and also for mesylation and subsequent amination with piperazine substituted thalidomide derivatives (Scheme 4).

First, both MEM groups of compound **64** (synthesized in Scheme 3), were hydrolyzed with 4 N hydrochloric acid in dioxane and methanol. The intermediate diol was reacted with substoichiometric amounts (0.8 equiv) of the acid chloride 22* (synthesized in Scheme 3) to afford monoester **74**. The remaining primary alcohol of **74** was mesylated by careful addition of MsCl in DCM at 0 °C to yield mesylate **75**. Careful addition is required due to the observed formation of a dimesylated product, where another mesyl group is attached to the secondary alcohol present on the hydroxyproline of the VH032 ligand, a group usually inert in other reactions. Next step was to functionalize thalidomide derivatives **19** and **65** by heating them at 90 °C with 1-Boc-piperazine and DIPEA in DMSO to give Boc-protected 4-piperazinyl (**76**) and 5-piperazinyl-6-fluoro (**77**) substituted thalidomide. The Boc groups of **76** and **77** were then hydrolyzed using 4 N hydrochloric acid in dioxane and DCM. The intermediate hydrogen chloride salts were then alkylated by heating to 80 °C with mesylate **75** and DIPEA in DMSO to yield the tertiary amine present in piperazine tethered heterotrivalent PROTACs **31** and **32**.

### 2.4 Cellular Evaluation of Second Generation Heterotrivalent PROTACs **23**–**32**

**Figure 4:**
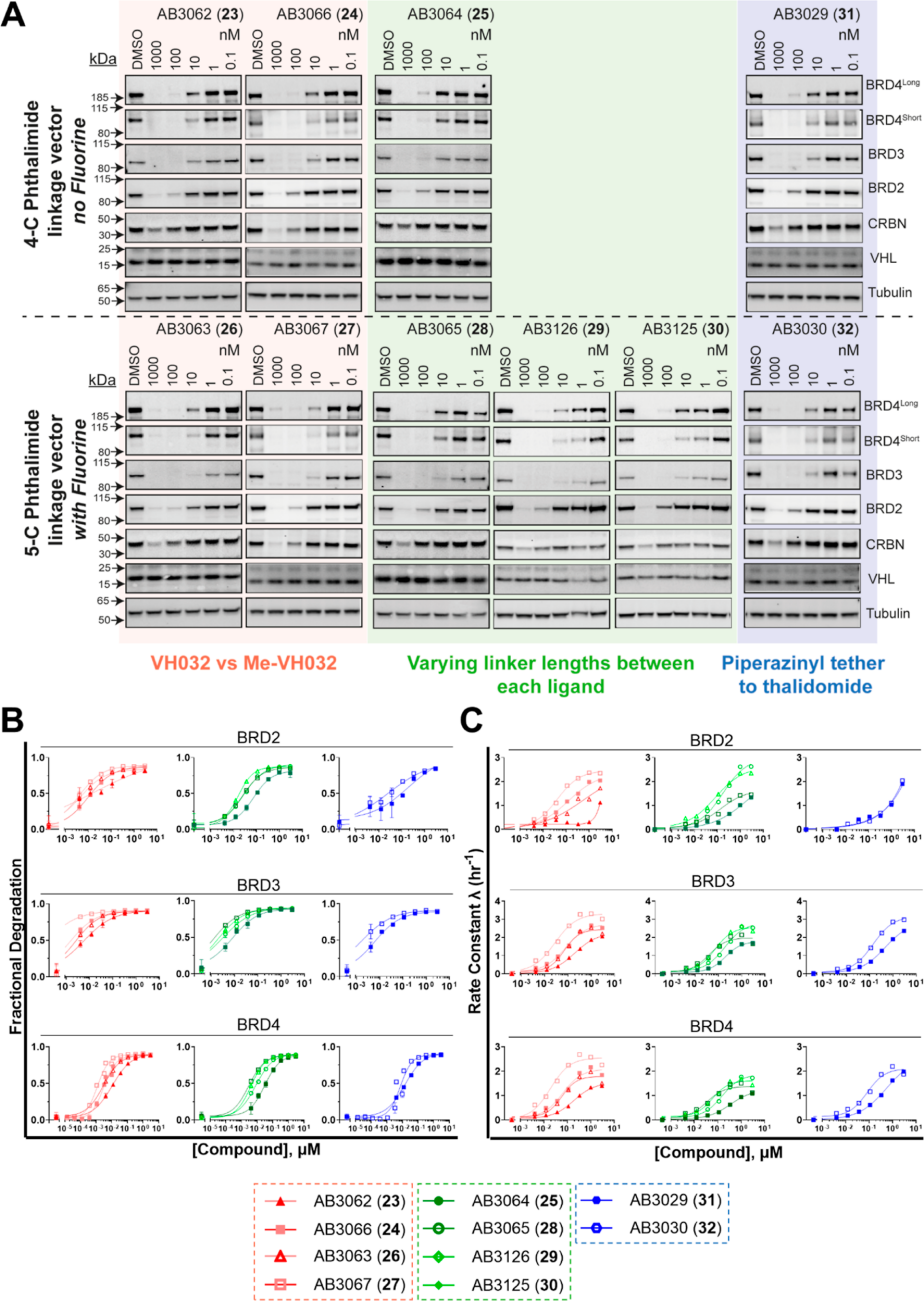
Evaluation of cellular BET degradation for heterotrivalent PROTACs **23–32** in HEK293 cells. (*A*) Western blot data for BET, CRBN and VHL protein levels monitored from 1 μM to 100 pM compound treatments over 6 h in HEK293 cells. Blots arranged with nonfluorinated compounds **23**–**25** and **31** on top, and fluorinated compounds **26**–**30** and **32** on bottom. Bands are normalized to tubulin and vehicle control (DMSO) to derive DC_50_ values that enable rank ordering of each PROTAC. (B) Degradation potency and (C) rate constants extracted from kinetic degradation profiles of HEK293 HiBiT-BRD2, HiBiT-BRD3, or HiBiT-BRD4 cells treated with 3 μM to 4 nM compound. Compounds with fluorine represented by open symbols, compounds with no fluorine represented by closed symbols. Mean ± S.D.; *n* = 2 biological replicates (six technical replicates) (BRD4) or *n* = 1 biological replicate (three technical replicates) (BRD2 and BRD3). *D*_max 50_ and λ_max_ values are tabulated in Tables S2 and S3, respectively, with 95% CI.

With the library of new heterotrivalent PROTACs in hand, we proceeded to evaluate the BET degradation profiles in cells after treatment of compounds **23**–**32**. We treated HEK293 cells with compounds **23**–**32** to monitor levels of on-target BET degradation, and off-target CRBN and VHL degradation (Figure 4A, Table 2).

**Table 2:**
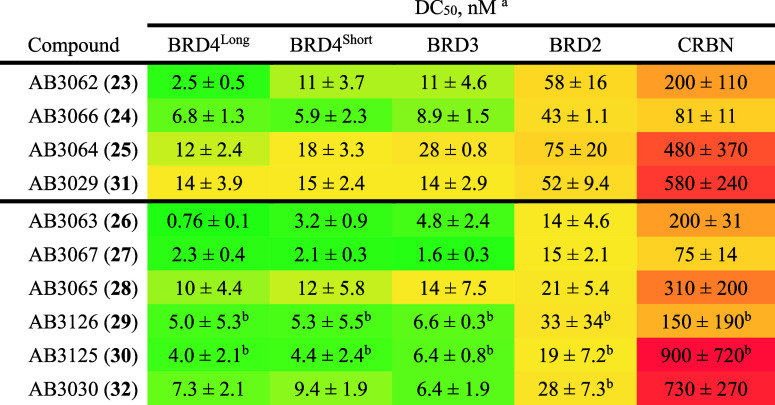
Quantification of Western-Blot Degradation Profile With Heterotrivalent PROTACs 23–32 Against BET Proteins and CRBN in HEK293 Cells

a Calculated as mean (±S.E.M) from three independent biological experiments.

b Calculated as mean (±S.D.) from two independent biological repeats. Data is color scaled for lowest (green), median (yellow), and highest (red) DC_50_ values.

Gratifyingly, all compounds were able to potently degrade all BET proteins, each showing a preference for BRD4 (long and short isoforms) and BRD3 over BRD2. There was no observed degradation of VHL, however, each compound displayed degradation of CRBN to varying extents at the top treatment concentration of 1 μM, and in some cases also at 100 nM (Figure 4A). Strikingly, fluorinated compounds with 5-C tethering to the phthalimide ring (**26**–**28** and **32**) were an average of threefold more potent at degrading each BET protein when compared to their nonfluorinated 4-C tethered matched pairs (**26** vs **23**, **27** vs **24**, **28** vs **25**, and **32** vs **31**, respectively, Figure 4A, Table 2).

Out of the entire series, compounds AB3063 (**26**) and AB3067 (**27**), were the most potent degraders of each BET protein, with DC_50_ values of 0.76 nM and 2.3 nM for BRD4^Long^; 3.2 nM and 2.1 nM for BRD4^Short^; 4.8 nM and 1.6 nM, for BRD3; and, 14 nM and 15 nM, for BRD2, respectively (Table 2). **26** and **27**, which have 5-C tethering, were an average of 3.4-fold more potent than their 4-C tethered counterparts AB3062 (**23**) and AB3066 (**24**), respectively. Interestingly, the additional methyl group on the benzylic position of the VHL ligand present in **27** and **24** had no significant effect on BET degradation when comparing to their nonmethylated matched pairs **26** and **23** respectively. However, the modification did lead to an unfavorable ∼2.6-fold increase in CRBN degradation.

Moreover, when investigating the changes in linker length between each of the three ligands, there is a slight preference for a longer linker between both E3 ligase ligands, and the BET ligand for BET degradation. In the case of BRD4^Long^, removing a PEG unit from each of the VH032-JQ1 and thalidomide-VH032 (and therefore, thalidomide-JQ1) linkers from **26** to form AB3125 (**30**), results in an unfavorable 5.3-fold decrease in degradation, while leading to a much minor ∼1.3-fold decrease for the degradation of the other BET proteins (Table 2). The shorter linker of **30** however, showed a favorable 4.5-fold decrease in CRBN degradation vs **26**, making it also an attractive compound, meeting the criteria for dialling out potential E3 ligase degradation.

Finally, comparing the 4-C and 5-C piperazinyl tethering vectors of AB3029 (**31**) and AB3030 (**32**), respectively, with their closest aniline tethered matched pairs **23** and **26**, respectively, we see that the piperazinyl tethered compounds were on average ∼threefold weaker at degrading the BET proteins than their respective aniline tethered matched pairs. Encouragingly, however, **31** and **32** did show a 2.9 to 3.7-fold weaker degradation of CRBN compared with **23** and **27**, respectively (Table 2). It is important to note that **31** and **32** have the longest thalidomide-VH032/JQ1 linkers of the entire series, differing to thalidomide-VH032/JQ1 linkers of **23** and **26** by just two methylene groups in length and may also be a contributing factor to the changes in observed degradation potency.

Next, we sought to use the same live cell kinetic degradation assay set up as described above to evaluate degradation potency (*D*_max 50_) and degradation rate (Rate Constant λ, hr^–1^) of compounds **23**–**32** in HiBiT-BRD2, HiBiT-BRD3, and HiBiT-BRD4 HEK293 cell lines. This provides an orthogonal degradation assay to Western blot, enables quantification of degradation rate, and allows for comparison with the initial compounds **1** and **2** (Figure 4B, kinetic traces provided in Figure S2). Reassuringly, the *D*_max 50_ values (Tables 3 and 2) correlated well with DC_50_ values (Table 2) from Western blot analysis. Compound **27** was shown to be the most potent degrader of BRD3 and BRD4 out of the series, giving a subnanomolar *D*_max 50_ value of 85 pM for BRD3 and 640 pM for BRD4, while giving a low nanomolar *D*_max 50_ value of 2 nM for BRD2 (Table 3). This directly correlates with **27** also having the greatest degradation rate of BRD3 and BRD4, with a λ_max_ of 2.68 h^–1^ for BRD4, 3.31 h^–1^ for BRD3 (which was the highest degradation rate of any compound in the series) and 2.37 h^−1^ for BRD2 (Tables 3 and S3).

**Table 3:**
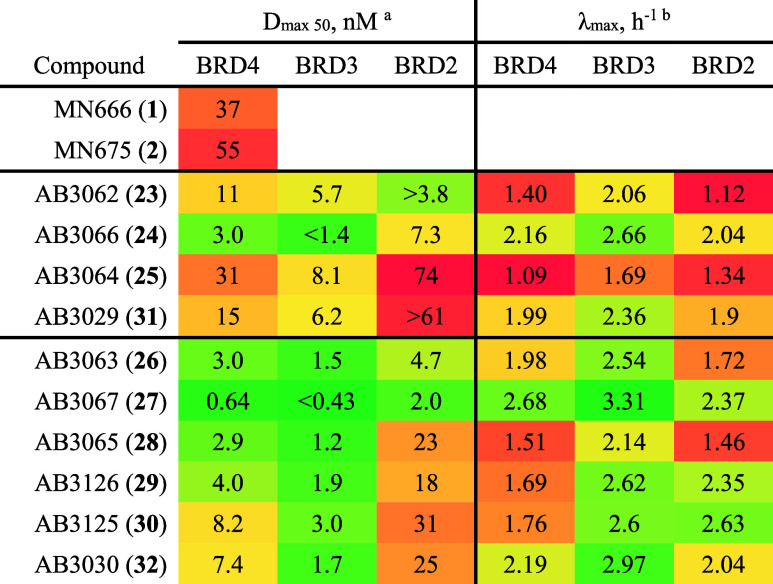
Quantification of Live-Cell Degradation Parameters *D*_max_ 50 and Degradation Rate (λ_max_) With Heterotrivalent PROTACs 1, 2, and 23–32 Against BET Proteins in HiBiT-BRD Knock-In HEK293 Cells

a Data is color scaled for lowest (green), median (yellow), and highest (red) *D*_max 50_ values. In cases where the fit of the curve was not sufficient to enable calculation of a 95% CI for either the upper or lower bound, the D _max 50_ value was reported as “greater than” or “less than” the CI bound which could be determined.

b Data is color scaled for highest (green), median (yellow), and lowest (red) λ_max_, h^–1^ values.

Encouragingly, all second generation heterotrivalent PROTACs performed 1.3 to 63-fold, and 1.6 to 78-fold better at degrading BRD4 than **1** and **2**, respectively (Table 3). The benefit of the amide-to-ester switch in the linker attachment point to JQ1 is evident when comparing molecular matched pairs, amide **1** and ester **23**. Ester **23** gave a 3.4-fold increase in the degradation of BRD4 than amide **1** (*D*_max 50_ = 11 vs 37 nM, respectively) (Table 3). BRD4 degradation was increased by a further 3.7-fold when further switching from the 4-C tethering to thalidomide of **23** to the 5-C tethering of 6-fluorothalidomide in **26** (*D*_max 50_ = 11 vs 3.0 nM, respectively), showing that there is a positive combinatory effect of applying each modification to the parent **1**. A similar combinatory effect is seen when applying both amide-to-ester substitution and 5-C tethering of 6-fluorothalidomide to **2**, to give molecular matched pair **30**, a compound which degrades BRD4 6.7-fold more than **2** (*D*_max 50_ = 8.2 vs 55 nM, respectively).

### 2.5 Further Biological Evaluation of Lead Heterotrivalent PROTACs **26** & **27**

**Figure 5:**
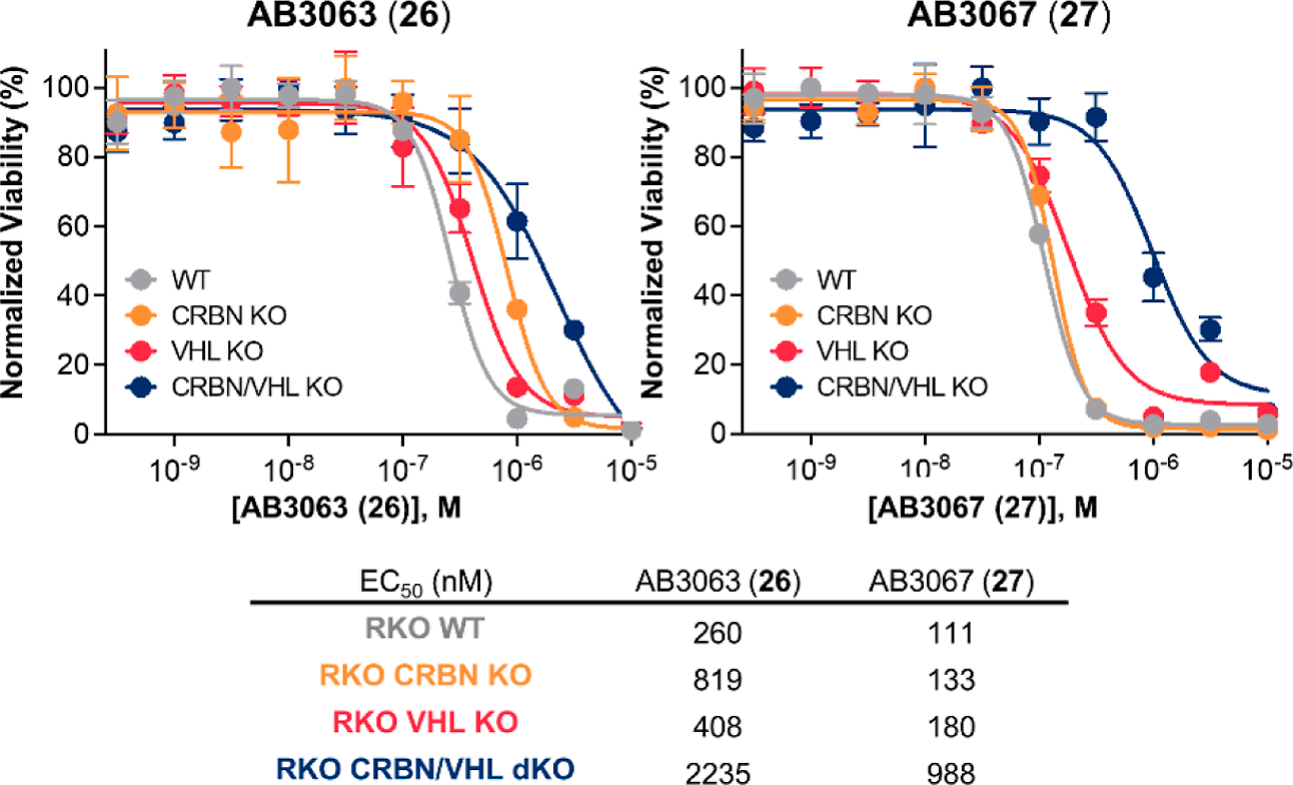
Cell viability assay with **26** and **27** in BET sensitive WT and CRBN/VHL KO RKO cell lines. Effect on cellular proliferation of **26** (left) and **27** (right) after 316 pM to 10 μM treatment in WT, CRBN KO, VHL KO or CRBN/VHL dKO RKO cell lines. Mean ± S.D.; *n* = 3 biological replicates. EC_50_ values are tabulated below and in Table S5 with 95% CI.

To determine whether our heterotrivalent PROTACs could drive antiproliferative effects, we evaluated the cytotoxicity of compounds **23**–**32** in cell viability assays performed in three different cell lines: RKO, KBM7 and K562 (Figure S3 and Table S4). The results of this assay show that the cytotoxicity of the compounds, as measured by their EC_50_ values, follows the same trends observed in the Western blot and HiBiT data. This further indicates that degradation is the major driver of cytotoxicity for these compounds, and that most of the second generation heterotrivalent PROTACS are potent cytotoxic compounds, with **26** and **27** standing out as the most cytotoxic across cell lines (Figure S3 and Table S4). The degradation profiles from both Western blot and live cell HiBiT assay, and the cell viability data indicate that compounds **26** and **27** are the most potent BET degraders of the series. We therefore wanted to further discriminate between these two compounds by evaluating their cell antiproliferation activity in BET sensitive RKO wild-type (WT) and CRBN and/or VHL KO/dKO cell lines (Figure 5 and Table S5).

We treated each RKO cell line with varying concentrations of either **26** or **27**. Strikingly, **26** and **27** showed ∼ninefold greater antiproliferation in RKO WT cells when compared to RKO dKO cells with EC_50_ values of 260 nM and 111 nM, vs 2235 nM and 988 nM, respectively, with **27** giving the largest window between WT and dKO cells (Figure 5). Interestingly, **27** gave a greater antiproliferative effect in each cell line with a 2.3-fold greater effect in WT, VHL KO, and VHL/CRBN dKO cells, and a 6.2-fold greater effect in CRBN KO cells when compared to **26** (Figure 5 and Table S5). Importantly, the antiproliferative effect of **27** in CRBN KO and VHL KO cells was comparable (within twofold) with WT cells (EC_50_ = 133, 180, and 111 nM, respectively). **27** was less effective in VHL KO cells compared to CRBN KO, suggesting that degradation is more VHL-driven. Conversely, for **26**, there is more discrepancy in antiproliferation, especially in CRBN KO cell lines over WT cells (Figure 5 & Table S5). Additionally, **26** gave 3.2-fold less antiproliferation in CRBN KO cells than in WT cells and 1.6-fold less than in VHL KO cells, suggesting that the mode-of-action of **26** is more CRBN-driven, contradictory to what we see for **27**. As **26** and **27** are molecular matched pairs in all ways except for the additional benzylic methyl group present in the VH032 ligand of **27**, this switch in selectivity is likely due to an increased binary binding affinity for VHL by **27** relative to **26**.

**Figure 6:**
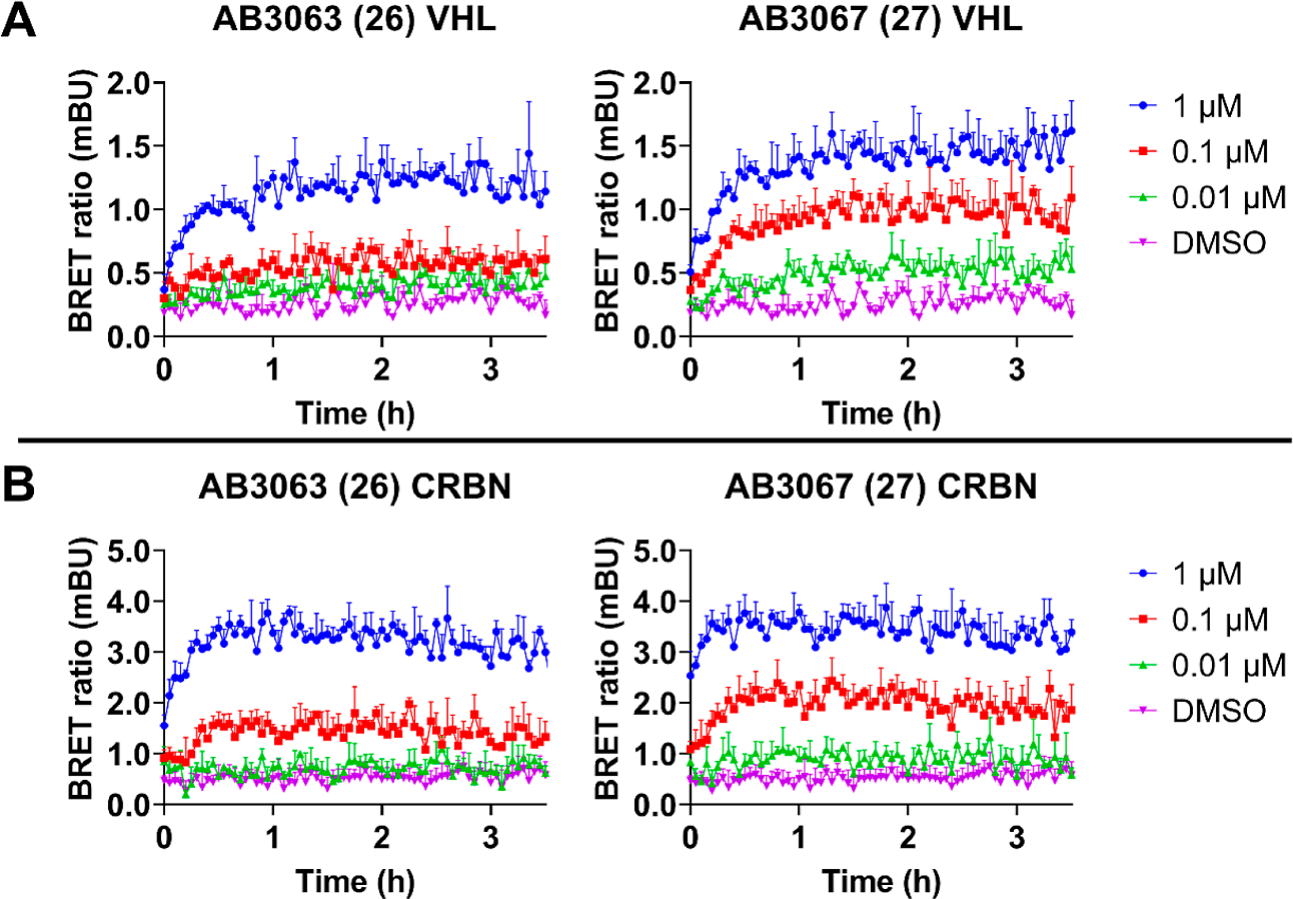
Live cell ternary complex formation between VHL or CRBN with **26** or **27** and BRD4. NanoBRET kinetic ternary complex formation in endogenous HiBiT-BRD4 HEK293 cells stably expressing LgBiT and transiently expressing (*A*) HaloTag-VHL or (*B*) HaloTag-CRBN. Cells were pretreated with 1 μM of proteasome inhibitor MG132, and subsequently treated with 0.01, 0.1, and 1 μM of **26**, **27** or DMSO control. Donor and acceptor signal was continuously monitored for 3.5 h after compound addition. *N* = 1 biological replicate, data is presented as mean values with error bars representing the S.D. of technical triplicates.

To investigate the differences in ternary complex formation induced by either **26** or **27** between BRD4 and VHL/CRBN, we monitored live cell ternary complex formation using NanoBRET (Figure 6).^23^ In this assay, the endogenously tagged HiBiT-BRD4 complemented with LgBiT served as the energy donor and transiently expressed HaloTag-CRBN or HaloTag-VHL served as the energy acceptor. A NanoBRET signal is observed when the donor and acceptor are in close proximity, making it ideal to measure cellular ternary complex formation and stability.^11,23^ We treated HEK293 HiBiT-BRD4 (LgBiT stable) cells that were transiently expressing either HaloTag-VHL or HaloTag-CRBN with a pretreatment of proteasome inhibitor, MG132,^39^ followed by varying concentrations of **26** and **27**, and monitored the NanoBRET signal over 3.5 h (Figure 6).

Encouragingly, both **26** and **27** can engage and form ternary complexes between BRD4 and either VHL or CRBN, with each compound showing slightly faster association kinetics for CRBN, plateauing after just 30 min. Interestingly, **27** gave more robust dose–response with both VHL and CRBN than **26**, suggesting that **27** may form a more stable and/or more highly populated ternary complex. This is likely to be one of the reasons why **27** is a more rapid and potent BRD4 degrader, evidencing that increased ternary complex population and stability positively correlates with the amount of ubiquitination and subsequent degradation.^23^

**Figure 7:**
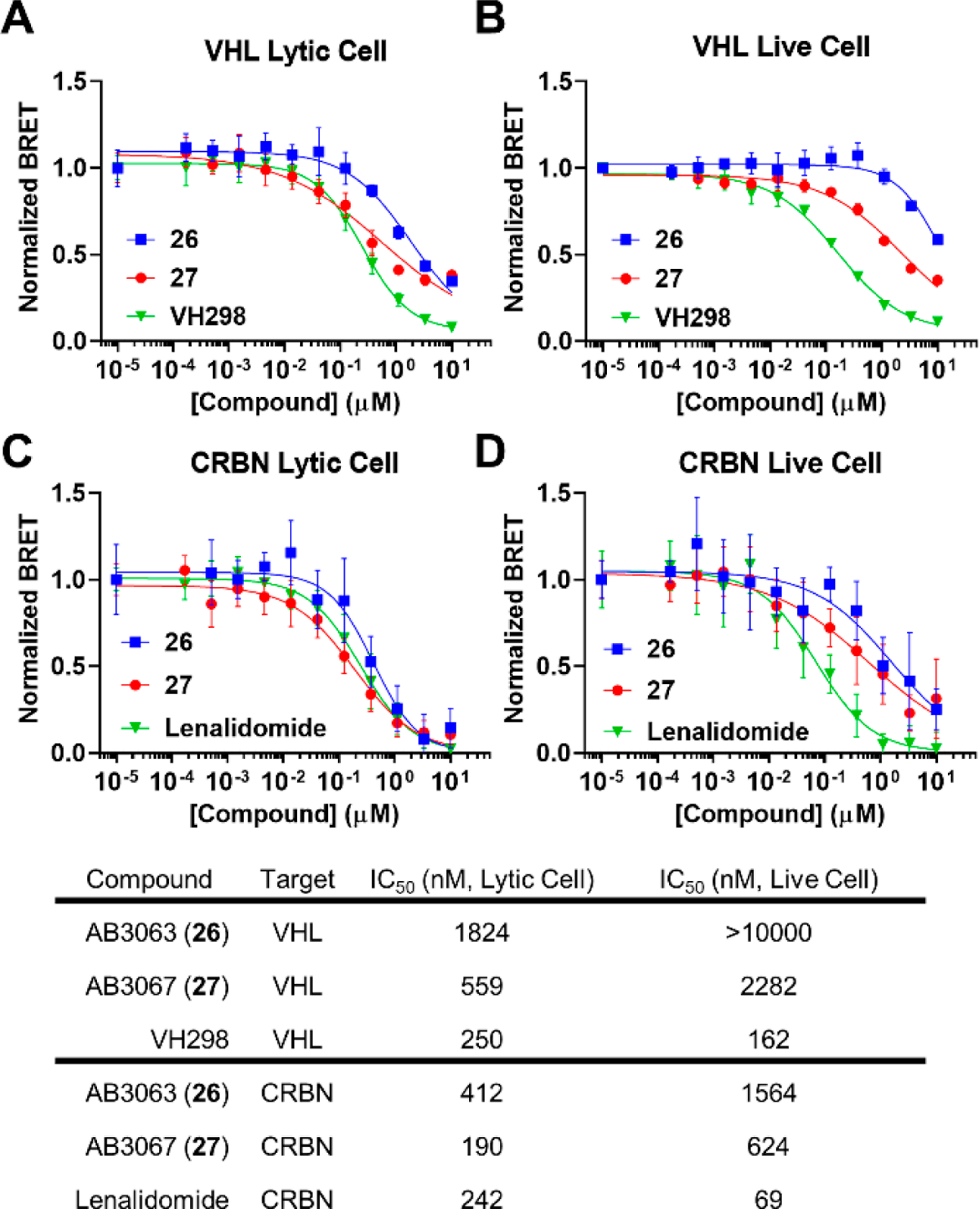
NanoBRET lytic and live cell target engagement assay of **26** and **27**. (*A*) & (*B*) Competitive displacement profiles of HEK293 cells transiently transfected with NanoLuc-VHL, which are incubated with a VHL fluorescent tracer in the presence of serial dilutions of **26**, **27** or VH298 in cells lysed with digitonin (*A*) or in live cells for 2 h (*B*). (*C*) & (*D*) Competitive displacement profiles of HEK293 cells transiently transfected with NanoLuc-CRBN which are incubated with a CRBN fluorescent tracer in the presence of serial dilutions of **26**, **27** or lenalidomide in cells lysed with digitonin (C) or in live cells for 2 h (D). Data are represented as NanoBRET ratios normalized to 0 μM compound. Error bars are expressed as S.D. of the mean of *n* = 2 biological replicates (each consisting of 3 technical replicates) (*A*) & (*B*) or *n* = 3 biological replicates (each consisting of 3 technical replicates) (*C*) & (*D*). IC_50_ values are tabulated below for indicated target, compound, and assay format.

To further understand whether these improvements may be attributed to enhanced intracellular availability of **27** relative to **26**, we assessed the binary target engagement of **26** and **27** to either CRBN or VHL using a lytic and live cell NanoBRET target engagement assay (Figure 7).^23,40^

Competitive displacement of the VHL tracer molecule by **26** and **27** in lytic format showed that engagement of VHL was >threefold stronger by **27** than **26** (IC_50_s = 559 nM and 1.82 μM, respectively) (Figure 7). **27** differs to **26** only by an extra methyl group at the benzylic position of its VHL ligand VH032, a modification known to give rise to >twofold binding affinity to VHL.^27−29^ When in live cell format, **26** and **27** are >5.5-fold and fourfold weaker, respectively, at engaging VHL (IC_50_s = 2.3 μM and >10 μM, respectively), with **27** now showing >4.4-fold (vs > threefold in lytic format) stronger binding with respect to **26** (Figure 7). This increased difference in binding affinity to VHL of **27** relative to **26** when comparing the live cell to lytic cell data suggests that **27** has a higher cell permeability than **26**. Interestingly, although comprising of the same fluorothalidomide-based ligand, **27** was able to engage CRBN > twofold more strongly than **26** (IC_50_s = 190 nM and 412 nM, respectively) in lytic cell format (Figure 7). In live cell format, **27** engages CRBN 2.5-fold more strongly over **26**, again indicated that **27** is more cell permeable than **26**.

Taken together, out of the data presented above for all second generation compounds qualified compounds **26** and **27** as most potent degraders, with **27** emerging as the fittest of the two.

### 2.6 Design, Synthesis, and Characterization of a Heterotetravalent PROTAC

**Scheme 5:**
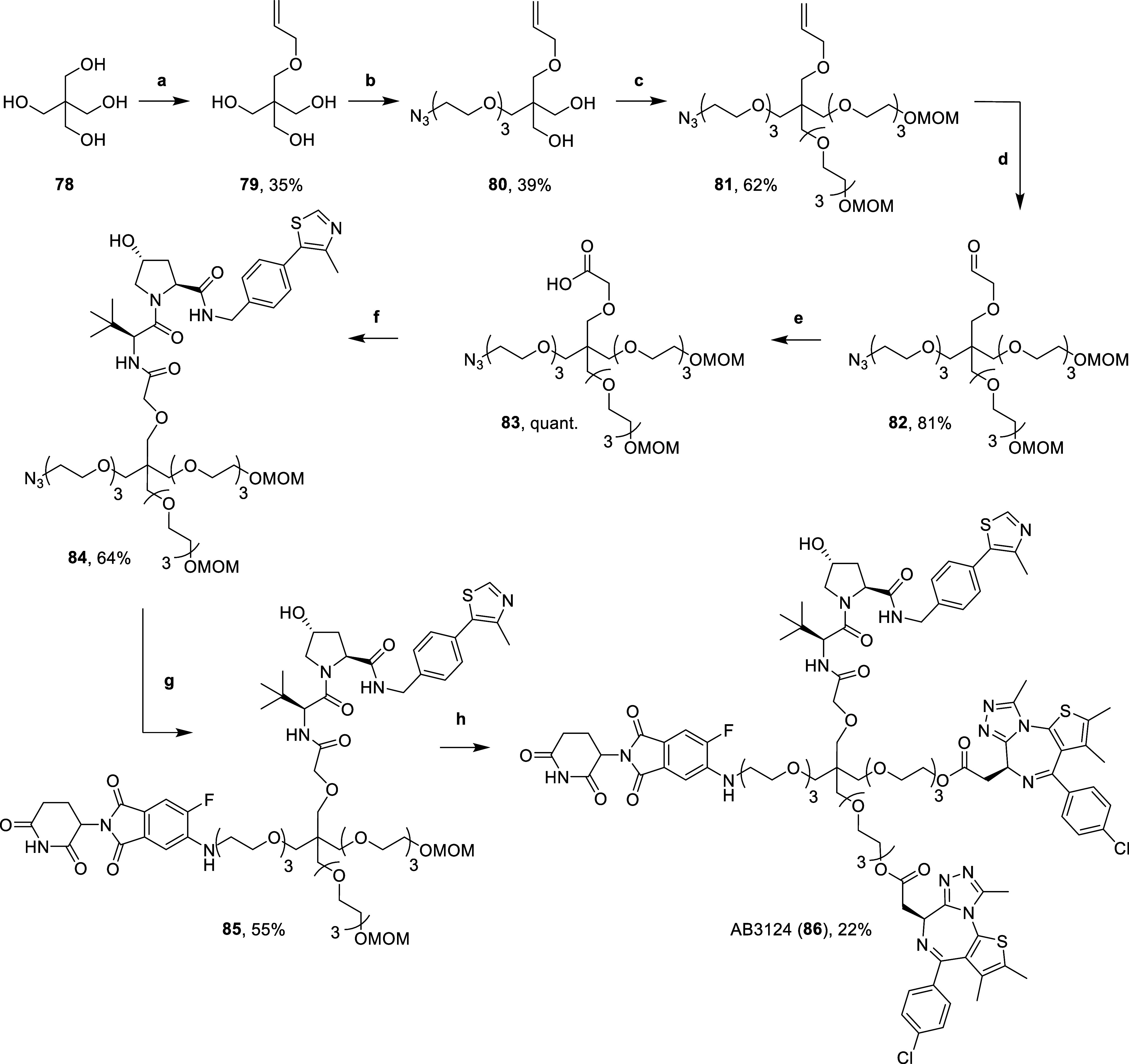
Synthesis of Heterotetravalent PROTAC AB3124 (**86**) Reaction conditions: (a) (i) NaH, DMF, 0 °C, 15 min, (ii) allyl bromide, r.t., 16 h; (b) (i) NaH, DMF, r.t., 30 min, (ii) **7**, DMF, 60 °C, 16 h; (c) (i) NaH, DMF, r.t., 30 min, (ii) **40**, DMF, 60 °C, 16 h; (d) OsO_4_, NaIO_4_, 2,6-lutidine, dioxane, H_2_O, r.t., 16 h; (e) 2-methyl-2-butene, NaH_2_PO_4_, NaClO_2_, *t*-BuOH, H_2_O, r.t., 16 h; (f) **14**, HATU, DIPEA, DMF, r.t., 2 h; (g) (i) H_2_, 10% Pd/C, MeOH, r.t., 16 h, (ii) **65**, DIPEA, DMSO, 90 °C, 16 h; (h) (i) 4 N HCl in dioxane, MeOH, r.t., 3 h, (ii) 22*, DIPEA, DCM, r.t., 16 h.

With the encouraging results presented by the heterotrivalent PROTAC series, we wanted to further investigate the chemical space and synthesize a compound which would more closely resemble SIM1. We aimed to retain the avidity and BET bivalency that SIM1 displays,^11^ but now adding the ability to recruit two E3 ligases instead of just one. The so-called “heterotetravalent PROTAC” would be a combination of heterotrivalent PROTAC AB3063 (**26**) and BET-bivalent, trivalent PROTAC SIM1. We therefore decided to functionalize from the methyl group of the central quaternary carbon present in the linker of SIM1 as a potential vector to recruit CRBN by adding another linker tethered to thalidomide. We chose to synthesize a linker which would again allow for: simple amide coupling to the VH032; future S_N_Ar to a fluorothalidomide derivative; and diesterification to JQ1 (Scheme 5).

The synthesis follows a similar route to the one used for heterotrivalent PROTACs (Schemes 2 & 3). First, the tetrafunctional, pentaerythritol (**78**) was monoalkylated by first deprotonating with sodium hydride in DMF, followed by the addition of allyl bromide to yield the allyl ether triol **79**. Triol **79** was then carefully deprotonated with sodium hydride (1.2 equiv) in DMF, before heating to 60 °C with azido mesylate **7** (1 equiv) to yield ether **80**. Double deprotonation of diol **80** with excess sodium hydride in DMF at 0 °C before subsequent addition of mesylate **40** and heating at 60 °C yielded **81**. Then, alkene **81** was oxidatively cleaved with sodium periodate, 2,6-lutidine and a catalytic amount of osmium tetroxide in dioxane and water to yield aldehyde **82**. Aldehyde **82** underwent a Pinnick oxidation by treating with 2-methyl-2-butene, monobasic sodium phosphate and sodium chlorite in *tert*-butanol and water to yield carboxylic acid **83** in quantitative yields. Next, acid **83** was coupled to VH032-amine (**14**) with HATU and DIPEA in DMF to yield amide **84**. Then, the azide of **84** was reduced with a suspension of 10% Pd/C in methanol, under an atmosphere of hydrogen gas. The intermediate amine subsequently underwent an S_N_Ar reaction with 5,6-difluorothalidomide **65**, by heating with DIPEA in DMSO at 90 °C to yield 5-substituted-6-fluoro aniline **85**. Finally, MOM protecting groups of **85** were hydrolyzed with 4 N hydrochloric acid in dioxane and methanol. The subsequent diol was immediately conjugated to an intermediate acid chloride (22*, synthesized in Scheme 3), formed after treating (+)-JQ1-acid (**22**) with thionyl chloride in DCM, to afford the diester of heterotetravalent PROTAC AB3124 (**86**) (Scheme 5).

**Figure 8:**
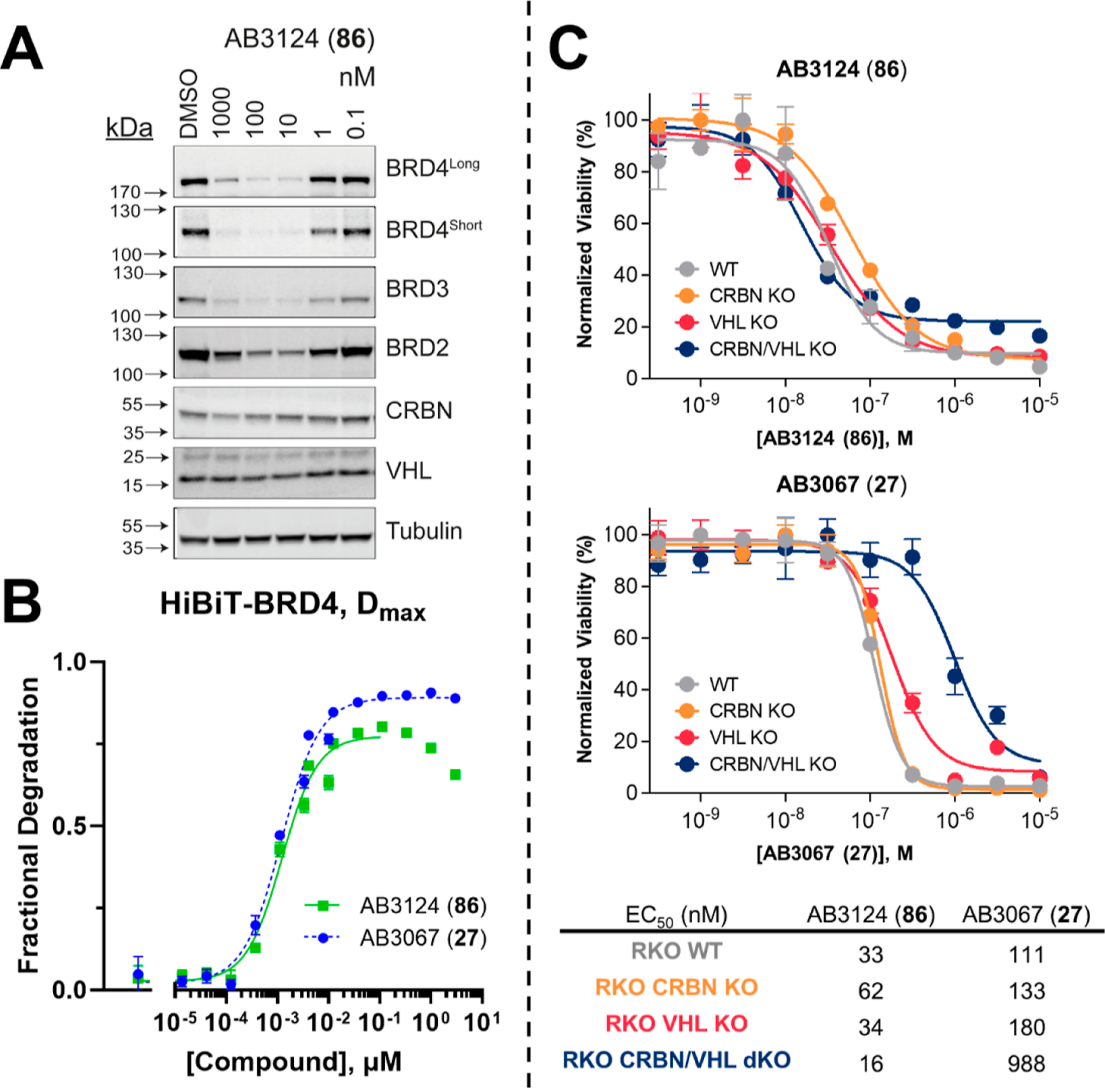
Cellular evaluation of heterotetravalent PROTAC AB3124 (**86**). (*A*) Western blot data for BET, CRBN and VHL protein levels monitored after 1 μM to 100 pM treatments of **86** over 6 h in HEK293 cells. Bands are normalized to tubulin and vehicle control (DMSO) to derive DC_50_ values that enable rank order of each PROTAC. (*B*) Plots of *D*_max_ expressed as fractional degradation versus concentration of **86** and **27** from live cell degradation kinetics in HiBiT-BRD4 CRISPR knock in HEK293 cells. Cells were treated with DMSO and a threefold serial dilution of **86** and **27** over a concentration range of 5 pM to 3 μM in HiBiT-BRD4 knock in cells. Data points ≥333 μM for **86** were excluded from the data fitting due to appeared onset of hook-effect. Mean ± S.D.; *n* = 2 biological replicates (each consisting of 3 technical replicates). (*C*) Cell viability assay with **86** and **27** in BET sensitive WT and CRBN/VHL KO RKO cell lines. Cell antiproliferation of **86** (top) and **27** (bottom) after 316 pM to 10 μM treatment in WT, CRBN KO, VHL KO or CRBN/VHL dKO RKO cell lines. Mean ± S.D.; *n* = 3 biological replicates. EC_50_ values are tabulated below and in Table S5 with 95% CI.

We then moved to assess the degradation profile of **86** by Western blot and live cell kinetics. First, we treated HEK293 cells with varying concentrations of **86** and monitored intracellular levels of on-target BET, and off-target CRBN and VHL degradation (Figure 8A, Table 4).

**Table 4:** Quantification of the Degradation Profile of Heterotetravalent PROTAC AB3124 (**86**) and Heterotrivalent PROTACs AB3063 (**26**) and AB3067 (**27**) Against BET Proteins and CRBN in HEK293 Cells

	Western Blot DC_50_ (nM)^a^					HiBiT *D*_max 50_ (nM)^b^	
Compound	BRD4^Long^	BRD4^Short^	BRD3	BRD2	CRBN	BRD4	95% CI
AB3124 (**86**)	2.2 ± 0.1	2.9 ± 0.3	3.5 ± 0.3	1.0 ± 0.2	113 ± 3.2	1.1	0.91 to 1.4
AB3063 (**26**)	0.76 ± 0.1	3.2 ± 0.9	4.8 ± 2.4	14 ± 4.6	200 ± 31	3.0	2.6 to 3.3
AB3067 (**27**)	2.3 ± 0.4	2.1 ± 0.3	1.6 ± 0.3	15 ± 2.1	75 ± 14	0.64	0.50 to 0.80

a Calculated as mean (±S.E) from three independent biological experiments.

b Calculated as mean from two independent biological experiments.

Compound **86** was able to potently degrade all BET proteins, with DC_50_ values of 2.2 nM for BRD4^Long^; 2.9 nM for BRD4^Short^; 3.5 nM for BRD3; and 1 nM for BRD2. Similarly, to the heterotrivalent PROTAC series, **86** showed no observed degradation of VHL, while showing degradation of CRBN at high concentrations (DC_50_ = 113 nM). In contrast with heterotrivalent PROTACs **23**–**32**, **86** showed potent and preferential degradation for BRD2, albeit incomplete (*D*_max_ ∼ 80%) and showing an earlier onset of the hook effect at 1 μM (Figure 8A). This hook effect can also be seen to a weaker extent in BRD4^Long^ blot (Figure 8A). The earlier onset of the hook effect is likely due to **86** inhibiting BRD2 and BRD4 more strongly due to its extra linkage to a second JQ1 molecule and potential BET bivalency. The switch in BET protein preference and increase in potency that **86** has for degrading BRD2 compared with the heterotrivalent compounds is likely due to the extra JQ1 “arm”. Trivalent PROTAC SIM1, also shows preferential degradation of BRD2 by simultaneously engaging both BD1 and BD2 of the same BRD2 protein with high avidity, forming a stable 1:1:1 (BRD2^BD1–BD2^:SIM1:VHL) ternary complex with VHL.^11^ This is likely the reason for the observed switch in selectivity, especially when comparing heterotetravalent PROTAC **86** to its heterotrivalent matched pair **26** (Table 4).

In addition to the Western blot analysis, we assessed the live cell kinetic degradation displayed by **86** in HiBiT-BRD4 HEK293 cells (Figure 8B, Table 4). Strikingly, **86** showed near equipotent degradation of BRD4 with the best heterotrivalent degrader **27**, with a *D*_max 50_ value of 1.1 nM vs 0.6 nM. Interestingly, **86** was ∼threefold more potent than its trivalent counterpart **26**, with a *D*_max 50_ value of 1.1 nM vs 3 nM (Table 4). Although **86** displayed potent degradation of BRD4 in this assay, the compound gave a final *D*_max_ > 10% less than for **26** and **27**, with an observable hook-effect at treatment concentrations ≥333 nM (Figure 8). Furthermore, BRD4 degradation mediated by **86** was remarkably slow compared to the rapid degradation mediated by **27** (Figure S4), likely due to reduced cellular permeability.

Finally, we evaluated cell antiproliferation caused by **86** in BET sensitive RKO WT and CRBN and/or VHL KO/dKO cell lines. We treated cells with concentrations ranging from 316 pM to 10 μM of **86** and measured cell viability normalized to a DMSO control (Figure 8C and Table S5). **86** displayed a potent cell antiproliferation in WT, CRBN KO and VHL KO RKO lines, with EC_50_ values of 33, 62, and 34 nM, respectively, hence suggesting that both VHL and CRBN driven degradation occurred. When comparing the EC_50_ values of **86** with that of the most cytotoxic heterotrivalent PROTAC, **27**, the antiproliferative effect of **86** was 3.4-fold greater in WT cells (EC_50_ = 33 vs 111 nM); 2.1-fold greater in CRBN KO cells (EC_50_ = 62 vs 133 nM); and 5.3-fold greater in VHL KO cells (EC_50_ = 34 vs 180 nM); confirming the enhanced potency of the compound. Surprisingly, **86** also had a marked antiproliferative effect on VHL/CRBN dKO cells (EC_50_ = 15 nM), which was slightly more potent, albeit only twofold, compared to both WT and single VHL KO cells (EC_50_ = 33 nM). This might suggests that **86** is acting more strongly as a potent bivalent BET inhibitor in the absence of both E3 ligases.

### 2.7 Further Biological Characterization of AB3067 (**27**)

**Figure 9:**
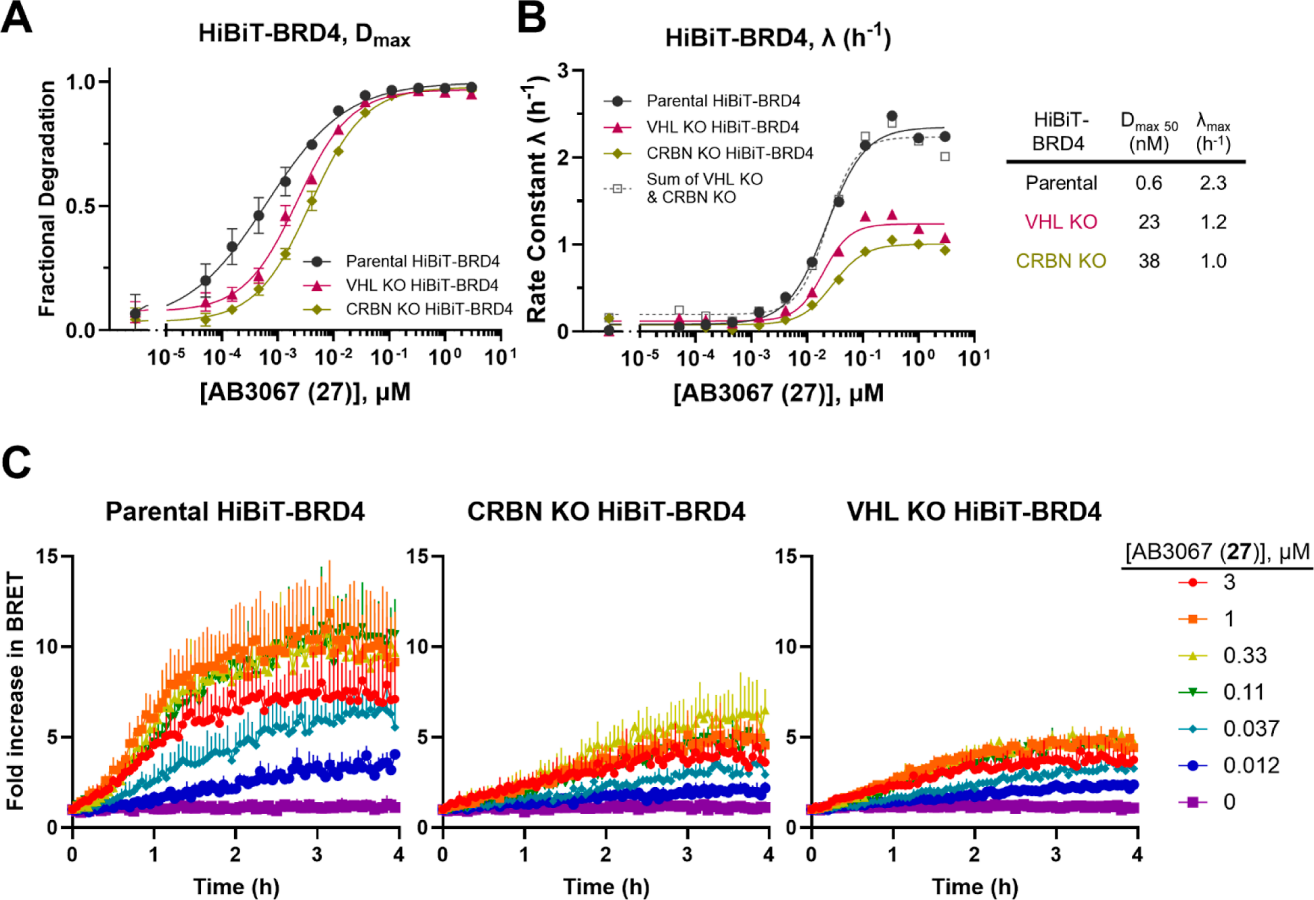
Degradation and ubiquitination profiles for AB3067 (**27**) in HiBiT-BRD4 CRISPR knock-in HEK293 cells with/without CRBN or VHL knocked out. Plots of (*A*) *D*_max_ expressed as fractional degradation and (*B*) rate constant λ (h^–1^) versus concentration of **27** from live cell degradation kinetics in HiBiT-BRD4 CRISPR knock in HEK293 cells with normal E3 ligase expression or with a CRBN or VHL KO. Cells were treated with DMSO and a threefold serial dilution of **27** over a concentration range of 5 pM to 3 μM. *N* = 2 biological replicates, a single representative experiment is shown. Error bars in A represent S.D. of technical triplicates. (*C*) Ubiquitination plots of HiBiT-BRD4 parental (left), with CRBN KO (middle), and with VHL KO (right) CRISPR knock-in HEK293 cells. Cells were first transiently transfected with HaloTag-Ubiquitin and were then treated with DMSO and a threefold serial dilution of **27** over a concentration range of 12 nM to 3 μM. The BRET signal was then measured at regular time points over 4 h. *N* = 3 biological replicates, a single representative experiment is shown. Error bars in (*C*) represent S.D. of technical triplicates.

After profiling all heterotrivalent (**23**–**32**) and heterotetravalent (**86**) compounds in various biological assays, we established AB3067 (**27**) as the most suitable heteromultivalent compound to take forward for further study. We wanted to further assess the relative contribution of both VHL and CRBN to degrade BET proteins with **27**. To this end, we investigated live cell kinetic degradation of endogenous HiBiT-BRD4 in the presence of either a VHL or CRBN KO (Figure 9).

In each VHL and CRBN KO HiBiT-BRD4 cell line, the *D*_max 50_ values for the degradation of BRD4 were 40 and 60-fold less, respectively, than in the parent HiBiT-BRD4 cells (*D*_max 50_ = 23 vs 0.6 nM, and 38 vs 0.6 nM, respectively, Figure 9A). This implies that **27** is almost equally reliant on VHL and CRBN to drive the degradation of BRD4, but performing slightly worse in CRBN KO cells than VHL KO or parental cells, and so showing a slight preferential reliance on CRBN. The rate of degradation (λ) for both VHL and CRBN KO cell lines is twofold slower than in parental cells (λ_max_ = 1.2 and 1.0 h^–1^, vs 2.3 h^–1^, Figure 9B). Remarkably, the sum of the rate constants from **27** in both VHL and CRBN KO cells equal the rate constant in the parental cells, indicating that both VHL and CRBN are contributing to the degradation of BRD4 in an additive fashion.

Next, we wanted to compare how intracellular ubiquitination levels in parental, CRBN KO, and VHL KO HiBiT-BRD4 cells differed (Figure 9C). To this end, we adopted a NanoBRET ubiquitination assay similar to the ternary complex assay described previously. In the NanoBRET ubiquitination assay, parental, CRBN KO, or VHL KO HiBiT-BRD4 cell lines were transiently transfected with HaloTag-Ubiquitin and treated with a dilution series of **27**.^11,40^ Ubiquitination of BRD4 in parental cells treated with **27** occurs more rapidly than in either the CRBN KO or VHL KO cells, and the parental cells also exhibit a larger magnitude in BRET fold-change. Taken together, this indicates that each ligase is contributing to ubiquitination and therefore helping to drive the degradation of BRD4 when cells are treated with **27**.

**Scheme 6:**
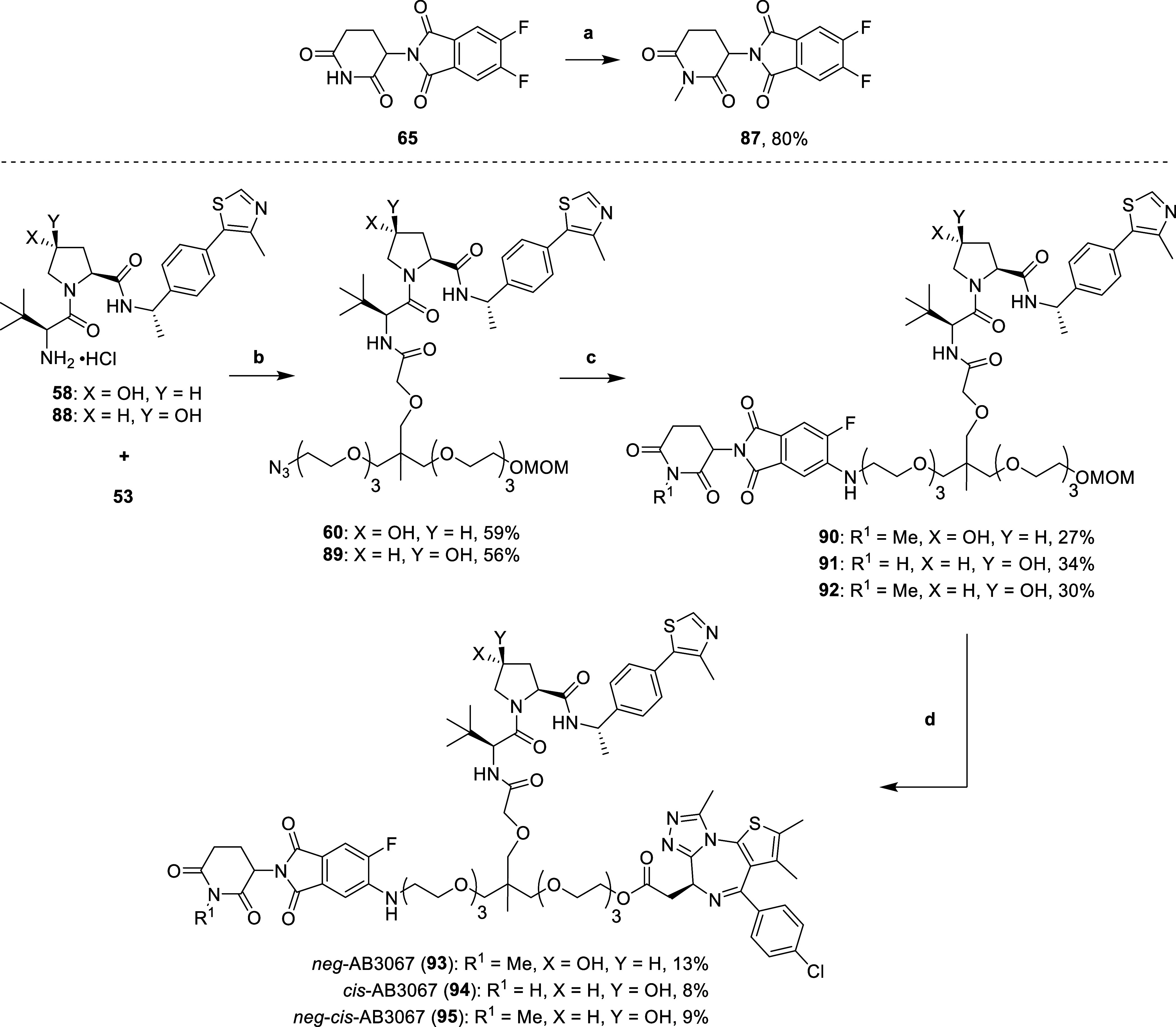
Synthesis of AB3067 (**27**) Control Compounds, **93–95** Reaction conditions: (a) MeI, K_2_CO_3_, DMF, 0 °C – r.t., 5.5 h; (b) HATU, DIPEA, DMF, r.t., 2 h; (b) (i) H_2_, 10% Pd/C, MeOH, r.t., 16 h, (ii) **87** or **65**, DIPEA, DMSO, 90 °C, 16 h; (c) (i) 4 N HCl in dioxane, MeOH, r.t., 3 h, (ii) 22*, DIPEA, DCM, r.t., 16 h.

Additionally, we sought to synthesize a series of control compounds which should complement the data in CRBN KO and or VHL KO cell lines and allow us to gain a better understanding of the contributions from each ligase. To this end, we synthesized control compounds *neg*-AB3067 (**93**), structurally identical to **27**, but with the glutarimide nitrogen of the CRBN ligand methylated, a modification well-known to block CRBN binding;^41^*cis*-AB3067 (**94**), a diastereomer of **27** bearing the *cis*-instead of *trans*-hydroxyproline group to abrogate binding to VHL;^16^ and *neg*-*cis*-AB3067 (**95**), a diastereomer of **93**, which has both the glutarimide methylated and the *cis*-hydroxyproline, to prevent both CRBN and VHL binding to provide a completely nondegrader control compound (Scheme 6).

The synthetic route for the control compounds **93**–**95** was similar to that of **27** (Schemes 2 & 3). First, glutarimide **65** was methylated after treatment with potassium carbonate and methyl iodide in DMF to yield methylated difluorothalidomide **87**. Next, carboxylic acid **53** was coupled to both Me-VH032-amine (**58**) and *cis*-Me-VH032-amine (**88**, synthesized according to literature procedures^38^) using HATU and DIPEA in DMF to yield amides **60** and **89**. Next, the azides of **60** and **89** were reduced with a suspension of 10% Pd/C in methanol, under an atmosphere of hydrogen gas. The intermediate amines subsequently underwent an S_N_Ar reaction with 5,6-difluorothalidomide derivatives **87** and **65**, by heating with DIPEA in DMSO at 90 °C to give anilines **90**–**92**. Finally, the MOM protecting groups of **90**–**92** were hydrolyzed with 4 N hydrochloric acid in dioxane and methanol. The subsequent primary alcohols were immediately conjugated to an intermediate acid chloride (22*, synthesized in Scheme 3), formed after treating (+)-JQ1-acid (**22**) with thionyl chloride in DCM, to afford the esters of control compounds **93**–**95** (Scheme 6).

**Figure 10:**
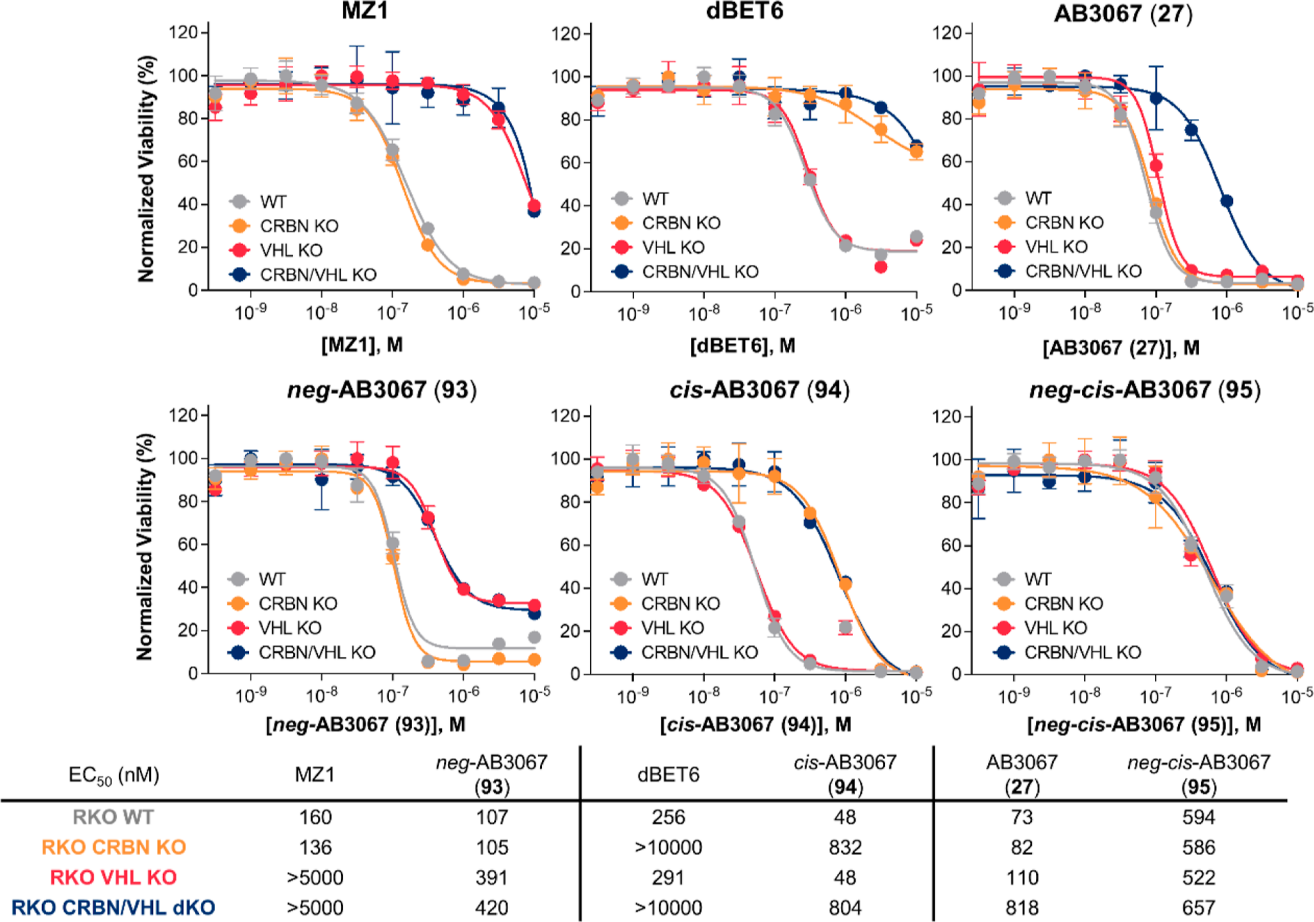
Cell viability assay with control compound **93–95** in BET sensitive wild-type and CRBN/VHL knockout RKO cell lines compared with, MZ1, dBET6 and **27**. Cell antiproliferation of heterobivalent (MZ1 and dBET6) and heterotrivalent (**27**) BET degraders, and control compounds **93**–**95** after 500 pM to 10 μM treatment in wild-type, CRBN knockout, VHL knockout or CRBN/VHL double knockout RKO cell lines. EC_50_ values are tabulated below and in Table S6 with 95% CI.

With compounds **93**–**95** in hand, we validated their on-target BRD4 degradation activity in parental (WT VHL and CRBN expression), CRBN KO, and VHL KO cell lines all expressing endogenous HiBiT-BRD4 (Figure S5). As expected, **95** showed no degradation of BRD4 in any of the cell lines, owing to its inability to engage either ligase, while **93** was inactive in VHL KO cells, and **94** was inactive in CRBN KO cells. While the potency of **93** was decreased relative to **27** in parental and CRBN KO cells, **94** exhibited an unexpected increase in degradation potency relative to **27** in both parental and VHL KO cells. We explored if the increase in potency of **94** relative to **27** might be due to alterations in permeability and/or intracellular accumulation by assessing target engagement of **94** and **27** with CRBN (Figure S6). **94** and **27** exhibited similar engagement profiles of CRBN in the lytic format (indicating that binding of CRBN is unaltered between the molecules); however, in the live cell format **94** showed a slight improvement in binding to CRBN after 2 h and even greater binding to CRBN after 5 h compared to **27**. This suggests that the increase in potency of **94** over **27** is due to an increase in cellular permeability and/or accumulation from inverting the hydroxy proline OH stereocenter. To further explore the functional impact of degradation mediated by these control compounds, we next evaluated the cell antiproliferative effect of **93**–**95**, using the same cell viability assay described above, in BET sensitive RKO WT and CRBN and/or VHL KO/dKO cell lines. We again treated cells with ranging concentrations of compound and measured cell viability normalized to a DMSO control (Figure 10).

Expectedly, double negative control, *neg*-*cis*-AB3067 (**95**) gave a similar antiproliferative effect in each WT, KO and dKO cell line with EC_50_s between 522–657 nM, comparable with the dKO plot of AB3067 (**27**, EC_50_ = 818 nM) (Figure 10 & Table S6).

Encouragingly, *neg*-AB3067 (**93**, inactive CRBN ligand) displays the same antiproliferative effect in both WT and CRBN KO cells with EC_50_s ∼ 106 nM, again, very comparable with the WT and CRBN KO plots of **27** (EC_50_ = 73 and 82 nM, respectively). This effect is fourfold weaker in VHL KO cells which shares the same antiproliferative effect as for VHL/CRBN dKO cells with EC_50_s ∼ 400 nM. Reassuringly, the antiproliferative profiles of **93** share the same trends as for heterobivalent PROTAC MZ1, which also has an enhanced antiproliferative effect in WT and CRBN KO cells (EC_50_s = 160 and 136 nM, respectively), compared to VHL KO and VHL/CRBN dKO cells (EC_50_s > 5 μM, Figure 10 & Table S6). Interestingly, **93** displayed a slightly greater antiproliferative effect than MZ1 in WT (EC_50_ = 107 vs 160 nM, respectively) and CRBN KO (EC_50_ = 105 vs 136 nM, respectively) cell lines, while also giving a marked >10-fold increased antiproliferative effect in both VHL KO and VHL/CRBN dKO cells compared to MZ1 (EC_50_ ∼ 400 vs > 5 μM, respectively), the latter likely due to a stronger BET inhibitory potential relative to MZ1.

Furthermore, *cis*-AB3067 (**94**, inactive VHL ligand) displays the same antiproliferative effect in both WT and VHL KO cells with EC_50_s of 48 nM, interestingly performing slightly better than **27** in both the WT and VHL KO cell lines (EC_50_ = 73 and 110 nM, respectively). This is likely due to the increases in BRD4 degradation potency displayed by **94** relative to **27** (Figure S5) from increases in cellular permeability (Figure S6). This effect is 15-fold weaker in CRBN KO cells which share a similar antiproliferative nature as for VHL/CRBN dKO cells with EC_50_ = 832 and 804 nM, respectively. Reassuringly, the antiproliferation profiles of **94** share the same trends as for heterobivalent PROTAC dBET6, which also gives enhanced antiproliferation in WT and VHL KO cells (EC_50_s = 256 and 291 nM, respectively), than in CRBN KO and VHL/CRBN dKO cells (EC_50_*s* > 10 μM, Figure 10). Interestingly, **94** gave a > fivefold larger antiproliferative effect than dBET6 in WT (EC_50_ = 48 vs 256 nM, respectively) and VHL KO (EC_50_ = 48 vs 291 nM, respectively) cell lines, while also giving a marked >12-fold increased antiproliferative effect in both CRBN KO and VHL/CRBN dKO cells compared to dBET6 (EC_50_ ∼ 820 nM vs > 10 μM, respectively), a trend similar to the comparison between **93** and MZ1. Curiously, while comprising of the same BET ligand JQ1, **27**, and **93**–**95**, show a much greater cell antiproliferation in VHL/CRBN dKO cells than MZ1 and dBET6, suggesting that **27**, and **93**–**95** have a stronger BET inhibitory potential (Figure 10 and Table S6).

We further assessed the effects of antiproliferation when dosing heterotrivalent BET PROTAC **27** (CRBN and VHL-recruiting) alone vs dosing two heterobivalent BET PROTACs, MZ1 (VHL-recruiting) and dBET6 (CRBN-recruiting), at the same time (Figure S7 and Table S7). Encouragingly, **27** was shown to be more cytoxic than the 1:1 mixture of dBET6 and MZ1, further exemplifying the advantages of having all three ligands in one molecule.

**Figure 11:**
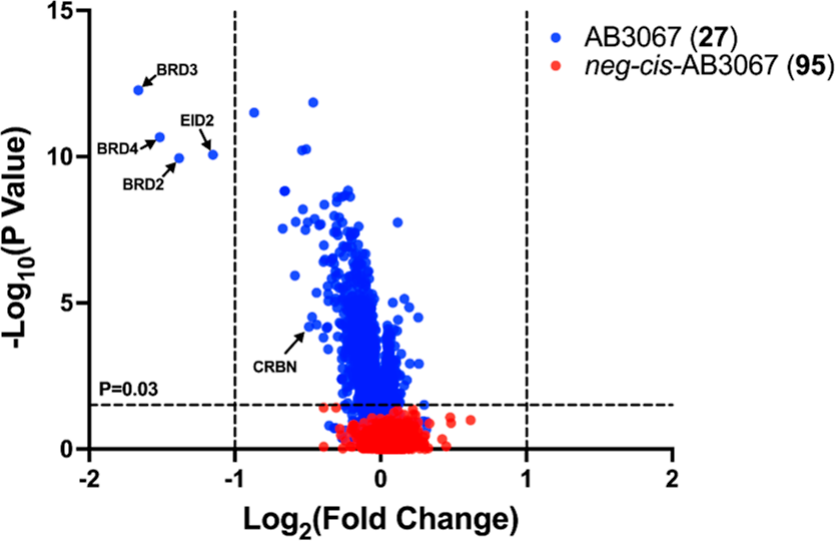
Proteomics of AB3067 (**27**) and *neg-cis*-AB3067 (**95**) treated HEK293 cells. Volcano plot showing impact on the proteome of HEK293 cells after 4 h following a 250 nM treatment of either **27** (blue) or **95** (red) relative to a vehicle control (DMSO). The data plotted is log2 of the normalized fold change in abundance against -log_10_ of the P value per protein identified from TMT (tandem mass tagging) mass spectrometry analysis produced from five independent repeats. A total of 7276 proteins were identified in this experiment. Dashed lines on the *x*-axis indicates boundary line for proteins to be considered differentially expressed at [Log_2_2 = 1]. Dashed line on the *y*-axis indicates boundary line for proteins to be considered statistically significant; any proteins with a -log_10_(P value) ≥ 1.5 to have a P value ≤ 0.03.

Finally, to evaluate both the on- and off-target impact of **27**, we performed an unbiased mass spectrometry proteomics experiment by treating HEK293 cells with **27**, using *cis-neg*-AB3067 (**95**) and DMSO as negative and vehicle controls, respectively. Of the 7276 proteins detected in this experiment, all three BET proteins, BRD2, BRD3 and BRD4, were significantly depleted upon **27** treatment, while no protein was significantly downregulated upon treatment with the inactive nondegrading control, **95** (Figure 11). Beyond the BET proteins, another protein that was significantly downregulated by **27** was EP300 interacting inhibitor protein of differentiation 2 (EID2). EID2 is a 28-kDa protein associated with inhibiting the acetyltransferase activity of p300.^42^ We speculate that EID2 depletion upon **27** treatment is an immediate response to the loss of BET regulation of the cellular acetylation state. Notably, CRBN was not significantly depleted at this treatment concentration (250 nM) and treatment time (4 h) of **27**, even though previous Western blots analysis of **27** showed observable degradation of CRBN at 6 h at concentrations between 100 and 1000 nM (Figure 4A). This is likely contributed by the well-known ratio compression phenomenon of TMT labeling proteomics. Nonetheless, the proteomics data, consistent with our substantive data on compound degradation profiling and selectivity, highlights and confirms the existence of a sweet spot of compound treatment concentration and time that allows achievement of a significant window between on-target BET protein degradation while minimizing undesired cross-E3 degradation of CRBN.

### 2.8 Development of Heterotrivalent BromoTag PROTAC AB3145 (**97**)

**Scheme 7:**
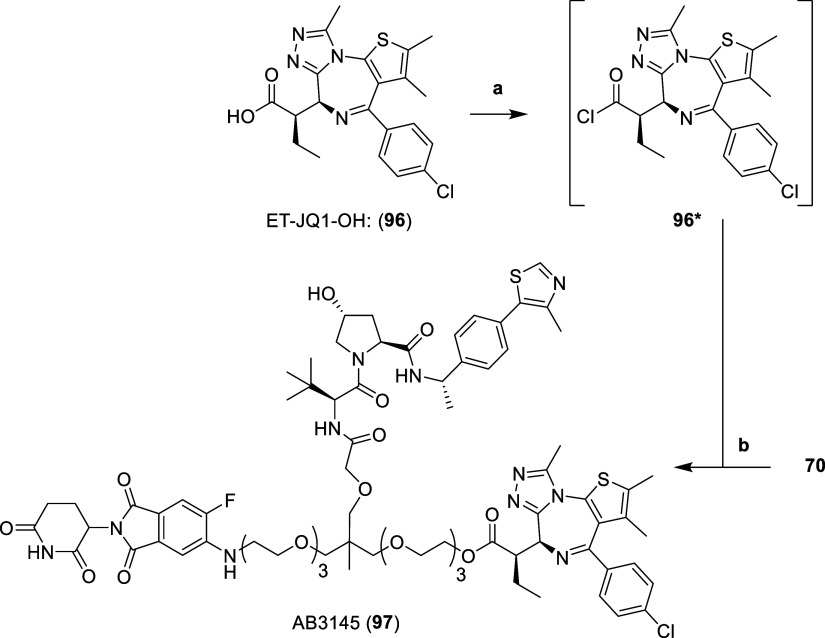
**Synthesis of Heterotrivalent BromoTag PROTAC AB3145 (97)** Reaction conditions: (a) SOCl_2_, DCM, r.t., 3 h; (b) (i) 4 N HCl in dioxane, MeOH, r.t., 3 h, (ii) 96*, DIPEA, DCM, r.t., 16 h.

To show general applicability of our heterotrivalent PROTAC strategy, we designed an AB3067-like compound for targeting BromoTag.^28,43^ BromoTag is our recently reported inducible degradation system that leverages an engineered Leu–Ala version of BRD4-BD2 as a universal tag for targeted protein degradation.^28^ We designed and synthesized compound AB3145 (**97**), which (analogous to VHL-based bifunctional degrader AGB1) bears an ethyl-“bump” in the BET ligand, allowing for exquisite selectivity toward the BromoTag while sparingly degrading endogenous BET proteins. To make the heterotrivalent BromoTag PROTAC, we followed a similar synthesis to **27** (c.f. Scheme 3), but now using the BromoTag selective ligand ET-JQ1-OH (**96**) instead of endogenous pan-BET ligand JQ1 (**22**) (Scheme 7).

First, the MOM protecting group of **70** was hydrolyzed with 4 N hydrochloric acid in dioxane and methanol. The subsequent primary alcohol was immediately conjugated to an intermediate acid chloride (96*), formed after treating ET-JQ1-OH (**96**, synthesized through literature procedures^43^) with thionyl chloride in DCM, to afford the ester of heterotrivalent BromoTag PROTAC AB3145 (**97**) (Scheme 7).

**Figure 12:**
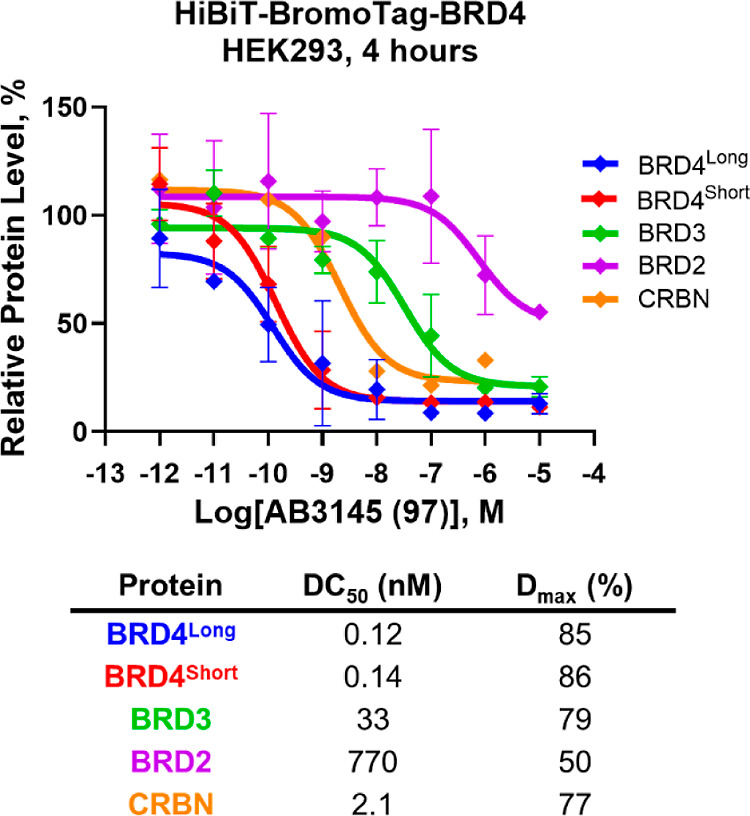
Western blot evaluation of heterotrivalent BromoTag PROTAC AB3145 (**97**) in homozygous CRISPR knock-in HiBiT-BromoTag-BRD4 HEK293 cell line. Plot of Western blot data for BET and CRBN protein levels after 10 μM to 1 pM treatments of **97** over 4 h in a homozygous endogenous HiBiT-BromoTag-BRD4 HEK293 cell line. Protein levels are normalized to tubulin and vehicle controls (DMSO) to derive DC_50_ values. Data is mean ± S.D.; *n* = 2 biological replicates (BRD4, BRD3 and BRD2) or *n* = 1 biological replicate (CRBN). Calculated DC_50_ and *D*_max_ values are tabulated below.

Western blot degradation assays in a homozygous CRISPR knock-in BromoTag-BRD4 HEK293 cell line evidence the highly potent degradation activity of **97** on the BromoTag-BRD4 protein (DC_50_: 120–140 pM; *D*_max_: 85–86%), maintaining a 250- and 6000-fold selectivity window over BRD3 (DC_50_: 33 nM; *D*_max_: 79%) and BRD2 (DC_50_: 770 nM; *D*_max_: 50%), respectively (Figure 12, S11 and Table S8).

Notably, **97** also displayed potent degradation of CRBN (DC_50_: 2.1 nM; *D*_max_: 77%) which is ∼35-fold more potent than **27** (DC_50_: 75 nM, Table 2). This is likely due to increases in the cellular permeability of **97** through increases in lipophilicity from the additional ethyl group. Later analogues would focus on trying to dial out this unwanted CRBN degradation. Crucially, **97** proved to be 10 to 100-fold more potent than the current BromoTag degrader AGB1 at degrading the BromoTag-BRD4 (Figure S11 and Table S8), evidencing the advantage of the heterotrivalent strategy in augmenting protein degradation fitness for proteins of interest.

## 3 Conclusion

In summary, we report novel heterotrivalent dual ligase recruiting PROTACs that potently and rapidly degrade the engaged target protein. Trivalent CRBN-VHL-BET PROTAC AB3067 (**27**) qualified as the most potent and fastest degrader, and most cytotoxic in BET sensitive cells. AB3067-induced BRD4 degradation was shown to be a result of ternary complexes with VHL and CRBN, and ubiquitination by each E3, suggesting that both E3 ligases are contributing to its activity. This is consistent with the evidence that loss of AB3067 cellular activity requires simultaneous loss of both recruited E3 ligases. We further exemplify a heterotetravalent PROTAC bearing a further copy of the BET ligand, and a heterotrivalent PROTAC with much improved degradation potency for BromoTag. Altogether, our work suggests that increasing valency to recruit two E3 ligases by the same PROTAC molecule can be an attractive strategy to augment the efficacy of targeted protein degradation. This approach could offer an opportunity to delay or overcome resistance to PROTAC degraders. Future work will be directed at exploring this important concept in cancer cells. Establishing further mechanistic features of multifunctional PROTACs, for example, illuminating the formation of a potential 1:1:1:1 quaternary complex will also be warranted. It is also envisaged that exploration of other chemistries, and tri- or multifunctional core scaffolds will accelerate rapid high-throughput assembly and direct-to-biology testing of larger libraries of multifunctional PROTACs and other proximity-inducing agents.

## 4 Experimental Section

### 4.1 Synthesis

Chemicals, commercially available, were purchased from Apollo Scientific, Sigma-Aldrich, Fluorochem, or Manchester Organics and used without any further purification. All reactions were carried out using anhydrous solvents. Reactions were monitored using either: an Agilent Technologies 1200 series analytical HPLC (High Performance Liquid Chromatography) connected to an Agilent Technologies 6130 quadrupole LC-MS containing an Agilent diode array detector and a Waters XBridge C18 column (50 mm × 2.1 mm, 3.5 μm particle size). Samples were eluted with a 3 min gradient of 5% to 95% MeCN/water containing 0.1% HCOOH at a flow rate of 0.7 mL/min; or a Shimadzu HPLC/MS 2020 with photodiode array detector and a Hypersil Gold column (1.9 μm 50 × 2.1 mm). Samples were eluted with a 3 min gradient of 5% to 95% MeCN/water containing 0.1% HCOOH at a flow rate of 0.8 mL/min. Intermediates were purified by flash column chromatography using a Teledyne Isco Combiflash Rf or Rf200i, with Normal Phase RediSep Rf Disposable Columns or with Reverse Phase RediSep Rf Gold C18 Reusable Columns. Final compounds were purified by HPLC using a Gilson Preparative HPLC System equipped with a Waters X-Bridge C18 column (100 mm × 19 mm; 5 μm particle size) using a gradient from 5% to 95% of MeCN in water containing 0.1% HCOOH or ammonia over 10 min at a flow rate of 25 mL/min unless stated otherwise. Compound characterization using NMR was performed either on a Bruker 500 Ultrashield or Bruker Ascend 400 spectrometers. The proton (^1^H) and carbon (^13^C) reference solvents used were as follows: *d*_1_-CDCl_3_ (δH = 7.26 ppm/δC = 77.15 ppm), *d*_4_-CD_3_OD (δH = 3.31 ppm/δC = 49.00 ppm), *d*_6_-(CD_3_)_2_SO (δH = 2.50 ppm/δC = 39.52 ppm). Signal patterns are described as singlet (s), doublet (d), triplet (t), quartet (q), quintet (quint.), multiplet (m), broad (br), or a combination of the listed splitting patterns. Coupling constants (*J*) are measured in Hertz (Hz). NMR spectra for all compounds were processed using Bruker TopSpin 4.1.1. High resolution mass spectrometry (HRMS) data was performed on a Bruker MicrOTOF II focus ESI Mass Spectrometer connected in parallel to Dionex Ultimate 3000 RSLC system with diode array detector and a Waters XBridge C18 column (50 mm × 2.1, 3.5 μm particle size). Samples were eluted with a 6 min gradient of 5% to 95% MeCN: water containing 0.1% HCOOH at a flow rate of 0.6 mL/min. All compounds are >95% pure by HPLC.

### 4.2 General Procedure A

Alcohol/diol (1.0 equiv) was dissolved in DMF (2.4 mL/mmol) under N_2_ and cooled to 0 °C. 60% NaH in paraffin oil (1.2–4.0 equiv) was carefully added and the flask was left to stir at 0 °C for 30 min. A solution of mesylate (1.0–3.0 equiv) in DMF (0.5–0.8 mL/mmol) was then added to the flask dropwise and the reaction was left to stir at 60 °C for 16 h. The mixture was then filtered through PTFE filters or Celite and concentrated in vacuo and purified.

### 4.3 General Procedure B

To a solution of alkene (1.0 equiv) in dioxane (18 mL/mmol) and water (4.6 mL/mmol) was added NaIO_4_ (4.0 equiv), 2,6-lutidine (2.0 equiv) and 4% OsO_4_ in water (0.01 equiv). The reaction was left to stir at r.t. for 16 h. The resulting white suspension was quenched with saturated Na_2_SO_3_ solution, extracted with DCM (4 × 10 mL), dried with MgSO_4,_ and concentrated in vacuo. The residue is then purified by flash column chromatography using a linear gradient of 0% to 10% MeOH in DCM to yield aldehydes as colorless oils.

### 4.4 General Procedure C

To a solution of aldehyde (1.0 equiv) in *t*-BuOH (18 mL/mmol) and water (5.9 mL/mmol) was added NaH_2_PO_4_ (1.0 equiv), NaClO_2_ (3.95 equiv) followed by 2 M 2-methyl-2-butene in THF (5.0 equiv), and the reaction left to stir at r.t. for 16 h. The reaction was diluted with 2 M NaOH (aq) solution (1 mL) and then carefully neutralized 2 M HCl (1 mL). The mixture was extracted with DCM (4 × 10 mL), dried with MgSO_4_ and concentrated in vacuo to yield carboxylic acids as colorless oils without the need for further purification.

### 4.5 General Procedure D

To a solution of carboxylic acid (1.0 equiv) in DMF (7.7 mL/mmol) was added DIPEA (4.0 equiv). HATU (1.1 equiv) was then added, and the reaction was stirred at r.t. for 5 min. VH032-amine (**14** synthesized according to literature^16,21^), Me-VH032-amine (**58**, synthesized according to literature^29^) or *cis*-Me-VH032-amine (**88**, synthesized according to literature^29^) (1.1 equiv) was then added and the reaction left to stir at r.t. for 2 h. The reaction is then concentrated under vacuum and purified by reverse phase flash column chromatography (C18 gold column) using a linear gradient from 0% to 100% MeCN in 0.1% HCOOH in water to afford amides as colorless oils.

### 4.6 General Procedure E

(Step 1) MOM/MEM protected compound (1.0 equiv) was dissolved in MeOH (26 mL/mmol). 4 N HCl in dioxane (13 mL/mmol) was then added and the reaction was left to stir for at r.t. for 2 h. The reaction was then concentrated under vacuum to quantitatively yield alcohols without the need for purification. (Step 2) In a separate flask was dissolved (+)-JQ1 carboxylic acid (**22**) or ET-JQ1-OH (**96**, synthesized through literature procedures^43^) (1.5–3.0 equiv) in anhydrous DCM (9.4 mL/mmol) under an atmosphere of N_2_. Neat SOCl_2_ (22.5–45 equiv) was then added and left to stir at r.t. Conversion to the acid chloride was monitored by LC–MS by dissolving a sample in MeOH and observing the mass of the methyl ester of JQ1 (calc. for C_20_H_20_ClN_4_O_2_S [M + H]^+^ 415.9) or ET-JQ1-OH (calc. for C_22_H_24_ClN_4_O_2_S [M + H]^+^ 443.1). Complete conversion was observed after 1.5 h and the mixture was concentrated in vacuo. (Step 3) The acid chloride intermediate (1.5–3 equiv) was redissolved in anhydrous DCM (9.6 mL/mmol) and added to a N_2_ purged flask containing alcohol (1.0 eq., from Step 1). Anhydrous DIPEA was added (3.0–5.0 eq., or until pH 9.0) and left to stir at r.t. for 16 h. The mixtures were then concentrated in vacuo and the residues were purified by HPLC.

### 4.7 General Procedure F

To a N_2_ flushed flask containing a solution of triethylene (**33**) or diethylene glycol (**34**) (5.25 equiv) in DCM (0.5 mL/mmol), was added DIPEA (1.1 equiv). MEMCl or MOMBr (1.0 equiv) were then added dropwise, and the reaction was left to stir at r.t. for 16 h. The mixture was then diluted with DCM (20 mL) and water (20 mL), and the organic layer separated. The aqueous phase was extracted with DCM (3 × 20 mL), and the combined organic layers were dried with MgSO_4_ and concentrated in vacuo. The residue was then purified by flash column chromatography to yield mono-MEM/MOM protected alcohols as colorless oils.

### 4.8 General Procedure G

To a solution of alcohol (1.0 equiv) dissolved in anhydrous DCM (4.9 mL/mmol) was added DIPEA (3.0 equiv) before flushing the flask with N_2_ and cooling to 0 °C. MsCl (3.0 equiv) was then added dropwise, and the reaction was left to stir at 0 °C for 20 min before warming to r.t. and stirring for 2 h. The reaction mixture was concentrated in vacuo. The residue was then purified by flash column chromatography using a linear gradient from 0% to 100% EtOAc in heptane to yield mesylates as orange/red oils.

### 4.9 General Procedure H

Azide (1.0 equiv) was dissolved in MeOH (58 mL/mmol). A catalytic amount of 10 wt % Pd/C was added, and the reaction was stirred under an atmosphere of H_2_ for 16 h. The reaction mixture was then filtered through PTFE syringe filters and evaporated to dryness to obtain the desired amine in quantitative yields. The resulting amine (1.0 equiv) was added to a solution of thalidomide derivatives **19**, **65** or **87** (1.0 equiv) and DIPEA (4.0 equiv) in DMSO (24 mL/mmol) and the reaction was left to stir in a sealed vial at 90 °C for 4 h. The reaction was then purified by HPLC using a linear gradient of 5% to 95% MeCN in 0.1% HCOOH in water over 10 min gradient unless otherwise stated.

#### 4.9.1 (2*S*,4*R*)-1-((20*S*)-20-(*tert*-Butyl)-1-((*S*)-4-(4-chlorophenyl)-2,3,9-trimethyl-6*H*-thieno[3,2-*f*][1,2,4]triazolo[4,3-*a*][1,4]diazepin-6-yl)-14-((2-(2-(2-((2-(2,6-dioxopiperidin-3-yl)-1,3-dioxoisoindolin-4-yl)amino)ethoxy)ethoxy)ethoxy)methyl)-14-methyl-2,18-dioxo-6,9,12,16-tetraoxa-3,19-diazahenicosan-21-oyl)-4-hydroxy-*N*-(4-(4-methylthiazol-5-yl)benzyl)pyrrolidine-2-carboxamide (MN666) (**1**)

Azide **20** (12 mg, 10.6 μmol) was dissolved in MeOH (600 μL). A catalytic amount of 10 wt % Pd/C (3 mg) was added, and the reaction was stirred under an atmosphere of hydrogen for 24 h. The reaction mixture was then filtered through a PTFE syringe filter and evaporated to dryness to leave crude amine intermediate. The crude amine (8 mg, 7.2 μmol) was dissolved in DMF (100 μL) and added to a solution of (+)-JQ1-acid (**22**) (3 mg, 7.5 μmol), HATU (3 mg, 7.9 μmol) and DIPEA (5 μL, 28.7 μL) in DMF (400 μL) and stirred at r.t. for 2 h. After completion, the reaction was directly purified by HPLC using a linear gradient over 10 min from 25% to 95% MeCN in 0.1% HCOOH in water. Yield: 5.4 mg (34%); Contains a mixture of four diastereomers; ^1^H NMR (500 MHz, CDCl_3_): δ = 9.50–9.19 (m, 1H), 8.73–8.71 (m, 1H), 7.78–7.71 (m, 1H), 7.49–7.31 (m, 10H), 7.23–7.17 (m, 1H), 7.09–7.06 (m, 1H), 6.92–6.88 (m, 1H), 6.49 (s, 1H), 4.89–4.45 (m, 6H), 4.35–4.24 (m, 1H), 4.09–3.93 (m, 3H), 3.73–3.21 (m, 32H), 2.83–2.62 (m, 6H), 2.52–2.50 (m, 3H), 2.44–2.37 (m, 4H), 2.19–2.02 (m, 2H), 1.64 (s, 3H), 1.39–1.29 (m, 1H), 1.21–1.17 (m, 1H), 0.98–0.95 (m, 9H), 0.90–0.88 ppm (m, 3H); HRMS *m*/*z* calc. for C_73_H_92_ClN_12_O_16_S_2_ [M + H]^1 +^ 1491.5879, found: 1491.5907.

#### 4.9.2 (2*S*,4*R*)-1-((17*S*)-17-(*tert*-Butyl)-1-((*S*)-4-(4-chlorophenyl)-2,3,9-trimethyl-6*H*-thieno[3,2-*f*][1,2,4]triazolo[4,3-*a*][1,4]diazepin-6-yl)-11-((2-(2-((2-(2,6-dioxopiperidin-3-yl)-1,3-dioxoisoindolin-4-yl)amino)ethoxy)ethoxy)methyl)-11-methyl-2,15-dioxo-6,9,13-trioxa-3,16-diazaoctadecan-18-oyl)-4-hydroxy-*N*-(4-(4-methylthiazol-5-yl)benzyl)pyrrolidine-2-carboxamide (MN675) (**2**)

Azide **21** (13 mg, 12.4 μmol) was dissolved in MeOH (600 μL). A catalytic amount of 10 wt % Pd/C (3 mg) was added, and the reaction was stirred under an atmosphere of hydrogen for 24 h. The reaction mixture was then filtered through a PTFE syringe filter and evaporated to dryness to leave crude amine intermediate. The crude amine (13 mg, 12.4 μmol) was dissolved in DMF (100 μL) and added to a solution of (+)-JQ1-acid (**22**) (5 mg, 12.4 μmol), HATU (5 mg, 13.1 μmol) and DIPEA (10 μL, 57.4 μL) in DMF (400 μL) and stirred at r.t. for 2 h. After completion, the reaction was directly purified by HPLC using a linear gradient over 10 min from 25% to 95% MeCN in 0.1% HCOOH in water. Yield: 10 mg (59%); Contains a mixture of four diastereomers; ^1^H NMR (500 MHz, CDCl_3_): δ = 9.89–9.55 (m, 1H), 8.72 (s, 1H), 7.93–7.79 (m, 1H), 7.49–7.31 (m, 10H), 7.28–7.23 (m, 1H), 7.07–7.05 (m, 1H), 6.92–6.88 (m, 1H), 6.50 (s, 1H), 4.90–4.83 (m, 1H), 4.78–4.47 (m, 5H), 4.32–4.27 (m, 1H), 4.15–3.95 (m, 3H), 3.71–3.22 (m, 24H), 2.78–2.61 (m, 6H), 2.52–2.50 (m, 3H), 2.40–2.31 (m, 4H), 2.17–1.98 (m, 2H), 1.64 (s, 3H), 1.38–1.29 (m, 1H), 1.21–1.16 (m, 1H), 0.98–0.94 (m, 9H), 0.90–0.87 ppm (m, 3H); HRMS *m*/*z* calc. for C_69_H_84_ClN_12_O_14_S_2_ [M + H]^+^ 1403.5354, found: 1403.5402.

#### 4.9.3 5-((allyloxy)methyl)-2,2,5-trimethyl-1,3-dioxane (**4**)

(2,2,5-trimethyl-1,3-dioxan-5-yl)methanol (**3**) (2.0 g, 12.5 mmol) was dissolved in toluene (12.5 mL). KOH (2.1 g, 37.5 mmol) was dissolved in H_2_O (2.1 mL) and added to the flask, followed by TBAB (403 mg, 1.25 mmol) and allyl bromide (4.53 g, 37.5 mmol). The reaction was stirred vigorously at r.t. for 16 h. DCM (20 mL) was added, and the aqueous layer was extracted with DCM (3 × 20 mL), dried with MgSO_4_ and concentrated in vacuo. The residue was purified by flash column chromatography (40 g silica column) using a linear gradient from 0% to 50% EtOAc in heptane to afford **121** (**4**) as a colorless oil. Yield: 1.4 g (54%); Analytics matched those reported in literature (ref (11)); ^1^H NMR (400 MHz, CDCl_3_): δ = 5.95–5.85 (m, 1H), 5.27 (qd, *J* = 1.7, 17.3 Hz, 1H), 5.16 (qd, *J* = 1.5, 10.4 Hz, 1H), 4.00 (td, *J* = 1.5, 5.4 Hz, 2H), 3.72 (d, *J* = 12.0 Hz, 2H), 3.55 (d, *J* = 11.9 Hz, 2H), 3.43 (s, 2H), 1.43 (s, 3H), 1.40 (s, 3H), 0.89 ppm (s, 3H); ^13^C NMR (101 MHz, CDCl_3_): δ = 135.2, 116.5, 98.0, 73.3, 72.5, 66.8, 34.5, 26.5, 21.4, 18.5.

#### 4.9.4 2-((allyloxy)methyl)-2-methylpropane-1,3-diol (**5**)

5-((allyloxy)methyl)-2,2,5-trimethyl-1,3-dioxane (**4**) (1.4 g, 6.99 mmol) was dissolved in MeOH (10 mL) and H_2_O (6 mL). TFA (600 μL) was then added, and the reaction was left to stir at r.t. for 3 h. The mixture was then evaporated to dryness and the residue was purified by flash column chromatography (24 g silica column) using a linear gradient from 0% to 20% MeOH in DCM to afford **5** as a colorless oil. Yield: 874 mg (78%); Analytics matched those reported in literature (ref (11)); ^1^H NMR (400 MHz, CDCl_3_): δ = 5.94–5.84 (m, 1H), 5.26 (qd, *J* = 1.6, 17.3 Hz, 1H), 5.20 (qd, *J* = 1.4, 10.4 Hz, 1H), 3.99 (td, *J* = 1.4, 5.5 Hz, 2H), 3.72 (d, *J* = 11.0 Hz, 2H), 3.61 (d, *J* = 10.9 Hz, 2H), 3.45 (s, 2H), 2.39 (br s, 2H), 0.84 ppm (s, 3H).

#### 4.9.5 11-((allyloxy)methyl)-1,21-diazido-11-methyl-3,6,9,13,16,19-hexaoxahenicosane (**8**)

Follow General Procedure A, using 1.0 eq. of diol **5**, 4 eq. of NaH and 3 eq. of 2-(2-(2-azidoethoxy)ethoxy)ethylmethanesulfonate (**6**). Purified by reverse phase flash column chromatography (50 g C18 gold column) using a linear gradient over 11 min from 0% to 100% MeCN in 0.1% HCOOH in water. Yield: 179 mg (60%); Analytics matched those reported in literature (ref (11)); ^1^H NMR (500 MHz, CDCl_3_) δ = 5.96–5.84 (m, 1H), 5.26 (dd, *J* = 1.7, 17.2 Hz, 1H), 5.15 (dd, *J* = 1.5, 10.4 Hz, 1H), 4.01–3.93 (m, 2H), 3.76–3.61 (m, 16H), 3.62–3.55 (m, 4H), 3.44–3.37 (m, 4H), 3.37–3.33 (m, 4H), 3.33–3.29 (m, 2H), 0.96 (s, 3H); ^13^C NMR (126 MHz, CDCl_3_) δ = 135.3, 116.0, 74.0, 73.0, 72.3, 71.1, 70.8, 70.7, 70.5, 70.0, 50.7, 41.0, 17.4.

#### 4.9.6 8-((allyloxy)methyl)-1,15-diazido-8-methyl-3,6,10,13-tetraoxapentadecane (**9**)

Follow General Procedure A, using 1.0 eq. of diol **5**, 4 eq. of NaH and 3 eq. of 2-(2-azidoethoxy)ethylmethanesulfonate (**7**). Purified by reverse phase flash column chromatography (50 g C18 gold column) using a linear gradient over 11 min from 0% to 100% MeCN in 0.1% HCOOH in water. Yield: 507 mg (60%); ^1^H NMR (500 MHz, CDCl_3_): δ = 5.93–5.84 (m, 1H), 5.25 (qd, *J* = 1.7, 17.2 Hz, 1H), 5.14 (qd, *J* = 1.5, 10.4 Hz, 1H), 3.94 (td, *J* = 1.5, 5.3 Hz, 2H), 3.69–3.67 (m, 4H), 3.65–3.61 (m, 4H), 3.60–3.56 (m, 4H), 3.37 (t, *J* = 5.1 Hz, 4H), 3.35 (s, 4H), 3.31 (s, 2H), 0.96 ppm (s, 3H); ^13^C NMR (126 MHz, CDCl_3_): δ = 135.4, 116.2, 74.2, 73.2, 72.4, 71.3, 70.7, 70.2, 51.0, 41.1, 17.6.

#### 4.9.7 1-Azido-11-((2-(2-(2-azidoethoxy)ethoxy)ethoxy)methyl)-11-methyl-3,6,9,13-tetraoxapentadecan-15-al (**10**)

Follow General Procedure B, using alkene **8**. Yield: 16 mg (64%); Analytics matched those reported in literature (ref (11)); ^1^H NMR (500 MHz, CDCl_3_) δ = 9.73 (s, 1H), 4.02 (s, 2H), 3.74–3.52 (m, 20H), 3.46–3.26 (m, 10H), 0.98 (s, 3H); ^13^C NMR (126 MHz, CDCl_3_) δ = 202.2, 77.0, 74.7, 73.9, 71.2, 70.9, 70.8, 70.7, 70.2, 50.9, 41.3, 17.5.

#### 4.9.8 2-(3-(2-(2-azidoethoxy)ethoxy)-2-((2-(2-azidoethoxy)ethoxy)methyl)-2-methylpropoxy)acetaldehyde (**11**)

Follow General Procedure B, using alkene **9**. Yield: 83%; ^1^H NMR (400 MHz, CDCl_3_): δ = 9.73 (t, *J* = 1.0 Hz, 1H), 4.02 (d, *J* = 1.0 Hz, 2H), 3.70–3.55 (m, 12H), 3.44 (s, 2H), 3.40–3.33 (m, 8H), 0.99 ppm (s, 3H); ^13^C NMR (101 MHz, CDCl_3_): δ = 202.1, 77.0, 74.7, 73.9, 71.2, 70.7, 70.2, 50.9, 41.3, 17.4.

#### 4.9.9 1-Azido-11-((2-(2-(2-azidoethoxy)ethoxy)ethoxy)methyl)-11-methyl-3,6,9,13-tetraoxapentadecan-15-oic Acid (**12**)

Follow General Procedure C, using aldehyde **10**. Yield: 150 mg (97%); Analytics matched those reported in literature (ref (11)); 1H NMR (500 MHz, CDCl_3_) δ = 4.05 (s, 2H), 3.70–3.58 (m, 20H), 3.45–3.33 (m, 10H), 0.95 (s, 3H); ^13^C NMR (126 MHz, CDCl_3_) δ = 172.1, 75.4, 74.7, 71.3, 70.9, 70.7, 70.4, 70.2, 68.8, 50.8, 40.8, 18.0.

#### 4.9.10 2-(3-(2-(2-azidoethoxy)ethoxy)-2-((2-(2-azidoethoxy)ethoxy)methyl)-2-methylpropoxy)acetic Acid (**13**)

Follow General Procedure C, using aldehyde **11**. Yield: 422 mg (95%); ^1^H NMR (500 MHz, CDCl_3_): δ = 4.06 (s, 2H), 3.69–3.60 (m, 12H), 3.48–3.44 (m, 4H), 3.40 (d, *J* = 9.3 Hz, 2H), 3.39–3.35 (m, 4H), 0.97 ppm (s, 3H); ^13^C NMR (126 MHz, CDCl_3_): δ = 171.9, 75.7, 75.0, 71.4, 70.5, 70.2, 68.7, 50.9, 40.8, 18.0.

#### 4.9.11 (2*S*,4*R*)-1-((*S*)-1-azido-11-((2-(2-(2-azidoethoxy)ethoxy)ethoxy)methyl)-17-(*tert*-Butyl)-11-methyl-15-oxo-3,6,9,13-tetraoxa-16-azaoctadecan-18-oyl)-4-hydroxy-*N*-(4-(4-methylthiazol-5-yl)benzyl)pyrrolidine-2-carboxamide (**15**)

Follow General Procedure D, using carboxylic acid **13** and VH032-amine (**14**, synthesized through literature procedures^21,22^). Yield: 55 mg (50%); Analytics matched those reported in literature (ref (11)); 1H NMR (500 MHz, CDCl_3_) δ (ppm) = 8.68 (s, 1H), 7.39–7.30 (m, 5H), 7.10 (d, *J* = 8.5 Hz, 1H), 4.73 (t, *J* = 7.8 Hz, 1H), 4.59–4.51 (m, 2H), 4.48 (d, *J* = 8.7 Hz, 1H), 4.35 (dd, *J* = 5.5, 15.0 Hz, 1H), 4.09 (d, *J* = 12.0 Hz, 1H), 3.94 (dd, *J* = 15.4, 17.7 Hz, 2H), 3.71–3.53 (m, 21H), 3.46–3.30 (m, 10H), 2.60–2.49 (m, 1H), 2.51 (s, 3H), 2.16–2.08 (m, 1H), 0.96 (s, 3H), 0.95 (s, 9H). 13C NMR (126 MHz, CDCl_3_) δ (ppm) = 171.5, 170.8, 170.7, 150.5, 148.6, 138.3, 131.8, 131.1, 129.7, 128.3, 74.8, 74.2, 74.1, 71.2, 70.9, 70.8, 70.7, 70.6, 70.3, 70.2, 58.5, 57.2, 56.7, 50.8, 43.4, 41.1, 35.9, 35.0, 26.5, 17.7, 16.2. LC–MS *m*/*z* calc. for C_41_H_65_N_10_O_11_S [M + H]^+^ 905.5, found 905.3.

#### 4.9.12 (2*S*,4*R*)-1-((S)-15-azido-8-((2-(2-azidoethoxy)ethoxy)methyl)-2-(*tert*-Butyl)-8-methyl-4-oxo-6,10,13-trioxa-3-azapentadecanoyl)-4-hydroxy-*N*-(4-(4-methylthiazol-5-yl)benzyl)pyrrolidine-2-carboxamide (**16**)

Follow General Procedure D, using carboxylic acid **13** and VH032-amine (**14**, synthesized through literature procedures^21,22^). Yield: 312 mg (77%); ^1^H NMR (500 MHz, CDCl_3_): δ = 8.68 (s, 1H), 7.39–7.30 (m, 5H), 7.12 (d, *J* = 8.3 Hz, 1H), 4.75 (t, *J* = 7.9 Hz, 1H), 4.60–4.51 (m, 2H), 4.45 (d, *J* = 8.5 Hz, 1H), 4.34 (dd, *J* = 5.4, 15.0 Hz, 1H), 4.14 (d, *J* = 11.1 Hz, 1H), 3.93 (s, 2H), 3.75–3.28 (m, 23H), 2.75 (s, 1H), 2.64–2.57 (m, 1H), 2.52 (s, 3H), 2.12 (dd, *J* = 7.9, 13.7 Hz, 1H), 0.99 (s, 3H), 0.95 ppm (s, 9H); LCMS calc. for C_37_H_57_N_10_O_9_S [M + H]^+^ is 817.4, found 817.9.

#### 4.9.13 (2*S*,4*R*)-1-((17*S*)-1-amino-11-((2-(2-(2-azidoethoxy)ethoxy)ethoxy)methyl)-17-(*tert*-Butyl)-11-methyl-15-oxo-3,6,9,13-tetraoxa-16-azaoctadecan-18-oyl)-4-hydroxy-*N*-(4-(4-methylthiazol-5-yl)benzyl)pyrrolidine-2-carboxamide (**17**)

Diazide **15** (148 mg, 0.163 mmol) was dissolved in a 4:1:5 ratio of EtOAc (4.5 mL), THF (1.1 mL) and 1 M HCl (aq) solution (5.6 mL). PPh_3_ (43 mg, 0.163 mmol) was then dissolved in EtOAc (4.2 mL) and added dropwise over 3 h (0.5 mL/h) to the flask containing the diazide solution. The reaction was then left to stir vigorously at r.t. for 16 h. The reaction was then diluted with 2 M HCl (aq) solution (5 mL), and the aqueous layer was separated and concentrated in vacuo. The residue was then purified by HPLC using a linear gradient over 10 min from 5% to 95% MeCN in 0.1% NH_3_ in water to afford mono amine **17**. Yield: 51 mg (36%); Contains a mixture of two diastereomers; Analytics matched those reported in literature (ref (11)); ^1^H NMR (500 MHz, CDCl_3_) δ (ppm) = 8.67 (s, 1H), 8.52 (br s, 1H), 7.38–7.33 (m, 4H), 7.17 (m, 1H), 4.67 (m, 1H), 4.55–4.47 (m, 3H), 4.38 (m, 1H), 4.07–3.94 (m, 4H), 3.70–3.21 (m, 31H), 2.98 (m, 2H), 2.51 (s, 3H), 2.31 (s, 1H), 2.23 (s, 1H), 1.02–0.93 (m, 12H); ^13^C NMR (126 MHz, CDCl_3_) δ (ppm) = 171.4, 171.03, 170.98, 170.72, 170.65, 169.4, 150.2, 148.4, 138.51, 138.47, 131.6, 130.67, 130.66, 129.36, 129.35, 127.97, 74.2, 74.1, 74.0, 73.9, 73.1, 71.1, 70.87, 70.84, 70.80, 71.72, 70.70, 70.62, 70.45, 70.42, 70.38, 70.34, 70.3, 70.2, 70.2, 70.0, 69.9, 68.6, 68.5, 59.0, 57.3, 57.0, 50.6, 43.0, 42.99, 41.03, 41.01, 39.8, 37.07, 36.98, 35.17, 35.10, 26.4, 17.5, 16.0; LC–MS calc. for C_41_H_67_N_8_O_11_S [M + H]^+^ 879.5, found 879.5.

#### 4.9.14 (2*S*,4*R*)-1-((2*S*)-15-amino-8-((2-(2-azidoethoxy)ethoxy)methyl)-2-(*tert*-Butyl)-8-methyl-4-oxo-6,10,13-trioxa-3-azapentadecanoyl)-4-hydroxy-*N*-(4-(4-methylthiazol-5-yl)benzyl)pyrrolidine-2-carboxamide (**18**)

Diazide **16** (256 mg, 0.313 mmol) was dissolved in a 1:1 ratio of EtOAc (2 mL) and 2 M HCl (aq) solution (2 mL). PPh_3_ (82 mg, 0.313 mmol) was then dissolved in EtOAc (2 mL) and added dropwise over 3 h (0.5 mL/h) to the flask containing the diazide solution. The reaction was then left to stir at r.t. for 16 h. The reaction was then diluted with 2 M HCl (aq) solution (3 mL), and the aqueous layer was separated, neutralized with 7 N NH_3_ in MeOH and then concentrated in vacuo. The residue was then purified by HPLC using a linear gradient over 10 min from 5% to 95% MeCN in 0.1% HCOOH in water to afford amine **18**. Yield: 120 mg (48%); Contains a mixture of two diastereomers; ^1^H NMR (500 MHz, CDCl_3_): δ = 8.65 (s, 1H), 8.44 (s, 1H), 8.33–8.26 (m, 1H), 7.36 (d, *J* = 8.1 Hz, 4H), 7.32 (d, *J* = 8.3 Hz, 4H), 7.20–7.14 (m, 1H), 4.62 (t, *J* = 8.7 Hz, 1H), 4.57–4.44 (m, 3H), 4.32 (dd, *J* = 5.3, 14.7 Hz, 1H), 4.04–3.99 (m, 3H), 3.69–3.22 (m, 22H), 3.01–2.86 (m, 2H), 2.50 (s, 3H), 2.24–2.12 (m, 2H), 1.01 (s, 9H), 0.96–0.92 ppm (m, 3H); ^13^C NMR (126 MHz, CDCl_3_): δ = 171.8, 171.02, 171.00, 170.96, 170.9, 169.0, 150.3, 148.5, 139.01, 138.99, 131.8, 130.7, 129.4, 128.1, 74.7, 74.6, 74.5, 74.3, 73.5, 73.4, 71.31, 71.26, 71.20, 71.16, 70.82, 70.77, 70.7, 70.5, 70.2, 70.1, 67.3, 59.3, 57.6, 57.24, 57.21, 50.9, 43.0, 41.2, 39.6, 37.6, 35.4, 26.5, 17.81, 17.78, 16.2; LC–MS calc. for C_37_H_59_N_8_O_9_S [M + H]^+^ 791.4, found 879.5.

#### 4.9.15 (2*S*,4*R*)-1-((17*S*)-1-azido-17-(*tert*-Butyl)-11-((2-(2-(2-((2-(2,6-dioxopiperidin-3-yl)-1,3-dioxoisoindolin-4-yl)amino)ethoxy)ethoxy)ethoxy)methyl)-11-methyl-15-oxo-3,6,9,13-tetraoxa-16-azaoctadecan-18-oyl)-4-hydroxy-*N*-(4-(4-methylthiazol-5-yl)benzyl)pyrrolidine-2-carboxamide (**20**)

To a solution of amine **17** (17 mg, 19.3 μmol) and DIPEA (20 μL, 116 μmol) dissolved in NMP (300 μL) was added 2-(2,6-dioxopiperidin-3-yl)-4-fluoroisoindoline-1,3-dione (**19**) (5.3 mg, 19.3 μmol). The reaction was left to stir in a sealed vial at 100 °C for 4 h. The reaction was then purified by HPLC using a linear gradient of 5% to 95% MeCN in 0.1% HCOOH in water over 10 min gradient. Yield: 12 mg (54%); Contains a mixture of four diastereomers; ^1^H NMR (400 MHz, CDCl_3_): δ = 9.36–9.13 (m, 1H), 8.68 (s, 1H), 7.50–7.40 (m, 2H), 7.38–7.33 (m, 4H), 7.19–7.13 (m, 1H), 7.09 (d, *J* = 6.7 Hz, 1H), 6.92–6.88 (m, 1H), 6.53–6.48 (m, 1H), 4.91–4.81 (m, 1H), 4.73–4.68 (m, 1H), 4.63–4.46 (m, 3H), 4.36–4.26 (m, 1H), 4.11 (d, *J* = 11.5 Hz, 1H), 3.98–3.88 (m, 2H), 3.73–3.25 (m, 32H), 2.87–2.61 (m, 3H), 2.56–2.48 (m, 4H), 2.15–2.04 (m, 2H), 0.96–0.93 ppm (m, 12H); ^13^C NMR (101 MHz, CDCl_3_): δ = 171.79, 171.76, 171.7, 171.5, 171.4, 171.0, 170.90, 170.85, 170.83, 170.78, 169.43, 169.38, 168.95, 168.90, 168.8, 167.8, 150.4, 148.7, 148.6, 147.01, 146.98, 138.5, 138.4, 136.1, 132.7, 131.8, 131.0, 129.6, 128.35, 128.29, 116.91, 116.87, 111.8, 111.7, 110.6, 74.5, 74.11, 74.06, 73.9, 73.8, 71.31, 71.25, 71.2, 71.01, 70.97, 70.9, 70.83, 70.78, 70.7, 70.6, 70.39, 70.37, 70.2, 69.6, 69.5, 58.75, 58.66, 57.2, 57.1, 56.8, 50.9, 49.1, 49.0, 43.4, 42.6, 42.6, 41.1, 36.2, 36.1, 35.1, 35.0, 31.6, 26.5, 22.9, 17.6, 16.2 LC–MS calc. for C_54_H_76_N_10_O_15_S [M+2H]^2+^ is 568.3, found 568.4.

#### 4.9.16 (2*S*,4*R*)-1-((2*S*)-15-azido-2-(*tert*-Butyl)-8-((2-(2-((2-(2,6-dioxopiperidin-3-yl)-1,3-dioxoisoindolin-4-yl)amino)ethoxy)ethoxy)methyl)-8-methyl-4-oxo-6,10,13-trioxa-3-azapentadecanoyl)-4-hydroxy-*N*-(4-(4-methylthiazol-5-yl)benzyl)pyrrolidine-2-carboxamide (**21**)

To a solution of amine **18** (36 mg, 45.5 μmol) and DIPEA (50 μL, 287 μmol) dissolved in NMP (1 mL) was added 2-(2,6-dioxopiperidin-3-yl)-4-fluoroisoindoline-1,3-dione (**19**) (15 mg, 54.3 μmol). The reaction was left to stir in a sealed vial at 120 °C for 4 h. The reaction was then purified by HPLC using a linear gradient of 40% to 95% MeCN in 0.1% HCOOH in water over 8 min gradient. Yield: 13 mg (28%); Contains a mixture of four diastereomers; ^1^H NMR (400 MHz, CDCl_3_): δ = 8.67 (s, 1H), 7.50–7.40 (m, 2H), 7.40–7.29 (m, 4H), 7.20–7.11 (m, 1H), 7.08 (d, *J* = 7.2 Hz, 1H), 6.92–6.88 (m, 1H), 6.55–6.46 (m, 1H), 4.93–4.83 (m, 1H), 4.71 (t, *J* = 7.8 Hz, 1H), 4.62–4.47 (m, 3H), 4.36–4.27 (m, 1H), 4.09 (d, *J* = 11.6 Hz, 1H), 3.98–3.82 (m, 2H), 3.75–3.27 (m, 23H), 2.86–2.61 (m, 3H), 2.54–2.46 (m, 4H), 2.19–2.02 (m, 2H), 0.98–0.93 ppm (m, 12H); ^13^C NMR (101 MHz, CDCl_3_): δ = 172.30, 172.28, 172.09, 172.07, 171.5, 171.4, 171.0, 170.85, 170.82, 169.4, 168.95, 168.91, 168.8, 167.8, 150.4, 148.6, 147.0, 146.9, 138.4, 136.1, 132.8, 132.7, 131.8, 131.7, 131.0, 129.6, 128.3, 116.9, 116.8, 111.72, 111.69, 110.5, 74.65, 74.56, 74.54, 74.46, 74.0, 73.93, 73.86, 73.8, 71.31, 71.27, 71.22, 71.17, 71.15, 70.8, 70.6, 70.4, 70.2, 69.67, 69.66, 69.59, 69.55, 58.82, 58.78, 58.76, 57.14, 57.11, 57.0, 56.9, 56.8, 50.9, 49.1, 49.0, 43.3, 42.7, 41.1, 36.2, 35.2, 35.0, 31.6, 26.5, 22.9, 17.5, 16.2; LC–MS calc. for C_50_H_68_N_10_O_13_S [M+2H]^2+^ is 524.3, found 524.3.

#### 4.9.17 (17*S*)-11-((2-(2-(2-((2-(2,6-dioxopiperidin-3-yl)-1,3-dioxoisoindolin-4-yl)amino)ethoxy)ethoxy)ethoxy)methyl)-17-((2*S*,4*R*)-4-hydroxy-2-((4-(4-methylthiazol-5-yl)benzyl)carbamoyl)pyrrolidine-1-carbonyl)-11,18,18-trimethyl-15-oxo-3,6,9,13-tetraoxa-16-azanonadecyl 2-((*S*)-4-(4-chlorophenyl)-2,3,9-trimethyl-6*H*-thieno[3,2-*f*][1,2,4]triazolo[4,3-*a*][1,4]diazepin-6-yl)acetate (AB3062) (**23**)

Follow General Procedure E, using compound **66** and 1.5 equiv of JQ1-acid (**22**). Purified by HPLC using a linear gradient over 10 min from 25% to 95% MeCN in 0.1% HCOOH in water. Yield: 2.0 mg (26%); Contains a mixture of four diastereomers; ^1^H NMR (500 MHz, CDCl_3_): δ = 9.36–9.09 (m, 1H), 8.76 (s, 1H), 7.50–7.44 (m, 2H), 7.42–7.32 (m, 8H), 7.20–7.13 (m, 1H), 7.09 (d, *J* = 7.0 Hz, 1H), 6.91 (dd, *J* = 5.8, 8.0 Hz, 1H), 6.52 (m, 1H), 4.94–4.80 (m, 1H), 4.73–4.69 (m, 1H), 4.64–4.48 (m, 4H), 4.37–4.26 (m, 3H), 4.11 (d, *J* = 11.7 Hz, 1H), 3.98–3.88 (m, 2H), 3.77–3.70 (m, 4H), 3.68–3.50 (m, 19H), 3.47–3.24 (m, 8H), 2.86–2.66 (m, 5H), 2.54–2.46 (m, 4H), 2.42 (s, 3H), 2.35–2.32 (m, 1H), 2.16 (m, 1H), 2.07 (m, 1H), 1.69 (s, 3H), 0.99–0.94 ppm (m, 12H); HRMS *m*/*z* calc. for C_73_H_91_ClN_11_O_17_S_2_ [M + H]^+^ 1492.5719, found: 1492.5336.

#### 4.9.18 (17*S*)-11-((2-(2-(2-((2-(2,6-dioxopiperidin-3-yl)-1,3-dioxoisoindolin-4-yl)amino)ethoxy)ethoxy)ethoxy)methyl)-17-((2*S*,4*R*)-4-hydroxy-2-(((*S*)-1-(4-(4-methylthiazol-5-yl)phenyl)ethyl)carbamoyl)pyrrolidine-1-carbonyl)-11,18,18-trimethyl-15-oxo-3,6,9,13-tetraoxa-16-azanonadecyl 2-((*S*)-4-(4-chlorophenyl)-2,3,9-trimethyl-6*H*-thieno[3,2-*f*][1,2,4]triazolo[4,3-*a*][1,4]diazepin-6-yl)acetate (AB3066) (**24**)

Follow General Procedure E, using compound **67** and 1.5 equiv of JQ1-acid (**22**). Purified by HPLC using a linear gradient over 10 min from 25% to 95% MeCN in 0.1% HCOOH in water. Yield: 2.6 mg (25%); Contains a mixture of four diastereomers; ^1^H NMR (500 MHz, CDCl_3_): δ = 9.07–8.90 (m, 1H), 8.67 (s, 1H), 7.50–7.36 (m, 8H), 7.32 (d, *J* = 8.3 Hz, 2H), 7.20–7.16 (m, 1H), 7.09 (d, *J* = 6.9 Hz, 1H), 6.91 (dd, *J* = 2.0, 8.6 Hz, 1H), 6.52–6.49 (m, 1H), 5.09 (dq, *J* = 7.2, 7.2 Hz, 1H), 4.92–4.88 (m, 1H), 4.73 (t, *J* = 7.6 Hz, 1H), 4.60 (dd, *J* = 6.6, 7.4 Hz, 1H), 4.56–4.50 (m, 2H), 4.34–4.26 (m, 2H), 4.12 (d, *J* = 10.9 Hz, 1H), 3.95 (s, 2H), 3.76–3.70 (m, 4H), 3.68–3.54 (m, 19H), 3.47–3.29 (m, 8H), 2.87–2.65 (m, 6H), 2.53–2.47 (m, 4H), 2.41 (s, 3H), 2.12–2.06 (m, 2H), 1.69 (s, 3H), 1.48 (d, *J* = 6.7 Hz, 3H), 1.05 (s, 9H), 0.98–0.95 ppm (m, 3H); HRMS *m*/*z* calc. for C_74_H_93_ClN_11_O_17_S_2_ [M + H]^+^ 1506.5875, found: 1506.6135.

#### 4.9.19 (14*S*)-8-((2-(2-(2-((2-(2,6-dioxopiperidin-3-yl)-1,3-dioxoisoindolin-4-yl)amino)ethoxy)ethoxy)ethoxy)methyl)-14-((2*S*,4*R*)-4-hydroxy-2-((4-(4-methylthiazol-5-yl)benzyl)carbamoyl)pyrrolidine-1-carbonyl)-8,15,15-trimethyl-12-oxo-3,6,10-trioxa-13-azahexadecyl 2-((*S*)-4-(4-chlorophenyl)-2,3,9-trimethyl-6*H*-thieno[3,2-*f*][1,2,4]triazolo[4,3-*a*][1,4]diazepin-6-yl)acetate (AB3064) (**25**)

Follow General Procedure E, using compound **68** and 1.5 equiv of JQ1-acid (**22**). Purified by HPLC using a linear gradient over 10 min from 25% to 95% MeCN in 0.1% HCOOH in water. Yield: 2.0 mg (32%); Contains a mixture of four diastereomers; ^1^H NMR (500 MHz, CDCl_3_): δ = 9.25–8.99 (m, 1H), 8.69 (s, 1H), 7.50–7.43 (m, 2H), 7.40 (d, *J* = 8.1 Hz, 2H), 7.37–7.34 (m, 4H), 7.33 (d, *J* = 8.1 Hz, 2H), 7.19–7.13 (m, 1H), 7.09 (d, *J* = 6.9 Hz, 1H), 6.91 (dd, *J* = 6.0, 8.4 Hz, 1H), 6.55–6.48 (m, 1H), 4.92–4.82 (m, 1H), 4.76–4.71 (m, 1H), 4.63–4.48 (m, 4H), 4.37–4.24 (m, 3H), 4.11 (d, *J* = 10.9 Hz, 1H), 3.98–3.88 (m, 2H), 3.75–3.69 (m, 4H), 3.68–3.51 (m, 15H), 3.47–3.27 (m, 8H), 2.86–2.66 (m, 5H), 2.53–2.46 (m, 4H), 2.41 (s, 3H), 2.36–2.33 (m, 1H), 2.19–2.14 (m, 1H), 2.11–2.05 (m, 1H), 1.69 (s, 3H), 0.97–0.93 ppm (m, 12H); HRMS *m*/*z* calc. for C_71_H_87_ClN_11_O_16_S_2_ [M + H]^+^ 1448.5457, found: 1448.5256.

#### 4.9.20 (17*S*)-11-((2-(2-(2-((2-(2,6-dioxopiperidin-3-yl)-6-fluoro-1,3-dioxoisoindolin-5-yl)amino)ethoxy)ethoxy)ethoxy)methyl)-17-((2*S*,4*R*)-4-hydroxy-2-((4-(4-methylthiazol-5-yl)benzyl)carbamoyl)pyrrolidine-1-carbonyl)-11,18,18-trimethyl-15-oxo-3,6,9,13-tetraoxa-16-azanonadecyl 2-((*S*)-4-(4-chlorophenyl)-2,3,9-trimethyl-6*H*-thieno[3,2-*f*][1,2,4]triazolo[4,3-*a*][1,4]diazepin-6-yl)acetate (AB3063) (**26**)

Follow General Procedure E, using compound **69** and 1.5 equiv of JQ1-acid (**22**). Purified by HPLC using a linear gradient over 10 min from 25% to 95% MeCN in 0.1% HCOOH in water. Yield: 2.6 mg (25%); Contains a mixture of four diastereomers; ^1^H NMR (500 MHz, CDCl_3_): δ = 8.68 (s, 1H), 8.58–8.53 (m, 1H), 7.46–7.38 (m, 8H), 7.35 (d, *J* = 2.2 Hz, 8H), 7.32 (d, *J* = 8.8 Hz, 2H), 7.17–7.13 (m, 1H), 7.13–7.10 (m, 1H), 5.28–5.23 (m, 1H), 4.89 (dd, *J* = 5.1, 12.5 Hz, 1H), 4.72 (t, *J* = 7.9 Hz, 1H), 4.62–4.52 (m, 3H), 4.51–4.47 (m, 1H), 4.38–4.25 (m, 3H), 4.09 (d, *J* = 11.7 Hz, 1H), 3.95–3.86 (m, 2H), 3.77–3.72 (m, 4H), 3.70–3.51 (m, 19H), 3.48–3.42 (m, 2H), 3.41–3.27 (m, 6H), 2.86 (d, *J* = 17.2 Hz, 1H), 2.82–2.66 (m, 5H), 2.55–2.48 (m, 4H), 2.41 (s, 3H), 2.20–2.09 (m, 2H), 1.68 (s, 3H), 0.96–0.94 ppm (m, 12H); ^13^C NMR (126 MHz, CDCl_3_): δ = 171.7, 171.4, 171.3, 171.0, 170.6, 170.5, 168.44, 168.40, 167.6, 164.0, 155.4, 153.99 (d, *J*_C–F_ = 248.5 Hz), 150.5, 150.1, 148.4, 142.89 (d, *J*_C–F_ = 13.2 Hz), 138.5, 137.0, 136.7, 132.3, 131.9, 131.1, 131.0, 130.9, 130.6, 130.18 (d, *J*_C–F_ = 2.2 Hz), 130.0, 129.59, 129.58, 128.8, 128.3, 118.70 (d, *J*_C–F_ = 6.2 Hz), 110.4, 110.30 (d, *J*_C–F_ = 21.0 Hz), 106.02–105.96 (m), 74.52, 74.50, 74.48, 73.99, 73.98, 73.9, 73.83, 73.81, 73.79, 71.23, 71.19, 71.1, 70.9, 70.84, 70.81, 70.75, 70.72, 70.6, 70.5, 70.3, 69.21, 69.18, 64.2, 58.8, 58.7, 57.1, 57.05, 57.03, 56.8, 53.9, 49.4, 43.4, 43.1, 41.1, 36.9, 36.3, 35.2, 35.2, 31.6, 26.5, 22.9, 17.6, 16.1, 14.6, 13.3, 11.9; ^19^F{^1^H} NMR (471 MHz, CDCl_3_) δ = −127.25*, −127.28*, −127.30*, −127.33* (1F); HRMS *m*/*z* calc. for C_73_H_90_ClFN_11_O_17_S_2_ [M + H]^+^ 1510.5625, found: 1510.5262.

#### 4.9.21 (17S)-11-((2-(2-(2-((2-(2,6-dioxopiperidin-3-yl)-6-fluoro-1,3-dioxoisoindolin-5-yl)amino)ethoxy)ethoxy)ethoxy)methyl)-17-((2*S*,4*R*)-4-hydroxy-2-(((*S*)-1-(4-(4-methylthiazol-5-yl)phenyl)ethyl)carbamoyl)pyrrolidine-1-carbonyl)-11,18,18-trimethyl-15-oxo-3,6,9,13-tetraoxa-16-azanonadecyl 2-((*S*)-4-(4-chlorophenyl)-2,3,9-trimethyl-6*H*-thieno[3,2-*f*][1,2,4]triazolo[4,3-*a*][1,4]diazepin-6-yl)acetate (AB3067) (**27**)

Follow General Procedure E, using compound **70** and 1.5 equiv of JQ1-acid (**22**). Purified by HPLC using a linear gradient over 10 min from 30% to 95% MeCN in 0.1% HCOOH in water. Yield: 2.4 mg (21%); Contains a mixture of four diastereomers; ^1^H NMR (500 MHz, CDCl_3_): δ = 8.68 (s, 1H), 8.64–8.58 (m, 1H), 7.50–7.45 (m, 1H), 7.41–7.35 (m, 7H), 7.32 (d, *J* = 8.6 Hz, 2H), 7.22–7.18 (m, 1H), 7.11 (d, *J*_H–F_ = 6.9 Hz, 1H), 5.31–5.25 (m, 1H), 5.08 (dq, *J* = 7.2, 7.2 Hz, 1H), 4.90 (dd, *J* = 5.5, 12.4 Hz, 1H), 4.72 (t, *J* = 7.9 Hz, 1H), 4.60 (dd, *J* = 6.1, 7.9 Hz, 1H), 4.56–4.49 (m, 2H), 4.35–4.25 (m, 2H), 4.09 (d, *J* = 11.0 Hz, 1H), 3.95 (d, *J* = 15.9 Hz, 1H), 3.92 (d, *J* = 16.4 Hz, 1H), 3.77–3.71 (m, 4H), 3.67–3.54 (m, 19H), 3.48–3.30 (m, 8H), 2.90–2.70 (m, 3H), 2.66 (s, 3H), 2.52–2.45 (m, 4H), 2.41 (s, 3H), 2.14–2.07 (m, 2H), 1.68 (s, 3H), 1.49–1.45 (m, 3H), 1.05 (s, 9H), 0.98–0.96 ppm (m, 3H); ^13^C NMR (126 MHz, CDCl_3_): δ = 171.7, 171.49, 171.46, 171.38, 171.36, 170.7, 170.0, 168.54, 168.49, 167.6, 167.04, 167.02, 164.0, 162.7, 155.4, 153.97 (d, *J*_C–F_ = 248.7 Hz), 150.5, 150.1, 148.5, 143.5, 142.87 (d, *J*_C–F_ = 12.5 Hz), 136.9, 136.7, 132.3, 131.8, 131.04, 131.02, 130.8, 130.6, 130.16 (d, *J*_C–F_ = 2.1 Hz), 130.04, 130.00, 129.6, 128.8, 126.6, 118.65 (d, *J*_C–F_ = 8.3 Hz), 110.28 (d, *J*_C–F_ = 22.5 Hz), 105.96 (d, *J*_C–F_ = 5.3 Hz), 74.5, 74.0, 73.9, 73.8, 72.7, 71.2, 71.1, 70.9, 70.84, 70.76, 70.7, 70.6, 70.5, 70.2, 69.2, 69.11, 69.09, 69.08, 64.2, 58.64, 58.61, 57.1, 56.8, 53.8, 49.4, 49.0, 43.0, 41.1, 36.9, 35.8, 35.3, 31.6, 26.6, 22.9, 22.4, 17.6, 16.2, 14.5, 13.2, 11.9; ^19^F{^1^H} NMR (471 MHz, CDCl_3_) δ = −127.22*, −127.26*, −127.26*, −127.29* (1F); HRMS *m*/*z* calc. for C_74_H_92_ClFN_11_O_17_S_2_ [M + H]^+^ 1524.5781, found: 1524.5365.

#### 4.9.22 (14*S*)-8-((2-(2-(2-((2-(2,6-dioxopiperidin-3-yl)-6-fluoro-1,3-dioxoisoindolin-5-yl)amino)ethoxy)ethoxy)ethoxy)methyl)-14-((2*S*,4*R*)-4-hydroxy-2-((4-(4-methylthiazol-5-yl)benzyl)carbamoyl)pyrrolidine-1-carbonyl)-8,15,15-trimethyl-12-oxo-3,6,10-trioxa-13-azahexadecyl 2-((*S*)-4-(4-chlorophenyl)-2,3,9-trimethyl-6*H*-thieno[3,2-*f*][1,2,4]triazolo[4,3-*a*][1,4]diazepin-6-yl)acetate (AB3065) (**28**)

Follow General Procedure E, using compound **71** and 1.5 equiv of JQ1-acid (**22**). Purified by HPLC using a linear gradient over 10 min from 25% to 95% MeCN in 0.1% HCOOH in water. Yield: 3.1 mg (27%); ^1^H NMR (500 MHz, CDCl_3_): δ = 8.67 (s, 1H), 8.67–8.48 (m, 1H), 7.45–7.38 (m, 4H), 7.37–7.34 (m, 4H), 7.32 (d, *J* = 8.4 Hz, 2H), 7.17–7.07 (m, 2H), 5.27–5.23 (m, 1H), 4.89 (dd, *J* = 4.7, 12.3 Hz, 1H), 4.74 (t, *J* = 8.1 Hz, 1H), 4.62–4.47 (m, 4H), 4.40–4.24 (m, 3H), 4.10 (d, *J* = 12.4 Hz, 1H), 3.94 (d, *J* = 15.1 Hz, 1H), 3.89 (d, *J* = 15.0 Hz, 1H), 3.75 (t, *J* = 5.2 Hz, 2H), 3.71 (t, *J* = 5.0 Hz, 2H), 3.69–3.54 (m, 15H), 3.47–3.30 (m, 8H), 2.88–2.66 (m, 5H), 2.53–2.47 (m, 4H), 2.41 (s, 3H), 2.38–2.34 (m, 1H), 2.22–2.10 (m, 2H), 1.69 (s, 3H), 0.97–0.93 ppm (m, 12H); ^19^F NMR (471 MHz, CDCl_3_) δ = −127.23–127.34 (m, 1F); HRMS *m*/*z* calc. for C_71_H_86_ClFN_11_O_16_S_2_ [M + H]^+^ 1466.5362, found: 1466.5243.

#### 4.9.23 (17*S*)-11-((2-(2-((2-(2,6-dioxopiperidin-3-yl)-6-fluoro-1,3-dioxoisoindolin-5-yl)amino)ethoxy)ethoxy)methyl)-17-((2*S*,4*R*)-4-hydroxy-2-((4-(4-methylthiazol-5-yl)benzyl)carbamoyl)pyrrolidine-1-carbonyl)-11,18,18-trimethyl-15-oxo-3,6,9,13-tetraoxa-16-azanonadecyl 2-((*S*)-4-(4-chlorophenyl)-2,3,9-trimethyl-6*H*-thieno[3,2-*f*][1,2,4]triazolo[4,3-*a*][1,4]diazepin-6-yl)acetate (AB3126) (**29**)

Follow General Procedure E, using compound **72** and 1.5 equiv of JQ1-acid (**22**). Purified by HPLC using a linear gradient over 15 min from 10% to 95% MeCN in 0.1% HCOOH in water. Yield: 3.1 mg (27%); ^1^H NMR (500 MHz, CDCl_3_): δ = 8.68 (s, 1H), 8.66–8.57 (m, 1H), 7.49–7.44 (m, 1H), 7.43–7.39 (m, 3H), 7.36 (s, 4H), 7.32 (d, *J* = 8.0 Hz, 2H), 7.17 (d, *J* = 7.9 Hz, 1H), 7.14–7.10 (m, 1H), 5.28–5.22 (m, 1H), 4.89 (d, *J* = 12.1 Hz, 1H), 4.73 (t, *J* = 7.8 Hz, 1H), 4.63–4.47 (m, 4H), 4.39–4.27 (m, 3H), 4.11–4.08 (m, 1H), 3.98–3.86 (m, 2H), 3.76–3.70 (m, 4H), 3.69–3.51 (m, 15H), 3.47–3.25 (m, 8H), 2.87–2.65 (m, 6H), 2.52–2.46 (m, 4H), 2.41 (s, 3H), 2.21–2.10 (m, 2H), 1.67 (s, 3H), 0.96 (s, 9H), 0.93 ppm (s, 3H); ^19^F{^1^H} NMR (471 MHz, CDCl_3_) δ = −127.35*, −127.36*, −127.41*, −127.41* (1F); HRMS *m*/*z* calc. for C_71_H_86_ClFN_11_O_16_S_2_ [M + H]^+^ 1466.5362, found: 1466.5441.

#### 4.9.24 (14*S*)-8-((2-(2-((2-(2,6-dioxopiperidin-3-yl)-6-fluoro-1,3-dioxoisoindolin-5-yl)amino)ethoxy)ethoxy)methyl)-14-((2*S*,4*R*)-4-hydroxy-2-((4-(4-methylthiazol-5-yl)benzyl)carbamoyl)pyrrolidine-1-carbonyl)-8,15,15-trimethyl-12-oxo-3,6,10-trioxa-13-azahexadecyl 2-((*S*)-4-(4-chlorophenyl)-2,3,9-trimethyl-6*H*-thieno[3,2-*f*][1,2,4]triazolo[4,3-*a*][1,4]diazepin-6-yl)acetate (AB3125) (**30**)

Follow General Procedure E, using compound **73** and 1.5 equiv of JQ1-acid (**22**). Purified by HPLC using a linear gradient over 15 min from 10% to 95% MeCN in 0.1% HCOOH in water. Yield: 3.2 mg (44%); ^1^H NMR (500 MHz, CDCl_3_): δ = 8.68 (s, 1H), 8.61–8.52 (m, 1H), 7.50–7.45 (m, 1H), 7.43–7.34 (m, 7H), 7.32 (d, *J* = 8.7 Hz, 2H), 7.20–7.16 (m, 1H), 7.14–7.11 (m, 1H), 5.31–5.26 (m, 1H), 4.92–4.87 (m, 1H), 4.73 (t, *J* = 8.1 Hz, 1H), 4.62–4.54 (m, 3H), 4.51–4.48 (m, 1H), 4.37–4.29 (m, 2H), 4.28–4.23 (m, 1H), 4.13–4.09 (m, 1H), 3.96–3.86 (m, 2H), 3.74–3.69 (m, 4H), 3.69–3.54 (m, 11H), 3.47–3.27 (m, 8H), 2.88–2.65 (m, 5H), 2.52–2.47 (m, 4H), 2.41 (s, 3H), 2.37–2.33 (m, 1H), 2.22–2.16 (m, 1H), 2.13–2.10 (m, 1H), 1.68 (s, 3H), 0.96 (s, 9H), 0.94–0.91 ppm (m, 3H); ^19^F{^1^H} NMR (471 MHz, CDCl_3_) δ = −127.34*, −127.34*, −127.39*, −127.40* (1F); HRMS *m*/*z* calc. for C_69_H_82_ClFN_11_O_15_S_2_ [M + H]^+^ 1422.5100, found: 1422.5332.

#### 4.9.25 (17*S*)-11-((2-(2-(2-(4-(2-(2,6-dioxopiperidin-3-yl)-1,3-dioxoisoindolin-4-yl)piperazin-1-yl)ethoxy)ethoxy)ethoxy)methyl)-17-((2*S*,4*R*)-4-hydroxy-2-((4-(4-methylthiazol-5-yl)benzyl)carbamoyl)pyrrolidine-1-carbonyl)-11,18,18-trimethyl-15-oxo-3,6,9,13-tetraoxa-16-azanonadecyl 2-((*S*)-4-(4-chlorophenyl)-2,3,9-trimethyl-6*H*-thieno[3,2-*f*][1,2,4]triazolo[4,3-*a*][1,4]diazepin-6-yl)acetate (AB3029) (**31**)

To solution of Boc-protected compound **76** (55 mg, 0.12 mmol) in DCM (2 mL), was added 4 N HCl in dioxane (0.62 mL, 2.48 mmol) and the solution was left to stir at r.t. for 16 h. The reaction was then evaporated to dryness to quantitatively yield an amine intermediate as a HCl salt (46 mg, 0.12 mmol). The amine intermediate (3.4 mg, 9.1 μmol) was dissolved in DMF (100 μL) followed by the addition of DIPEA (5.4 μL, 30 μmol). This was then added to a solution containing mesylate **75** (5.3 mg, 4.0 μmol) in DMF (200 μL) and stirred for 16 h at 80 °C. The reaction was concentrated in vacuo and purified by HPLC using a linear gradient over 10 min from 5% to 95% MeCN in 0.1% HCOOH in water. Yield: 4.0 mg (68%); Contains a mixture of four diastereomers; ^1^H NMR (500 MHz, CDCl_3_): δ = 8.75–8.66 (m, 2H), 7.59 (t, *J* = 7.7 Hz, 1H), 7.45–7.39 (m, 4H), 7.36 (s, 4H), 7.32 (d, *J* = 8.5 Hz, 2H), 7.18–7.15 (m, 2H), 4.96–4.91 (m, 1H), 4.74–4.70 (m, 1H), 4.62–4.49 (m, 4H), 4.39–4.26 (m, 3H), 4.09 (d, *J* = 11.4 Hz, 1H), 3.96 (d, *J* = 15.3 Hz, 1H), 3.92 (d, *J* = 15.1 Hz, 1H), 3.75–3.72 (m, 2H), 3.69–3.53 (m, 23H), 3.43–3.29 (m, 10H), 2.88–2.66 (m, 11H), 2.54–2.48 (m, 4H), 2.41 (s, 3H), 2.19–2.13 (m, 1H), 2.12–2.06 (m, 1H), 1.69 (s, 3H), 0.98–0.96 ppm (m, 12H); HRMS *m*/*z* calc. for C_77_H_98_ClN_12_O_17_S_2_ [M + H]^+^ 1561.6297, found: 1561.6552.

#### 4.9.26 (17*S*)-11-((2-(2-(2-(4-(2-(2,6-dioxopiperidin-3-yl)-6-fluoro-1,3-dioxoisoindolin-5-yl)piperazin-1-yl)ethoxy)ethoxy)ethoxy)methyl)-17-((2*S*,4*R*)-4-hydroxy-2-((4-(4-methylthiazol-5-yl)benzyl)carbamoyl)pyrrolidine-1-carbonyl)-11,18,18-trimethyl-15-oxo-3,6,9,13-tetraoxa-16-azanonadecyl 2-((*S*)-4-(4-chlorophenyl)-2,3,9-trimethyl-6*H*-thieno[3,2-*f*][1,2,4]triazolo[4,3-*a*][1,4]diazepin-6-yl)acetate (AB3030) (**32**)

To solution of Boc-protected compound **77** (77 mg, 0.16 mmol) in DCM (1.3 mL), was added 4 N HCl in dioxane (0.84 mL, 3.3 mmol) and the solution was left to stir at r.t. for 16 h. The reaction was then evaporated to dryness to quantitatively yield an amine intermediate as a HCl salt (63 mg, 0.16 mmol). The amine intermediate (3.6 mg, 9.1 μmol) was dissolved in DMF (100 μL) followed by the addition of DIPEA (5.4 μL, 30 μmol). This was then added to a solution containing mesylate **75** (5.3 mg, 4.0 μmol) in DMF (200 μL) and stirred for 16 h at 80 °C. The reaction was concentrated in vacuo and purified by HPLC using a linear gradient over 10 min from 5% to 95% MeCN in 0.1% HCOOH in water. Yield: 4.1 mg (68%); Contains a mixture of four diastereomers; ^1^H NMR (500 MHz, CDCl_3_): δ = 8.68 (s, 1H), 8.28 (d, *J* = 31.3 Hz, 1H), 7.49–7.36 (m, 9H), 7.32 (d, *J* = 8.6 Hz, 2H), 7.15 (d, *J* = 8.0 Hz, 1H), 4.92 (dd, *J* = 5.2, 12.2 Hz, 1H), 4.73 (t, *J* = 7.7 Hz, 1H), 4.62–4.53 (m, 3H), 4.50 (d, *J* = 8.4 Hz, 1H), 4.40–4.26 (m, 3H), 4.08 (d, *J* = 11.1 Hz, 1H), 3.98–3.88 (m, 2H), 3.76–3.72 (m, 2H), 3.70–3.53 (m, 23H), 3.44–3.29 (m, 10H), 2.91–2.66 (m, 11H), 2.51 (s, 4H), 2.41 (s, 3H), 2.20–2.12 (m, 2H), 1.68 (s, 3H), 0.98–0.94 ppm (m, 12H); ^19^F NMR (471 MHz, CDCl_3_) δ = −110.75–110.81 (m, 1F); HRMS *m*/*z* calc. for C_77_H_97_ClFN_12_O_17_S_2_ [M + H]^+^ 1579.6203, found: 1579.6465.

#### 4.9.27 2,5,7,10,13-Pentaoxapentadecan-15-ol (**35**)

Follow General Procedure F, using triethylene glycol (**33**) and MEMCl. Purified by flash column chromatography (80 g silica column) using a linear gradient from 0% to 20% MeOH in DCM. Yield: 3.15 g (66%); ^1^H NMR (500 MHz, CDCl_3_): δ = 4.76 (s, 2H), 3.75–3.70 (m, 6H), 3.70–3.65 (m, 6H), 3.63–3.60 (m, 2H), 3.58–3.55 (m, 2H), 3.39 (s, 3H), 2.39 ppm (t, *J* = 6.3 Hz, 1H); ^13^C NMR (126 MHz, CDCl_3_): δ = 95.8, 72.6, 71.9, 70.8, 70.7, 70.6, 67.0, 67.0, 61.9, 59.1.

#### 4.9.28 2,5,7,10-Tetraoxadodecan-12-ol (**36**)

Follow General Procedure F, using diethylene glycol (**34**) and MEMCl. Purified by flash column chromatography (24 g silica column) using a linear gradient from 0% to 6% MeOH in DCM. Yield: 615 mg (40%); ^1^H NMR (500 MHz, CDCl_3_): δ = 4.76 (s, 2H), 3.76–3.70 (m, 6H), 3.70–3.67 (m, 2H), 3.61 (dd, *J* = 4.6, 4.6 Hz, 2H), 3.56 (dd, *J* = 4.7, 4.7 Hz, 2H), 3.39 (3H, s), 2.31 ppm (dt, *J* = 2.3, 6.3 Hz, 1H); ^13^C NMR (126 MHz, CDCl_3_): δ = 95.8, 72.5, 72.0, 70.6, 67.2, 67.1, 62.0, 59.1.

#### 4.9.29 2,4,7,10-Tetraoxadodecan-12-ol (**37**)

Follow General Procedure F, using triethylene glycol (**33**) and MOMBr. Purified by flash column chromatography (80 g silica column) using a linear gradient from 0% to 8% MeOH in DCM. Yield: 2.86 g (74%); ^1^H NMR (500 MHz, CDCl_3_): δ = 4.66 (s, 2H), 3.75–3.66 (m, 10H), 3.61 (dd, *J* = 4.5, 4.5 Hz, 2H), 3.37 (s, 3H), 2.44 ppm (t, *J* = 6.3 Hz, 1H); ^13^C NMR (126 MHz, CDCl_3_): δ = 96.7, 72.6, 70.8, 70.7, 70.6, 66.9, 61.9, 55.4.

#### 4.9.30 2,5,7,10,13-Pentaoxapentadecan-15-yl methanesulfonate (**38**)

Follow General Procedure G, using alcohol **35**. Yield: 729 mg (37%); ^1^H NMR (400 MHz, CDCl_3_): δ = 4.75 (s, 2H), 4.39–4.36 (m, 2H), 3.78–3.75 (m, 2H), 3.73–3.63 (m, 10H), 3.57–3.54 (m, 2H), 3.39 (s, 3H), 3.07 ppm (s, 3H); ^13^C NMR (101 MHz, CDCl_3_): δ = 95.8, 71.9, 70.8, 70.7, 69.3, 69.2, 67.0, 59.1, 37.9.

#### 4.9.31 2,5,7,10-Tetraoxadodecan-12-yl methanesulfonate (**39**)

Follow General Procedure G, using alcohol **36**. Yield: 659 mg (77%); ^1^H NMR (500 MHz, CDCl_3_): δ = 4.74 (s, 2H), 4.39–4.37 (m, 2H), 3.79–3.76 (m, 2H), 3.75–3.66 (m, 6H), 3.57–3.54 (m, 2H), 3.39 (s, 3H), 3.06 ppm (s, 3H); ^13^C NMR (126 MHz, CDCl_3_): δ = 95.8, 71.9, 70.8, 69.22, 69.16, 67.1, 67.0, 59.2, 37.8.

#### 4.9.32 2,4,7,10-Tetraoxadodecan-12-yl methanesulfonate (**40**)

Follow General Procedure G, using alcohol **37**. Yield: 1.14 g (81%); ^1^H NMR (500 MHz, CDCl_3_): δ = 4.65 (s, 2H), 4.39–4.37 (m, 2H), 3.79–3.76 (m, 2H), 3.71–3.63 (m, 8H), 3.37 (s, 3H), 3.07 ppm (s, 3H); ^13^C NMR (126 MHz, CDCl_3_): δ = 96.7, 70.9, 70.8, 70.7, 69.3, 69.2, 66.9, 55.4, 37.9.

#### 4.9.33 3-(allyloxy)-2-((2-(2-(2-azidoethoxy)ethoxy)ethoxy)methyl)-2-methylpropan-1-ol (**41**)

Follow General Procedure A, using 1.0 eq. diol **5**, 1.2 eq. of NaH and 1.0 eq. of 2-(2-(2-azidoethoxy)ethoxy)ethylmethanesulfonate (**6**). Purified by reverse phase flash column chromatography (50 g C18 gold column) using a linear gradient over 11 min from 0% to 100% MeCN in 0.1% HCOOH in water. Yield: 315 mg (60%); ^1^H NMR (500 MHz, CDCl_3_): δ = 5.93–5.84 (m, 1H), 5.26 (qd, *J* = 1.7, 17.4 Hz, 1H), 5.16 (qd, *J* = 1.4, 10.6 Hz, 1H), 3.97 (td, *J* = 1.3, 5.3 Hz, 2H), 3.69–3.59 (m, 11H), 3.56 (s, 2H), 3.51 (d, *J* = 9.0 Hz, 1H), 3.44–3.38 (m, 5H), 2.99 (br s, 1H), 0.88 ppm (s, 3H); ^13^C NMR (126 MHz, CDCl_3_): δ = 135.0, 116.7, 76.0, 74.7, 72.6, 71.1, 70.9, 70.8, 70.6, 70.2, 69.2, 50.9, 40.8, 17.7.

#### 4.9.34 3-(allyloxy)-2-((2-(2-azidoethoxy)ethoxy)methyl)-2-methylpropan-1-ol (**42**)

Follow General Procedure A, using 1.0 eq. diol **5**, 1.2 eq. of NaH and 1.0 eq. of 2-(2-azidoethoxy)ethylmethanesulfonate (**7**). Purified by reverse phase flash column chromatography (50 g C18 gold column) using a linear gradient over 10 min from 0% to 100% MeCN in 0.1% HCOOH in water. Yield: 244 mg (33%); ^1^H NMR (400 MHz, CDCl_3_): δ = 5.95–5.84 (m, 1H), 5.26 (dd, *J* = 1.3, 17.2 Hz, 1H), 5.16 (d, *J* = 10.3 Hz, 1H), 3.97 (d, *J* = 5.3 Hz, 2H), 3.70–3.59 (m, 6H), 3.57 (s, 2H), 3.53 (d, *J* = 9.1 Hz, 1H), 3.47–3.35 (m, 5H), 2.91 (br s, 1H), 0.88 ppm (s, 3H); ^13^C NMR (101 MHz, CDCl_3_): δ = 134.9, 116.8, 76.0, 74.9, 72.6, 71.2, 70.6, 70.2, 69.3, 50.9, 40.8, 17.7.

#### 4.9.35 15-((allyloxy)methyl)-25-azido-15-methyl-2,4,7,10,13,17,20,23-octaoxapentacosane (**43**)

Follow General Procedure A, using 1.0 eq of alcohol **41**, 1.5 eq. of NaH and 1.5 eq. of mesylate **40**. Purified by reverse phase flash column chromatography (15.5 g C18 gold column) using a linear gradient over 10 min from 0% to 100% MeCN in 0.1% HCOOH in water. Yield: 78 mg (50%); ^1^H NMR (500 MHz, CDCl_3_): δ = 5.92–5.83 (m, 1H), 5.24 (dd, *J* = 1.5, 17.3 Hz, 1H), 5.13 (dd, *J* = 1.2, 10.5 Hz, 1H), 4.65 (s, 2H), 3.94 (d, *J* = 5.4 Hz, 2H), 3.70–3.60 (m, 18H), 3.58–3.53 (m, 4H), 3.40–3.36 (m, 5H), 3.33 (s, 4H), 3.29 (s, 2H), 0.94 ppm (s, 3H); ^13^C NMR (126 MHz, CDCl_3_): δ = 135.5, 116.2, 96.7, 74.1, 73.2, 72.4, 71.24, 71.22, 71.0, 70.9, 70.85, 70.82, 70.7, 70.6, 70.2, 67.0, 55.3, 50.9, 41.1, 17.5.

#### 4.9.36 15-((allyloxy)methyl)-25-azido-15-methyl-2,5,7,10,13,17,20,23-octaoxapentacosane (**44**)

Follow General Procedure A, using 1.0 eq of alcohol **40**, 1.5 eq. of NaH and 1.5 eq. of mesylate **39**. Purified by reverse phase flash column chromatography (15.5 g C18 gold column) using a linear gradient over 10 min from 0% to 100% MeCN in 0.1% HCOOH in water. Yield: 82 mg (53%); ^1^H NMR (500 MHz, CDCl_3_): δ = 5.92–5.84 (m, 1H), 5.24 (dd, *J* = 1.6, 17.2 Hz, 1H), 5.13 (dd, *J* = 1.1, 10.4 Hz, 1H), 4.75 (s, 2H), 3.94 (d, *J* = 5.3 Hz, 2H), 3.72–3.60 (m, 16H), 3.58–3.54 (m, 6H), 3.40–3.36 (m, 5H), 3.33 (s, 4H), 3.29 (s, 2H), 0.94 ppm (s, 3H)); ^13^C NMR (126 MHz, CDCl_3_): δ = 135.5, 116.2, 95.8, 74.1, 73.2, 72.4, 72.0, 71.2, 71.0, 70.9, 70.7, 70.65, 70.58, 70.2, 67.2, 67.0, 59.1, 50.9, 41.1, 17.6.

#### 4.9.37 15-((allyloxy)methyl)-22-azido-15-methyl-2,4,7,10,13,17,20-heptaoxadocosane (**45**)

Follow General Procedure A, using 1.0 eq of alcohol **42**, 1.5 eq. of NaH and 1.5 eq. of mesylate **40**. Purified by reverse phase flash column chromatography (30 g C18 gold column) using a linear gradient over 10 min from 0% to 100% MeCN in 0.1% HCOOH in water. Yield: 130 mg (52%); ^1^H NMR (400 MHz, CDCl_3_): δ = 5.94–5.83 (m, 1H), 5.25 (dd, *J* = 1.6, 17.3 Hz, 1H), 5.14 (dd, *J* = 1.4, 10.3 Hz, 1H), 3.94 (d, *J* = 5.3 Hz, 2H), 3.71–3.53 (m, 18H), 3.40–3.29 (m, 11H), 0.96 ppm (s, 3H); ^13^C NMR (101 MHz, CDCl_3_): δ = 135.5, 116.2, 96.7, 74.2, 74.1, 73.2, 72.4, 71.3, 71.2, 70.9, 70.8, 70.7, 70.6, 70.2, 67.0, 55.3, 51.0, 41.1, 17.6.

#### 4.9.38 15-((allyloxy)methyl)-22-azido-15-methyl-2,5,7,10,13,17,20-heptaoxadocosane (**46**)

Follow General Procedure A, using 1.0 eq of alcohol **42**, 1.5 eq. of NaH and 1.5 eq. of mesylate **39**. Purified by reverse phase flash column chromatography (30 g C18 gold column) using a linear gradient over 10 min from 0% to 100% MeCN in 0.1% HCOOH in water. Yield: 111 mg (56%); ^1^H NMR (400 MHz, CDCl_3_): δ = 5.94–5.84 (m, 1H), 5.25 (dd, *J* = 1.5, 17.2 Hz, 1H), 5.13 (dd, *J* = 1.1, 10.5 Hz, 1H), 3.94 (d, *J* = 5.5 Hz, 2H), 3.73–3.55 (m, 18H), 3.40–3.34 (m, 9H), 3.30 (s, 2H), 0.95 ppm (s, 3H); ^13^C NMR (101 MHz, CDCl_3_): δ = 135.5, 116.2, 95.8, 74.2, 74.1, 73.2, 72.4, 72.0, 71.3, 71.2, 70.7, 70.65, 70.59, 70.2, 67.2, 67.0, 59.1, 51.0, 41.1, 17.6.

#### 4.9.39 18-((allyloxy)methyl)-18-methyl-2,5,7,10,13,16,20,23,26,29,31,34-dodecaoxapentatriacontane (**47**)

Follow General Procedure A, using 1.0 eq of diol **5**, 4 eq. of NaH and 3 eq. of mesylate **38**. Purified by reverse phase flash column chromatography (50 g C18 gold column) using a linear gradient over 11 min from 0% to 100% MeCN in 0.1% HCOOH in water. Yield: 205 mg (55%); ^1^H NMR (400 MHz, CDCl_3_): δ = 5.93–5.83 (m, 1H), 5.24 (qd, *J* = 1.7, 17.2 Hz, 1H), 5.13 (qd, *J* = 1.5, 10.4 Hz, 1H), 4.75 (s, 4H), 3.94 (td, *J* = 1.5, 5.4 Hz, 2H), 3.74–3.60 (m, 24H), 3.57–3.53 (m, 8H), 3.39 (s, 6H), 3.32 (s, 4H), 3.29 (s, 2H), 0.94 ppm (s, 3H); ^13^C NMR (101 MHz, CDCl_3_): δ = 135.5, 116.2, 95.8, 74.1, 73.2, 72.4, 72.0, 71.2, 70.84, 70.81, 70.7, 70.6, 67.1, 67.0, 59.1, 41.1, 17.6.

#### 4.9.40 15-((2-(2-(2-azidoethoxy)ethoxy)ethoxy)methyl)-15-methyl-2,4,7,10,13,17-hexaoxanonadecan-19-al (**48**)

Follow General Procedure B, using alkene **43**. Yield: 200 mg (89%); ^1^H NMR (500 MHz, CDCl_3_): δ = 9.71 (s, 1H), 4.63 (s, 2H), 4.00 (d, *J* = 0.8 Hz, 2H), 3.70–3.28 (m, 32H), 0.95 ppm (s, 3H); ^13^C NMR (126 MHz, CDCl_3_): δ = 202.2, 96.7, 77.0, 74.7, 73.9, 71.22, 71.20, 70.9, 70.83, 70.81, 70.7, 70.6, 70.2, 67.0, 55.3, 50.9, 41.3, 17.5.

#### 4.9.41 15-((2-(2-(2-azidoethoxy)ethoxy)ethoxy)methyl)-15-methyl-2,5,7,10,13,17-hexaoxanonadecan-19-al (**49**)

Follow General Procedure B, using alkene **44**. Yield: 62 mg (76%); ^1^H NMR (500 MHz, CDCl_3_): δ = 9.71 (s, 1H), 4.72 (s, 2H), 4.00 (s, 2H), 3.73–3.29 (m, 33H), 0.95 ppm (s, 3H); ^13^C NMR (126 MHz, CDCl_3_): 202.1, 95.8, 77.0, 74.7, 73.90, 73.86, 71.9, 71.2, 70.9, 70.8, 70.7, 70.65, 70.57, 70.2, 67.1, 67.0, 59.1, 50.9, 41.3, 17.5.

#### 4.9.42 15-((2-(2-azidoethoxy)ethoxy)methyl)-15-methyl-2,4,7,10,13,17-hexaoxanonadecan-19-al (**50**)

Follow General Procedure B, using alkene **45**. Yield: 62 mg (76%); ^1^H NMR (400 MHz, CDCl_3_): δ = 9.74 (s, 1H), 4.66 (s, 2H), 4.02 (s, 2H), 3.72–3.30 (m, 29H), 0.99 ppm (s, 3H); ^13^C NMR (101 MHz, CDCl_3_): δ = 202.1, 96.7, 77.0, 74.7, 74.0, 73.9, 71.25, 71.21, 70.84, 70.81, 70.75, 70.7, 70.6, 70.2, 67.0, 55.3, 51.0, 41.3, 17.5.

#### 4.9.43 15-((2-(2-azidoethoxy)ethoxy)methyl)-15-methyl-2,5,7,10,13,17-hexaoxanonadecan-19-al (51)

Follow General Procedure B, using alkene **46**. Yield: 97 mg (90%); ^1^H NMR (400 MHz, CDCl_3_): δ = 9.74 (s, 1H), 4.75 (s, 2H), 4.02 (s, 2H), 3.72–3.31 (m, 29H), 0.98 ppm (s, 3H); ^13^C NMR (126 MHz, CDCl_3_): δ = 202.1, 95.8, 74.7, 74.0, 73.9, 72.0, 71.25, 71.22, 70.8, 70.7, 70.6, 70.2, 67.1, 67.0, 59.1, 51.0, 41.3, 17.5.

#### 4.9.44 18-(2,5,7,10,13,16-Hexaoxaheptadecan-17-yl)-18-methyl-2,5,7,10,13,16,20-heptaoxadocosan-22-al (**52**)

Follow General Procedure B, using alkene **47**. Yield: 170 mg (87%); ^1^H NMR (500 MHz, CDCl_3_): δ = 9.73 (t, *J* = 1.0 Hz, 1H), 4.75 (s, 4H), 4.02 (d, *J* = 1.1 Hz, 2H), 3.73–3.55 (m, 32H), 3.43–3.31 (m, 12H), 0.97 ppm (s, 3H); ^13^C NMR (126 MHz, CDCl_3_): δ = 202.1, 95.8, 77.1, 74.7, 73.9, 72.0, 71.2, 70.82, 70.80, 70.7, 70.6, 67.1, 67.0, 59.1, 41.3, 17.5.

#### 4.9.45 15-((2-(2-(2-azidoethoxy)ethoxy)ethoxy)methyl)-15-methyl-2,4,7,10,13,17-hexaoxanonadecan-19-oic Acid (**53**)

Follow General Procedure C, using aldehyde **48**. Yield: 199 mg (98%); ^1^H NMR (500 MHz, CDCl_3_): δ = 4.63 (s, 2H), 4.02 (s, 2H), 3.68–3.52 (m, 22H), 3.43–3.33 (m, 11H), 0.92 ppm (s, 3H); ^13^C NMR (126 MHz, CDCl_3_): δ = 172.3, 96.6, 75.2, 74.60, 74.55, 71.3, 71.2, 70.8, 70.7, 70.65, 70.58, 70.42, 70.36, 70.1, 66.9, 55.3, 50.8, 40.8, 17.8.

#### 4.9.46 15-((2-(2-(2-azidoethoxy)ethoxy)ethoxy)methyl)-15-methyl-2,5,7,10,13,17-hexaoxanonadecan-19-oic Acid (**54**)

Follow General Procedure C, using aldehyde **49**. Yield: 64 mg (99%); ^1^H NMR (500 MHz, CDCl_3_): δ = 4.72 (s, 2H), 3.95 (s, 2H), 3.72–3.51 (m, 22H), 3.42–3.23 (m, 11H), 0.90 ppm (s, 3H); ^13^C NMR (126 MHz, CDCl_3_): δ = 173.7, 95.7, 75.3, 74.6, 71.9, 71.1, 70.8, 70.6, 70.5, 70.4, 70.3, 70.1, 67.0, 66.91, 66.88, 59.0, 50.8, 40.9, 17.8.

#### 4.9.47 15-((2-(2-azidoethoxy)ethoxy)methyl)-15-methyl-2,4,7,10,13,17-hexaoxanonadecan-19-oic Acid (**55**)

Follow General Procedure C, using aldehyde **50**. Yield: 88 mg (95%); ^1^H NMR (400 MHz, CDCl_3_): δ = 4.64 (s, 2H), 4.03 (s, 2H), 3.71–3.50 (m, 18H), 3.43–3.27 (m, 11H), 0.94 ppm (s, 3H); ^13^C NMR (101 MHz, CDCl_3_): δ = 172.3, 96.6, 75.3, 74.8, 74.5, 71.3, 71.2, 70.70, 70.65, 70.6, 70.5, 70.4, 70.1, 68.8, 66.9, 55.3, 50.9, 40.8, 17.8.

#### 4.9.48 15-((2-(2-azidoethoxy)ethoxy)methyl)-15-methyl-2,5,7,10,13,17-hexaoxanonadecan-19-oic Acid (**56**)

Follow General Procedure C, using aldehyde **51**. Yield: 62 mg (63%); ^1^H NMR (400 MHz, CDCl_3_): δ = 4.73 (s, 2H), 4.01 (s, 2H), 3.72–3.51 (m, 18H), 3.42–3.30 (m, 11H), 0.93 ppm (s, 3H); ^13^C NMR (101 MHz, CDCl_3_): δ = 173.0, 95.7, 75.5, 74.9, 74.8, 71.9, 71.3, 71.2, 70.5, 70.5, 70.2, 70.1, 69.5, 67.0, 66.9, 59.1, 50.9, 40.8, 17.9.

#### 4.9.49 18-(2,5,7,10,13,16-Hexaoxaheptadecan-17-yl)-18-methyl-2,5,7,10,13,16,20-heptaoxadocosan-22-oic Acid (**57**)

Follow General Procedure C, using aldehyde **52**. Yield: 147 mg (91%); ^1^H NMR (500 MHz, CDCl_3_): δ = 4.75 (s, 4H), 4.05 (s, 2H), 3.74–3.55 (m, 32H), 3.44 (s, 2H), 3.42 (d, *J* = 9.0 Hz, 2H), 3.39 (s, 6H), 3.36 (d, *J* = 9.1 Hz, 2H), 0.95 ppm (s, 3H): ^13^C NMR (126 MHz, CDCl_3_): δ = 171.9, 95.7, 75.2, 74.7, 71.9, 71.3, 70.8, 70.7, 70.6, 70.4, 68.8, 67.0, 66.9, 59.1, 40.7, 18.0.

#### 4.9.50 (2*S*,4*R*)-1-((21*S*)-15-((2-(2-(2-azidoethoxy)ethoxy)ethoxy)methyl)-21-(*tert*-Butyl)-15-methyl-19-oxo-2,4,7,10,13,17-hexaoxa-20-azadocosan-22-oyl)-4-hydroxy-*N*-(4-(4-methylthiazol-5-yl)benzyl)pyrrolidine-2-carboxamide (**59**)

Follow General Procedure D, using carboxylic acid **53** and VH032-amine (**14**, synthesized through literature procedures^21,22^). Yield: 64 mg (59%); Contains a mixture of two diastereomers; ^1^H NMR (500 MHz, CDCl_3_): δ = 8.65 (s, 1H), 7.39 (t, *J* = 6.0 Hz, 1H), 7.34 (d, *J* = 8.4 Hz, 2H), 7.31 (d, *J* = 8.5 Hz, 2H), 7.13 (d, *J* = 8.9 Hz, 1H), 4.69 (t, *J* = 7.8 Hz, 1H), 4.62 (s, 2H), 4.56–4.47 (m, 3H), 4.32 (dd, *J* = 5.3, 15.0 Hz, 1H), 4.01 (d, *J* = 11.1 Hz, 1H), 3.92 (d, *J* = 15.9 Hz, 1H), 3.88 (d, *J* = 15.8 Hz, 1H), 3.68–3.50 (m, 23H), 3.41 (d, *J* = 9.0 Hz, 1H), 3.38 (d, *J* = 9.6 Hz, 1H), 3.31 (t, *J* = 18.7 Hz, 9H), 2.51–2.45 (m, 4H), 2.33 ppm (br s, 1H), 2.09 (dd, *J* = 7.9, 13.6 Hz, 1H), 0.95 (s, 3H), 0.93 ppm (s, 9H); ^13^C NMR (126 MHz, CDCl_3_): δ = 171.3, 170.9, 170.4, 150.3, 148.5, 138.3, 131.7, 131.0, 129.5, 128.2, 96.6, 74.6, 73.9, 73.8, 71.1, 71.1, 70.8, 70.71, 70.69, 70.66, 70.6, 70.54, 70.45, 70.1, 66.9, 58.6, 56.9, 56.7, 55.2, 50.8, 43.3, 41.1, 36.1, 35.2, 26.5, 17.5, 16.1; LC–MS *m*/*z* calc. for C_43_H_70_N_7_O_13_S [M + H]^+^ 924.5, found: 924.8.

#### 4.9.51 (2*S*,4*R*)-1-((21*S*)-15-((2-(2-(2-azidoethoxy)ethoxy)ethoxy)methyl)-21-(*tert*-Butyl)-15-methyl-19-oxo-2,4,7,10,13,17-hexaoxa-20-azadocosan-22-oyl)-4-hydroxy-*N*-((*S*)-1-(4-(4-methylthiazol-5-yl)phenyl)ethyl)pyrrolidine-2-carboxamide (**60**)

Follow General Procedure D, using carboxylic acid **53** and Me-VH032-amine (**58**, synthesized through literature procedure^38^). Yield: 89 mg (66%); Contains a mixture of two diastereomers; ^1^H NMR (500 MHz, CDCl_3_): δ = 8.66 (s, 1H), 7.46 (d, *J* = 7.8 Hz, 1H), 7.38 (d, *J* = 8.3 Hz, 2H), 7.34 (d, *J* = 8.2 Hz, 2H), 7.17 (d, *J* = 8.5 Hz, 1H), 5.05 (dq, *J* = 7.1, 7.2 Hz, 1H), 4.71 (t, *J* = 7.7 Hz, 1H), 4.63 (s, 2H), 4.52 (d, *J* = 8.8 Hz, 1H), 4.48 (br s, 1H), 4.03 (d, *J* = 11.9 Hz, 1H), 3.92 (s, 2H), 3.68–3.51 (m, 23H), 3.43 (d, *J* = 9.0 Hz, 1H), 3.39 (d, *J* = 8.9 Hz, 1H), 3.38–3.27 (m, 9H), 2.53–2.45 (m, 4H), 2.35 (br s, 1H), 2.03 (dd, *J* = 8.3, 13.1 Hz, 1H), 1.45 (d, *J* = 7.0 Hz, 3H), 1.04 (s, 9H), 0.97 ppm (s, 3H); ^13^C NMR (101 MHz, CDCl_3_): δ = 171.6, 170.6, 169.8, 150.4, 148.6, 143.3, 131.7, 131.0, 129.7, 126.5, 96.7, 74.7, 73.94, 73.87, 71.2, 71.1, 70.9, 70.8, 70.7, 70.65, 70.60, 70.5, 70.1, 66.9, 58.4, 57.1, 56.6, 55.3, 50.8, 49.0, 41.1, 35.5, 35.1, 26.6, 22.3, 17.6, 16.2; LC–MS *m*/*z* calc. for C_44_H_72_N_7_O_13_S [M + H]^+^ 938.5, found: 938.8.

#### 4.9.52 (2*S*,4*R*)-1-((21*S*)-15-((2-(2-(2-azidoethoxy)ethoxy)ethoxy)methyl)-21-(*tert*-Butyl)-15-methyl-19-oxo-2,5,7,10,13,17-hexaoxa-20-azadocosan-22-oyl)-4-hydroxy-*N*-(4-(4-methylthiazol-5-yl)benzyl)pyrrolidine-2-carboxamide (**61**)

Follow General Procedure D, using carboxylic acid **54** and VH032-amine (**14**, synthesized through literature procedures^21,22^). Yield: 58 mg (50%); Contains a mixture of two diastereomers; ^1^H NMR (500 MHz, CDCl_3_): δ = 8.65 (s, 1H), 7.38 (t, *J* = 5.9 Hz, 1H), 7.33 (d, *J* = 8.5 Hz, 2H), 7.32 (d, *J* = 8.6 Hz, 2H), 7.13 (d, *J* = 9.0 Hz, 1H), 4.71–4.67 (m, 3H), 4.55–4.46 (m, 3H), 4.32 (dd, *J* = 5.3, 15.0 Hz, 1H), 4.01 (d, *J* = 11.3 Hz, 1H), 3.90 (s, 2H), 3.68–3.51 (m, 23H), 3.40 (d, *J* = 8.8 Hz, 1H), 3.38 (d, *J* = 9.0 Hz, 1H), 3.32 (d, *J* = 34.6 Hz, 9H), 2.50–2.44 (m, 4H), 2.30 (br s, 1H), 2.08 (dd, *J* = 7.7, 13.5 Hz, 1H), 0.95 (s, 3H), 0.92 ppm (s, 9H); ^13^C NMR (126 MHz, CDCl_3_): δ = 171.3, 171.0, 170.4, 150.3, 148.5, 138.3, 131.7, 131.0, 129.5, 128.1, 95.7, 74.6, 73.9, 73.8, 71.8, 71.12, 71.09, 70.8, 70.7, 70.5, 70.4, 70.1, 67.0, 66.9, 59.0, 58.6, 56.9, 56.7, 50.8, 43.3, 41.1, 36.1, 35.2, 26.4, 17.5, 16.1; LC–MS *m*/*z* calc. for C_43_H_70_N_7_O_13_S [M + H]^+^ 924.5, found: 924.8.

#### 4.9.53 (2*S*,4*R*)-1-((21*S*)-15-((2-(2-azidoethoxy)ethoxy)methyl)-21-(*tert*-Butyl)-15-methyl-19-oxo-2,4,7,10,13,17-hexaoxa-20-azadocosan-22-oyl)-4-hydroxy-*N*-(4-(4-methylthiazol-5-yl)benzyl)pyrrolidine-2-carboxamide (**62**)

Follow General Procedure D, using carboxylic acid **55** and VH032-amine (**14**, synthesized through literature procedures^21,22^). Yield: 51 mg (61%); Contains a mixture of two diastereomers; ^1^H NMR (400 MHz, CDCl_3_): δ = 8.65 (s, 1H), 7.39 (t, *J* = 5.5 Hz, 1H), 7.36–7.28 (m, 4H), 7.15 (d, *J* = 8.6 Hz, 1H), 4.70 (t, *J* = 7.7 Hz, 1H), 4.62 (s, 2H), 4.57–4.46 (m, 3H), 4.32 (dd, *J* = 5.1, 15.1 Hz, 1H), 4.02 (d, *J* = 11.1 Hz, 1H), 3.90 (s, 2H), 3.68–3.47 (m, 19H), 3.42 (d, *J* = 9.3 Hz, 1H), 3.38 (d, *J* = 9.2 Hz, 1H), 3.36–3.26 (m, 9H), 2.50–2.42 (m, 4H), 2.13–2.03 (m, 1H), 0.96 (s, 3H), 0.94 ppm (s, 9H); ^13^C NMR (101 MHz, CDCl_3_): δ = 171.3, 171.0, 170.4, 150.4, 148.5, 138.3, 131.7, 131.0, 129.5, 128.1, 96.6, 74.6, 74.0, 73.9, 73.84, 73.78, 71.15, 71.08, 70.8, 70.69, 70.66, 70.6, 70.5, 70.1, 66.9, 58.7, 56.9, 56.7, 55.2, 50.8, 43.3, 41.1, 36.1, 35.2, 26.5, 17.5, 16.1; LC–MS *m*/*z* calc. for C_41_H_64_N_7_O_12_S [M-H]^−^ 878.4, found: 878.2.

#### 4.9.54 (2*S*,4*R*)-1-((21*S*)-15-((2-(2-azidoethoxy)ethoxy)methyl)-21-(*tert*-Butyl)-15-methyl-19-oxo-2,5,7,10,13,17-hexaoxa-20-azadocosan-22-oyl)-4-hydroxy-*N*-(4-(4-methylthiazol-5-yl)benzyl)pyrrolidine-2-carboxamide (**63**)

Follow General Procedure D, using carboxylic acid **56** and VH032-amine (**14**, synthesized through literature procedures^21,22^). Yield: 45 mg (78%); Contains a mixture of two diastereomers; ^1^H NMR (400 MHz, CDCl_3_): δ = 8.66 (s, 1H), 7.40–7.29 (m, 5H), 7.13 (d, *J* = 8.7 Hz, 1H), 4.75–4.66 (m, 3H), 4.58–4.45 (m, 3H), 4.32 (dd, *J* = 5.3, 15.0 Hz, 1H), 4.05 (d, *J* = 10.8 Hz, 1H), 3.92 (s, 2H), 3.71–3.49 (m, 19H), 3.45–3.27 (m, 11H), 2.56–2.45 (m, 4H), 2.14–2.04 ppm (m, 1H); ^13^C NMR (101 MHz, CDCl_3_): δ = 171.4, 170.9, 170.5, 150.4, 148.6, 138.3, 131.7, 131.0, 129.6, 128.2, 95.7, 74.6, 74.0, 73.93, 73.91, 73.8, 71.9, 71.2, 71.1, 70.8, 70.63, 70.57, 70.5, 70.2, 67.1, 66.9, 59.1, 58.6, 57.0, 56.7, 50.9, 43.3, 41.1, 36.0, 35.1, 26.5, 17.6, 16.1; LC–MS *m*/*z* calc. for C_41_H_66_N_7_O_12_S [M + H]^+^ 880.4, found: 880.7.

#### 4.9.55 (2*S*,4*R*)-1-((*S*)-24-(*tert*-Butyl)-18-(2,5,7,10,13,16-hexaoxaheptadecan-17-yl)-18-methyl-22-oxo-2,5,7,10,13,16,20-heptaoxa-23-azapentacosan-25-oyl)-4-hydroxy-*N*-(4-(4-methylthiazol-5-yl)benzyl)pyrrolidine-2-carboxamide (**64**)

Follow General Procedure D, using carboxylic acid **57** and VH032-amine (**14**, synthesized through literature procedures^21,22^). Yield: 179 mg (73%); ^1^H NMR (400 MHz, CDCl_3_): δ = 8.67 (s, 1H), 7.40–7.29 (m, 5H), 7.13 (d, *J* = 8.9 Hz, 1H), 4.73–4.67 (m, 5H), 4.58–4.46 (m, 3H), 4.32 (dd, *J* = 5.3, 15.0 Hz, 1H), 4.03 (d, *J* = 11.3 Hz, 1H), 3.93 (d, *J* = 15.4 Hz, 1H), 3.87 (d, *J* = 15.5 Hz, 1H), 3.71–3.49 (m, 33H), 3.43–3.26 (m, 12H), 2.55–2.45 (m, 4H), 2.09 (dd, *J* = 8.0, 13.2 Hz, 1H), 0.95 (s, 3H), 0.94 ppm (s, 9H); ^13^C NMR (101 MHz, CDCl_3_): δ = 171.4, 170.9, 170.4, 150.4, 148.5, 138.3, 131.7, 131.0, 129.6, 128.2, 95.7, 74.6, 73.9, 73.8, 71.9, 71.1, 70.8, 70.71, 70.68, 70.6, 70.5, 70.2, 67.0, 66.9, 59.1, 58.6, 57.0, 56.7, 43.3, 41.1, 36.1, 35.2, 26.5, 17.6, 16.1.

#### 4.9.56 (2*S*,4*R*)-1-((21*S*)-21-(*tert*-Butyl)-15-((2-(2-(2-((2-(2,6-dioxopiperidin-3-yl)-1,3-dioxoisoindolin-4-yl)amino)ethoxy)ethoxy)ethoxy)methyl)-15-methyl-19-oxo-2,4,7,10,13,17-hexaoxa-20-azadocosan-22-oyl)-4-hydroxy-*N*-(4-(4-methylthiazol-5-yl)benzyl)pyrrolidine-2-carboxamide (**66**)

Follow General Procedure H, using azide **59** and 2-(2,6-dioxopiperidin-3-yl)-4-fluoroisoindoline-1,3-dione (**19**). Yield: 6 mg (47%); Contains a mixture of four diastereomers; ^1^H NMR (500 MHz, CDCl_3_): δ = 9.34–9.09 (m, 1H), 8.68 (s, 1H), 7.51–7.29 (m, 6H), 7.17–7.12 (m, 1H), 7.09 (d, *J* = 7.3 Hz, 1H), 6.91 (dd, *J* = 6.5, 8.1 Hz, 1H), 6.53–6.49 (m, 1H), 4.95–4.81 (m, 1H), 4.74–4.69 (m, 1H), 4.64 (s, 2H), 4.62–4.45 (m, 3H), 4.36–4.26 (m, 1H), 4.12 (d, *J* = 11.0 Hz, 1H), 3.97–3.89 (m, 2H), 3.78–3.50 (m, 23H), 3.48–3.25 (m, 11H), 2.88–2.64 (m, 3H), 2.56–2.49 (m, 4H), 2.14–2.05 (m, 2H), 0.97–0.93 ppm (m, 12H); LC–MS *m*/*z* calc. for C_56_H_80_N_7_O_17_S [M + H]^+^ 1154.5, found: 1155.1.

#### 4.9.57 (2*S*,4*R*)-1-((21*S*)-21-(*tert*-Butyl)-15-((2-(2-(2-((2-(2,6-dioxopiperidin-3-yl)-1,3-dioxoisoindolin-4-yl)amino)ethoxy)ethoxy)ethoxy)methyl)-15-methyl-19-oxo-2,4,7,10,13,17-hexaoxa-20-azadocosan-22-oyl)-4-hydroxy-*N*-((*S*)-1-(4-(4-methylthiazol-5-yl)phenyl)ethyl)pyrrolidine-2-carboxamide (**67**)

Follow General Procedure H, using azide **60** and 2-(2,6-dioxopiperidin-3-yl)-4-fluoroisoindoline-1,3-dione (**19**). Yield: 8 mg (43%); Contains a mixture of four diastereomers; ^1^H NMR (500 MHz, CDCl_3_): δ = 9.26–9.01 (m, 1H), 8.68 (s, 1H), 7.53–7.44 (m, 2H), 7.42–7.35 (m, 4H), 7.22–7.15 (m, 1H), 7.09 (d, *J* = 6.7 Hz, 1H), 6.90 (dd, *J* = 2.9, 8.6 Hz, 1H), 6.54–6.48 (m, 1H), 5.08 (dq, *J* = 7.1, 7.1 Hz, 1H), 4.94–4.87 (m, 1H), 4.75–4.71 (m, 1H), 4.65 (s, 2H), 4.57–4.47 (m, 2H), 4.13 (d, *J* = 11.7 Hz, 1H), 3.99–3.91 (m, 2H), 3.77–3.51 (m, 23H), 3.48–3.28 (m, 11H), 2.89–2.69 (m, 3H), 2.56–2.48 (m, 4H), 2.14–2.04 (m, 2H), 1.47 (d, *J* = 6.7 Hz, 3H), 1.05 (s, 9H), 0.98–0.95 ppm (m, 3H); ^13^C NMR (126 MHz, CDCl_3_): δ = 171.9, 171.7, 171.6, 170.90, 170.88, 170.87, 170.8, 169.9, 169.8, 169.41, 169.40, 168.81, 168.79, 168.78, 167.81, 167.80, 150.4, 148.6, 147.0, 143.4, 136.2, 132.7, 131.8, 131.0, 129.7, 126.6, 116.9, 111.78, 111.76, 110.50, 110.49, 96.7, 74.5, 74.1, 73.9, 73.8, 71.32, 71.26, 71.2, 71.1, 71.01, 70.99, 70.97, 70.9, 70.82, 70.76, 70.73, 70.71, 70.65, 70.53, 70.52, 70.3, 69.59, 69.57, 69.5, 66.9, 58.5, 57.2, 57.1, 56.8, 55.3, 49.07, 49.05, 49.0, 42.5, 41.1, 35.7, 35.6, 35.1, 35.1, 35.0, 31.6, 29.8, 26.6, 22.9, 22.4, 22.3, 17.6, 16.2; LC–MS *m*/*z* calc. for C_57_H_82_N_7_O_17_S [M + H]^+^ 1168.5, found: 1169.2.

#### 4.9.58 (2*S*,4*R*)-1-((21*S*)-21-(*tert*-Butyl)-15-((2-(2-(2-((2-(2,6-dioxopiperidin-3-yl)-1,3-dioxoisoindolin-4-yl)amino)ethoxy)ethoxy)ethoxy)methyl)-15-methyl-19-oxo-2,5,7,10,13,17-hexaoxa-20-azadocosan-22-oyl)-4-hydroxy-*N*-(4-(4-methylthiazol-5-yl)benzyl)pyrrolidine-2-carboxamide (**68**)

Follow General Procedure H, using azide **61** and 2-(2,6-dioxopiperidin-3-yl)-4-fluoroisoindoline-1,3-dione (**19**). Yield: 5 mg (32%); Contains a mixture of four diastereomers; ^1^H NMR (500 MHz, CDCl_3_): δ = 9.33–9.08 (m, 1H), 8.68 (s, 1H), 7.51–7.40 (m, 2H), 7.39–7.33 (m, 4H), 7.18–7.12 (m, 1H), 7.10 (d, *J* = 7.0 Hz, 1H), 6.93–6.88 (m, 1H), 6.54–6.48 (m, 1H), 4.95–4.81 (m, 1H), 4.74–4.70 (m, 3H), 4.64–4.45 (m, 3H), 4.36–4.27 (m, 1H), 4.12 (d, *J* = 10.8 Hz, 1H), 3.98–3.88 (m, 2H), 3.78–3.49 (m, 23H), 3.48–3.26 (m, 11H), 2.86–2.65 (m, 3H), 2.57–2.49 (m, 4H), 2.14–2.05 (m, 2H), 0.97–0.91 ppm (m, 12H); ^13^C NMR (126 MHz, CDCl_3_): δ = 171.83, 171.79, 171.5, 171.4, 171.0, 170.9, 170.8, 169.4, 169.4, 168.8, 167.8, 150.4, 148.6, 148.6, 147.0, 146.9, 138.5, 138.40, 138.38, 136.1, 132.7, 131.8, 131.0, 129.59, 129.56, 128.34, 128.28, 116.9, 116.8, 111.8, 111.7, 110.5, 110.5, 95.7, 74.4, 74.0, 73.9, 73.8, 71.9, 71.3, 71.23, 71.15, 71.1, 71.02, 70.96, 70.89, 70.86, 70.81, 70.77, 70.7, 70.59, 70.57, 70.5, 70.39, 70.36, 69.6, 69.5, 67.1, 66.9, 59.1, 58.7, 58.6, 57.1, 57.0, 56.9, 56.8, 49.3, 49.05, 49.00, 48.97, 43.4, 42.6, 42.5, 41.1, 36.2, 36.1, 35.1, 35.0, 31.6, 26.5, 22.9, 17.6, 16.2; LC–MS *m*/*z* calc. for C_56_H_80_N_7_O_17_S [M + H]^+^ 1154.5, found: 1155.2.

#### 4.9.59 (2*S*,4*R*)-1-((21*S*)-21-(*tert*-Butyl)-15-((2-(2-(2-((2-(2,6-dioxopiperidin-3-yl)-6-fluoro-1,3-dioxoisoindolin-5-yl)amino)ethoxy)ethoxy)ethoxy)methyl)-15-methyl-19-oxo-2,4,7,10,13,17-hexaoxa-20-azadocosan-22-oyl)-4-hydroxy-*N*-(4-(4-methylthiazol-5-yl)benzyl)pyrrolidine-2-carboxamide (**69**)

Follow General Procedure H, using azide **59** and 2-(2,6-dioxopiperidin-3-yl)-5,6-difluoroisoindoline-1,3-dione (**65**). Yield: 8 mg (62%); Contains a mixture of four diastereomers; ^1^H NMR (500 MHz, CDCl_3_): δ = 8.68 (s, 1H), 8.58 (d, *J* = 14.0 Hz, 1H), 7.43–7.34 (m, 6H), 7.13–7.12 (m, 2H), 5.22–5.20 (m, 1H), 4.90 (dd, *J* = 4.8, 12.0 Hz, 1H), 4.72 (t, *J* = 7.9 Hz, 1H), 4.64 (s, 2H), 4.58 (dd, *J* = 6.6, 14.9 Hz, 1H), 4.54 (br s, 1H), 4.47 (d, *J* = 8.1 Hz, 1H), 4.34 (dd, *J* = 4.3, 14.7 Hz, 1H), 4.11 (d, *J* = 11.3 Hz, 1H), 3.95–3.87 (m, 2H), 3.75 (t, *J* = 5.0 Hz, 2H), 3.70–3.50 (m, 21H), 3.48–3.42 (m, 2H), 3.42–3.26 (m, 9H), 2.88–2.67 (m, 3H), 2.59–2.51 (m, 4H), 2.16–2.10 (m, 2H), 0.96–0.90 ppm (m, 12H); ^13^C NMR (126 MHz, CDCl_3_): δ = 171.53, 171.50, 171.48, 171.40, 171.38, 170.83, 170.82, 170.73, 170.71, 168.49, 168.47, 167.6, 167.05, 167.03, 153.98 (d, *J*_C–F_ = 248.6 Hz), 150.4, 148.6, 143.2, 142.87 (d, *J*_C–F_ = 12.6 Hz), 138.3, 131.7, 131.0, 130.18, 130.16, 129.61, 129.60, 128.3, 118.72 (d, *J*_C–F_ = 7.4 Hz), 110.31 (d, *J*_C–F_ = 22.5 Hz), 105.99 (d, *J*_C–F_ = 4.8 Hz), 96.7, 74.50, 74.48, 74.4, 73.9, 73.8, 71.2, 71.12, 71.10, 70.9, 70.8, 70.75, 70.71, 70.66, 70.6, 70.5, 70.33, 70.32, 69.2, 69.13, 69.10, 66.9, 58.6, 58.5, 57.11, 57.08, 56.8, 55.3, 49.4, 43.4, 43.1, 41.1, 36.03, 36.00, 34.9, 31.6, 26.5, 22.9, 17.6, 16.2; ^19^F NMR (471 MHz, CDCl_3_): δ = −127.33–127.44 (m, 1F); LC–MS *m*/*z* calc. for C_56_H_79_FN_7_O_17_S [M + H]^+^ 1172.5, found: 1173.1.

#### 4.9.60 (2*S*,4*R*)-1-((21*S*)-21-(*tert*-Butyl)-15-((2-(2-(2-((2-(2,6-dioxopiperidin-3-yl)-6-fluoro-1,3-dioxoisoindolin-5-yl)amino)ethoxy)ethoxy)ethoxy)methyl)-15-methyl-19-oxo-2,4,7,10,13,17-hexaoxa-20-azadocosan-22-oyl)-4-hydroxy-*N*-((*S*)-1-(4-(4-methylthiazol-5-yl)phenyl)ethyl)pyrrolidine-2-carboxamide (**70**)

Follow General Procedure H, using azide (**60**) and 2-(2,6-dioxopiperidin-3-yl)-5,6-difluoroisoindoline-1,3-dione (**65**). Yield: 9 mg (47%); Contains a mixture of four diastereomers; ^1^H NMR (500 MHz, CDCl_3_): δ = 8.67 (s, 1H), 8.62–8.55 (m, 1H), 7.46 (d, *J* = 7.3 Hz, 1H), 7.42–7.39 (m, 3H), 7.36 (d, *J* = 8.3 Hz, 2H), 7.17 (d, *J*_H–F_ = 8.7 Hz, 1H), 7.13 (d, *J* = 6.9 Hz, 1H), 5.25–5.20 (m, 1H), 5.08 (dq, *J* = 7.0, 7.1 Hz, 1H), 4.90 (dd, *J* = 5.3, 12.4 Hz, 1H), 4.73 (t, *J* = 7.4 Hz, 1H), 4.65 (s, 2H), 4.53–4.49 (m, 2H), 4.12 (d, *J* = 11.2 Hz, 1H), 3.93 (s, 2H), 3.75 (t, *J* = 4.9 Hz, 2H), 3.70–3.55 (m, 21H), 3.48–3.29 (m, 11H), 2.99 (br s, 1H), 2.91–2.69 (m, 3H), 2.58–2.53 (m, 4H), 2.16–2.09 (m, 1H), 2.09–2.04 (m, 1H), 1.49–1.45 (m, 3H), 1.05 (s, 9H), 0.97–0.96 ppm (m, 3H); ^13^C NMR (126 MHz, CDCl_3_): δ = 171.71, 171.68, 171.3, 170.8, 169.8, 168.50, 168.45, 167.6, 167.04, 167.01, 153.98 (d, *J*_C–F_ = 248.7 Hz), 150.4, 148.6, 143.3, 142.86 (d, *J*_C–F_ = 12.7 Hz), 131.7, 131.0, 130.19 (d, *J*_C–F_ = 1.9 Hz), 129.7, 126.6, 118.73 (d, *J*_C–F_ = 8.8 Hz), 110.29 (d, *J*_C–F_ = 22.3 Hz), 105.96 (d, *J*_C–F_ = 5.2 Hz), 105.97, 96.7, 74.5, 74.0, 73.9, 73.8, 71.2, 71.1, 70.9, 70.78, 70.75, 70.73, 70.66, 70.6, 70.5, 70.3, 69.14, 69.09, 66.9, 58.5, 58.4, 57.2, 57.1, 56.7, 55.3, 49.4, 49.0, 43.1, 41.1, 35.5, 35.0, 31.6, 26.6, 22.9, 22.4, 17.6, 16.2; ^19^F NMR (471 MHz, CDCl_3_): δ = −127.34–127.44 (m, 1F); LC–MS *m*/*z* calc. for C_57_H_81_FN_7_O_17_S [M + H]^+^ 1186.5, found: 1187.2.

#### 4.9.61 (2*S*,4*R*)-1-((21*S*)-21-(*tert*-Butyl)-15-((2-(2-(2-((2-(2,6-dioxopiperidin-3-yl)-6-fluoro-1,3-dioxoisoindolin-5-yl)amino)ethoxy)ethoxy)ethoxy)methyl)-15-methyl-19-oxo-2,5,7,10,13,17-hexaoxa-20-azadocosan-22-oyl)-4-hydroxy-*N*-(4-(4-methylthiazol-5-yl)benzyl)pyrrolidine-2-carboxamide (**71**)

Follow General Procedure H, using azide **61** and 2-(2,6-dioxopiperidin-3-yl)-5,6-difluoroisoindoline-1,3-dione (**65**). Yield: 9 mg (57%); Contains a mixture of four diastereomers; ^1^H NMR (500 MHz, CDCl_3_): δ = 8.68 (s, 1H), 8.65–8.60 (m, 1H), 7.43–7.33 (m, 6H), 7.13–711 (m, 2H), 5.24–5.18 (m, 1H), 4.89 (dd, *J* = 4.5, 12.1 Hz, 1H), 4.74–4.71 (m, 3H), 4.58 (dd, *J* = 6.6, 14.8 Hz, 1H), 4.55–4.51 (m, 1H), 4.47 (d, *J* = 8.6 Hz, 1H), 4.34 (dd, *J* = 4.6, 15.5 Hz, 1H), 4.10 (d, *J* = 11.3 Hz, 1H), 3.93 (d, *J* = 17.1 Hz, 1H), 3.89 (d, *J* = 16.5 Hz, 1H), 3.77–3.72 (m, 2H), 3.71–3.50 (m, 21H), 3.48–3.42 (m, 2H), 3.42–3.34 (m, 5H), 3.31–3.26 (m, 4H), 2.90–2.67 (m, 3H), 2.58–2.51 (m, 4H), 2.16–2.10 (m, 2H), 0.96–0.91 ppm (m, 12H); ^13^C NMR (126 MHz, CDCl_3_): δ = 171.5, 171.43, 171.41, 170.9, 170.70, 170.69, 168.52, 168.50, 167.05, 167.03, 153.98 (d, *J*_C–F_ = 249.2 Hz), 150.4, 148.6, 142.85 (d, *J*_C–F_ = 11.6 Hz), 138.35, 138.34, 131.7, 131.0, 130.17 (d, *J*_C–F_ = 2.3 Hz), 129.60, 129.59, 128.3, 118.71 (d, *J*_C–F_ = 9.3 Hz), 110.29 (d, *J*_C–F_ = 22.4 Hz), 105.99 (d, *J*_C–F_ = 4.5 Hz), 95.7, 74.5, 73.91, 73.85, 73.8, 71.9, 71.2, 71.1, 70.9, 70.79, 70.78, 70.77, 70.74, 70.73, 70.69, 70.60, 70.59, 70.5, 70.32, 70.31, 69.2, 69.12, 69.08, 67.1, 66.9, 59.1, 58.6, 58.5, 57.10, 57.08, 57.06, 57.0, 56.8, 49.4, 43.4, 43.1, 41.1, 36.0, 35.0, 31.6, 26.5, 22.9, 17.6, 16.2; ^19^F NMR (471 MHz, CDCl_3_): δ = −127.32–127.44 (m, 1F); LC–MS *m*/*z* calc. for C_56_H_79_FN_7_O_17_S [M + H]^+^ 1172.5, found: 1173.1.

#### 4.9.62 (2*S*,4*R*)-1-((21*S*)-21-(*tert*-Butyl)-15-((2-(2-((2-(2,6-dioxopiperidin-3-yl)-6-fluoro-1,3-dioxoisoindolin-5-yl)amino)ethoxy)ethoxy)methyl)-15-methyl-19-oxo-2,4,7,10,13,17-hexaoxa-20-azadocosan-22-oyl)-4-hydroxy-*N*-(4-(4-methylthiazol-5-yl)benzyl)pyrrolidine-2-carboxamide (**72**)

Follow General Procedure H, using azide **62** and 2-(2,6-dioxopiperidin-3-yl)-5,6-difluoroisoindoline-1,3-dione (**65**). Yield: 9 mg (47%); Contains a mixture of four diastereomers; ^1^H NMR (500 MHz, CDCl_3_): δ = 8.97–8.92 (m, 1H), 8.67 (s, 1H), 7.47–7.42 (m, 1H), 7.41–7.39 (m, 1H), 7.36–7.31 (m, 4H), 7.15 (d, *J* = 8.7 Hz, 1H), 7.12–7.09 (m, 1H), 5.22–5.19 (m, 1H), 4.91–4.86 (m, 1H), 4.69 (t, *J* = 8.1 Hz, 1H), 4.63 (s, 2H), 4.59–4.48 (m, 3H), 4.35–4.30 (m, 1H), 4.07 (d, *J* = 11.3 Hz, 1H), 3.94–3.85 (m, 2H), 3.71 (t, *J* = 4.8 Hz, 2H), 3.68–3.50 (m, 17H), 3.44 (d, *J* = 5.2 Hz, 1H), 3.42 (d, *J* = 5.6 Hz, 1H), 3.39–3.25 (m, 9H), 2.87–2.66 (m, 3H), 2.50–2.45 (m, 4H), 2.14–2.08 (m, 2H), 0.95 (s, 9H), 0.92–0.92 ppm (m, 3H); ^13^C NMR (126 MHz, CDCl_3_): δ = 171.60, 171.56, 171.55, 171.34, 171.31, 171.01, 170.99, 170.98, 170.6, 168.7, 167.58, 167.56, 167.09, 167.06, 153.92 (d, *J*_C–F_ = 249.5 Hz), 150.4, 148.6, 142.79 (d, *J*_C–F_ = 12.7 Hz), 138.4, 138.4, 131.74, 131.73, 130.96, 130.95, 130.1, 129.5, 129.5, 128.2, 118.65 (d, *J*_C–F_ = 8.2 Hz), 110.34 (d, *J*_C–F_ = 22.3 Hz), 106.04–105.91 (m), 96.6, 74.52, 74.48, 74.4, 73.8, 73.74, 73.69, 73.67, 71.3, 71.21, 71.16, 71.09, 71.06, 70.89, 70.87, 70.71, 70.68, 70.68, 70.6, 70.52, 70.48, 70.46, 70.3, 69.1, 69.02, 69.00, 66.9, 58.73, 58.71, 58.69, 58.67, 57.02, 57.00, 56.97, 56.8, 55.3, 49.4, 43.3, 43.1, 41.11, 41.09, 41.07, 36.2, 36.2, 36.1, 35.13, 35.11, 35.1, 31.6, 26.5, 22.8, 17.53, 17.51, 17.50, 16.2; ^19^F{^1^H} NMR (471 MHz, CDCl_3_): δ = −127.39–127.44 (m, 1F); LC–MS *m*/*z* calc. for C_54_H_75_FN_7_O_16_S [M + H]^+^ 1128.5, found: 1128.4.

#### 4.9.63 (2*S*,4*R*)-1-((21*S*)-21-(*tert*-Butyl)-15-((2-(2-((2-(2,6-dioxopiperidin-3-yl)-6-fluoro-1,3-dioxoisoindolin-5-yl)amino)ethoxy)ethoxy)methyl)-15-methyl-19-oxo-2,5,7,10,13,17-hexaoxa-20-azadocosan-22-oyl)-4-hydroxy-*N*-(4-(4-methylthiazol-5-yl)benzyl)pyrrolidine-2-carboxamide (**73**)

Follow General Procedure H, using azide **63** and 2-(2,6-dioxopiperidin-3-yl)-5,6-difluoroisoindoline-1,3-dione (**65**). Yield: 6 mg (32%); Contains a mixture of four diastereomers; ^1^H NMR (500 MHz, CDCl_3_): δ = 8.87–8.78 (m, 1H), 8.67 (s, 1H), 7.48–7.38 (m, 2H), 7.38–7.32 (m, 4H), 7.17–7.09 (m, 2H), 5.23–5.17 (m, 1H), 4.92–4.87 (m, 1H), 4.73 (s, 2H), 4.70 (t, *J* = 8.0 Hz, 1H), 4.61–4.47 (m, 3H), 4.37–4.31 (m, 1H), 4.10 (d, *J* = 9.8 Hz, 1H), 3.94–3.86 (m, 2H), 3.74–3.50 (m, 19H), 3.48–3.27 (m, 11H), 2.89–2.64 (m, 3H), 2.55–2.46 (m, 4H), 2.16–2.07 (m, 2H), 0.95 (s, 9H), 0.93–0.91 ppm (m, 3H); ^13^C NMR (126 MHz, CDCl_3_): δ = 171.53, 171.48, 171.40, 171.39, 171.36, 171.0, 170.95, 170.93, 170.92, 170.7, 168.60, 168.59, 167.59, 167.58, 167.56, 167.56, 167.1, 153.93 (d, *J*_C–F_ = 248.2 Hz), 150.4, 148.6, 142.80 (d, *J*_C–F_ = 13.1 Hz), 138.39, 138.38, 138.37, 138.36, 131.75, 131.73, 130.99, 130.98, 130.13 (d, *J*_C–F_ = 1.8 Hz), 129.57, 129.56, 129.56, 129.5, 128.2, 118.66 (d, *J*_C–F_ = 8.9 Hz), 110.34 (d, *J*_C–F_ = 23.0 Hz), 106.08–105.98 (m), 95.7, 74.54, 74.49, 74.4, 73.9, 73.8, 73.7, 73.7, 71.9, 71.12, 71.11, 71.09, 70.91, 70.88, 70.57, 70.56, 70.5, 70.4, 70.31, 70.30, 70.28, 69.14, 69.12, 69.11, 69.04, 69.02, 69.01, 67.04, 67.04, 66.9, 59.1, 58.69, 58.67, 58.6, 58.6, 57.08, 57.06, 57.05, 57.0, 56.8, 56.80, 56.79, 56.78, 49.43, 49.42, 43.33, 43.32, 43.31, 43.11, 43.10, 43.09, 41.12, 41.10, 41.08, 35.07, 35.06, 35.04, 35.02, 31.6, 26.5, 22.8, 17.54, 17.52, 17.50, 16.2; ^19^F{^1^H} NMR (471 MHz, CDCl_3_): δ = −127.41–127.45 (m, 1F); LC–MS *m*/*z* calc. for C_54_H_75_FN_7_O_16_S [M + H]^+^ 1128.5, found: 1128.4.

#### 4.9.64 (17*S*)-17-((2*S*,4*R*)-4-hydroxy-2-((4-(4-methylthiazol-5-yl)benzyl)carbamoyl)pyrrolidine-1-carbonyl)-11-((2-(2-(2-hydroxyethoxy)ethoxy)ethoxy)methyl)-11,18,18-trimethyl-15-oxo-3,6,9,13-tetraoxa-16-azanonadecyl 2-((*S*)-4-(4-chlorophenyl)-2,3,9-trimethyl-6*H*-thieno[3,2-*f*][1,2,4]triazolo[4,3-*a*][1,4]diazepin-6-yl)acetate (**74**)

Follow General Procedure E, using 1.0 eq. of (+)-JQ1-acid (**22**) and 1.2 eq. of di-MEM protected compound **64**. Yield: 40 mg (40%); Contains a mixture of two diastereomers; ^1^H NMR (500 MHz, CDCl_3_): δ = 8.67 (s, 1H), 7.42–7.38 (m, 3H), 7.36 (s, 4H), 7.32 (d, *J* = 8.5 Hz, 2H), 7.19 (d, *J* = 8.6 Hz, 1H), 4.72 (t, *J* = 8.0 Hz, 1H), 4.62–4.50 (m, 4H), 4.40–4.26 (m, 3H), 4.08 (d, *J* = 11.1 Hz, 1H), 3.99 (d, *J* = 15.4 Hz, 1H), 3.92 (d, *J* = 15.4 Hz, 1H), 3.74 (t, *J* = 4.7 Hz, 2H), 3.72–3.53 (m, 23H), 3.46–3.40 (m, 2H), 3.39–3.28 (m, 4H), 2.66 (s, 3H), 2.53–2.46 (m, 4H), 2.41 (s, 3H), 2.16 (dd, *J* = 8.1, 13.3 Hz, 1H), 1.69 (s, 3H), 0.98 (s, 3H), 0.96 ppm (s, 9H); ^13^C NMR (126 MHz, CDCl_3_): δ = 171.7, 171.4, 171.1, 170.6, 164.0, 155.5, 150.4, 150.0, 148.6, 138.4, 136.9, 136.8, 132.4, 131.8, 131.0, 130.9, 130.6, 130.0, 129.6, 128.8, 128.3, 74.4, 74.1, 74.0, 73.8, 73.7, 72.8, 71.2, 71.10, 71.07, 71.0, 70.9, 70.7, 70.5, 70.2, 69.2, 64.2, 61.8, 58.7, 57.0, 56.9, 53.9, 43.4, 41.2, 36.9, 36.3, 35.3, 26.5, 17.7, 16.2, 14.5, 13.2, 11.9; LC–MS *m*/*z* calc. for C_60_H_83_ClN_8_O_14_S_2_ [M+2H]^2 +^ 619.3, found: 619.7.

#### 4.9.65 (17*S*)-17-((2*S*,4*R*)-4-hydroxy-2-((4-(4-methylthiazol-5-yl)benzyl)carbamoyl)pyrrolidine-1-carbonyl)-11,18,18-trimethyl-11-((2-(2-(2-((methylsulfonyl)oxy)ethoxy)ethoxy)ethoxy)methyl)-15-oxo-3,6,9,13-tetraoxa-16-azanonadecyl 2-((*S*)-4-(4-chlorophenyl)-2,3,9-trimethyl-6*H*-thieno[3,2-*f*][1,2,4]triazolo[4,3-*a*][1,4]diazepin-6-yl)acetate (**75**)

Alcohol **74** (40 mg, 32.3 μmol) was dissolved in DCM (380 μL) before the addition of DIPEA (16.9 μL, 96.9 μmol) and cooling to 0 °C. MsCl (2.5 μL, 32.3 μmol) was then added and the reaction was left to stir at 0 °C for 20 min before stirring at r.t. for 1 h. LC–MS showed 1:1 ratio of starting material to product, with no change after an additional 1 h of stirring. The flask was cooled to 0 °C and additional MsCl (1.3 μL, 16.2 μmol) was added. The flask was left to stir at 0 °C for 20 min before stirring at r.t. for 30 min. Careful addition of MsCl is required due to the formation of dimesyl product. The reaction was concentrated in vacuo and the residue purified by reverse phase flash column chromatography (50 g C18 gold column) using a linear gradient from 30% to 100% MeCN in 0.1% HCOOH in water over 11 min to afford compound **75** as a mixture of two diastereomers. Yield: 19 mg (44%); ^1^H NMR (500 MHz, CDCl_3_): δ = 8.67 (s, 1H), 7.41–7.36 (m, 3H), 7.35 (s, 4H), 7.32 (d, *J* = 8.6 Hz, 2H), 7.17–7.13 (m, 1H), 4.72 (t, *J* = 7.9 Hz, 1H), 4.62–4.50 (m, 4H), 4.40–4.26 (m, 5H), 4.06 (d, *J* = 11.2 Hz, 1H), 3.95 (d, *J* = 15.2 Hz, 1H), 3.90 (d, *J* = 15.3 Hz, 1H), 3.76–3.71 (m, 4H), 3.70–3.53 (m, 19H), 3.42 (d, *J* = 9.0 Hz, 1H), 3.40 (d, *J* = 9.3 Hz, 1H), 3.37–3.28 (m, 4H), 3.05 (s, 3H), 2.66 (s, 3H), 2.53–2.46 (m, 4H), 2.41 (s, 3H), 2.16 (dd, *J* = 8.6, 13.2 Hz, 1H), 1.68 (s, 3H), 0.99–0.96 (m, 3H), 0.95 ppm (s, 9H); ^13^C NMR (126 MHz, CDCl_3_): δ = 171.6, 171.4, 171.1, 170.4, 164.0, 155.5, 150.4, 150.0, 148.6, 138.4, 137.0, 136.7, 132.3, 131.8, 131.04, 130.99, 130.6, 130.0, 129.6, 128.8, 128.3, 74.6, 74.0, 73.9, 73.8, 71.2, 71.1, 70.93, 70.85, 70.72, 70.69, 70.53, 70.51, 70.2, 69.4, 69.21, 69.16, 64.2, 58.7, 57.0, 56.8, 53.9, 43.3, 41.2, 37.8, 36.9, 36.3, 35.3, 26.5, 17.6, 16.1, 14.5, 13.2, 11.9; LC–MS *m*/*z* calc. for C_61_H_85_ClN_8_O_16_S_3_ [M+2H]^2 +^ 658.3, found: 658.7.

#### 4.9.66 *tert*-butyl 4-(2-(2,6-dioxopiperidin-3-yl)-1,3-dioxoisoindolin-4-yl)piperazine-1-carboxylate (**76**)

2-(2,6-dioxopiperidin-3-yl)-4-fluoroisoindoline-1,3-dione (**19**) (50 mg, 18.1 μmol) and 1-Boc-piperazine (37 mg, 20.0 μmol) were dissolved in DMSO (1.1 mL). DIPEA (126.7 μL, 72.4 μmol) was then added and the reaction was stirred and heated to 90 °C in a closed microwave vial for 16 h. The mixture was then purified by reverse phase flash column chromatography (15.5 g C18 gold column) using a linear gradient from 0% to 100% MeCN in 0.1% HCOOH in water over 8 min to afford compound **76** as a yellow/orange solid. Yield: 58 mg (73%); Analytics were consistent with literature;^37^^1^H NMR (500 MHz, DMSO): δ = 11.06 (s, 1H), 7.72 (dd, *J* = 7.4, 8.2 Hz, 1H), 7.39 (d, *J* = 7.1 Hz, 1H), 7.35 (d, *J* = 8.4 Hz, 1H), 5.10 (dd, *J* = 5.5, 12.8 Hz, 1H), 3.54–3.49 (m, 4H), 3.27–3.23 (m, 4H), 2.92–2.84 (m, 1H), 2.63–2.51 (m, 2H), 2.05–2.01 (m, 1H), 1.43 ppm (s, 9H); LC–MS *m*/*z* calc. for C_18_H_18_N_4_O_6_ [M–(C(CH_3_)_3_)+H]^+^ 386.1, found: 386.9.

#### 4.9.67 *tert*-butyl 4-(2-(2,6-dioxopiperidin-3-yl)-6-fluoro-1,3-dioxoisoindolin-5-yl)piperazine-1-carboxylate (**77**)

2-(2,6-dioxopiperidin-3-yl)-5,6-difluoroisoindoline-1,3-dione (**65**) (50 mg, 17.0 μmol) and 1-Boc-piperazine (35 mg, 18.7 μmol) were dissolved in DMSO (1.1 mL). DIPEA (118.5 μL, 68.0 μmol) was then added and the reaction was stirred and heated to 90 °C in a closed microwave vial for 16 h. The mixture was then purified by reverse phase flash column chromatography (15.5 g C18 gold column) using a linear gradient from 0% to 100% MeCN in 0.1% HCOOH in water over 8 min to afford compound **77** as a pale-yellow solid. Yield: 72 mg (91%); Analytics were consistent with literature;^44^^1^H NMR (500 MHz, CDCl_3_): δ = 8.60 (br s, 1H), 7.47 (d, *J*_H–F_ = 10.8 Hz, 1H), 7.37 (d, *J*_H–F_ = 7.3 Hz, 1H), 4.93 (dd, *J* = 5.6, 12.2 Hz, 1H), 3.59 (dd, *J* = 4.9, 4.9 Hz, 4H), 3.20 (dd, *J* = 4.8, 4.8 Hz, 4H), 2.89–2.67 (m, 3H), 2.13–2.07 (m, 1H), 1.47 ppm (s, 9H); ^19^F NMR (471 MHz, CDCl_3_): δ = −111.01 (dd, *J*_F–H_ = 7.4, 10.8 Hz, 1F); LC–MS *m*/*z* calc. for C_18_H_17_FN_4_O_6_ [M–(C(CH_3_)_3_)+H]^+^ 404.1, found: 404.9.

#### 4.9.68 2-((allyloxy)methyl)-2-(hydroxymethyl)propane-1,3-diol (**79**)

Pentaerythritol (**78**) (3.0 g, 22.0 mmol) was dissolved/suspended in DMF (44 mL) and cooled 0 °C. 60% NaH in oil (1.32 g, 33.0 mmol) was then added and the reaction was left to stir at 0 °C for 15 min. Allyl bromide (2.23 mL, 26.4 mmol) was added dropwise and the reaction was left to stir at r.t. for 16 h. The mixture was then filtered through Celite and evaporated to dryness. The residue was purified by flash column chromatography (80 g silica column) using a linear gradient from 0% to 20% MeOH in DCM to afford **79** as a colorless oil. Yield: 1.364 g (35%); ^1^H NMR (400 MHz, CDCl_3_): δ = 5.93–5.83 (m, 1H), 5.26 (qd, *J* = 1.6, 17.2 Hz, 1H), 5.21 (qd, *J* = 1.3, 10.4 Hz, 1H), 3.99 (td, *J* = 1.4, 5.6 Hz, 2H), 3.74 (d, *J* = 5.6 Hz, 6H), 3.50 (s, 2H), 2.38 ppm (t, *J* = 5.6 Hz, 3H); ^13^C NMR (101 MHz, CDCl_3_): δ = 134.3, 117.6, 72.8, 72.6, 65.2, 45.1.

#### 4.9.69 2-((allyloxy)methyl)-2-((2-(2-(2-azidoethoxy)ethoxy)ethoxy)methyl)propane-1,3-diol (**80**)

Follow General Procedure A, using 1.0 eq. triol **79**, 1.2 eq. of NaH and 1.0 eq. of mesylate 2-(2-(2-azidoethoxy)ethoxy)ethylmethanesulfonate (**6**). Purified by reverse phase flash column chromatography (50 g C18 gold column) using a linear gradient over 12 min from 0% to 100% MeCN in 0.1% HCOOH in water. Yield: 155 mg (39%); ^1^H NMR (400 MHz, CDCl_3_): δ = 5.94–5.82 (m, 1H), 5.25 (dd, *J* = 1.5, 17.2 Hz, 1H), 5.19 (dd, *J* = 1.2, 10.4 Hz, 1H), 3.98 (d, *J* = 5.3 Hz, 2H), 3.70–3.62 (m, 14H), 3.59 (s, 2H), 3.48 (s, 2H), 3.42–3.36 (m, 2H), 2.76 ppm (br s, 2H); ^13^C NMR (101 MHz, CDCl_3_): δ = 134.5, 117.3, 72.7, 72.3, 70.88, 70.87, 70.7, 70.5, 70.2, 65.1, 50.9, 45.2.

#### 4.9.70 15-((allyloxy)methyl)-15-((2-(2-(2-azidoethoxy)ethoxy)ethoxy)methyl)-2,4,7,10,13,17,20,23,26,28-decaoxanonacosane (**81**)

Follow General Procedure A, using 1.0 eq. diol **80**, 4.0 eq. of NaH and 3.0 eq. of MOM mesylate **40**. Purified by reverse phase flash column chromatography (30 g C18 gold column) using a linear gradient over 10 min from 0% to 100% MeCN in 0.1% HCOOH in water. Yield: 191 mg (62%); ^1^H NMR (400 MHz, CDCl_3_): δ = 5.93–5.83 (m, 1H), 5.24 (dd, *J* = 1.6, 17.3 Hz, 1H), 5.12 (d, *J* = 10.4 Hz, 1H), 4.66 (s, 4H), 3.93 (d, *J* = 5.2 Hz, 2H), 3.72–3.52 (m, 34H), 3.46–3.36 ppm (m, 16H); ^13^C NMR (101 MHz, CDCl_3_): δ = 135.5, 116.1, 96.7, 72.4, 71.2, 71.0, 70.84, 70.78, 70.72, 70.66, 70.6, 70.3, 70.2, 69.4, 67.0, 55.3, 50.9, 45.7.

#### 4.9.71 15-((2-(2-(2-azidoethoxy)ethoxy)ethoxy)methyl)-15-(2,4,7,10,13-pentaoxatetradecan-14-yl)-2,4,7,10,13,17-hexaoxanonadecan-19-al (**82**)

Follow General Procedure B, using alkene **81**. Yield: 154 mg (81%); ^1^H NMR (400 MHz, CDCl_3_): δ = 9.73 (s, 1H), 4.65 (s, 4H), 4.01 (s, 2H), 3.72–3.53 (m, 36H), 3.48–3.35 ppm (m, 14H); ^13^C NMR (101 MHz, CDCl_3_): δ = 202.4, 96.7, 71.2, 70.9, 70.82, 70.77, 70.72, 70.65, 70.6, 70.2, 70.0, 67.0, 55.3, 50.9, 45.8.

#### 4.9.72 15-((2-(2-(2-azidoethoxy)ethoxy)ethoxy)methyl)-15-(2,4,7,10,13-pentaoxatetradecan-14-yl)-2,4,7,10,13,17-hexaoxanonadecan-19-oic Acid (**83**)

Follow General Procedure C, using aldehyde **82**. Yield: 155 mg (quant.); ^1^H NMR (400 MHz, CDCl_3_): δ = 4.65 (s, 4H), 3.98 (s, 2H), 3.72–3.54 (m, 36H), 3.49–3.45 (m, 6H), 3.42–3.35 ppm (m, 8H); ^13^C NMR (101 MHz, CDCl_3_): δ = 173.8, 96.5, 72.3, 71.2, 71.1, 71.0, 70.9, 70.63, 70.60, 70.5, 70.41, 70.37, 70.3, 70.1, 70.0, 66.7, 55.2, 50.7, 45.1.

#### 4.9.73 (2*S*,4*R*)-1-((*S*)-15-((2-(2-(2-azidoethoxy)ethoxy)ethoxy)methyl)-21-(*tert*-Butyl)-19-oxo-15-(2,4,7,10,13-pentaoxatetradecan-14-yl)-2,4,7,10,13,17-hexaoxa-20-azadocosan-22-oyl)-4-hydroxy-*N*-(4-(4-methylthiazol-5-yl)benzyl)pyrrolidine-2-carboxamide (**84**)

Follow General Procedure D, using carboxylic acid **83** and VH032-amine (**14**, synthesized through literature procedures^21,22^). Yield: 51 mg (64%); ^1^H NMR (400 MHz, CDCl_3_): δ = 8.65 (s, 1H), 7.41–7.28 (m, 5H), 7.13 (d, *J* = 8.8 Hz, 1H), 4.68 (t, *J* = 7.5 Hz, 1H), 4.62 (s, 4H), 4.58–4.48 (m, 3H), 4.32 (dd, *J* = 5.2, 15.0 Hz, 1H), 4.00 (d, *J* = 11.1 Hz, 1H), 3.93 (d, *J* = 15.3 Hz, 1H), 3.88 (d, *J* = 15.0 Hz, 1H), 3.68–3.45 (m, 37H), 3.42 (s, 6H), 3.38–3.32 (m, 8H), 2.54–2.44 (m, 4H), 2.29 (br s, 1H), 2.09 (dd, *J* = 8.3, 13.0 Hz, 1H), 0.93 ppm (s, 9H); ^13^C NMR (101 MHz, CDCl_3_): δ = 171.3, 170.9, 170.3, 150.3, 148.5, 138.3, 131.7, 131.0, 129.5, 128.2, 96.6, 71.2, 71.1, 71.03, 70.98, 70.8, 70.7, 70.6, 70.5, 70.4, 70.09, 70.07, 66.9, 58.6, 56.9, 56.7, 55.2, 50.8, 45.6, 43.3, 36.0, 35.2, 26.5, 16.1; LC–MS *m*/*z* calc. for C_51_H_86_ClN_7_O_18_S [M + H]^+^ 1116.6, found: 1116.4.

#### 4.9.74 (2*S*,4*R*)-1-((21*S*)-21-(*tert*-Butyl)-15-((2-(2-(2-((2-(2,6-dioxopiperidin-3-yl)-6-fluoro-1,3-dioxoisoindolin-5-yl)amino)ethoxy)ethoxy)ethoxy)methyl)-19-oxo-15-(2,4,7,10,13-pentaoxatetradecan-14-yl)-2,4,7,10,13,17-hexaoxa-20-azadocosan-22-oyl)-4-hydroxy-*N*-(4-(4-methylthiazol-5-yl)benzyl)pyrrolidine-2-carboxamide (**85**)

Follow General Procedure H, using azide **84** and 2-(2,6-dioxopiperidin-3-yl)-5,6-difluoroisoindoline-1,3-dione (**65**). Purified by HPLC using a linear gradient of 5% to 95% MeCN in 0.1% HCOOH in water over 15 min gradient. Yield: 27 mg (55%); Contains a mixture of two diastereomers; ^1^H NMR (500 MHz, CDCl_3_): δ = 8.68 (s, 1H), 8.66–8.61 (m, 1H), 7.45–7.38 (m, 2H), 7.38–7.32 (m, 4H), 7.14–7.11 (m, 2H), 5.28–5.22 (m, 1H), 4.90 (dd, *J* = 5.2, 12.0 Hz, 1H), 4.71 (t, *J* = 7.4 Hz, 1H), 4.64 (s, 4H), 4.57 (dd, *J* = 6.6, 14.9 Hz, 1H), 4.53 (s, 1H), 4.49 (d, *J* = 8.8 Hz, 1H), 4.35 (dd, *J* = 5.1, 14.7 Hz, 1H), 4.08 (d, *J* = 11.3 Hz, 1H), 3.94 (d, *J* = 15.4 Hz, 1H), 3.88 (d, *J* = 15.3 Hz, 1H), 3.75 (t, *J* = 5.1 Hz, 2H), 3.70–3.35 (m, 49H), 2.90–2.66 (m, 3H), 2.56–2.48 (m, 4H), 2.16–2.10 (m, 2H), 1.81 (br s, 1H), 0.94 ppm (s, 9H); ^13^C NMR (126 MHz, CDCl_3_): δ = 171.5, 171.42, 171.36, 170.9, 170.6, 168.53, 168.49, 167.5, 167.04, 167.02, 153.96 (d, *J*_C–F_ = 248.1 Hz), 150.4, 148.6, 142.84 (d, *J*_C–F_ = 12.5 Hz), 138.37, 138.35, 131.7, 131.0, 130.16 (d, *J*_C–F_ = 1.8 Hz), 129.60, 129.58, 128.3, 118.68 (d, *J*_C–F_ = 8.9 Hz), 110.30 (d, *J*_C–F_ = 21.3 Hz), 106.01–105.96 (m), 96.7, 71.13, 71.05, 71.0, 70.7, 70.6, 70.5, 70.4, 70.3, 70.0, 69.2, 69.1, 66.9, 58.6, 58.6, 57.02, 56.99, 56.8, 55.3, 49.4, 45.7, 43.3, 43.1, 43.0, 36.10, 36.06, 35.1, 31.6, 26.5, 22.9, 16.2; ^19^F NMR (471 MHz, CDCl_3_): δ = −127.24–127.33 (m, 1F); LC–MS *m*/*z* calc. for C_64_H_95_FN_7_O_22_S [M + H]^+^ 1364.6, found: 1365.0.

#### 4.9.75 11-((2-(2-(2-((2-(2,6-dioxopiperidin-3-yl)-6-fluoro-1,3-dioxoisoindolin-5-yl)amino)ethoxy)ethoxy)ethoxy)methyl)-11-((2-(((*S*)-1-((2*S*,4*R*)-4-hydroxy-2-((4-(4-methylthiazol-5-yl)benzyl)carbamoyl)pyrrolidin-1-yl)-3,3-dimethyl-1-oxobutan-2-yl)amino)-2-oxoethoxy)methyl)-3,6,9,13,16,19-hexaoxahenicosane-1,21-diyl bis(2-((*S*)-4-(4-chlorophenyl)-2,3,9-trimethyl-6*H*-thieno[3,2-*f*][1,2,4]triazolo[4,3-*a*][1,4]diazepin-6-yl)acetate) (AB3124) (**86**)

Follow General Procedure E, using compound **85** and 3.0 eq of JQ1-acid (**22**). Purified by HPLC using a linear gradient from 20% to 95% MeCN in 0.1% HCOOH in water over 15 min. Yield: 8.9 mg (22%); Contains a mixture of two diastereomers; ^1^H NMR (500 MHz, CDCl_3_): δ = 8.68 (s, 1H), 8.60 (s, 1H), 7.49–7.44 (m, 1H), 7.41–7.30 (m, 13H), 7.18 (d, *J* = 8.8 Hz, 1H), 7.10 (d, *J*_H–F_ = 7.2 Hz, 1H), 5.35–5.29 (m, 1H), 4.89 (dd, *J* = 5.2, 12.0 Hz, 1H), 4.71 (t, *J* = 8.0 Hz, 1H), 4.60 (t, *J* = 7.0 Hz, 2H), 4.58–4.53 (m, 3H), 4.38 (dd, *J* = 5.4, 14.9 Hz, 1H), 4.35–4.24 (m, 4H), 4.04 (d, *J* = 11.1 Hz, 1H), 3.95 (d, *J* = 15.4 Hz, 1H), 3.90 (d, *J* = 15.2 Hz, 1H), 3.77–3.70 (m, 6H), 3.70–3.41 (m, 40H), 2.88–2.66 (m, 9H), 2.50 (s, 3H), 2.49–2.40 (m, 7H), 2.20 (dd, *J* = 7.8, 13.0 Hz, 1H), 2.14–2.07 (m, 1H), 1.68 (s, 6H), 0.96 ppm (s, 9H); ^13^C NMR (126 MHz, CDCl_3_): δ = 171.7, 171.4, 171.35, 171.28, 171.18, 171.15, 170.2, 168.51, 168.47, 167.5, 167.03, 167.01, 164.0, 162.8, 155.4, 153.94 (d, *J*_C–F_ = 248.6 Hz), 150.4, 150.1, 148.5, 142.88 (d, *J*_C–F_ = 12.8 Hz), 138.5, 136.9, 136.7, 132.3, 131.8, 131.02, 130.98, 130.9, 130.5, 130.16 (d, *J*_C–F_ = 1.8 Hz), 130.0, 129.54, 129.53, 128.8, 128.2, 118.58 (d, *J*_C–F_ = 8.9 Hz), 110.27 (d, *J*_C–F_ = 21.9 Hz), 105.96 (d, *J*_C–F_ = 5.5 Hz), 71.2, 71.1, 70.8, 70.7, 70.6, 70.5, 70.4, 70.2, 70.0, 69.2, 69.14, 69.09, 64.1, 58.9, 56.9, 56.8, 53.8, 49.4, 45.7, 36.9, 36.6, 35.6, 31.6, 26.5, 22.9, 16.2, 14.6, 13.2, 11.9; ^19^F{^1^H} NMR (471 MHz, CDCl_3_): δ = −127.22*, −127.26* (1F); HRMS *m*/*z* calc. for C_98_H_117_Cl_2_FN_15_O_22_S_3_ [M + H]^+^ 2040.7015, found: 2040.6811.

#### 4.9.76 5,6-difluoro-2-(1-methyl-2,6-dioxopiperidin-3-yl) Isoindoline-1,3-dione (**87**)

2-(2,6-dioxopiperidin-3-yl)-5,6-difluoroisoindoline-1,3-dione (**65**) (50 mg, 17.0 μmol) was dissolved in DMF (500 μL). K_2_CO_3_ (47 mg, 34.0 μmol) was then added, and the flask was cooled to 0 °C under N_2_. Methyl iodide (29 mg, 20.4 μmol) was then added, and the reaction was stirred vigorously at r.t. for 5.5 h. The reaction was filtered with PTFE syringe filters before concentrating under vacuum. The residue was purified by reverse phase flash column chromatography (15.5 g C18 gold column) using a linear gradient over 9 min from 0% to 70% MeCN in 0.1% HCOOH in water to afford **87** as a white solid. Yield: 42 mg (80%); ^1^H NMR (500 MHz, CDCl_3_): δ = 7.69 (dd, *J*_H–H_ = 7.2, *J*_H–F_ = 7.2 Hz, 2H), 4.99–4.94 (m, 1H), 3.18 (s, 3H), 3.05–2.91 (m, 1H), 2.83–2.73 (m, 2H), 2.15–2.06 ppm (m, 1H); ^13^C NMR (126 MHz, CDCl_3_): δ = 171.0, 168.5, 165.5, 154.76 (dd, *J*_C–F_ = 15.6, 262.3 Hz), 128.71 (dd, *J*_C–F_ = 5.6, 5.6 Hz), 113.78 (dt, *J*_C–F_ = 10.1, 16.5 Hz), 50.6, 31.9, 27.4, 22.0; ^19^F NMR (471 MHz, CDCl_3_): δ = −124.58 (dd, *J*_F–F_ = 7.3, *J*_F–H_ = 7.3, 2F). LC–MS *m*/*z* calc. for C_14_H_11_F_2_N_2_O_4_ [M + H]^+^ 309.1, found: 309.0.

#### 4.9.77 (2*S*,4*S*)-1-((21*S*)-15-((2-(2-(2-azidoethoxy)ethoxy)ethoxy)methyl)-21-(*tert*-Butyl)-15-methyl-19-oxo-2,4,7,10,13,17-hexaoxa-20-azadocosan-22-oyl)-4-hydroxy-*N*-((*S*)-1-(4-(4-methylthiazol-5-yl)phenyl)ethyl)pyrrolidine-2-carboxamide (**89**)

Follow General Procedure D, using carboxylic acid **53** and *cis*-Me-VH032-amine (**88**, synthesized according to literature procedures^38^). Yield: 50 mg (56%); Contains a mixture of two diastereomers; ^1^H NMR (500 MHz, CDCl_3_): δ = 8.79 (s, 1H), 7.64 (d, *J* = 7.9 Hz, 1H), 7.40 (d, *J* = 8.3 Hz, 2H), 7.37 (d, *J* = 8.3 Hz, 2H), 7.09 (d, *J* = 9.1 Hz, 1H), 5.06 (dq, *J* = 7.1, 7.1 Hz, 1H), 4.72 (d, *J* = 9.0 Hz, 1H), 4.62 (s, 2H), 4.53 (d, *J* = 9.2 Hz, 1H), 4.46–4.42 (m, 1H), 3.97–3.89 (m, 3H), 3.79 (d, *J* = 11.1 Hz, 1H), 3.68–3.52 (m, 22H), 3.43 (d, *J* = 9.0 Hz, 1H), 3.39 (d, *J* = 8.9 Hz, 1H), 3.38–3.29 (m, 9H), 2.54 (s, 3H), 2.31 (d, *J* = 14.0 Hz, 1H), 2.13 (ddd, *J* = 5.0, 9.2, 14.2 Hz, 1H), 1.48 (d, *J* = 6.8 Hz, 3H), 1.04 (s, 9H), 0.98 ppm (s, 3H); ^13^C NMR (126 MHz, CDCl_3_): δ = 171.9, 171.7, 169.9, 150.7, 147.9, 142.8, 132.0, 130.8, 129.7, 126.6, 96.6, 74.6, 73.9, 73.8, 71.12, 71.09, 71.07, 70.9, 70.8, 70.72, 70.68, 70.6, 70.55, 70.47, 70.1, 66.8, 59.9, 58.7, 56.4, 55.2, 50.7, 49.3, 41.1, 35.3, 34.8, 26.5, 22.0, 17.5, 15.8; LC–MS *m*/*z* calc. for C_44_H_70_N_7_O_13_S [M-H]^−^ 936.5, found: 936.3.

#### 4.9.78 (2*S*,4*R*)-1-((21*S*)-21-(*tert*-Butyl)-15-((2-(2-(2-((6-fluoro-2-(1-methyl-2,6-dioxopiperidin-3-yl)-1,3-dioxoisoindolin-5-yl)amino)ethoxy)ethoxy)ethoxy)methyl)-15-methyl-19-oxo-2,4,7,10,13,17-hexaoxa-20-azadocosan-22-oyl)-4-hydroxy-*N*-((*S*)-1-(4-(4-methylthiazol-5-yl)phenyl)ethyl)pyrrolidine-2-carboxamide (**90**)

Follow General Procedure H, using azide **60** and thalidomide derivative **87**. Yield: 13 mg (27%); Contains a mixture of four diastereomers; ^1^H NMR (500 MHz, CDCl_3_): δ = 8.68 (s, 1H), 7.44 (d, *J* = 7.7 Hz, 1H), 7.42–7.38 (m, 3H), 7.36 (d, *J* = 8.3 Hz, 2H), 7.18 (d, *J* = 8.6 Hz, 1H), 7.08 (d, *J*_H–F_ = 7.1 Hz, 1H), 5.31–5.24 (m, 1H), 5.07 (dq, *J* = 7.0, 7.1 Hz, 1H), 4.93–4.88 (m, 1H), 4.73 (t, *J* = 7.6 Hz, 1H), 4.64 (s, 2H), 4.54–4.48 (m, 2H), 4.10 (d, *J* = 11.7 Hz, 1H), 3.95 (d, *J* = 15.9 Hz, 1H), 3.92 (d, *J* = 16.2 Hz, 1H), 3.75 (t, *J* = 5.0 Hz, 2H), 3.70–3.53 (m, 21H), 3.46–3.38 (m, 4H), 3.38–3.31 (m, 7H), 3.19 (s, 3H), 3.01–2.92 (m, 1H), 2.82–2.70 (m, 2H), 2.56–2.47 (m, 4H), 2.12–2.04 (m, 2H), 1.47 (d, *J* = 6.9 Hz, 3H), 1.05 (s, 9H), 0.98 ppm (s, 3H); ^13^C NMR (126 MHz, CDCl_3_): δ = 171.6, 171.3, 170.7, 169.8, 169.1, 167.7, 167.2, 162.8, 154.0 (d, *J*_C–F_ = 249.0 Hz), 150.5, 148.5, 143.3, 142.73 (d, *J*_C–F_ = 12.7 Hz), 131.7, 130.9, 130.2, 129.7, 126.6, 118.79 (d, *J*_C–F_ = 9.0 Hz), 110.24 (d, *J*_C–F_ = 22.7 Hz), 105.81 (d, *J*_C–F_ = 5.2 Hz), 96.6, 74.5, 73.9, 71.2, 71.1, 70.9, 70.7, 70.64, 70.57, 70.5, 70.2, 68.9, 66.9, 58.4, 57.1, 56.7, 55.3, 50.2, 49.0, 43.0, 41.1, 35.5, 35.1, 32.1, 27.4, 26.6, 22.4, 22.2, 17.6, 16.2; ^19^F NMR (471 MHz, CDCl_3_): δ = −127.29–127.35 (m, 1F); LC–MS *m*/*z* calc. for C_58_H_83_N_7_O_17_S [M + H]^+^ 1200.6, found: 1200.4.

#### 4.9.79 (2*S*,4*S*)-1-((21*S*)-21-(*tert*-Butyl)-15-((2-(2-(2-((2-(2,6-dioxopiperidin-3-yl)-6-fluoro-1,3-dioxoisoindolin-5-yl)amino)ethoxy)ethoxy)ethoxy)methyl)-15-methyl-19-oxo-2,4,7,10,13,17-hexaoxa-20-azadocosan-22-oyl)-4-hydroxy-*N*-((*S*)-1-(4-(4-methylthiazol-5-yl)phenyl)ethyl)pyrrolidine-2-carboxamide (**91**)

Follow General Procedure H, using azide **89** and 2-(2,6-dioxopiperidin-3-yl)-5,6-difluoroisoindoline-1,3-dione (**65**). Yield: 11 mg (34%); Contains a mixture of four diastereomers; ^1^H NMR (500 MHz, CDCl_3_): δ = 8.89–8.65 (m, 2H), 7.76–7.69 (m, 1H), 7.44–7.40 (m, 3H), 7.38 (d, *J* = 8.3 Hz, 2H), 7.14–7.09 (m, 2H), 5.62–5.57 (m, 1H), 5.26–5.20 (m, 1H), 5.08 (dq, *J* = 7.0, 7.1 Hz, 1H), 4.93–4.87 (m, 1H), 4.72 (d, *J* = 8.4 Hz, 1H), 4.64 (s, 2H), 4.58–4.53 (m, 1H), 4.49–4.43 (m, 1H), 3.97–3.90 (m, 3H), 3.86–3.81 (m, 1H), 3.77–3.74 (m, 2H), 3.70–3.51 (m, 20H), 3.48–3.25 (m, 11H), 2.91–2.66 (m, 3H), 2.53 (s, 3H), 2.30 (d, *J* = 14.3 Hz, 1H), 2.19–2.09 (m, 2H), 1.52–1.47 (m, 3H), 1.06 (s, 9H), 0.97–0.93 ppm (m, 3H); ^13^C NMR (126 MHz, CDCl_3_): δ = 171.99, 171.94, 171.73, 171.71, 171.51, 171.45, 171.4, 170.1, 168.5, 168.4, 167.5, 167.0, 153.98 (d, *J*_C–F_ = 248.7 Hz), 150.5, 148.7, 142.85 (d, *J*_C–F_ = 12.4 Hz), 142.60, 142.57, 131.6, 131.3, 130.2, 129.8, 126.6, 118.75 (d, *J*_C–F_ = 8.6 Hz), 110.30 (d, *J*_C–F_ = 22.6 Hz), 105.97–105.91 (m), 96.7, 74.5, 73.9, 71.21, 71.19, 71.1, 71.0, 70.8, 70.74, 70.65, 70.6, 70.5, 69.1, 69.0, 66.9, 60.2, 60.1, 58.8, 56.5, 55.3, 49.4, 49.4, 43.0, 41.1, 35.3, 35.05, 35.01, 31.6, 26.5, 22.9, 22.0, 17.5, 16.2; ^19^F NMR (471 MHz, CDCl_3_): δ = −127.22–127.38 (m, 1F); LC–MS *m*/*z* calc. for C_57_H_81_FN_7_O_17_S [M + H]^+^ 1186.5, found: 1186.4.

#### 4.9.80 (2*S*,4*S*)-1-((21*S*)-21-(*tert*-Butyl)-15-((2-(2-(2-((6-fluoro-2-(1-methyl-2,6-dioxopiperidin-3-yl)-1,3-dioxoisoindolin-5-yl)amino)ethoxy)ethoxy)ethoxy)methyl)-15-methyl-19-oxo-2,4,7,10,13,17-hexaoxa-20-azadocosan-22-oyl)-4-hydroxy-*N*-((*S*)-1-(4-(4-methylthiazol-5-yl)phenyl)ethyl)pyrrolidine-2-carboxamide (**92**)

Follow General Procedure H, using azide **89** and thalidomide derivative **87**. Yield: 9.5 mg (30%); Contains a mixture of four diastereomers; ^1^H NMR (500 MHz, CDCl_3_): δ = 8.68 (s, 1H), 7.62 (d, *J* = 7.8 Hz, 1H), 7.44–7.39 (m, 3H), 7.37 (d, *J* = 8.3 Hz, 2H), 7.12–7.07 (m, 2H), 5.45 (d, *J* = 8.9 Hz, 1H), 5.23 (dd, *J* = 4.8, 8.4 Hz, 1H), 5.08 (dq, *J* = 7.1, 7.2 Hz, 1H), 4.93–4.87 (m, 1H), 4.74 (d, *J* = 9.0 Hz, 1H), 4.64 (s, 2H), 4.56 (d, *J* = 9.2 Hz, 1H), 4.49–4.44 (m, 1H), 3.98–3.91 (m, 3H), 3.82 (d, *J* = 10.8 Hz, 1H), 3.75 (t, *J* = 4.9 Hz, 2H), 3.70–3.54 (m, 20H), 3.46–3.39 (m, 4H), 3.38–3.32 (m, 7H), 3.19 (s, 3H), 3.01–2.92 (m, 1H), 2.82–2.70 (m, 2H), 2.53 (s, 3H), 2.34 (d, *J* = 14.4 Hz, 1H), 2.17–2.06 (m, 2H), 1.50 (d, *J* = 6.8 Hz, 3H), 1.07 (s, 9H), 0.99 ppm (s, 3H); ^13^C NMR (126 MHz, CDCl_3_): δ = 172.0, 171.7, 171.3, 170.0, 169.1, 167.7, 167.2, 154.01 (d, *J*_C–F_ = 248.1 Hz), 150.5, 148.7, 142.71 (d, *J*_C–F_ = 12.9 Hz), 142.5, 131.6, 131.3, 130.2, 118.89 (d, *J*_C–F_ = 8.9 Hz), 110.29 (d, *J*_C–F_ = 22.4 Hz), 105.79 (d, *J*_C–F_ = 4.9 Hz), 96.7, 74.6, 73.95, 73.91, 71.22, 71.16, 71.0, 70.8, 70.7, 70.61, 70.55, 68.9, 66.9, 60.0, 58.9, 56.5, 55.3, 50.2, 49.4, 43.0, 41.2, 35.4, 34.9, 32.1, 27.4, 26.5, 22.2, 22.0, 17.6, 16.2; ^19^F NMR (471 MHz, CDCl_3_): δ = −127.38–127.42 (m, 1F); LC–MS *m*/*z* calc. for C_58_H_83_N_7_O_17_S [M + H]^+^ 1200.6, found: 1200.8.

#### 4.9.81 (17*S*)-11-((2-(2-(2-((6-fluoro-2-(1-methyl-2,6-dioxopiperidin-3-yl)-1,3-dioxoisoindolin-5-yl)amino)ethoxy)ethoxy)ethoxy)methyl)-17-((2*S*,4*R*)-4-hydroxy-2-(((*S*)-1-(4-(4-methylthiazol-5-yl)phenyl)ethyl)carbamoyl)pyrrolidine-1-carbonyl)-11,18,18-trimethyl-15-oxo-3,6,9,13-tetraoxa-16-azanonadecyl 2-((*S*)-4-(4-chlorophenyl)-2,3,9-trimethyl-6*H*-thieno[3,2-*f*][1,2,4]triazolo[4,3-*a*][1,4]diazepin-6-yl)acetate (*neg*-AB3067) (**93**)

Follow General Procedure E, using compound **90** and 1.5 equiv of JQ1-acid (**22**). Purified by HPLC using a linear gradient over 10 min from 30% to 95% MeCN in 0.1% HCOOH in water. Yield: 2.2 mg (13%); Contains a mixture of four diastereomers; ^1^H NMR (500 MHz, CDCl_3_): δ = 8.67 (s, 1H), 7.44 (d, *J* = 7.9 Hz, 1H), 7.42–7.34 (m, 7H), 7.32 (d, *J* = 8.6 Hz, 2H), 7.18 (d, *J* = 8.4 Hz, 1H), 7.08 (d, *J*_H–F_ = 7.0 Hz, 1H), 5.30–5.25 (m, 1H), 5.08 (dq, *J* = 7.2, 7.2 Hz, 1H), 4.90 (dd, *J* = 5.5, 12.5 Hz, 1H), 4.74 (t, *J* = 7.9 Hz, 1H), 4.60 (dd, *J* = 6.2, 7.9 Hz, 1H), 4.55–4.49 (m, 2H), 4.36–4.26 (m, 2H), 4.11 (d, *J* = 11.3 Hz, 1H), 3.98–3.90 (m, 2H), 3.77–3.73 (m, 4H), 3.68–3.56 (m, 19H), 3.47–3.40 (m, 4H), 3.39–3.31 (m, 4H), 3.19 (s, 3H), 2.98–2.93 (m, 1H), 2.82–2.70 (m, 2H), 2.66 (s, 3H), 2.55–2.48 (m, 4H), 2.41 (s, 3H), 2.12–2.06 (m, 2H), 1.68 (s, 3H), 1.48 (d, *J* = 7.0 Hz, 3H), 1.06 (s, 9H), 1.00–0.98 ppm (m, 3H); ^19^F{^1^H} NMR (471 MHz, CDCl_3_): δ = −127.31*, −127.31*, −127.34*, −127.34* (1F); HRMS *m*/*z* calc. for C_75_H_94_ClFN_11_O_17_S_2_ [M + H]^+^ 1538.5938, found: 1538.6122.

#### 4.9.82 (17*S*)-11-((2-(2-(2-((2-(2,6-dioxopiperidin-3-yl)-6-fluoro-1,3-dioxoisoindolin-5-yl)amino)ethoxy)ethoxy)ethoxy)methyl)-17-((2*S*,4*S*)-4-hydroxy-2-(((*S*)-1-(4-(4-methylthiazol-5-yl)phenyl)ethyl)carbamoyl)pyrrolidine-1-carbonyl)-11,18,18-trimethyl-15-oxo-3,6,9,13-tetraoxa-16-azanonadecyl 2-((*S*)-4-(4-chlorophenyl)-2,3,9-trimethyl-6*H*-thieno[3,2-*f*][1,2,4]triazolo[4,3-*a*][1,4]diazepin-6-yl)acetate (*cis*-AB3067) (**94**)

Follow General Procedure E, using compound **91** and 1.5 equiv of JQ1-acid (**22**). Purified by HPLC using a linear gradient over 10 min from 30% to 95% MeCN in 0.1% HCOOH in water. Yield: 1.2 mg (8%); Contains a mixture of four diastereomers; ^1^H NMR (500 MHz, CDCl_3_): δ = 8.74–8.55 (m, 2H), 7.76–7.71 (m, 1H), 7.43–7.36 (m, 7H), 7.32 (d, *J* = 8.6 Hz, 2H), 7.14–7.09 (m, 2H), 5.60–5.56 (m, 1H), 5.28–5.24 (m, 1H), 5.09 (dq, *J* = 7.1, 7.2 Hz, 1H), 4.93–4.87 (m, 1H), 4.73 (d, *J* = 9.0 Hz, 1H), 4.60 (dd, *J* = 6.1, 7.9 Hz, 1H), 4.56 (t, *J* = 8.0 Hz, 1H), 4.48–4.43 (m, 1H), 4.33–4.29 (m, 2H), 3.96–3.88 (m, 3H), 3.86–3.82 (m, 1H), 3.77–3.73 (m, 4H), 3.68–3.53 (m, 18H), 3.48–3.29 (m, 8H), 2.90–2.70 (m, 3H), 2.66 (s, 3H), 2.53 (s, 3H), 2.41 (s, 3H), 2.31 (d, *J* = 14.0 Hz, 1H), 2.18–2.10 (m, 2H), 1.68 (s, 3H), 1.52–1.47 (m, 3H), 1.07 (s, 9H), 0.98–0.94 ppm (m, 3H); ^19^F{^1^H} NMR (471 MHz, CDCl_3_): δ = −127.24*, −127.28*, −127.32*, −127.35* (1F); HRMS *m*/*z* calc. for C_74_H_92_ClFN_11_O_17_S_2_ [M + H]^+^ 1524.5781, found: 1524.7017.

#### 4.9.83 (17*S*)-11-((2-(2-(2-((6-fluoro-2-(1-methyl-2,6-dioxopiperidin-3-yl)-1,3-dioxoisoindolin-5-yl)amino)ethoxy)ethoxy)ethoxy)methyl)-17-((2*S*,4*S*)-4-hydroxy-2-(((*S*)-1-(4-(4-methylthiazol-5-yl)phenyl)ethyl)carbamoyl)pyrrolidine-1-carbonyl)-11,18,18-trimethyl-15-oxo-3,6,9,13-tetraoxa-16-azanonadecyl 2-((*S*)-4-(4-chlorophenyl)-2,3,9-trimethyl-6*H*-thieno[3,2-*f*][1,2,4]triazolo[4,3-*a*][1,4]diazepin-6-yl)acetate (*neg*-*cis*-AB3067) (**95**)

Follow General Procedure E, using compound **92** and 1.5 equiv of JQ1-acid (**22**). Purified by HPLC using a linear gradient over 10 min from 30% to 95% MeCN in 0.1% HCOOH in water. Yield: 0.7 mg (9%); Contains a mixture of four diastereomers; ^1^H NMR (500 MHz, CDCl_3_): δ = 8.68 (s, 1H), 7.65 (d, *J* = 8.1 Hz, 1H), 7.39 (q, *J* = 9.9 Hz, 7H), 7.32 (d, *J* = 8.7 Hz, 2H), 7.11 (d, *J* = 9.2 Hz, 1H), 7.08 (d, *J*_H–F_ = 7.1 Hz, 1H), 5.48 (d, *J* = 9.1 Hz, 1H), 5.29–5.24 (m, 1H), 5.08 (dq, *J* = 7.2, 7.2 Hz, 1H), 4.90 (dd, *J* = 5.2, 12.3 Hz, 1H), 4.75 (d, *J* = 9.0 Hz, 1H), 4.60 (dd, *J* = 6.2, 7.9 Hz, 1H), 4.56 (d, *J* = 9.3 Hz, 1H), 4.48–4.43 (m, 1H), 4.35–4.27 (m, 2H), 3.98–3.92 (m, 3H), 3.82 (d, *J* = 10.9 Hz, 1H), 3.77–3.73 (m, 4H), 3.70–3.56 (m, 19H), 3.47–3.40 (m, 4H), 3.38–3.33 (m, 4H), 3.19 (s, 3H), 3.01–2.93 (m, 1H), 2.82–2.70 (m, 2H), 2.66 (s, 3H), 2.53 (s, 3H), 2.41 (s, 3H), 2.33 (d, *J* = 14.2 Hz, 1H), 2.17–2.07 (m, 2H), 1.68 (s, 3H), 1.50 (d, *J* = 6.9 Hz, 3H), 1.07 (s, 9H), 1.00 ppm (s, 3H); ^19^F{^1^H} NMR (471 MHz, CDCl_3_): δ = −127.37*, −127.37*, −127.37*, −127.37* (1F); HRMS *m*/*z* calc. for C_75_H_94_ClFN_11_O_17_S_2_ [M + H]^+^ 1538.5938, found: 1538.6201.

#### 4.9.84 (17*S*)-11-((2-(2-(2-((2-(2,6-dioxopiperidin-3-yl)-6-fluoro-1,3-dioxoisoindolin-5-yl)amino)ethoxy)ethoxy)ethoxy)methyl)-17-((2*S*,4*R*)-4-hydroxy-2-(((*S*)-1-(4-(4-methylthiazol-5-yl)phenyl)ethyl)carbamoyl)pyrrolidine-1-carbonyl)-11,18,18-trimethyl-15-oxo-3,6,9,13-tetraoxa-16-azanonadecyl (2*R*)-2-((*S*)-4-(4-chlorophenyl)-2,3,9-trimethyl-6*H*-thieno[3,2-*f*][1,2,4]triazolo[4,3-*a*][1,4]diazepin-6-yl)butanoate (AB3145) (**97**)

Follow General Procedure E, using compound **70** and 1.5 equiv of ET-JQ1-OH (**96**, synthesized through literature procedures^43^). Purified by HPLC using a linear gradient over 10 min from 30% to 95% MeCN in 0.1% HCOOH in water. Yield: 2.2 mg (16%); Contains a mixture of four diastereomers; ^1^H NMR (500 MHz, CDCl_3_): δ = 8.67 (s, 1H), 8.53–8.48 (m, 1H), 7.48 (d, *J* = 7.8 Hz, 1H), 7.42–7.35 (m, 5H), 7.35–7.29 (m, 4H), 7.19 (d, *J* = 9.0 Hz, 1H), 7.12 (d, *J*_H–F_ = 7.1 Hz, 1H), 5.31–5.26 (m, 1H), 5.08 (dq, *J* = 7.1, 7.2 Hz, 1H), 4.90 (dd, *J* = 5.3, 12.0 Hz, 1H), 4.73 (dd, *J* = 7.8, 7.8 Hz, 1H), 4.55–4.49 (m, 2H), 4.47–4.41 (m, 1H), 4.37–4.33 (m, 1H), 4.24 (d, *J* = 10.9 Hz, 1H), 4.12 (d, *J* = 11.1 Hz, 1H), 4.01–3.88 (m, 3H), 3.82–3.72 (m, 4H), 3.70–3.53 (m, 17H), 3.48–3.28 (m, 8H), 2.91–2.68 (m, 3H), 2.66 (s, 3H), 2.53–2.47 (m, 4H), 2.41 (s, 3H), 2.17–2.06 (m, 3H), 1.73–1.57 (m, 4H), 1.47 (dd, *J* = 2.4, 6.9 Hz, 3H), 1.06–0.99 (m, 12H), 0.98–0.94 ppm (m, 3H); ^19^F{^1^H} NMR (471 MHz, CDCl_3_) δ = −127.23*, −127.26*, −127.26*, −127.30* (1F); HRMS *m*/*z* calc. for C_76_H_96_ClFN_11_O_17_S_2_ [M + H]^+^ 1552.6094, found: 1552.5944.

### 4.10 Biology

#### 4.10.1 CRISPR Knock-In Cell Line Generation for HiBiT-BRD2/3/4 in a HEK293 Cell Line Stably Expressing an 18 kDa LgBiT protein

HEK293 HiBiT-BRD2 (LgBiT stable), HEK293 HiBiT-BRD3 (LgBiT stable), and HEK293 HiBiT-BRD4 (LgBiT stable) were created using CRISPR/Cas9 to insert at HiBiT tag at the N-terminal genomic loci of BRD2, BRD3, or BRD4 in a cell line stably expressing LgBiT protein as described previously.^23^

#### 4.10.2 Live Kinetics HiBiT-BET Degradation Experiments

For kinetic degradation experiments, HEK293 HiBiT-BRD2, 3, or 4 (LgBiT stable) cells and HEK293 HiBiT-BRD4 (LgBiT) CRBN KO and VHL KO cells were plated at 20,000 cells/well in DMEM +10% FBS in white 96-well tissue culture plates and allowed to adhere overnight. The next day, media was removed and 90 μL of prewarmed CO_2_-independent medium (Gibco) containing Endurazine (Promega) at a 1:100 dilution from stock was added. Plates were incubated at 37 °C for 3 h to allow luminescence to equilibrate. A threefold dilution series of each PROTAC at 10x the desired final concentration was prepared (with the amount of DMSO in each treatment condition kept constant). After luminescence equilibration, a preread luminescence measurement was taken (prior to compound addition) to allow for baseline normalization to account for possible plating differences. Cells were then treated with 10 μL of the prepared PROTAC dilution series (in triplicate or quadruplicate). Treated plates were placed in a GloMax Discover luminometer (Promega) prewarmed to 37 °C and luminescence measurements were obtained every 7 min for 24 h. Kinetic degradation plots were obtained by first normalizing time course luminescence readings in each well to the preread measurement, then to the average value of the DMSO only control at each time point. Data was analyzed using GraphPad Prism (version 8) and excel. *D*_max_ values were identified as the lowest luminescence reading in each well. Rate constant values were calculated using a one phase exponential decay *Y* = (*Y*_0_-*Plateau*) e(K*^X^) + *Plateau* using Prism (Y_0_ constrained to 1). *D*_max 50_ values were calculated by plotting *D*_max_ as a function of concentration, then fitting plots using the following equation:  to determine IC_50_.

#### 4.10.3 Generation of VHL and CRBN Single and Double Knockout RKO cell Lines

RKO WT, VHL KO, CRBN KO and CRBN/VHL dKO cells were cultured in Dulbecco’s modified Eagle’s medium supplemented with 10% FCS and 1% penicillin–streptomycin. RKO CRBN KO and VHL KO were generated previously.^45^ RKO CRBN/VHL dKO cells were generated by transiently expressing pSpCas9(BB)-2A-GFP (PX458) (Addgene 48138) loaded with a short guide RNA (sgRNA) targeting VHL (Fwd: CACCGGCGATTGCAGAAGATGACCT, Rev: AAACAGGTCATCTTCTGCAATCGCC) in RKO CRBN KO cells. Clones were single cell seeded and checked for VHL and CRBN double deletion via Western blot or PCR on genomic DNA (gDNA).

#### 4.10.4 Cell Viability Assays

Cells were seeded in 96-well plates at a cell density of 3750 cells per well and treated for 3 days with DMSO or drug at ten √10 serial diluted concentrations. Starting concentration for all drugs was 10 μM, and each treatment was performed in biological triplicates. Cell viability was assessed using the CellTiter-Glo assay (CellTiter-Glo Luminescent Cell Viability Assay, Promega G7573) according to manufacturer instructions. Luminescence signal was measured on a Multilabel Plate Reader Platform Victor X3 model 2030 (PerkinElmer). Survival curves and EC_50_ values were determined using GraphPad Prism v.10.0.3 by fitting a nonlinear regression to the log_10_-transformed drug concentration and the relative viability after normalization of each data point to the mean luminescence of the lowest drug concentration.

#### 4.10.5 Western Blot Evaluation of Heterotrivalent PROTACs 23–32 in HEK293 cells

##### 4.10.5.1 Cell Culture

HEK293 cells were obtained from ATCC. HEK293 was cultured in DMEM supplemented with 10% (v/v) fetal bovine serum (FBS), 1% l-glutamate and 1% (v/v) penicillin/streptomycin (pen/strep) at 37 °C, 5% CO_2_, and 95% humidity.

##### 4.10.5.2 Heterotrivalent PROTACs 23–32 Dose–Response Degradation Assays

HEK293 cells were plated at a density of 5 × 10^5^ cells per well of a six well plate a day prior to initiation of the experiment. Compounds were dissolved to a 10 mM concentrated stock solution in DMSO from which the compounds were further diluted to a working concentration range of 1 μM to 100 pM in DMEM and was subsequently added to cells at the initiation of the experiment. An additional vehicle control was added to cells alongside compound treatments. Cells were left to incubate with compound for 6 h at 37 °C, 5% CO_2_, and 95% humidity. Cells were then subsequently washed twice with PBS before being harvested in RIPA buffer supplemented with protease inhibitor cocktail and Benzonase Nuclease before being store at −20 °C.

##### 4.10.5.3 Western Blotting Analysis of Heterotrivalent PROTACs 23–32 Dose–Response Degradation Assay

Protein concentration was determined using the BCA assay. Samples were then prepared in LDS buffer containing 5% 1 M DTT and subsequently loaded onto NuPAGE 4–12% Bis-Tris Midi gels, followed by the transfer of the proteins onto nitrocellulose membranes. The membranes were blocked for 1 h prior to incubation with the primary antibodies using 5% Milk TBST. Membranes were incubated at 4 °C in either BRD4**(Ab128874)**, BRD3**(Ab50818)**, BRD2**(Ab139690)**, VHL(**CST#68547**) and CRBN(**CST#71810**) primary antibody overnight. Following overnight incubation, the membranes were incubated with complementary IRDye 800CW secondary antibody and a hFAB Rhodamine Anti-Tubulin Primary Antibody (loading control) for 1 h and then imaged with a Bio-Rad imager. All Western blots were analyzed for band intensities using Image Lab (Bio-Rad). The data extracted from these blots were then plotted and analyzed using Prism (v. 10.2.2, GraphPad). Linear regression curve fitting was used to calculate pDC50 values. The standard deviation of the pDC50 was calculated for all compounds that had two independent repeats and the standard error of the mean calculated for all compounds that had three independent repeats. All Western blotting figures were developed in Adobe illustrator.

##### 4.10.5.4 Live Cell Ternary Complex Formation Assay

NanoBRET ternary complex assays were performed using Promega kits for CRBN (ND2720) and VHL (ND2700) according to manufacturer’s instructions. One day prior to the assay, HEK293 HiBiT-BRD4 (LgBiT Stable) were plated at a density of 800,000 cells/well in a 6-well plate. Cells were allowed to adhere for at least 4 h. Transfection mixtures containing 100 μL Opti-MEM, 6 μL FuGENE HD, and either 2 μg HaloTag-CRBN or HaloTag-VHL were prepared and allowed to incubate for 10 min at room temperature prior to addition to the cells. The next day, cells were trypsinized and resuspended in phenol red-free Opti-MEM Reduced Serum Medium supplemented with 4% FBS. Cells were counted and density was adjusted to 200,000 cells/mL. Cells were divided into two pools: one which received HaloTag NanoBRET 618 Ligand at a final concentration of 100 nM and one which received the same volume of DMSO. Cells were plated on white tissue-culture 96-well plates (100 μL/well) and allowed to adhere overnight. The next day, media was aspirated from the cells and replaced with phenol red-free Opti-MEM Reduced Serum medium supplemented with 4% FBS and a 1:100 dilution of Nano-Glo Vivazine substrate (Promega) and 10 μM MG132. Luminescence was allowed to equilibrate at 37 °C, 5% CO2 for 1 h. During this time, a dilution series of each PROTAC at 10x the desired final concentration was prepared (keeping DMSO constant in all concentrations). 10 μL of this dilution series was added to the plate after luminescence equilibration, and kinetic measurements of donor emission at 460 nm and acceptor emission at 618 nm were collected every 3 min using a ClarioStar plate reader. milliBRET ratios were calculated as (Emission at 618 nm/Emission at 460 nm) × 1000. Donor-contributed background or bleedthrough was corrected for by subtracting milliBRET ratios from no ligand control cells from treated cells. Corrected milliBRET ratios were plotted as a function of time in GraphPad Prism.

##### 4.10.5.5 NanoBRET Live vs Lytic Target Engagement

CRBN (N2910, Promega) and VHL (N2930, Promega) NanoBRET target engagement assays were performed according to manufacturer’s instructions. Transfection complexes were prepared using 1 mL Opti-MEM, 30 μL FuGENE HD, and either 9 μg/mL DDB1 Expression Vector and 1 μg/mL NanoLuc-CRBN fusion vector, or 9 μg/mL Transfection Carrier DNA and 1 μg/mL VHL-NanoLuc fusion vector. Transfection complexes were allowed to form for 20 min at room temperature, then were added to 20 mL of HEK293 cells at density of 200,000 cells/mL. Transfected HEK293 cells were plated in a T75 flask and allowed to express protein overnight. The next day, 85 μL of transfected cells (at 200,000 cells/mL) were replated in white nonbinding 96-well plates for live and lytic target engagement measurements. For live mode, a 100x solution of NanoBRET tracer was prepared using 100% DMSO (50 μM for CRBN live-cell, 100 μM for VHL live-cell). 100x tracer was used to prepare 20× tracer using tracer dilution buffer. PROTACs or test compounds were prepared at 10x final concentrations in Opti-MEM. Five μL of the prepared tracer was then dispensed to each well and the plate was mixed on an orbital shaker at 300 rpm for 15 s. Ten μL of each 10x PROTAC dilution was added to the cells and the plate was mixed again before being incubated at a 37 °C, 5% CO2 incubator for 2 or 5 h (as indicated in each figure). After incubation, a 50 μL of a 3X substrate solution (containing 30 μL NanoBRET Nano-Glo Substrate, 10 μL Extracellular NanoLuc inhibitor, and 4960 μL of Opti-MEM) was added to each well. Plates were incubated for 2–3 min at room temperature, then donor emission (450 nm) and acceptor emission (at 610 nm) values were read using a GloMax Discover. For lytic mode, the following changes were made. 100x tracer concentration for CRBN was 13 μM, 100X tracer concentration for VHL was 25 μM, 75 μL of a 200,000 cell/mL solution was plated in white nonbinding 96-well plates, and 10 μL of a 10x digitonin solution was added immediately after the cells were treated with PROTACs. Plates were incubated in darkness for 5 min after digitonin addition to allow for permeabilization. 50 μL of 3X substrate (30 μL NanoBRET Nano-Glo substrate and 4970 μL Opti-MEM) was added to each well and plates were incubated for 1 min at room temperature before being read on a GloMax Discover as above.

##### 4.10.5.6 Generation of CRBN and VHL Knockout in CRISPR Knock-in HiBiT-BRD2/3/4 HEK293 Cells

HiBiT-BRD4 (LgBiT stable) HEK293 cells (Promega) were used to generate both VHL KO HiBiT-BRD4 (LgBiT stable) and CRBN KO HiBiT-BRD4 (LgBiT stable) cell lines. For VHL, gRNAs targeting exon 1 (TCGAAGTTGAGCCATACGGG) and exon 2 (TCTCTCAATGTTGACGGACA) and for CRBN, gRNAs targeting exon 3 (CTCAAGAAGTCAGTATGGTG) and exon 6 (TATAAGGAATACAGCCAGCG) were purchased from IDT DNA (Table 5). For each gRNA, tracrRNA/crRNA duplexes were formed by combining 10 μL of 100 μM Alt-R tracrDNA (IDT), 100 μM Alt-R crRNA (IDT) and heating at 95C for 5 min, then were allowed to cool to room temp for 20 min. RNPs were then formed by combining 75 pmol Cas9 (IDT) with 2.4 μL total of prepared tracrRNA/crRNA complex (1.2 μL for each gRNA targeting each exon). Cells were then trypsinized and resuspended at 10,000,000 cells/mL in 1 mL of Mirus Ingenio electroporation solution. Three μL of 100 μM electroporation enhancer (Mirus) was added, along with 3 μL of RNP solution. Cells were electroporated in 2 mm cuvettes using a BioRad system (190 V, 950 μF, infinite resistance) and were then plated in a T75 flask in full growth medium to allow for recovery. After recovery from electroporation, knockout pools were sorted for single cells in 96-well tissue culture treated plates. Clonal populations which expanded from single cells were then screened for loss of function of VHL by treating with MZ1, or loss of function of CRBN by treating with dBET6, using a HiBiT-BRD4 luminescent readout assay. Genomic DNA isolated from candidate clones was then PCR-amplified (Table of primers below), Gibson-assembled into a pF1A plasmid backbone, transformed into *E. coli*., and then Sanger sequenced to confirm presence of indels in the targeted exons. CRBN exon 3 was found to carry a homozygous 5-nucleotide deletion 323bp downstream from the start codon, resulting at a premature stop codon in exon 3. CRBN exon 6 did not have any mutations. VHL exon 1 was found to have a 29bp deletion 270bp downstream from the start codon on one allele, and a 14bp deletion 265bp downstream from the start codon on the other allele. VHL exon 3 carried a 19 bp deletion on one allele, and a 1 bp insertion on the other allele, both occurring after the premature stop codon in exon 1.

**Table 5:** VHL and CRBN gRNA Sequences

CRBN Exon 3 F	TTAGTAAGGAGCGATCGCCCACTGTGCCCGGCCTGTA
CRBN Exon 3 R	GCCTGCAGGTCGACT CTCACATTCTTACCCAACCTCTCC
CRBN Exon 6 F	TTAGTAAGGAGCGATCGCCACGTCATGGGATTATCTACAAAA
CRBN Exon 6 R	GCCTGCAGGTCGACTAAGGCACTAGAAACTGGAAAAACT
VHL Exon 1 F	TTAGTAAGGAGCGATCGCAAGAGTACGGCCCTGAAGAAGAC
VHL Exon 1 R	GCCTGCAGGTCGACTCTCAGTTCCCCGTCTGCAAAATG
VHL Exon 2 F	TTAGTAAGGAGCGATCGCGTGGCTCTTTAACAACCTTTGCTT
VHL Exon 2 R	GCCTGCAGGTCGACTCAGGCAAAAATTGAGAACTGGGC

##### 4.10.5.7 NanoBRET Ubiquitination Assays

NanoBRET ubiquitination experiments were conducted using Promega kit ND2690. HEK293 HiBiT-BRD4 parental, VHL KO, or CRBN KO cells (all stably expressing LgBiT) were plated in 6-well plates at 800,000 cells/well and allowed to adhere for 4–6 h prior to being transfected with 2 μg of HaloTag-Ubiquitin Fusion Vector. Cells were allowed to express overnight, then were trypsinized and resuspended in Opti-MEM (reduced serum, no phenol red) 4% FBS at a concentration of 220,000 cells/mL. HaloTag NanoBRET 618 ligand was added at a final concentration of 100 nM to cells (with a portion of cells retained without ligand for use as no-acceptor controls for normalization). 90 μL of cells were then dispensed into each well of a white tissue culture 96-well plate and cells were allowed to adhere overnight. The next day, media was aspirated from cells and replaced with 90 μL of a 1× solution of Opti-MEM 4% FBS containing a 1:100 dilution of Nano-Glo Vivazine substrate. Cells were incubated for 60 min at 37 °C (5% CO2) and a dilution series of each PROTAC were prepared at 10x the desired final concentration (keeping the amount of DMSO in each concentration constant). 10 μL of each 10 × PROTAC was added to each well and the plate was immediately placed in a ClarioStar plate reader (prewarmed to 37 °C) where donor emission (460 nm) and acceptor emission (618 nm) was collected every 3 min for 4 h. NanoBRET ratios were calculated as described in the ternary complex assay.

##### 4.10.5.8 Mass Spectrometry Proteomics. S-Trap Processing for Quantitative Proteomics

S-Trap micro spin column digestion was performed according to the manufacturer’s protocol. Briefly, HEK293 cells were treated with DMSO, 250 nM *neg-cis*-AB3067 (**95**) or 250 nM AB3067 (**27**) for 4 h; cells were harvested and washed with PBS and spun. Cell pellets were solubilized in 5% SDS, 50 mM triethylammonium bicarbonate, reduced with 100 mM DTT solution and alkylated with iodoacetamide to a final concentration of 40 mM. Aqueous phosphoric acid was added to a final concentration of 1.2%. Protein particulate was formed by adding S-Trap binding buffer [90% aqueous methanol, 100 mM TEAB (pH 7.1)]. The mixture was placed on S-Trap micro 1.7 mL columns and centrifuged at 4000 × *g* for 10 s. Columns were washed with 150 μL of S-Trap binding buffer five times and centrifuged at 4000 × *g* for 10 s. Samples were digested with 2 μg of trypsin (Promega) at 37 °C for four h. Peptides were eluted with 40 μL of 50 mM TEAB followed by 40 μL of 0.2% aqueous formic acid, and peptides were finally vacuum-dried. TMT15plex labeling of DMSO, 250 nM *neg-cis*-AB3067 (**95**) or 250 nM AB3067 (**27**) conditions were performed, with five biological replicates for each condition and peptide cleanup was performed according to the manufacturer’s protocol. After checking the labeling efficiency, samples were combined, desalted, and dried under vacuum. TMT samples were fractionated using offline high-pH reverse-phase chromatography. Peptides were separated, concatenated to 22 fractions, dried, and peptides redissolved in 5% formic acid and analyzed by LC–MS.

##### 4.10.5.9 Proteomics Quantification and Bioinformatics Analysis

The raw mass spectrometric data were loaded into MaxQuant. Enzyme specificity was set to that of trypsin/P, allowing for cleavage of N-terminal to proline residues and between aspartic acid and proline residues. Other parameters used were as follows: (i) variable modifications—methionine oxidation, protein N-acetylation; (ii) fixed modifications, cysteine carbamidomethylation; (iii) database: Uniprot; -Human (iv) labels: 15-plex TMT (v) MS/MS tolerance: FTMS- 20 ppm, (vi) minimum peptide length, 7; (vii) maximum missed cleavages, 2; and (viii) and (ix) PSM and Protein false discovery rate, 1%. Reporter ion intensities (corrected) results from MaxQuant were imported into excel for bioinformatic analysis. The normalized corrected reporter ion intensities for each label were used to calculate ratios, and all “Contaminant,″ “Reverse”, and “Only identified by site” proteins were removed from the data. Proteins above or below twofold change [log_2_(2) = 1], and a nominal p-value less than 0.03 [−log_10_(0.03) = 1.5] were considered as differentially expressed proteins. The final volcano plot was produced in GraphPad prism 10 version [10.2.2].

##### 4.10.5.10 BromoTag Heterotrivalent PROTAC 97 in HEK293 Cells

**Cell Culture** HEK293 cells were obtained from ATCC. HEK293 was cultured in DMEM supplemented with 10% (v/v) fetal bovine serum (FBS), 1% l-glutamate and 1% (v/v) penicillin/streptomycin (pen/strep) at 37 °C, 5% CO_2_, and 95% humidity.

##### 4.10.5.11 Development of eGFP-IRES-HiBiT-BromoTag-BRD4 HEK293 Cell Line

To perform the single gRNA Cas9 CRISPR KI of BromoTag into BRD4 in HEK293. HEK293 cells were plated at a density of 5 × 10^5^ cells into individual wells of a six-well plate in 1 mL of DMEM and left overnight to adhere to the plate. The cells were subsequently transfected the following day using GeneJuice lipofectamine reagent simultaneously with a custom donor vector pMK-RQ containing 500bp BRD4 homology arms on either side of an eGFP-IRES-HiBiT-BromoTag and two individual pBABED vector harboring U6-sgRNA and puromycin expression cassettes containing two BRD4 targeting gRNA sequences: GTGGGATCACTAGCATGTCTG and GACTAGCATGTCTGCGGAGAG. The construction of these plasmids was performed by Thomas Macartney of the MRC-PPU CRISPR services. The following day cells were washed with PBS before fresh DMEM media was applied cells were left to recover in DMEM for a further 4 days. Cells were subsequently FACS sorted for GFP expression and expanded from single cells. HEK293 clones were validated for eGFP-IRES-HiBIT-BromoTag-BRD4 integration via WB, junction PCR and sequencing.

##### 4.10.5.12 Dose–Response Degradation Assay

Dose–response degradation assay **97** of was performed on the genotype verified eGFP-IRES-HiBIT-BromoTag-BRD4 homozygous HEK293 cell lines. The cells were plated at a density of 5 × 10^5^ cells per well of a six well plate a day prior to initiation of the experiment. **97** was dissolved into a 10 mM concentrated stock solution in DMSO from which **97** was further diluted to a working concentration range of 10 μM to 10 pM in DMEM and was subsequently added to cells. Additional controls including, vehicle, AGB1 (1 μM) and *cis*-AGB1 (1 μM) were similarly added to cells alongside **97** treatments. Cells were left to incubate with compound for 4 h at 37 °C, 5% CO_2_, and 95% humidity. Cells were then subsequently washed twice with PBS before being harvested in RIPA buffer supplemented with protease inhibitor cocktail and Benzonase Nuclease before being store at −20 °C.

##### 4.10.5.13 Western Blotting Analysis of Dose–Response Degradation Assay

Total protein quantity was determined using the BCA protein assay. Protein concentration was determined using the BCA assay. Samples were then prepared in LDS buffer and subsequently loaded onto NuPAGE 4–12% Bis-Tris Midi gels, followed by the transfer of the proteins onto nitrocellulose membranes. The membranes were blocked for 1 h prior to incubation with the primary antibodies using 5% Milk TBST. Membranes were incubated at 4 °C in either BRD4(**Ab128874**), BRD3(**Ab50818**) or BRD2(**Ab139690**) primary antibody overnight. Following overnight incubation, the membranes were incubated with complementary IRDye 800CW secondary antibodies and a hFAB Rhodamine Anti-Tubulin Primary Antibody (loading control) for 1 h and then imaged with a Bio-Rad imager. All Western blots were analyzed for band intensities using Image Lab (Bio-Rad). The data extracted from these blots were then plotted and analyzed using Prism (v. 10.2.2, GraphPad). All Western blotting figures were developed in Adobe Illustrator.

## Data Availability

The mass spectrometry raw data and MaxQuant search output tables has been deposited in Proteo-meXchange, PRIDE database: https://www.ebi.ac.uk/pride/archive/.

## Supporting Information Available

Quantification of EC_50_ values 95% CI from cellular proliferation and viability assays in RKO, KBM7 and K562 cells; raw kinetic traces for HiBiT-BET degradation of heterotrivalent (**23–32**) and heterotetravalent (86) PROTACs; quantification of *D*_max 50_ and λ_max_ values with 95% CI from live cell HiBiT-BET degradation assay; uncropped Western blots for BET, VHL and CRBN degradation by compounds **23–32**, **86** and **97** in HEK293 cells; live cell HiBiT-BET degradation for control compounds **93–95**; Western blots and quantification for BromoTag-BRD4 degradation with **97** and AGB1 in BromoTag-BRD4 HEK294 cells; HPLC and HRMS traces for compounds **1, 2, 23–32, 86, 93–95** and **97** (PDF)
