# Supplementary material for: Leveraging Dual-Ligase Recruitment to Enhance Protein Degradation via a Heterotrivalent Proteolysis Targeting Chimera
Source: J Am Chem Soc. 2024 Nov 28;146(49):33675–711. doi: 10.1021/jacs.4c11556 (PMC11638965; doi:10.1021/jacs.4c11556)

Supplementary information for:

Leveraging dual-ligase recruitment to enhance protein degradation via a heterotrivalent proteolysis targeting chimera

Authors: Adam G. Bond,[‡,1] Miquel Muñoz i Ordoño,[‡,2] Celia M. Bisbach,[‡,3] Conner Craigon,[1] Nikolai Makukhin,[1] Elizabeth A. Caine,[3] Manjula Nagala,[1] Marjeta Urh,[3] Georg E. Winter,[*,2] Kristin M. Riching,[*,3] Alessio Ciulli[*,1]

Authors affiliations:

[1] Centre for Targeted Protein Degradation, School of Life Sciences, University of Dundee, 1 James Lindsay Place, DD1 5JJ, Dundee, United Kingdom

[2] CeMM Research Center for Molecular Medicine of the Austrian Academy of Sciences, 1090 Vienna, Austria

[3] Promega Corporation, 2800 Woods Hollow Road, Madison, WI U.S.A 53711.

*Co-corresponding authors. E-mails: GWinter@cemm.oeaw.ac.at ; kristin.riching@promega.com ; a.ciulli@dundee.ac.uk

‡ A.G.B., M.M.O. and C.M.B. contributed equally as co-first author

# Present addresses:

A.G.B and N.M.: Kesmalea Therapeutics LTD, ARC West London, Manbre Wharf, Manbre Road, London, W6 9RH, UK. C.M.B.: Waisman Center, 1500 Highland Ave, Madison, WI 53705, USA

Table of Contents

Supplementary Figure 1. Raw kinetic traces of first generation heterotrivalent PROTACs in HiBiT-BET in HEK293 cells……………………………………………………………………….**S5**

Supplementary Table 1. Quantification of EC_{50} values from cellular proliferation assays with dBET6, MZ1 and heterotrivalent PROTACs **1** and **2** in WT, CRBN KO, VHL KO and CRBN/VHL dKO RKO cell lines……………………………………………………………..**S5**

Supplementary Figure 2. Raw kinetic traces of second generation heterotrivalent PROTACs in HiBiT-BET in HEK293 cells……………………………………………………………………….**S6**

Supplementary Table 2. Quantification of live-cell degradation parameter $D_{max\ 50}$ with heterotrivalent PROTACs **1**, **2** and **23** – **32** against BET proteins in HiBiT-BRD knock-in HEK293 cells……………………………………………………………………………………………**S7**

Supplementary Table 3. Quantification of live-cell degradation parameter degradation rate (λ_{max}) with heterotrivalent PROTACs **1**, **2** and **23** – **32** against BET proteins in HiBiT-BRD knock-in HEK293 cells………………………………………………………………………………….**S7**

Supplementary Figure 3. Cell viability evaluation of heterotrivalent PROTACs **23** – **32** in RKO, KBM7 & K562 cell lines…………………………………………………………………………**S8**

Supplementary Table 4. Quantification of EC_{50} values from cellular proliferation assays with dBET6, MZ1 and heterotrivalent PROTACs **2** and **23** – **32** in WT RKO, KBM7 and K562 cell lines…………………………………………………………………………………………………**S9**

Supplementary Table 5. Quantification of EC_{50} values from cellular proliferation assays with heterotrivalent PROTACs **26**, **27** and **86** in WT, CRBN KO, VHL KO and CRBN/VHL dKO RKO cell lines……………………………………………………………..………...................**S9**

Supplementary Figure 4. Kinetic traces associated with heterotetravalent PROTAC **86**………………………………………………………………………………………...**S1 0**

Supplementary Figure 5. Degradation potency and rate of control compounds in HiBiT-BRD4 parental, VHL KO, and CRBN KO cells…………………………………………………………**S10**

Supplementary Figure 6. NanoBRET lytic and live cell target engagement assay of **27** and **94**………………………………………………………………………………………….**S11**

Supplementary Table 6. Quantification of EC_{50} values from cellular proliferation assays with dBET6, MZ1 and heterotrivalent PROTACs **27** and **93** – **95** in WT, CRBN KO, VHL KO and CRBN/VHL dKO RKO cell lines……………………………………………………………….**S11**
Supplementary Figure 7. Cell viability assay with dBET6, MZ1, **27** and a 1:1 combination of dBET6 and MZ1 in BET sensitive RKO WT cells…………………………………………...**S12**
Supplementary Table 7. Quantification of EC_{50} values from cellular proliferation assays with dBET6, MZ1, **27** and 1:1 combination of dBET6 and MZ1 in BET sensitive RKO WT cells…..**S12**
Supplementary Figure 8. Uncropped Western Blotting images of the evaluation of heterotrivalent PROTACs **23** – **32** & **86** in HEK293 cells…………………………………………………...…**S13**
Supplementary Figure 9. Uncropped independent replicate western blotting images of the evaluation of heterotrivalent PROTACs **23** – **32** & **86** in HEK293 cells………………………..**S28**
Supplementary Figure 10. Uncropped independent replicate western blotting images of the evaluation of heterotrivalent **97** in homozygous BromoTag-BRD4 HEK293 cells……………..**S51**
Supplementary Figure 11. Cropped western blotting images of the evaluation of heterotrivalent AB3145 (97) and AGB1 in homozygous BromoTag-BRD4 HEK293 cells…………………….**S59**
Supplementary Table 8. DC_{50} values of AB3145 (**97**) and AGB1 in homozygous BromoTag-BRD4 HEK293 cells………………………………………………………………………**S58**
HPLC & HRMS Traces for Compounds 1, 2, 23 – 32, 86, 93 – 95 and 97...………………...**S60**

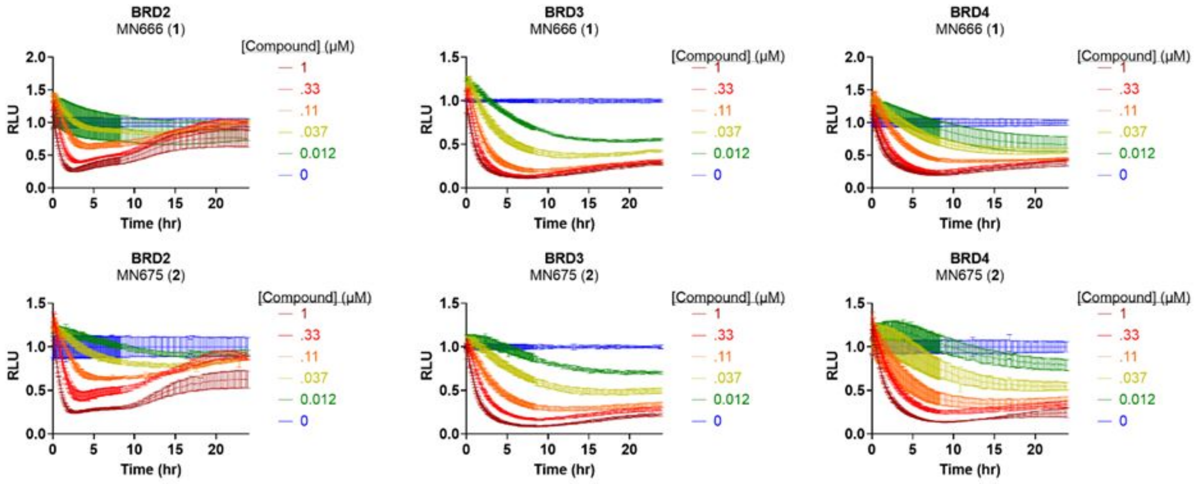

Supplementary Figure 1 (related to Figure 3): Representative kinetic traces of first generation heterotrivalent PROTACs in HiBiT-BRD2, BRD3, and BRD4 HEK293 cells. Cells were treated with a dilution series ranging from 1 µM - 12 nM compound. For all panels, each plot shows a single representative experiment with n=3 technical replicates; error bars represent S.D.

Supplementary Table 1 (related to Figure 3)**: Quantification of EC_{50} values from cellular proliferation assays with dBET6, MZ1 and heterotrivalent PROTACs 1 and 2 in WT, CRBN KO, VHL KO and CRBN/VHL dKO RKO cell lines.**

	EC_{50} (nM) [a]							
RKO line	dBET6	95% CI	MZ1	95% CI	MN666 (**1**)	95% CI	MN675 (**2**)	95% CI
WT	281	237 to 327	212	183 to 246	720	550 to 1001	489	435 to 552
CRBN KO	>10000	-	181	144 to 228	703	589 to 844	601	467 to 773
VHL KO	233	203 to 267	>10000	-	336	287 to 397	870	732 to 1079
CRBN/VHL dKO	>10000	-	>10000	-	1579	1184 to 2464	2063	1585 to 3112

[a] Data is colour scaled for lowest (green), median (yellow) and highest (red) EC_{50} values in nM.

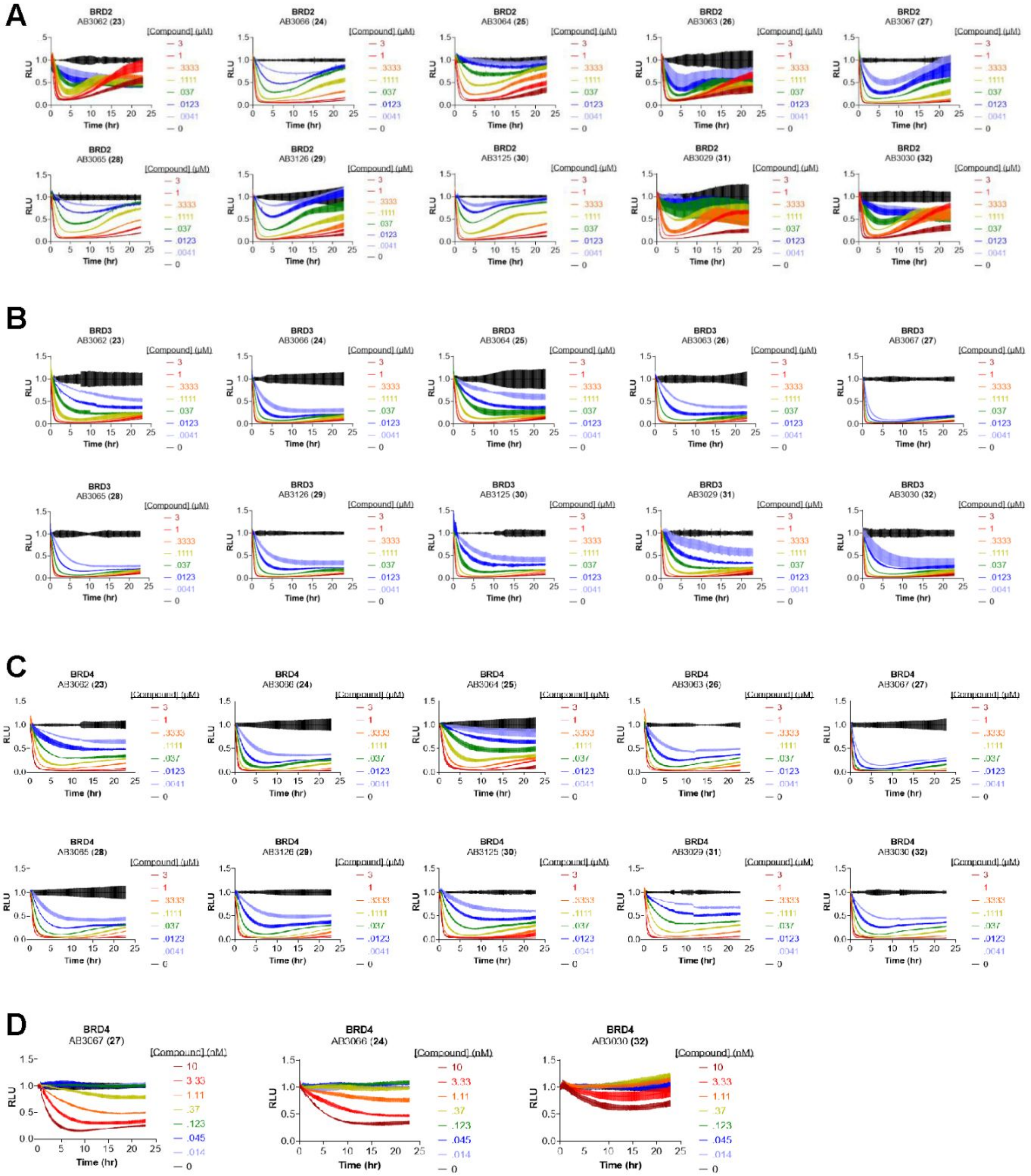

Supplementary Figure 2 (related to Figure 4): (*A-C*) Kinetic traces of second generation heterotrivalent PROTACs in HiBiT-BRD2 (*A*), BRD3 (*B*), and BRD4 (*C*) HEK293 cells. Cells were treated with a dilution series ranging from 3 µM - 4 nM compound. (*D*) Raw kinetic traces of HiBiT-BRD4 HEK293 cells treated with an extended nanomolar 3-fold dilution series ranging from 10 nM to 0.014 nM of compounds **27**, **24**, and **32**. For all panels, each plot shows a single representative experiment with n=3 technical replicates; error bars represent S.D.

Supplementary Table 2 (related to Figure 4B & Table 3)**: Quantification of live-cell degradation parameter $D_{max\ 50}$ with heterotrivalent PROTACs 1, 2 and 23 – 32 against BET proteins in HiBiT-BRD knock-in HEK293 cells.**

	$D_{max\ 50}$, nM [a]					
Compound	BRD4	95% CI	BRD3	95% CI	BRD2	95% CI
MN666 (**1**)	37	29 to 48	-	-	-	-
MN675 (**2**)	55	37 to 97	-	-	-	-
AB3062 (**23**)	11	9.5 to 12	5.7	3.8 to 8.0	16	3.8 to und
AB3066 (**24**)	3.0	2.6 to 3.5	0.12	und[b] to 1.4	7.3	5.3 to 9.2
AB3064 (**25**)	31	24 to 40	8.1	5.8 to 12	74	59 to 93
AB3029 (**31**)	15	13 to 16	6.2	4.8 to 7.9	160	61 to und[b]
AB3063 (**26**)	3.0	2.6 to 3.3	1.5	0.70 to 2.2	4.7	2.7 to 7.5
AB3067 (**27**)	0.64	0.50 to 0.80	0.085	und[b] to 0.43	2.0	0.37 to 3.5
AB3065 (**28**)	2.9	2.4 to 3.4	1.2	0.92 to 1.5	23	20 to 27
AB3126 (**29**)	4.0	3.6 to 4.5	1.9	1.2 to 2.4	18	15 to 22
AB3125 (**30**)	8.2	7.2 to 9.3	3.0	2.2 to 3.9	31	22 to 29
AB3030 (**32**)	7.4	5.6 to 10	1.7	0.47 to 2.8	25	12 to 130

[a] Data is colour scaled for lowest (green), median (yellow), and highest (red) $D_{max\ 50}$ values. [b] "und." indicates the fit of the 95% CI is undefined.

Supplementary Table 3 (related to Figure 4C & Table 3)**: Quantification of live-cell degradation parameter degradation rate (λ_{max}) with heterotrivalent PROTACs 1, 2 and 23 – 32 against BET proteins in HiBiT-BRD knock-in HEK293 cells.**

	λ_{max}, h^{-1} [a]					
Compound	BRD4	95% CI	BRD3	95% CI	BRD2	95% CI
AB3062 (**23**)	1.40	1.36 to 1.44	2.06	1.99 to 2.13	1.12	0.93 to 1.33
AB3066 (**24**)	2.16	2.09 to 2.24	2.66	2.55 to 2.78	2.04	1.88 to 2.21
AB3064 (**25**)	1.09	1.06 to 1.11	1.69	1.62 to 1.75	1.34	1.25 to 1.44
AB3029 (**31**)	1.99	1.94 to 2.04	2.36	2.23 to 2.51	1.90	1.75 to 2.06
AB3063 (**26**)	1.98	1.89 to 2.07	2.54	2.45 to 2.64	1.72	1.57 to 1.88
AB3067 (**27**)	2.68	2.56 to 2.81	3.31	3.15 to 3.47	2.37	2.21 to 2.53
AB3065 (**28**)	1.51	1.46 to 1.55	2.14	2.06 to 2.23	1.46	1.34 to 1.58
AB3126 (**29**)	1.69	1.68 to 1.79	2.62	2.54 to 2.71	2.35	2.19 to 2.52
AB3125 (**30**)	1.76	1.70 to 1.84	2.60	2.48 to 2.72	2.63	2.54 to 2.72
AB3030 (**32**)	2.19	2.10 to 2.29	2.97	2.86 to 3.09	2.04	1.90 to 2.20

[a] Data is colour scaled for highest (green), median (yellow), and lowest (red) $D_{max\ 50}$ values.

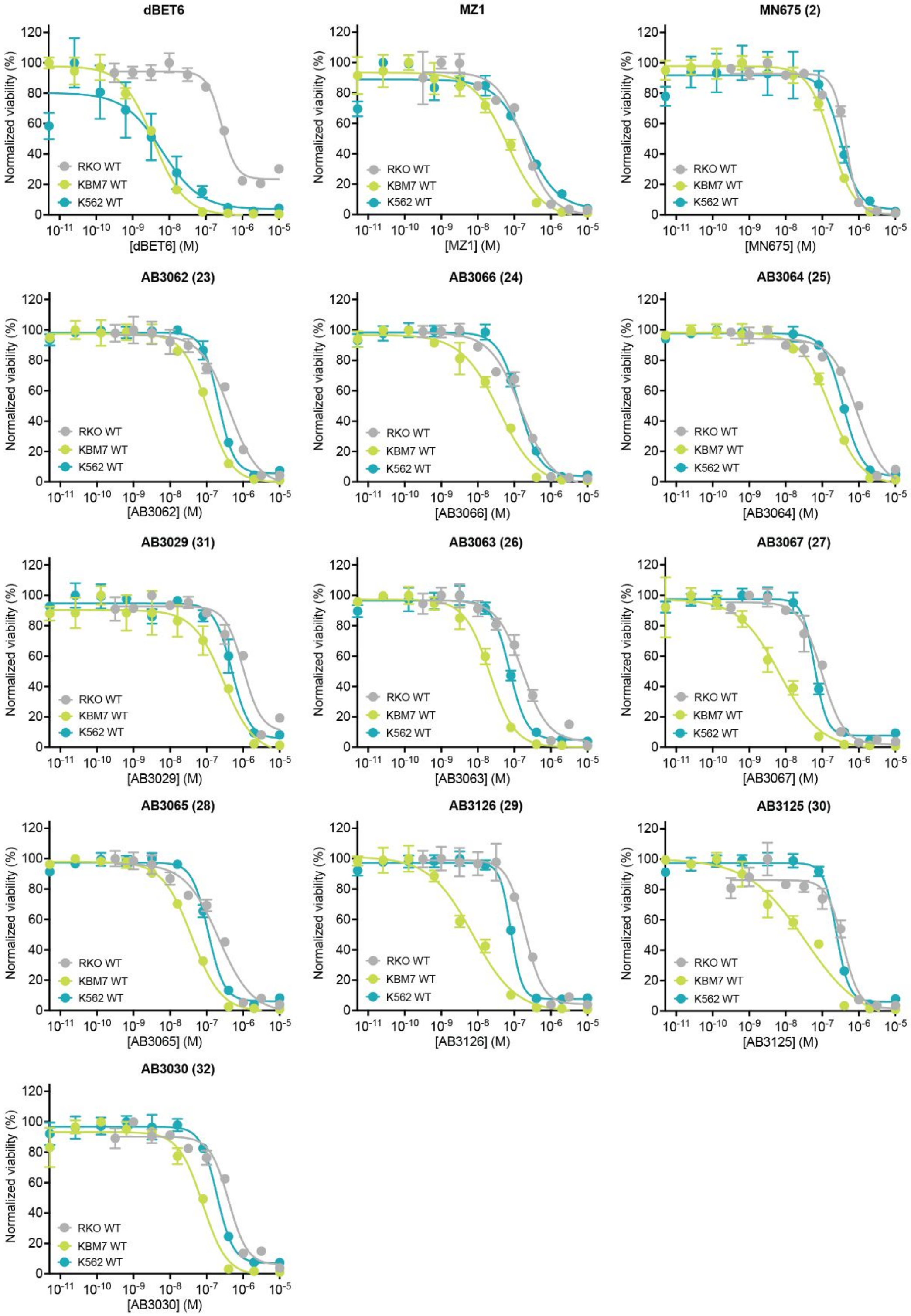

Supplementary Figure 3: **Cell viability evaluation of heterotrivalent PROTACs 23 - 32 in RKO, KBM7 & K562 cell lines.** Effect on cellular proliferation of dBET6, MZ1 and heterotrivalent PROTACS **2** and **23 - 32** after 316 pM to 10 µM treatment in WT RKO or 5.12 pM to 10 uM treatment in WT KBM7 and K562 cell lines. Mean ± S.D.; *n* = 3 biological replicates. EC_{50} values are tabulated in Supplementary Table 2.

Supplementary Table 4 (related to Supplementary Figure 2)**: Quantification of EC_{50} values from cellular proliferation assays with dBET6, MZ1 and heterotrivalent PROTACs 2 and 23 – 32 in WT RKO, KBM7 and K562 cell lines.**

Compound	EC_{50} (nM) [a]					
	RKO	95% CI	KBM7	95% CI	K562	95% CI
dBET6	271.6	223.0 to 322.6	3.7	3.0 to 4.6	6.4	2.3 to 20.1
MZ1	187.9	134.5 to 259.9	66.2	44.9 to 97.7	216.3	121.2 to 478.8
MN675 (**2**)	444.6	390.4 to 510.5	175.4	144.1 to 214.0	327.3	219.1 to 513.0
AB3062 (**23**)	419.8	319.8 to 563.7	104.2	87.7 to 124.4	212.3	187.9 to 239.3
AB3066 (**24**)	151.4	115.7 to 196.3	38.0	28.4 to 50.7	133.7	113.4 to 158.5
AB3064 (**25**)	903.6	668.5 to 1363.0	168.3	141.3 to 201.5	369.0	337.9 to 403.2
AB3029 (**31**)	1016.2	729.0 to 1332.0	294.4	185.4 to 507.9	494.3	394.1 to 648.5
AB3063 (**26**)	165.2	125.0 to 216.8	21.2	18.0 to 25.0	74.1	63.8 to 86.2
AB3067 (**27**)	97.5	78.6 to 119.4	6.9	4.3 to 10.8	62.1	53.4 to und[b]
AB3065 (**28**)	213.8	151.4 to 302.8	39.4	34.6 to 44.8	110.2	96.6 to 126.4
AB3126 (**29**)	195.4	161.5 to 236.1	7.3	5.2 to 10.2	80.5	74.9 to 87.1
AB3125 (**30**)	369.0	284.4 to 468.4	34.1	19.1 to 64.1	242.1	207.8 to 295.1
AB3030 (**32**)	401.8	311.7 to 509.8	78.0	58.3 to 102.7	192.3	158.0 to 231.7

[a] Data is colour scaled for lowest (green), median (yellow) and highest (red) EC_{50} values in nM. [b] "und." indicates the fit of the 95% CI is undefined.

Supplementary Table 5 (related to Figure 5 and Figure 8C)**: Quantification of EC_{50} values from cellular proliferation assays with heterotrivalent PROTACs 26, 27 and 86 in WT, CRBN KO, VHL KO and CRBN/VHL dKO RKO cell lines.**

RKO line	EC_{50} (nM) [a]					
	AB3063 (**26**)	95% CI	AB3067 (**27**)	95% CI	AB3124 (**86**)	95% CI
WT	260	226 to 294	111	104 to 121	33	22 to 52
CRBN KO	408	335 to 501	180	149 to 215	34	26 to 44
VHL KO	819	651 to 1013	133	121 to 145	62	51 to 75
CRBN/VHL dKO	2235	1435 to 6027	988	695 to 1902	16	13 to 20

[a] Data is colour scaled for lowest (green), median (yellow) and highest (red) EC_{50} values in nM.

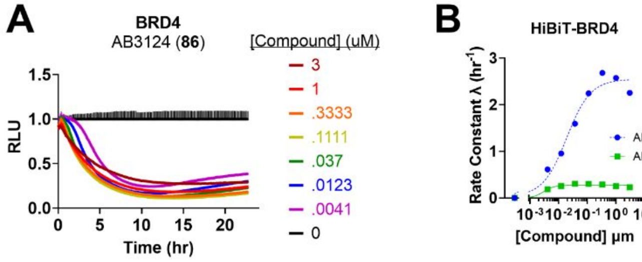

Supplementary Figure 4 (related to Figure 8): Kinetic traces associated with Figure 8. (A) 24-hour kinetic luminescence timecourse of HiBiT-BRD4 HEK293 cells supplied with an 8-point, 3-fold dilution series of **86** starting at 3 µM (n=3 technical replicates per concentration, error bars represent standard deviation). (B) BRD4 degradation rate constants mediated by **86** from the experiment in panel A of this figure) plotted alongside BRD4 degradation rate constants mediated by **27** (data replotted from Figure 4B) to allow for comparison of differences in degradation rate.

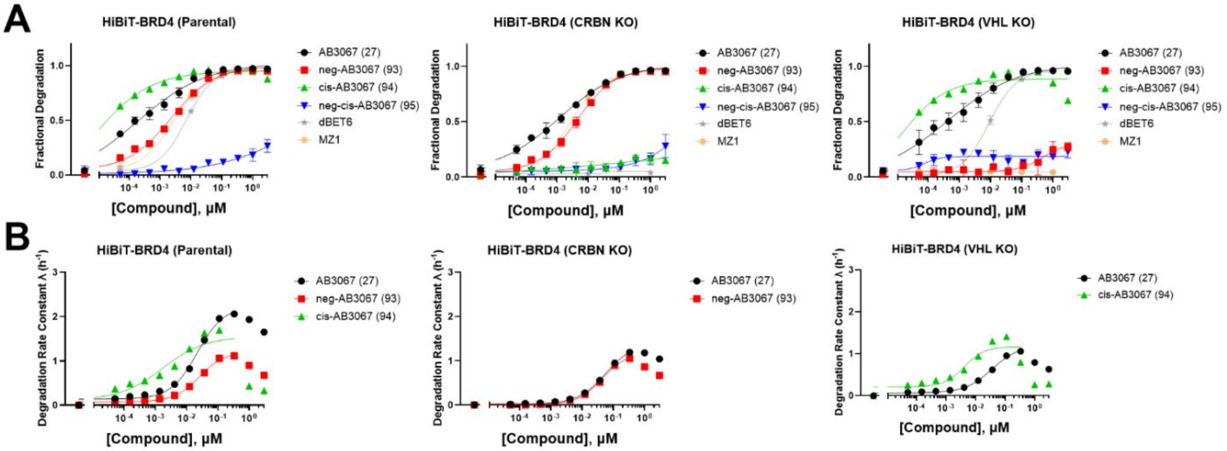

Supplementary Figure 5: **Degradation potency and rate of control compounds in HiBiT-BRD4 parental, VHL KO, and CRBN KO cells**. (A, B) Fractional degradation representing the D_{max} taken from individual kinetic profiles (A) and degradation rate constant determined from the kinetic profiles (B) of each cell line treated for 24h with an 8-point, 3-fold dilution series starting at 3 µM of **27**, **93**, **94**, or **95.** A 3-point, 10-fold dilution series starting at 1 µM of dBET6 and MZ1 were included in this experiment to show the specificity of KO of each E3 ligase.

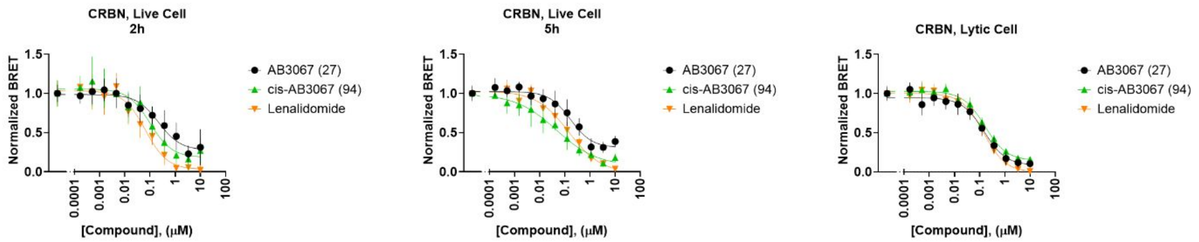

Supplementary Figure 6. NanoBRET lytic and live cell target engagement assay of 27 and 94. Competitive displacement profiles of HEK293 cells transiently transfected with NanoLuc-CRBN, which are incubated with a CRBN fluorescent tracer in the presence of serial dilutions of **27, 94** or lenalidomide in cells lysed with digitonin (Lytic cell) or in live cells for 2 h or 5 h. Data are represented as NanoBRET ratios normalized to 0 µM compound. Error bars are expressed as S.D. of the mean of n = 2 biological replicates (each consisting of 3 technical replicates) for 2 h Live Cell and Lytic and n=1 biological replicate (consisting of 4 technical replicates) for 5 h Live Cell.

Supplementary Table 6 (related to Figure 10)**: Quantification of EC_{50} values from cellular proliferation assays with dBET6, MZ1 and heterotrivalent PROTACs 27 and 93 - 95 in WT, CRBN KO, VHL KO and CRBN/VHL dKO RKO cell lines.**

	EC_{50} (nM) [a]			
RKO line	MZ1	95% CI	*neg*-AB3067 (**93**)	95% CI
WT	160	136 to 189	107	96 to 124
CRBN KO	136	118 to 158	105	94 to 117
VHL KO	>5000	-	391	324 to 504
CRBN/VHL dKO	>5000	-	420	328 to 550
RKO line	dBET6	95% CI	*cis*-AB3067 (**94**)	95% CI
WT	256	215 to 302	48	40 to 58
CRBN KO	>10000	-	832	652 to 1079
VHL KO	291	236 to 357	48	39 to 60
CRBN/VHL dKO	>10000	-	804	626 to 1051
RKO line	AB3067 (**27**)	95% CI	*neg-cis*-AB3067 (**95**)	95% CI
WT	73	66 to 81	594	460 to 786
CRBN KO	82	71 to 94	586	403 to 924
VHL KO	110	94 to 129	522	388 to 726
CRBN/VHL dKO	818	641 to 1078	657	449 to 1014

[a] Data is colour scaled for lowest (green), median (yellow) and highest (red) EC_{50} values in nM

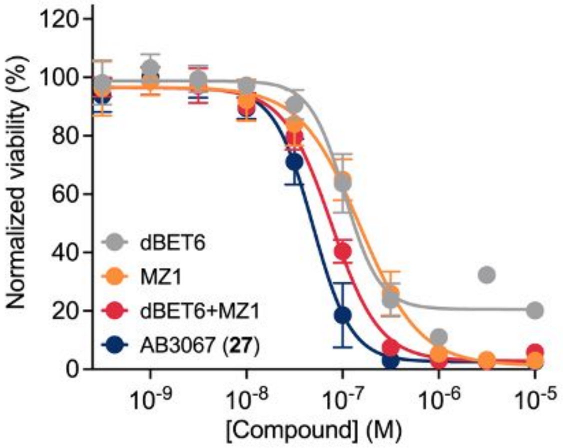

Supplementary Figure 7 (related to Figure 10)**. Cell viability assay with dBET6, MZ1, 27 and a 1:1 combination of dBET6 and MZ1 in BET sensitive RKO WT cells.** Effect on cellular proliferation of dBET6, MZ1, **27** and a 1:1 combination of dBET6 and MZ1 after 316 pM to 10 µM treatment in WT RKO cells. dBET6 and MZ1 were added at 5 µM for a total starting concentration of 10 µM BET PROTAC and diluted therein. Mean ± S.D.; n = 6 biological replicates.

Supplementary Table 7 (related to Supplementary Figure 7)**: Quantification of EC_{50} values from cellular proliferation assays with dBET6, MZ1, 27 and 1:1 combination of dBET6 and MZ1 in BET sensitive RKO WT cells.**

	EC_{50} (nM) [a]	
Compound	RKO	95% CI
dBET6	106.0	89 to 123
MZ1	152.0	132 to 175
dBET6 + MZ1	77.0	71 to 83
AB3067 (**27**)	49.0	44 to 54

[a] Data is colour scaled for lowest (green), median (yellow) and highest (red) EC_{50} values in nM.

Supplementary Figure 8 (related to Figure 4**). Uncropped Western Blotting images of the evaluation of heterotrivalent PROTACs 23 – 32 & 86 in HEK293 cells.** The boxed region represents the cropped area containing the target protein expression upon treatment with compound.

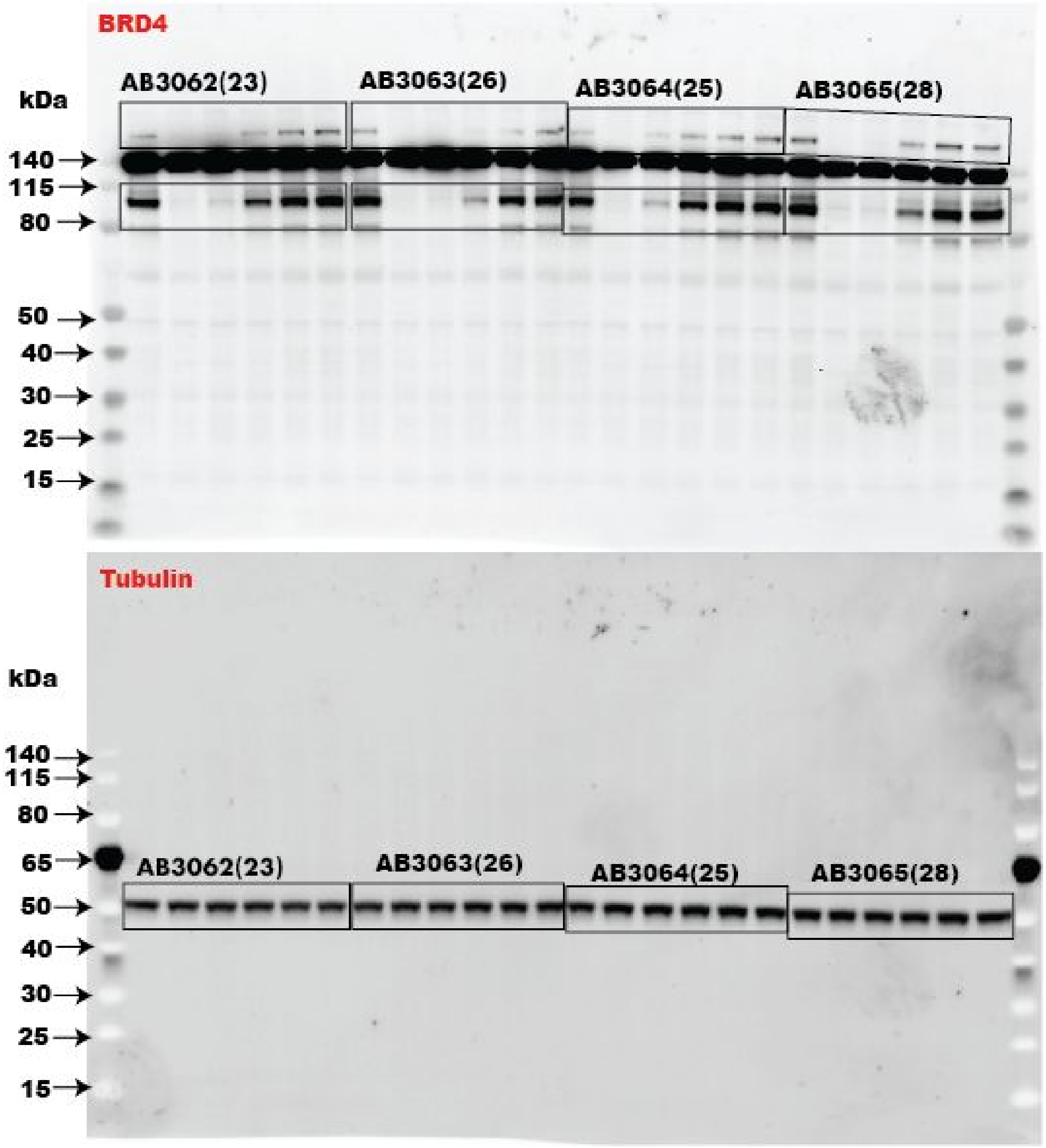

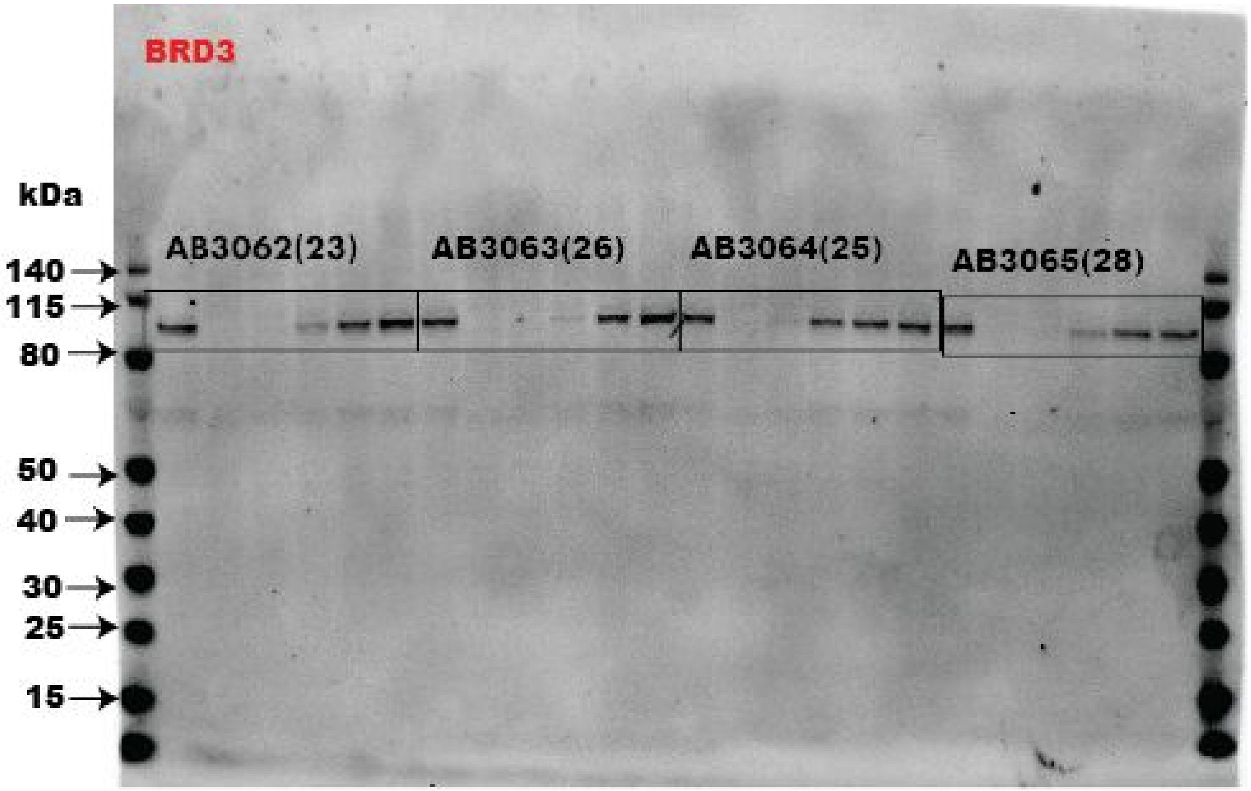
BRD3
kDa
140
115
80
50
40
30
25
15
AB3062(23)
AB3063(26)
AB3064(25)
AB3065(28)

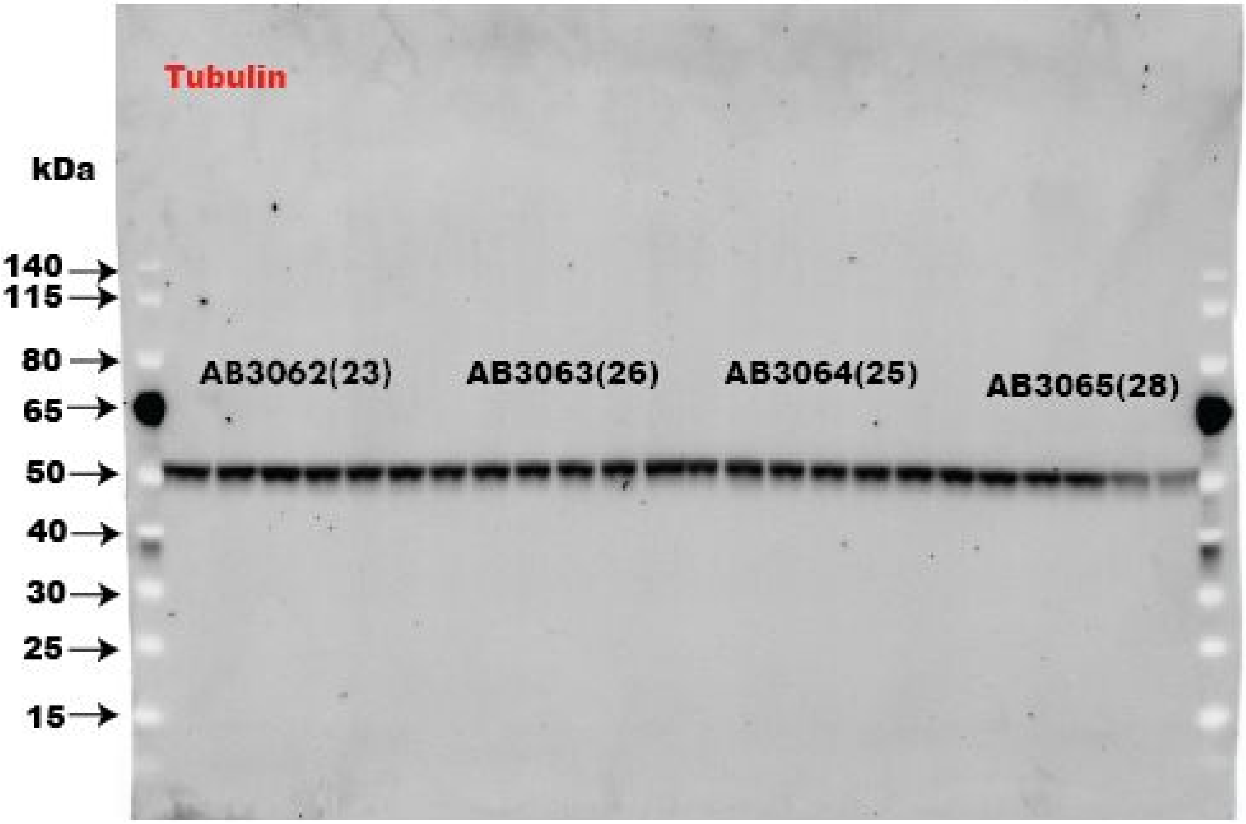
Tubulin
kDa
140
115
80
65
50
40
30
25
15
AB3062(23)
AB3063(26)
AB3064(25)
AB3065(28)

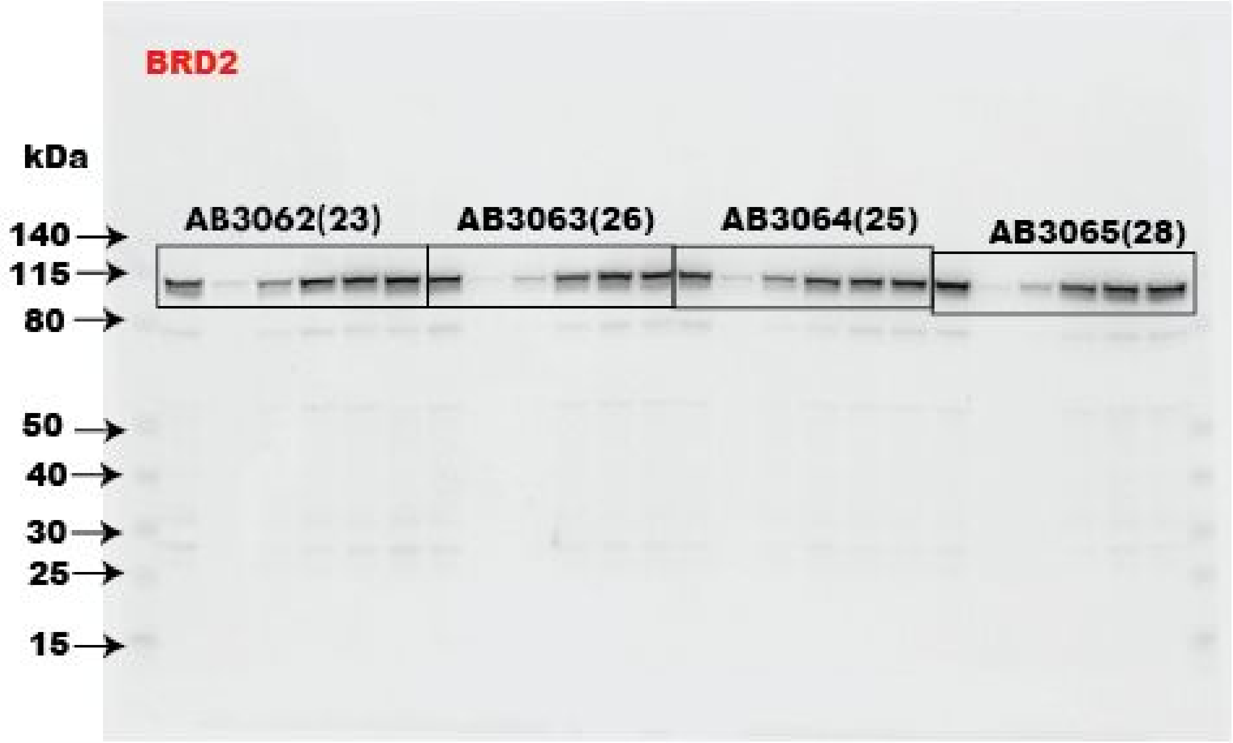

BRD2
kDa
140
115
80
50
40
30
25
15
AB3062(23)
AB3063(26)
AB3064(25)
AB3065(28)

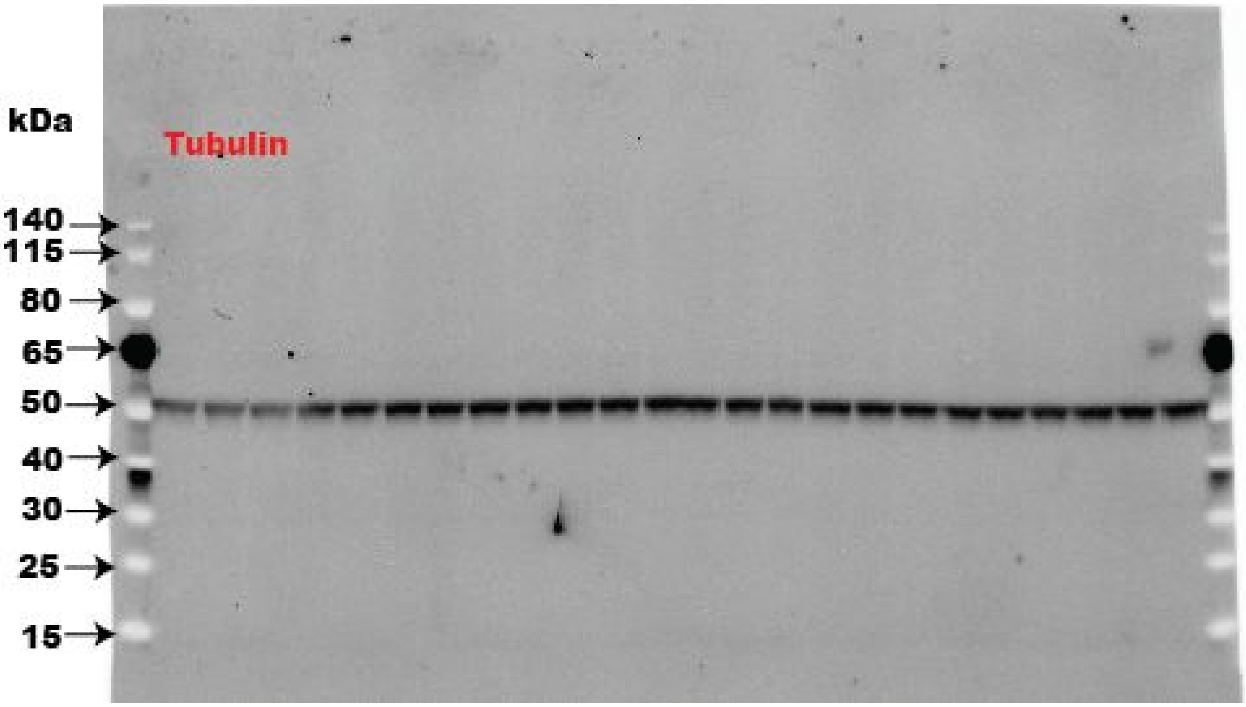

kDa
Tubulin
140
115
80
65
50
40
30
25
15

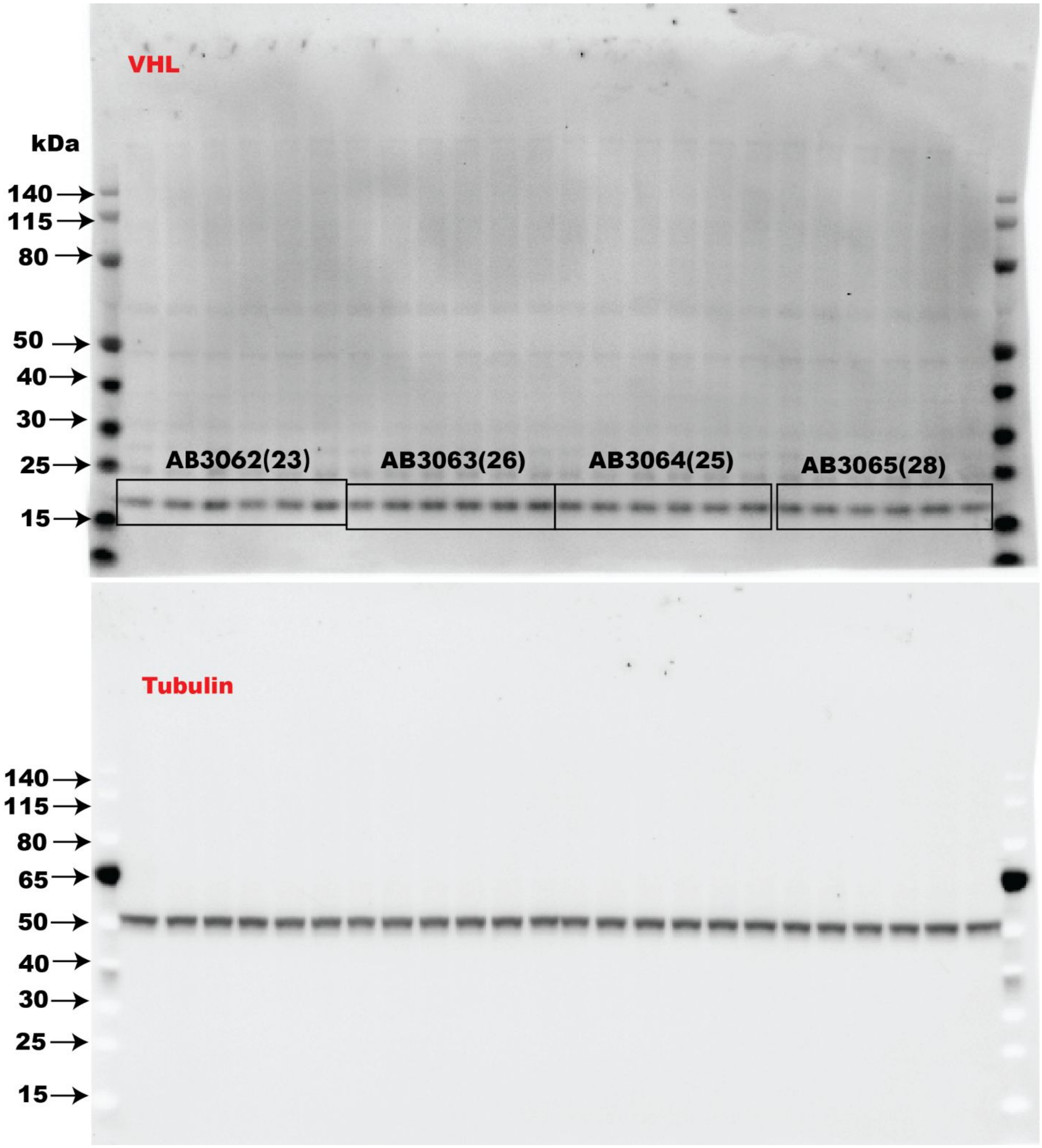
VHL
kDa
140
115
80
50
40
30
25
15
AB3062(23)
AB3063(26)
AB3064(25)
AB3065(28)
Tubulin
140
115
80
65
50
40
30
25
15

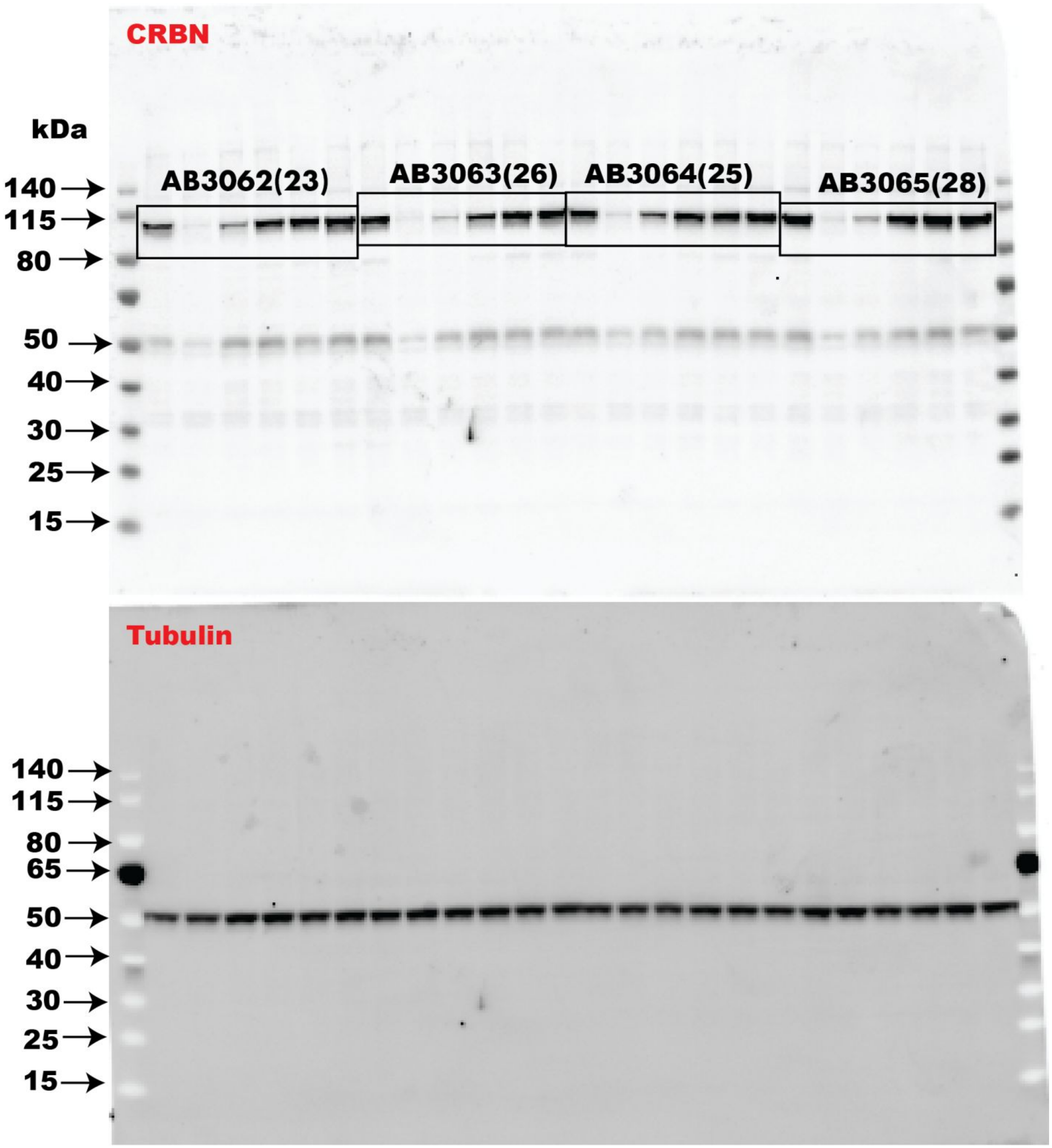
CRBN
kDa
140
115
80
50
40
30
25
15
AB3062(23)
AB3063(26)
AB3064(25)
AB3065(28)
Tubulin
140
115
80
65
50
40
30
25
15

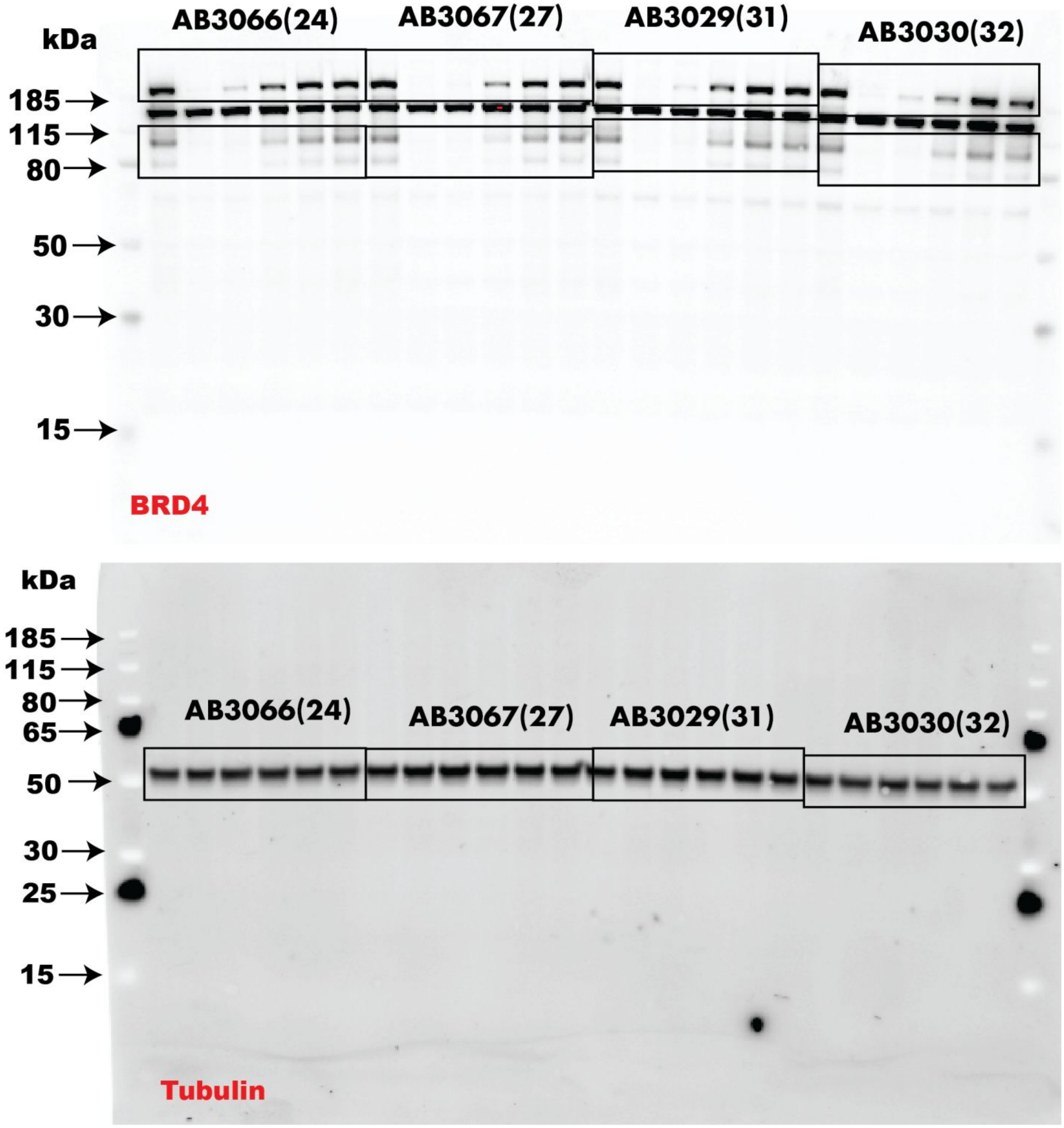
kDa
AB3066(24)
AB3067(27)
AB3029(31)
AB3030(32)
185
115
80
50
30
15
BRD4
kDa
185
115
80
65
50
30
25
15
AB3066(24)
AB3067(27)
AB3029(31)
AB3030(32)
Tubulin

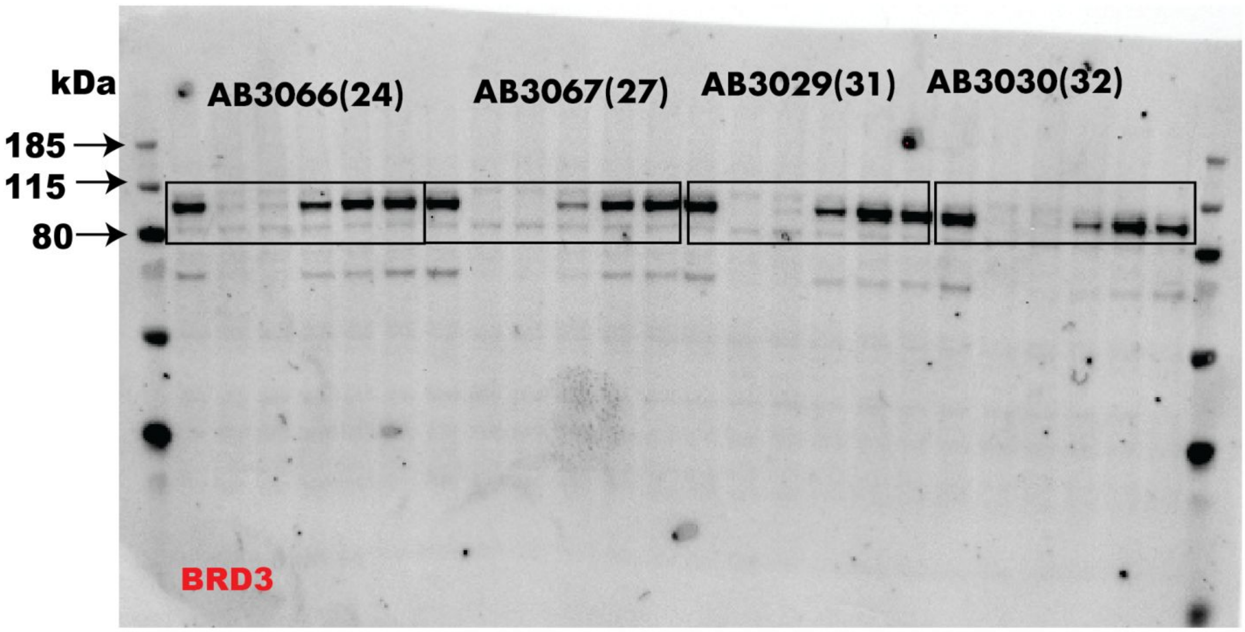
kDa
185
115
80
AB3066(24)
AB3067(27)
AB3029(31)
AB3030(32)
BRD3

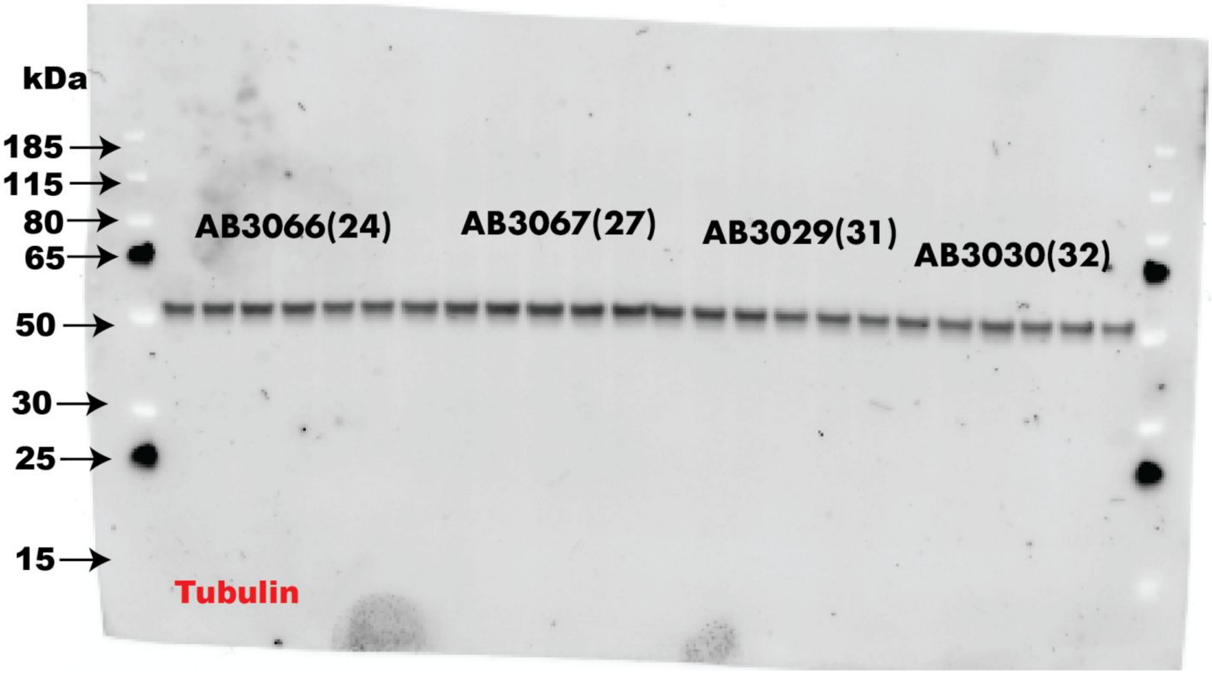
kDa
185
115
80
65
50
30
25
15
AB3066(24)
AB3067(27)
AB3029(31)
AB3030(32)
Tubulin

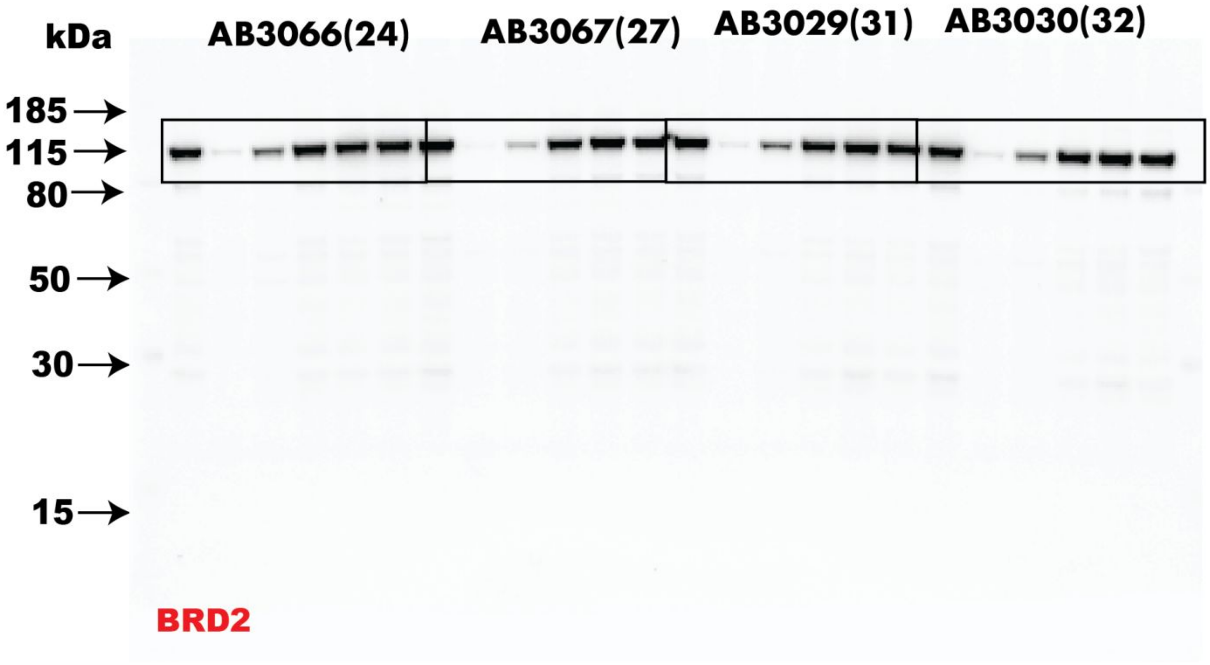
kDa
AB3066(24)
AB3067(27)
AB3029(31)
AB3030(32)
185
115
80
50
30
15
BRD2

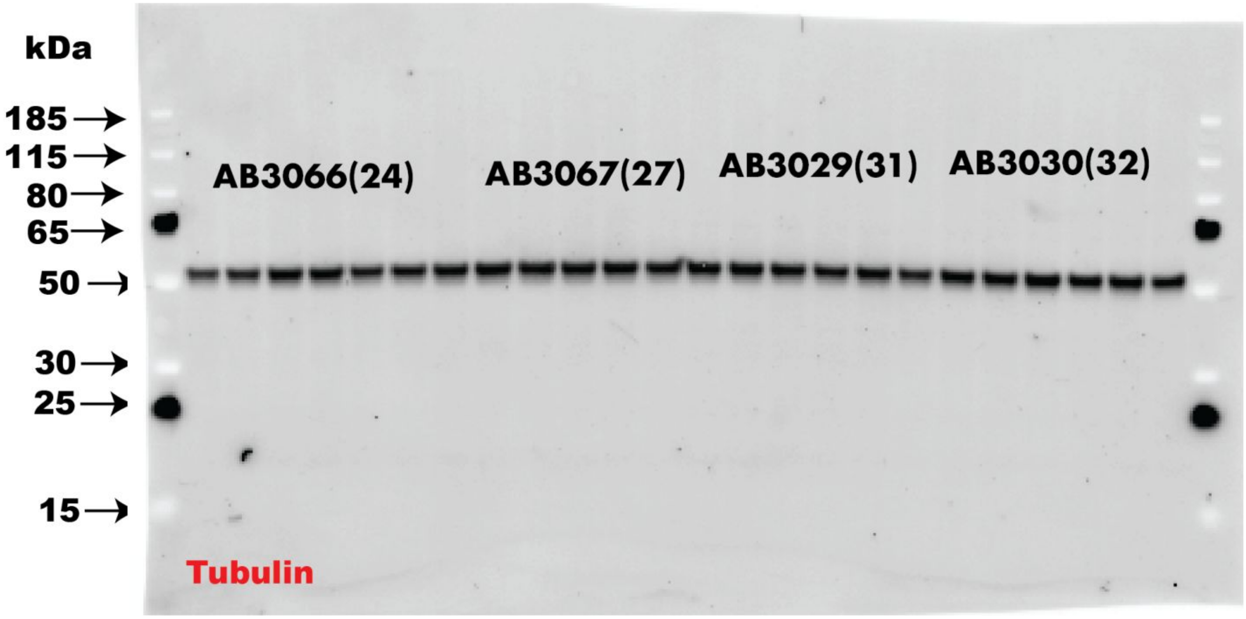
kDa
185
115
80
65
50
30
25
15
AB3066(24)
AB3067(27)
AB3029(31)
AB3030(32)
Tubulin

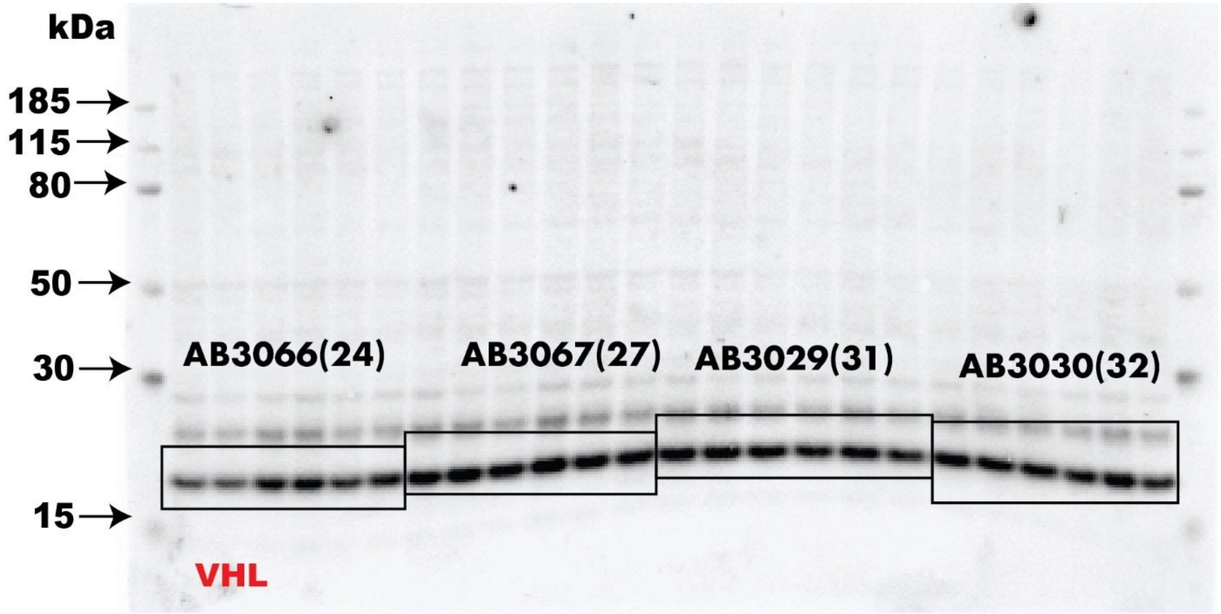
kDa
185
115
80
50
30
15
AB3066(24)
AB3067(27)
AB3029(31)
AB3030(32)
VHL

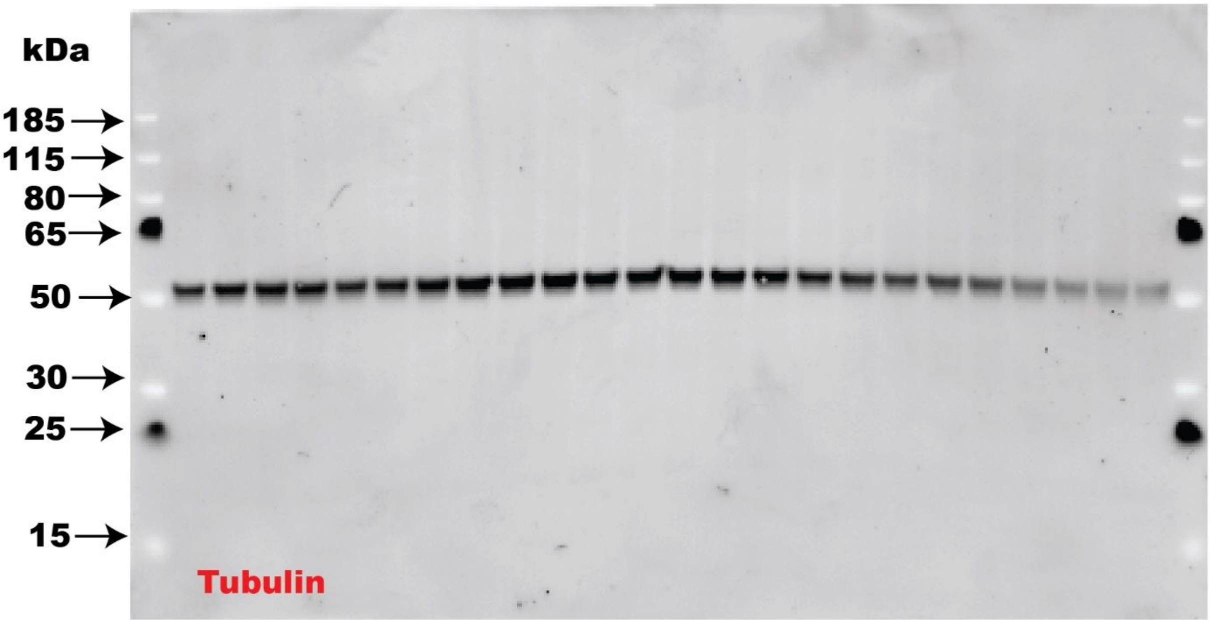
kDa
185
115
80
65
50
30
25
15
Tubulin

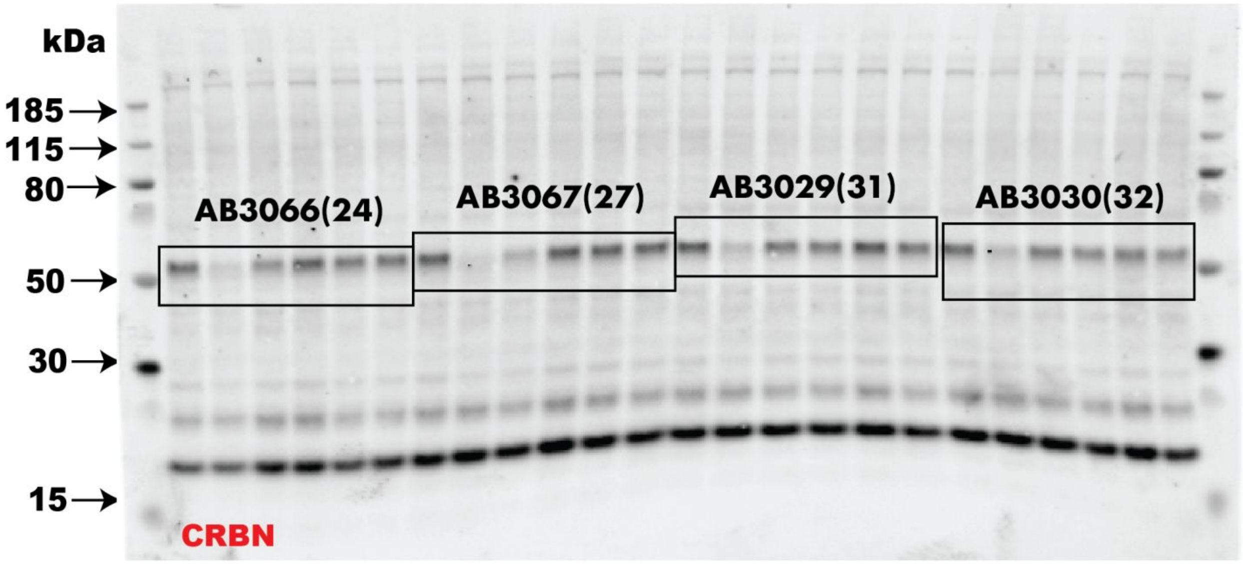
kDa
185
115
80
50
30
15
AB3066(24)
AB3067(27)
AB3029(31)
AB3030(32)
CRBN

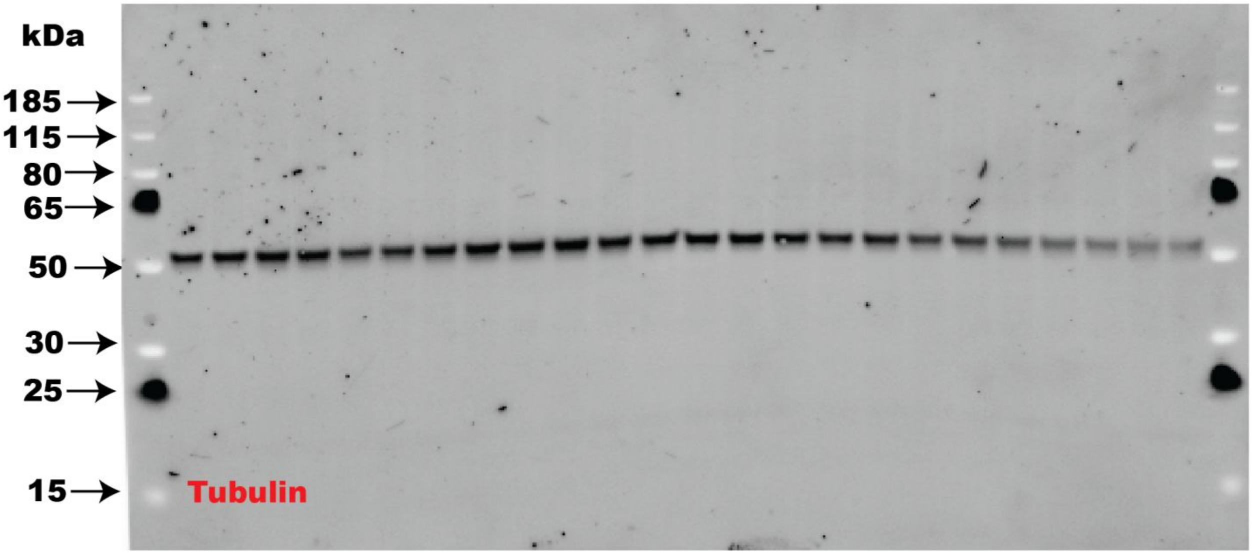
kDa
185
115
80
65
50
30
25
15
Tubulin

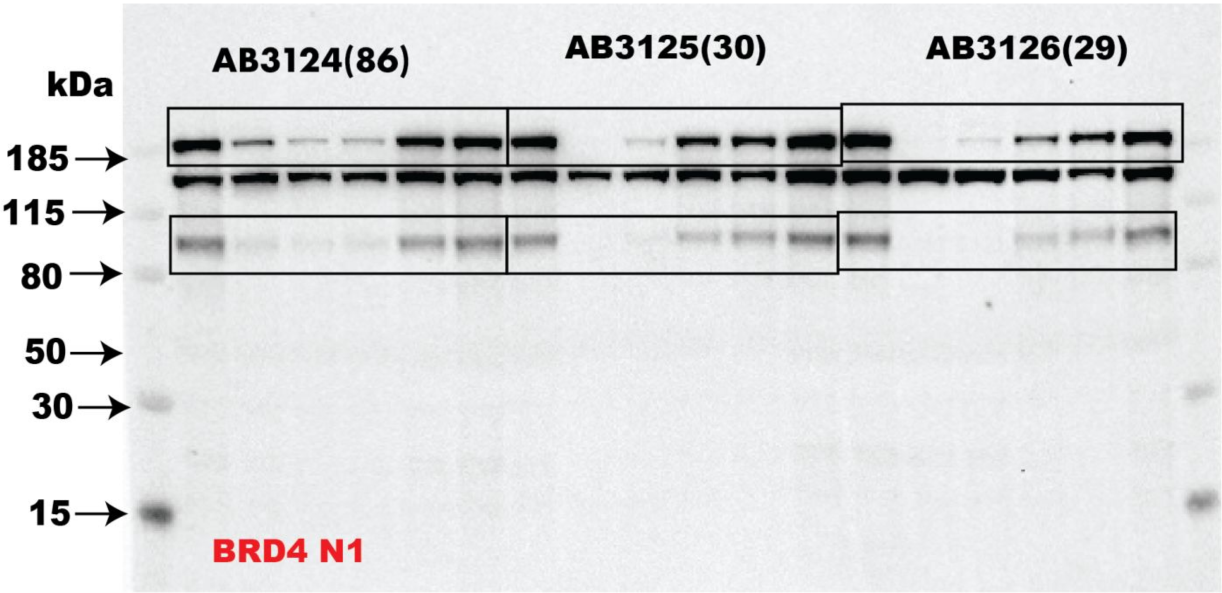
AB3124(86)
AB3125(30)
AB3126(29)
kDa
185
115
80
50
30
15
BRD4 N1

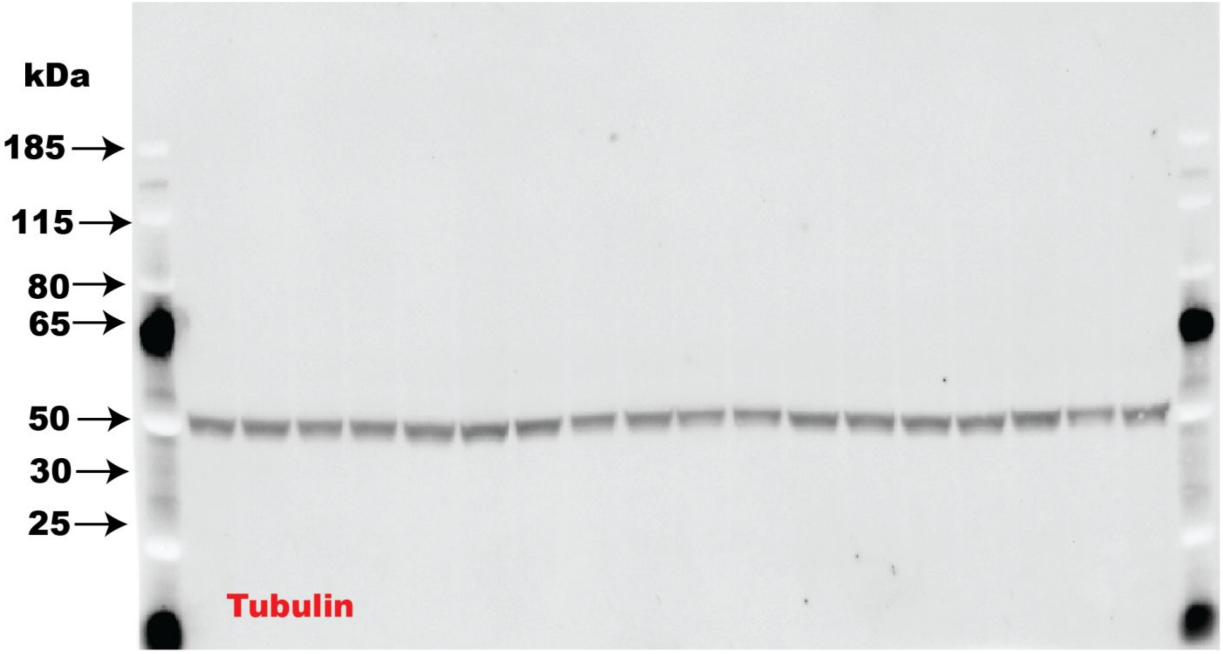
kDa
185
115
80
65
50
30
25
Tubulin

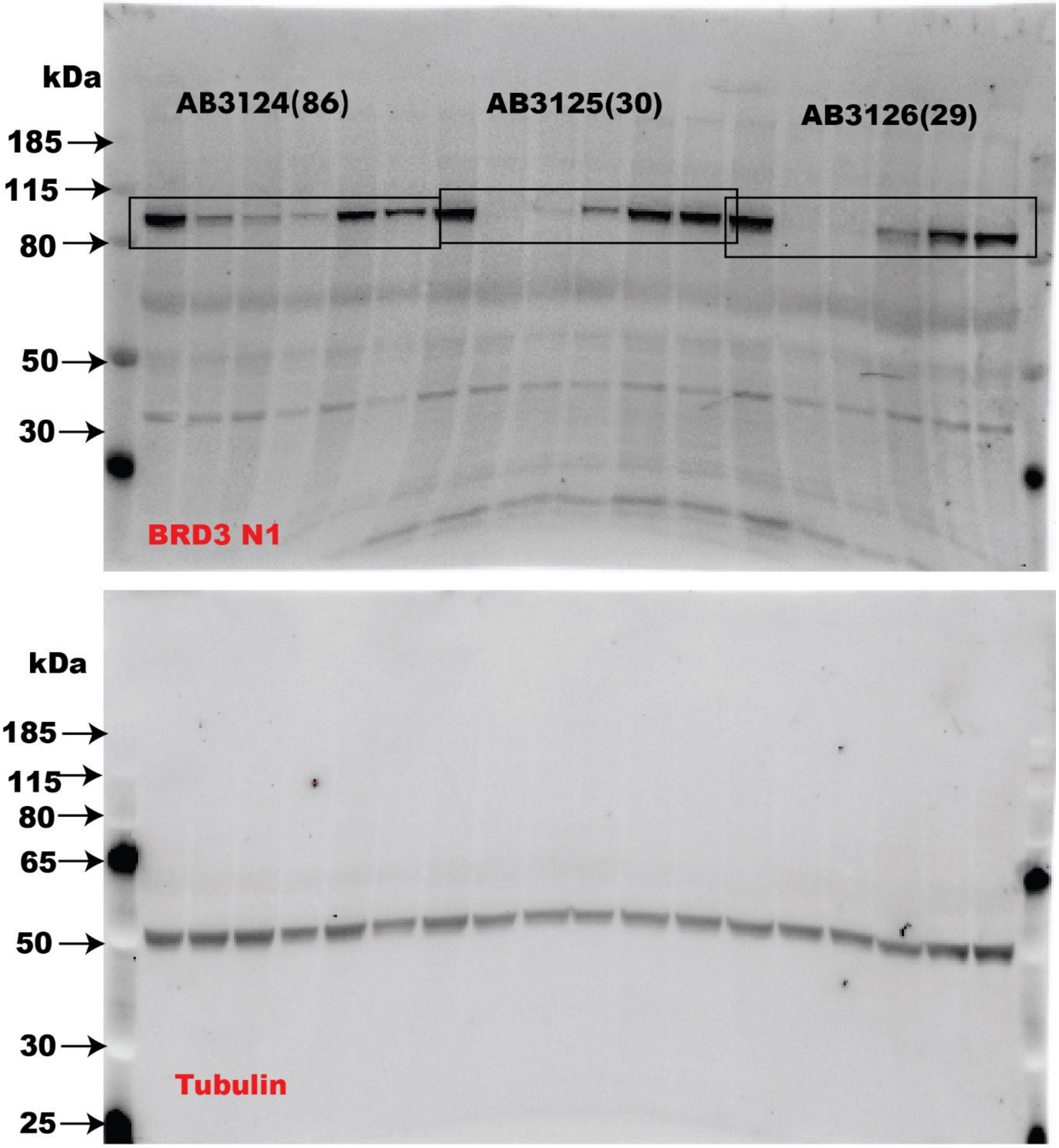
kDa
AB3124(86)
AB3125(30)
AB3126(29)
185
115
80
50
30
BRD3 N1
kDa
185
115
80
65
50
30
Tubulin
25

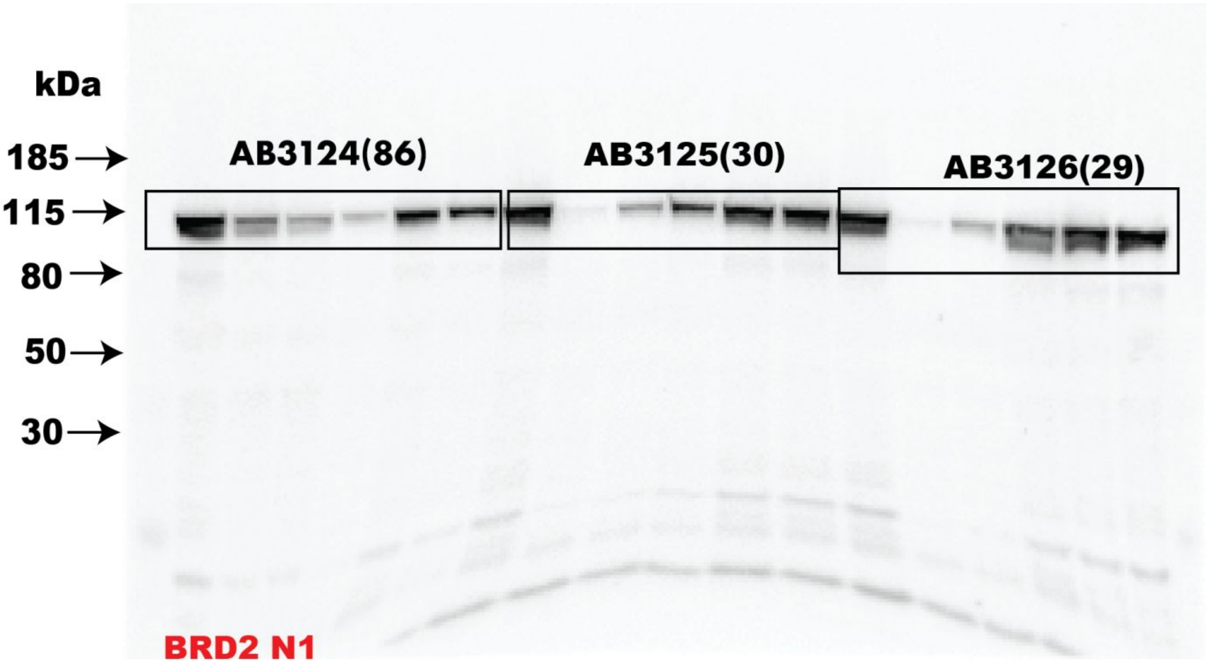
kDa
185
115
80
50
30
AB3124(86)
AB3125(30)
AB3126(29)
BRD2 N1

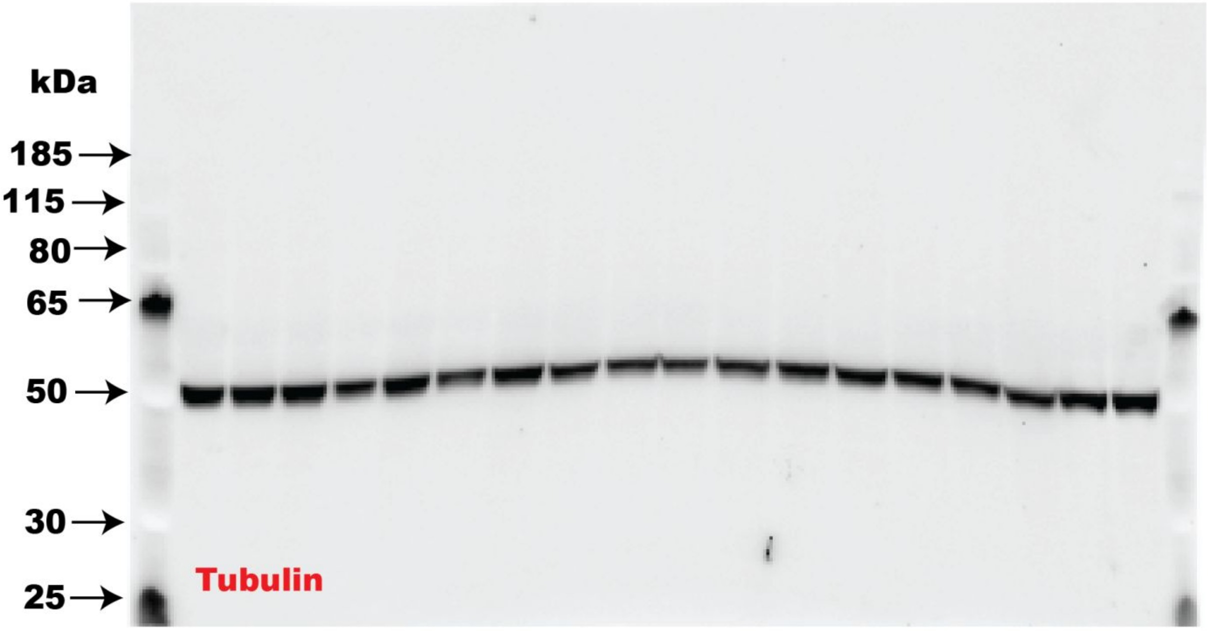
kDa
185
115
80
65
50
30
25
Tubulin

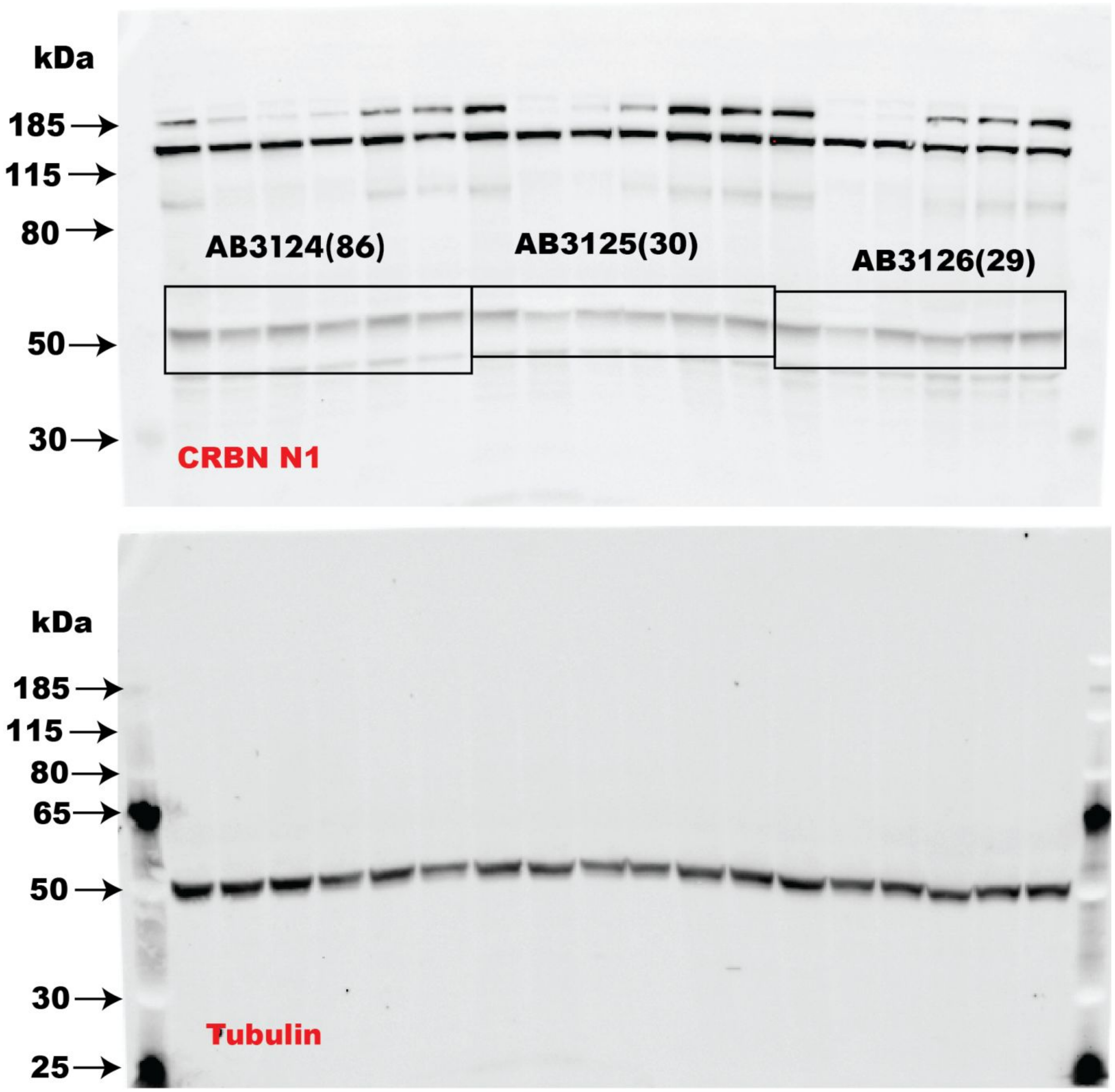
kDa
185
115
80
AB3124(86)
AB3125(30)
AB3126(29)
50
30
CRBN N1
kDa
185
115
80
65
50
30
Tubulin
25

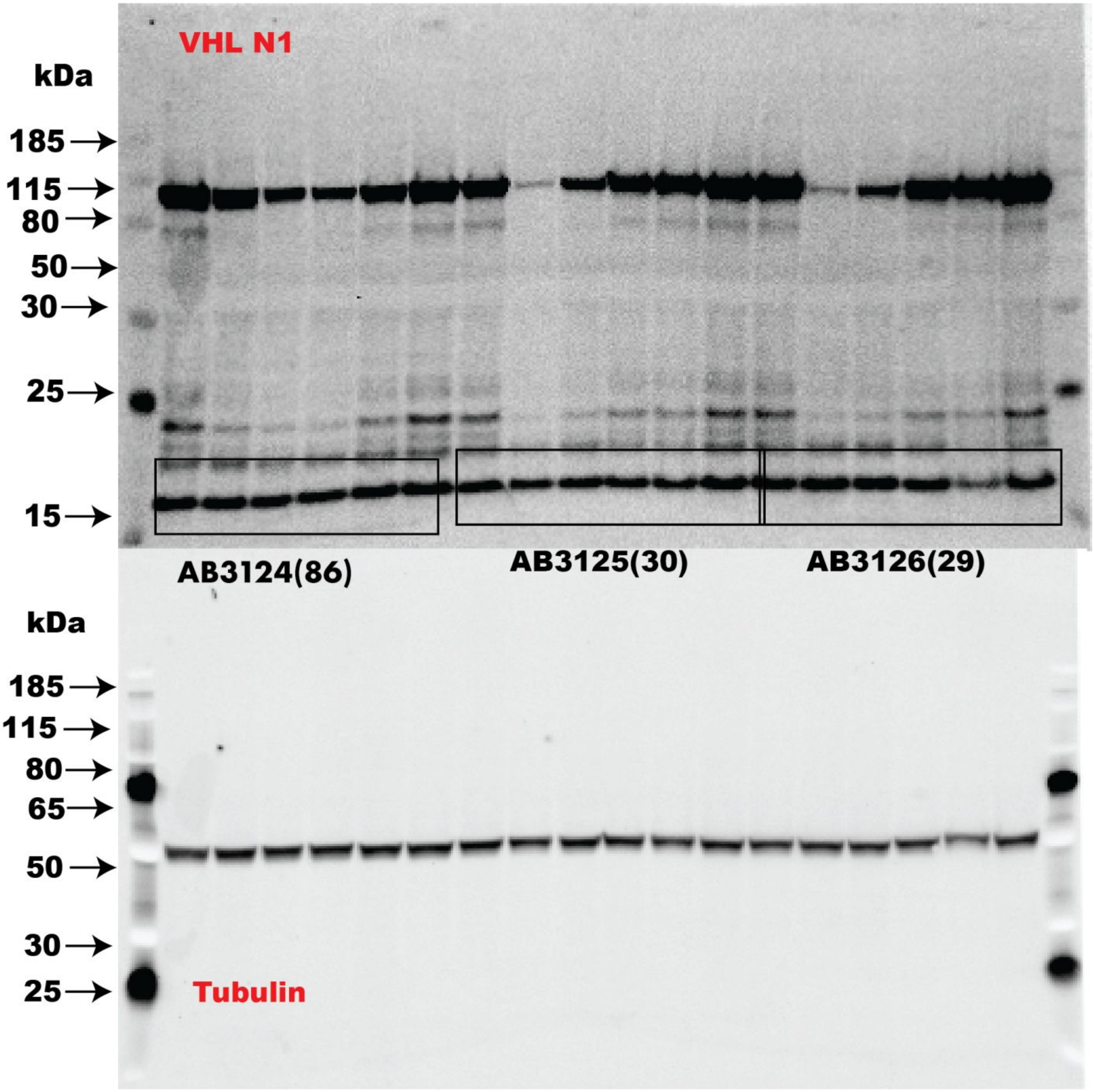
VHL N1
kDa
185
115
80
50
30
25
15
AB3124(86)
AB3125(30)
AB3126(29)
kDa
185
115
80
65
50
30
25
Tubulin

Supplementary Figure 9 (related to Figure 4). **Uncropped independent replicate western blotting images of the evaluation of heterotrivalent PROTACs 23 – 32 & 86 in HEK293 cells.**

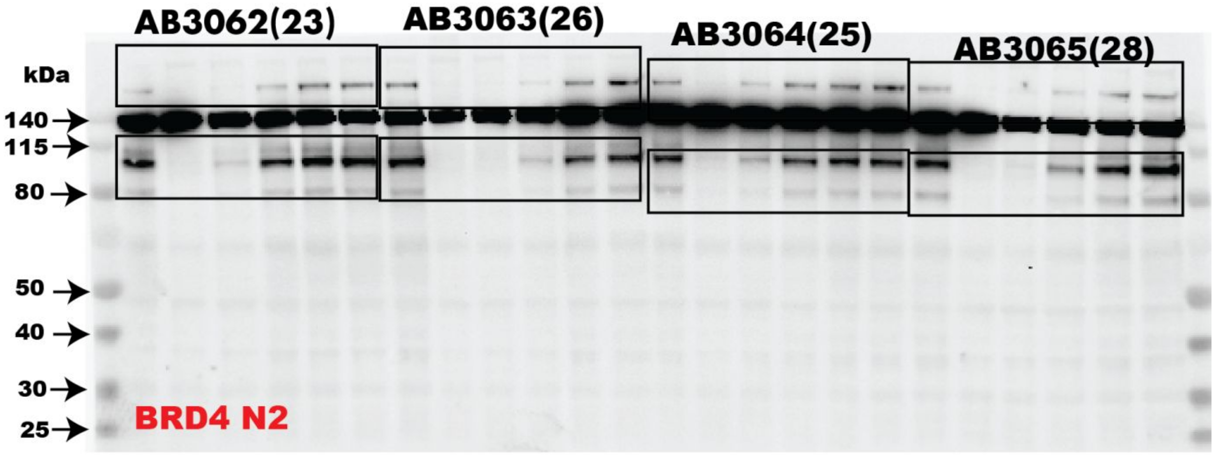

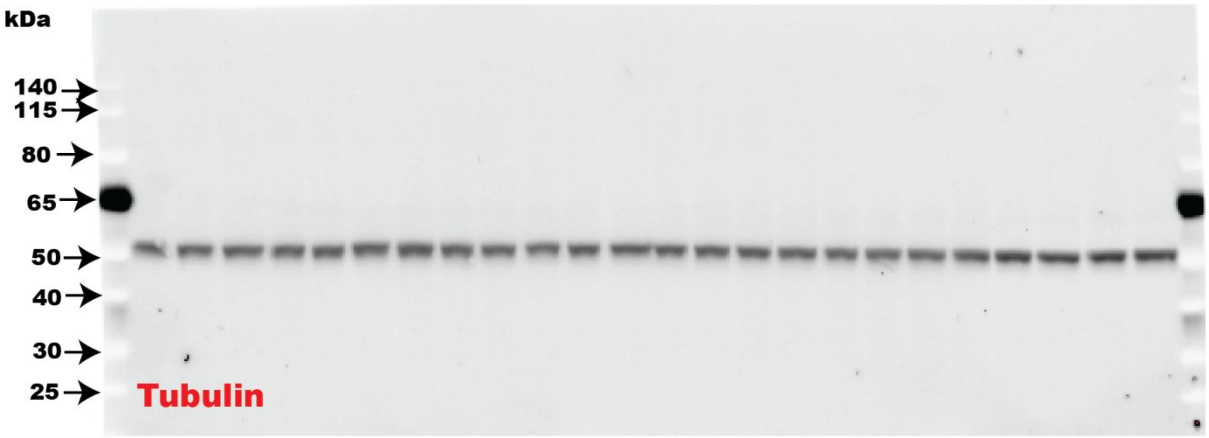

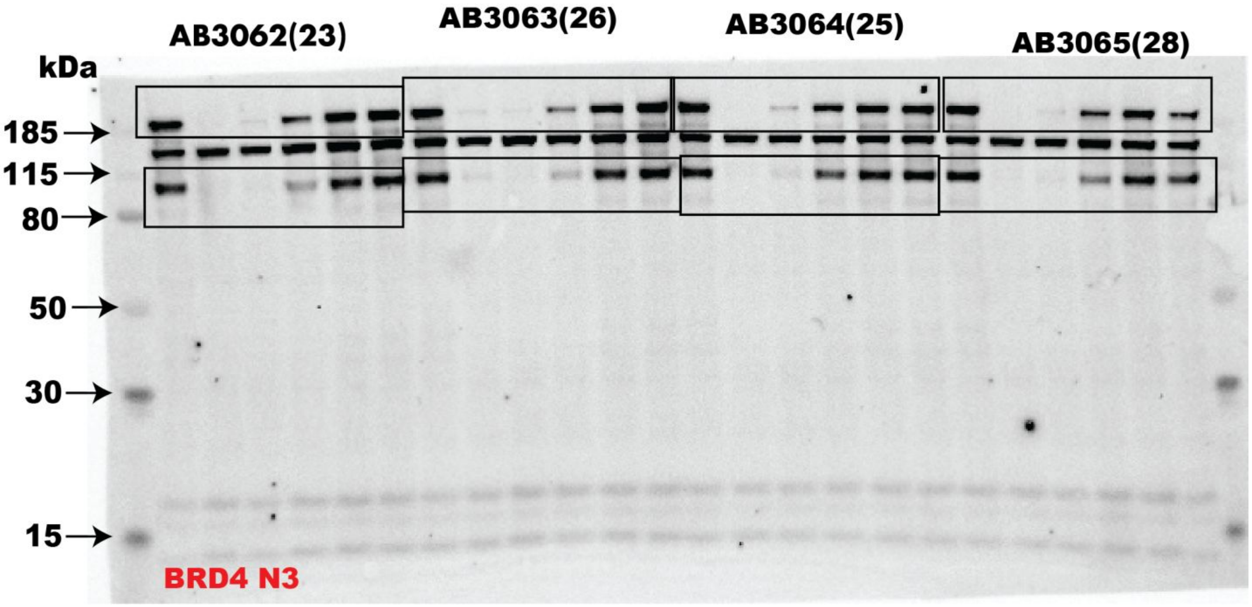
AB3062(23)
AB3063(26)
AB3064(25)
AB3065(28)
kDa
185
115
80
50
30
15
BRD4 N3

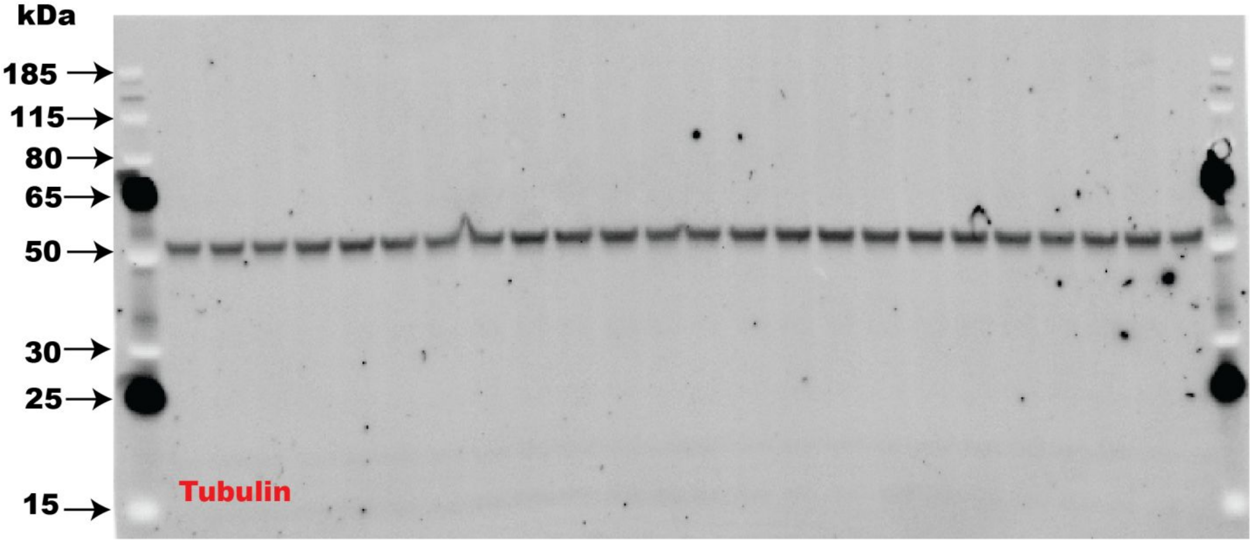
kDa
185
115
80
65
50
30
25
15
Tubulin

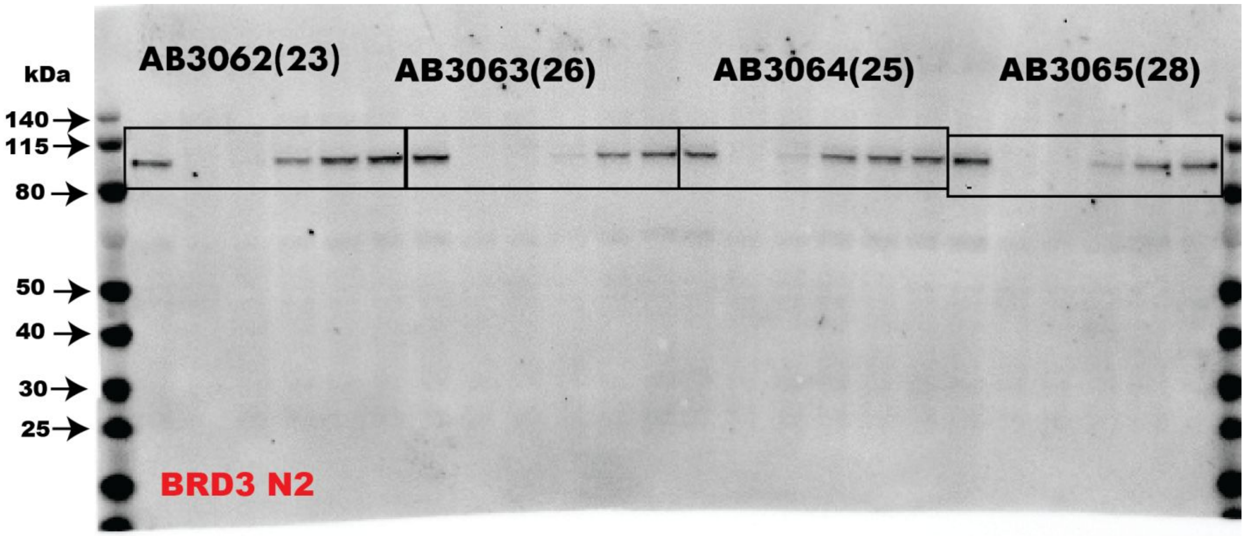
kDa
AB3062(23)
AB3063(26)
AB3064(25)
AB3065(28)
140
115
80
50
40
30
25
BRD3 N2

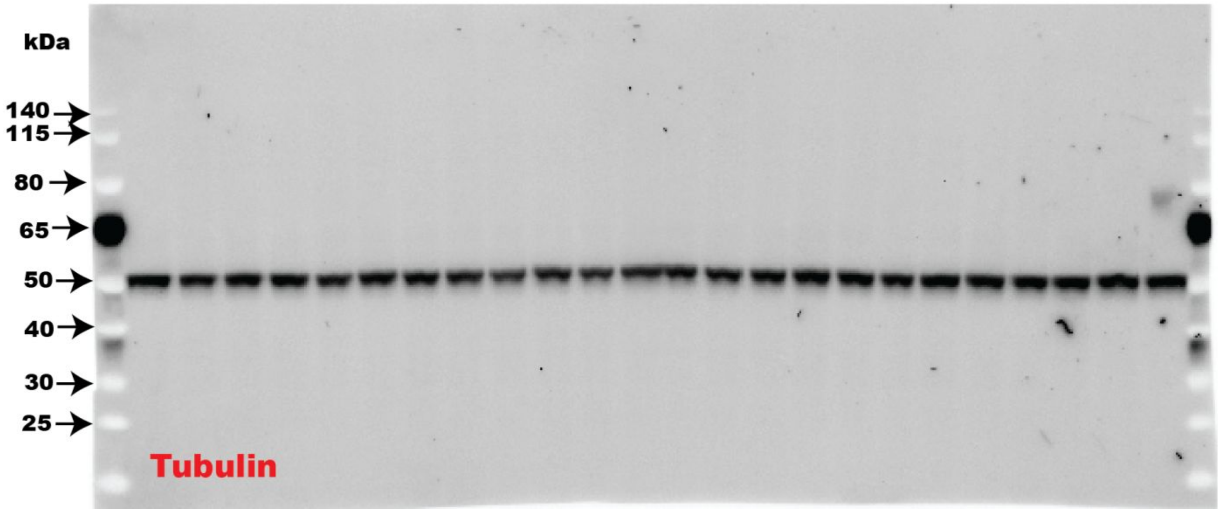
kDa
140
115
80
65
50
40
30
25
Tubulin

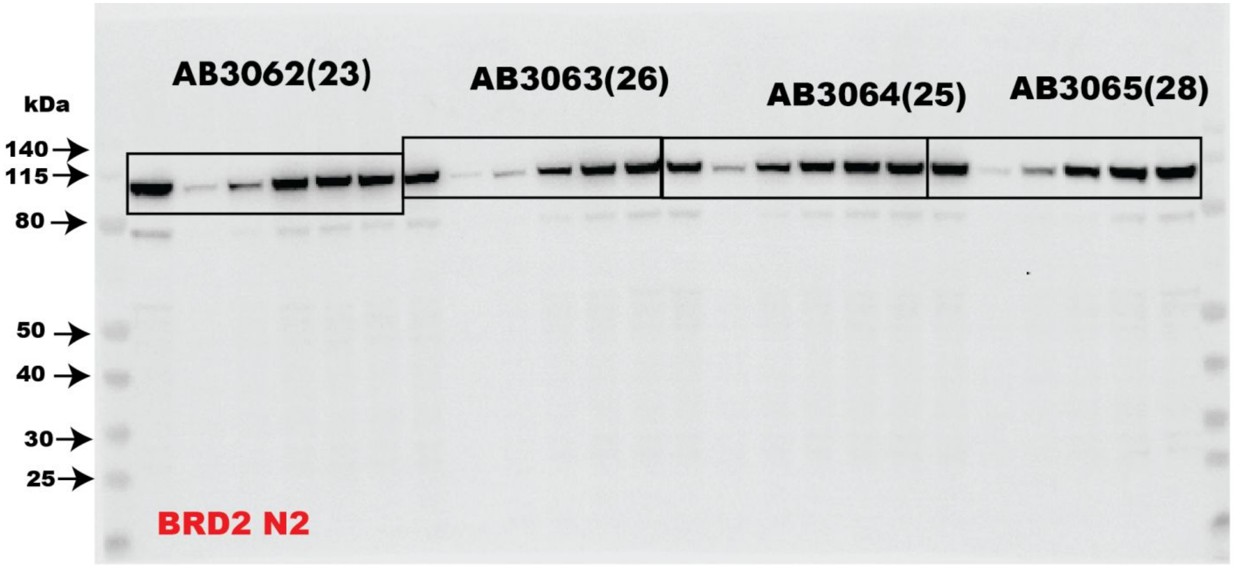
AB3062(23)
AB3063(26)
AB3064(25)
AB3065(28)
kDa
140
115
80
50
40
30
25
BRD2 N2

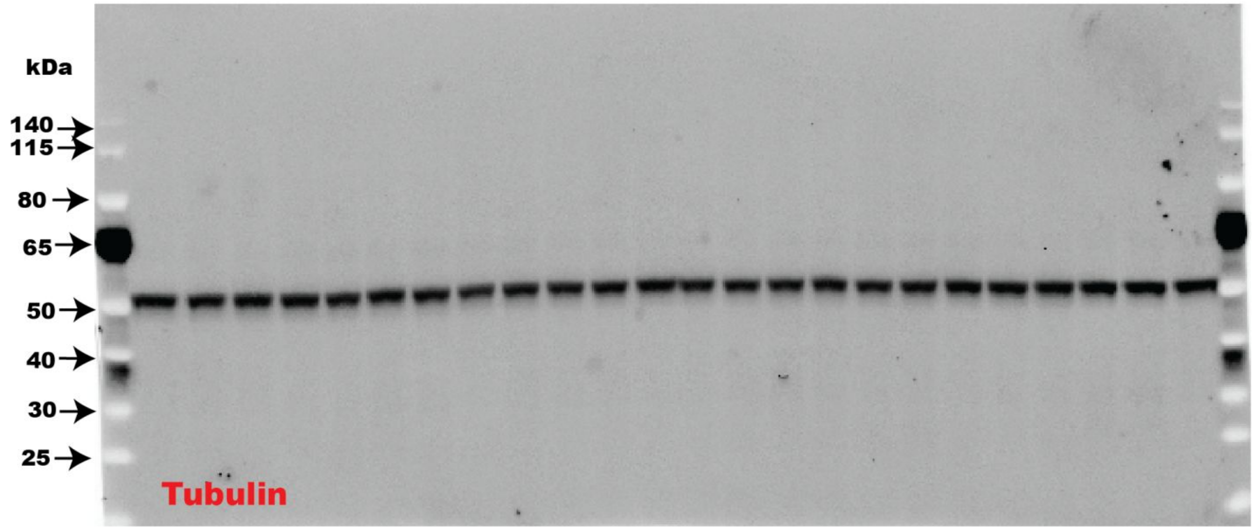
kDa
140
115
80
65
50
40
30
25
Tubulin

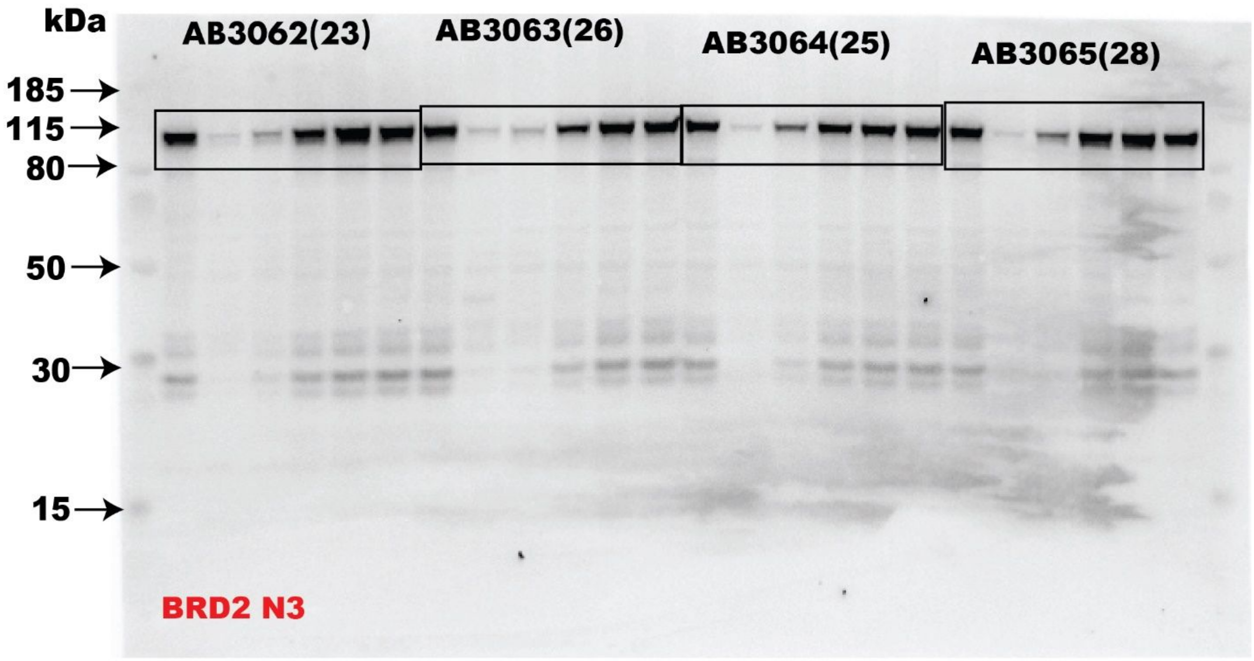
kDa
AB3062(23)
AB3063(26)
AB3064(25)
AB3065(28)
185
115
80
50
30
15
BRD2 N3

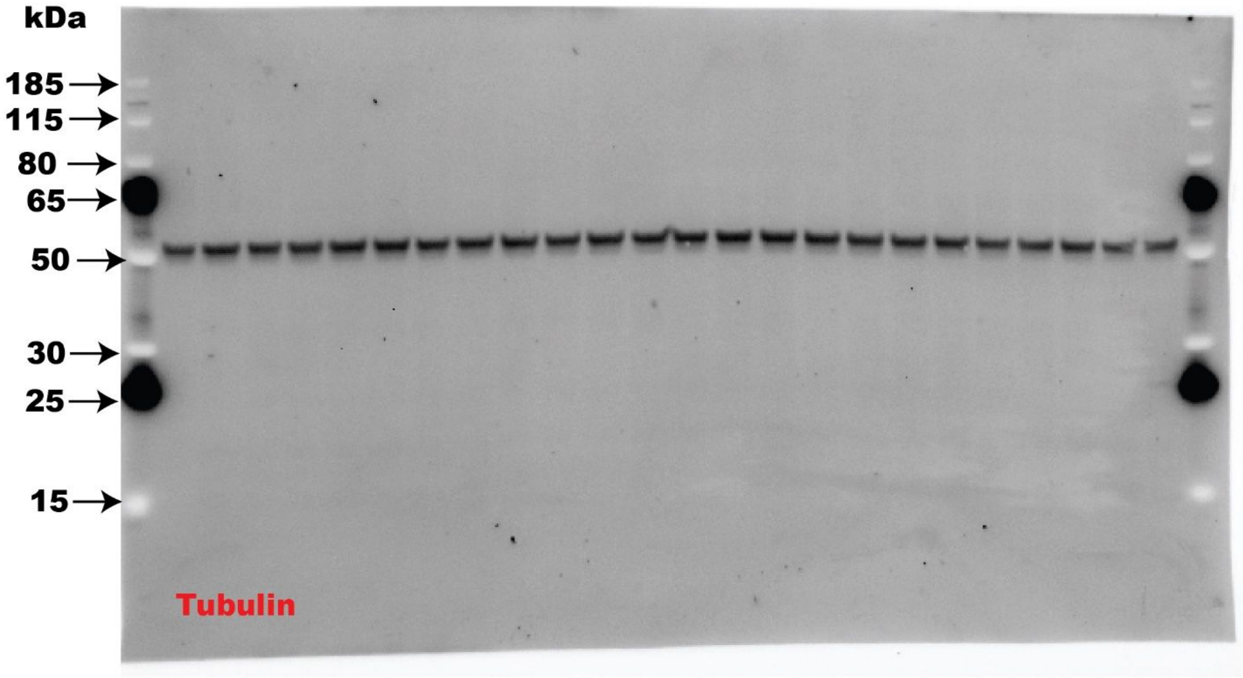
kDa
185
115
80
65
50
30
25
15
Tubulin

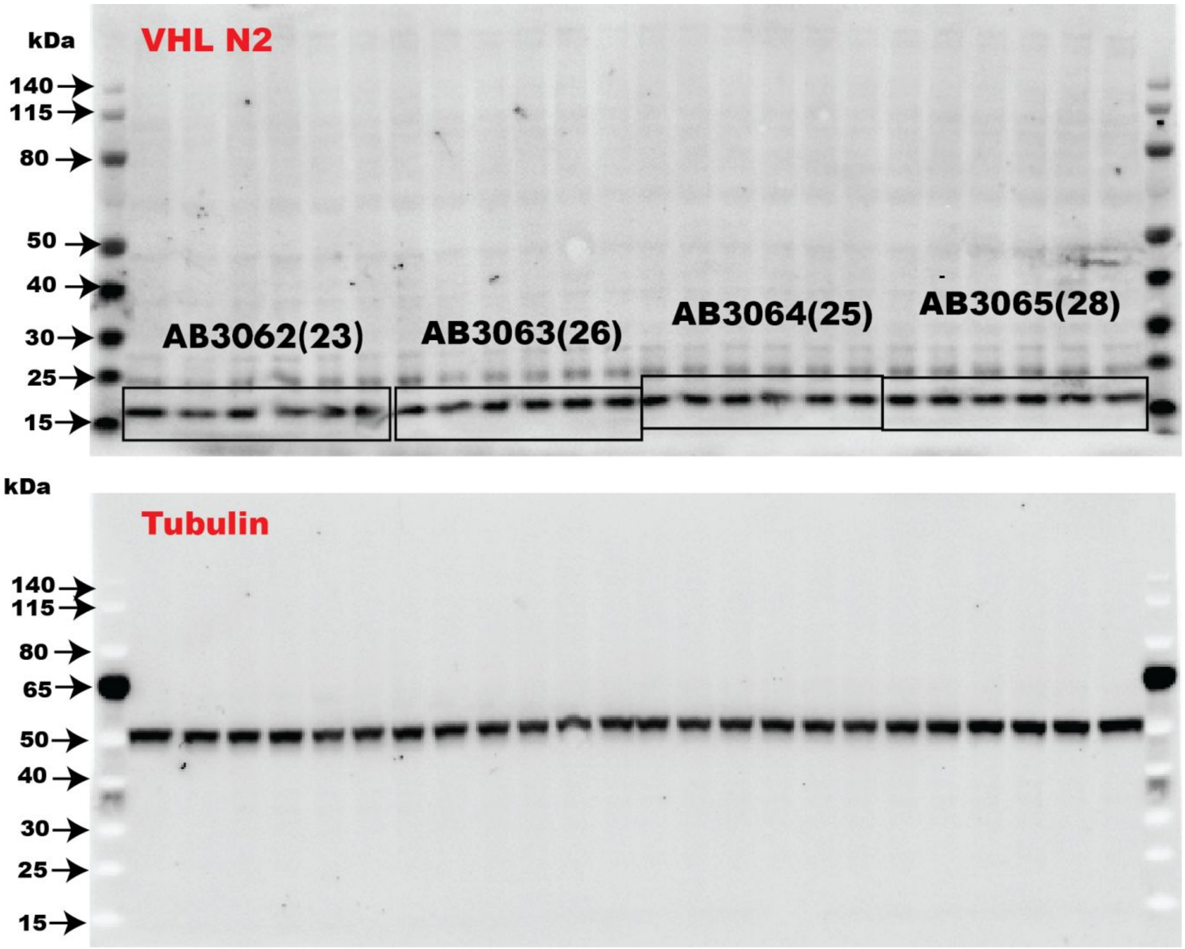
kDa
VHL N2
140
115
80
50
40
30
25
15
AB3062(23)
AB3063(26)
AB3064(25)
AB3065(28)
kDa
Tubulin
140
115
80
65
50
40
30
25
15

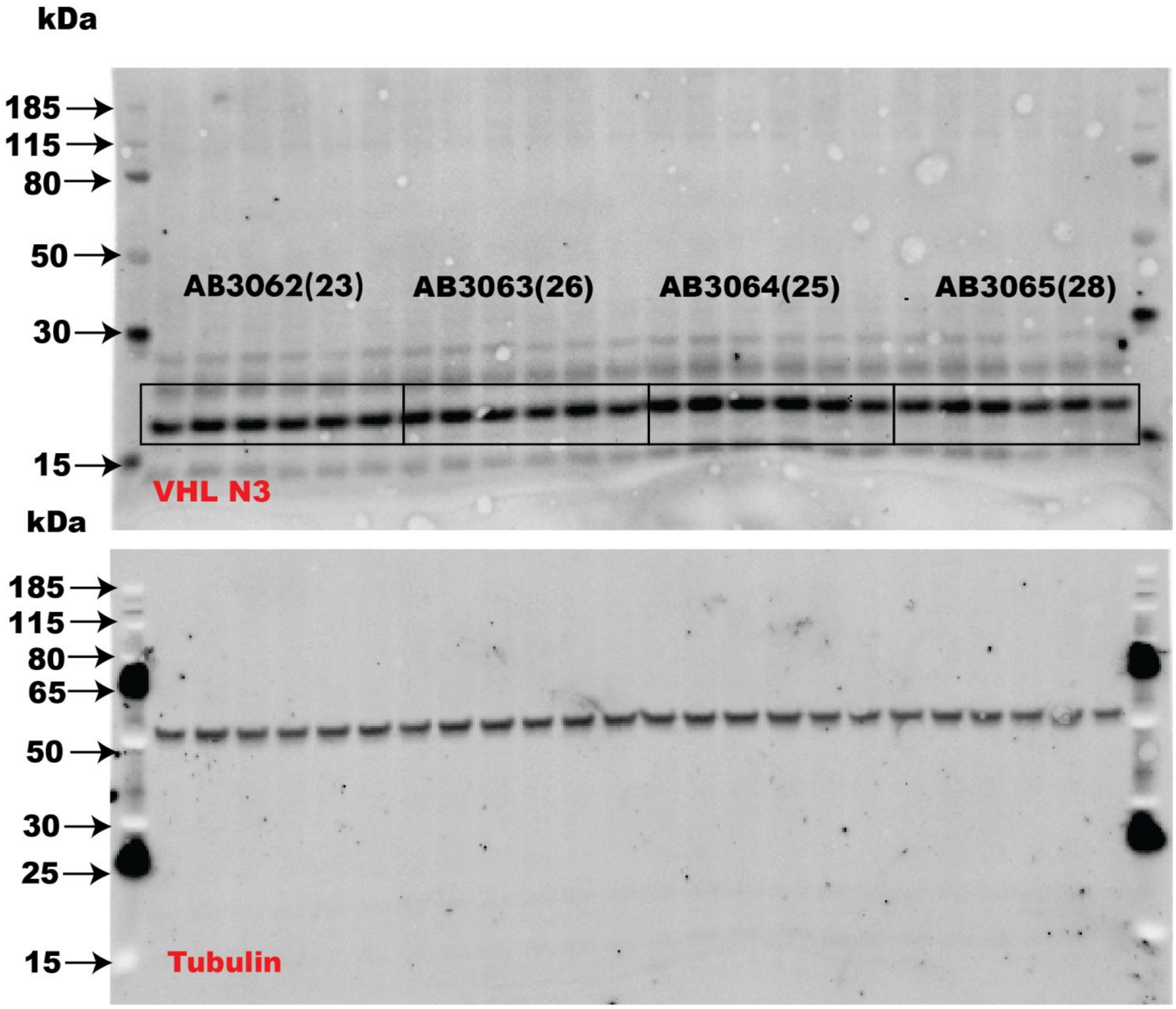

kDa
185
115
80
50
30
15
AB3062(23)
AB3063(26)
AB3064(25)
AB3065(28)
VHL N3
kDa
185
115
80
65
50
30
25
15
Tubulin

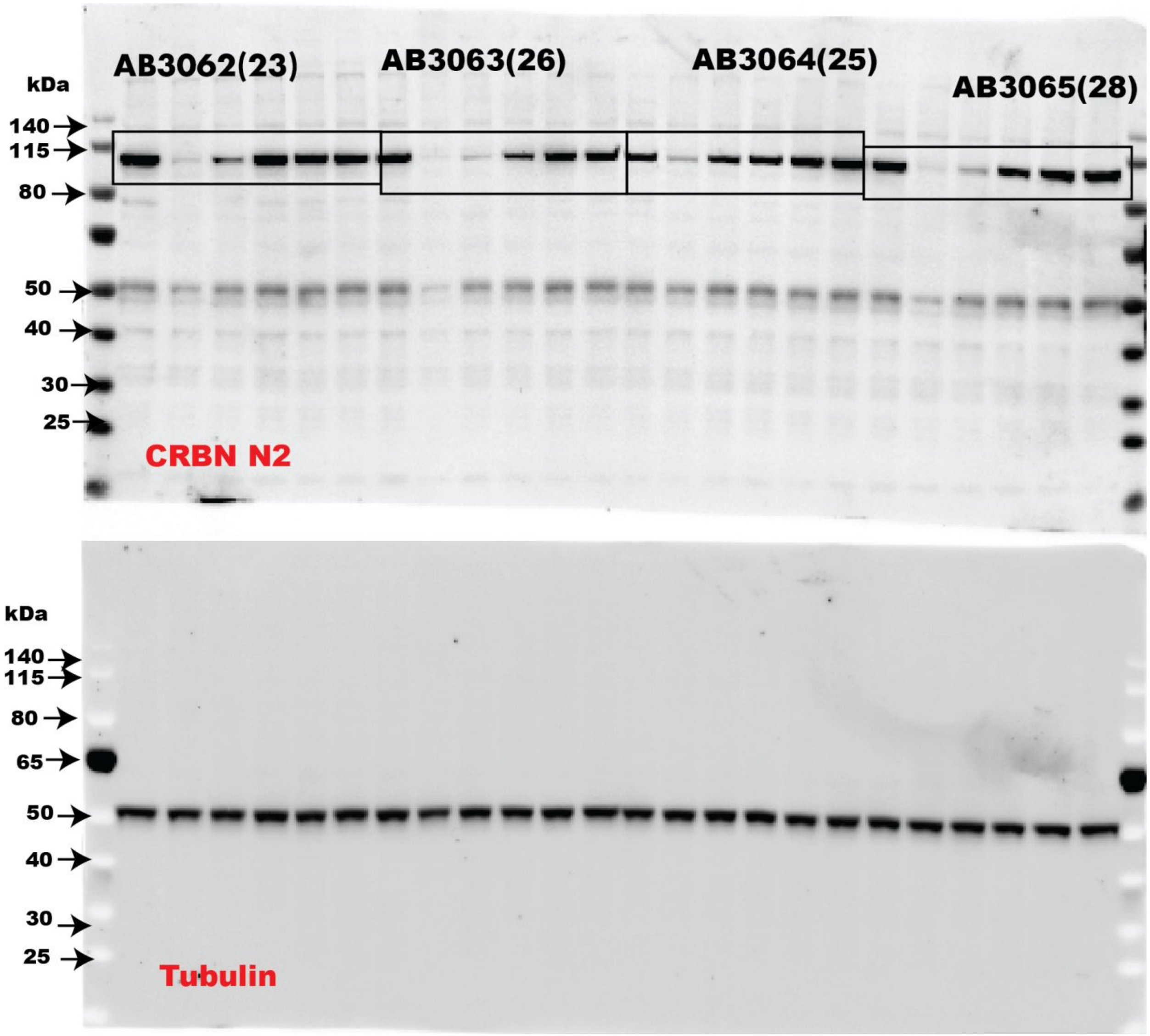

AB3062(23)
AB3063(26)
AB3064(25)
AB3065(28)
kDa
140
115
80
50
40
30
25
CRBN N2
kDa
140
115
80
65
50
40
30
25
Tubulin

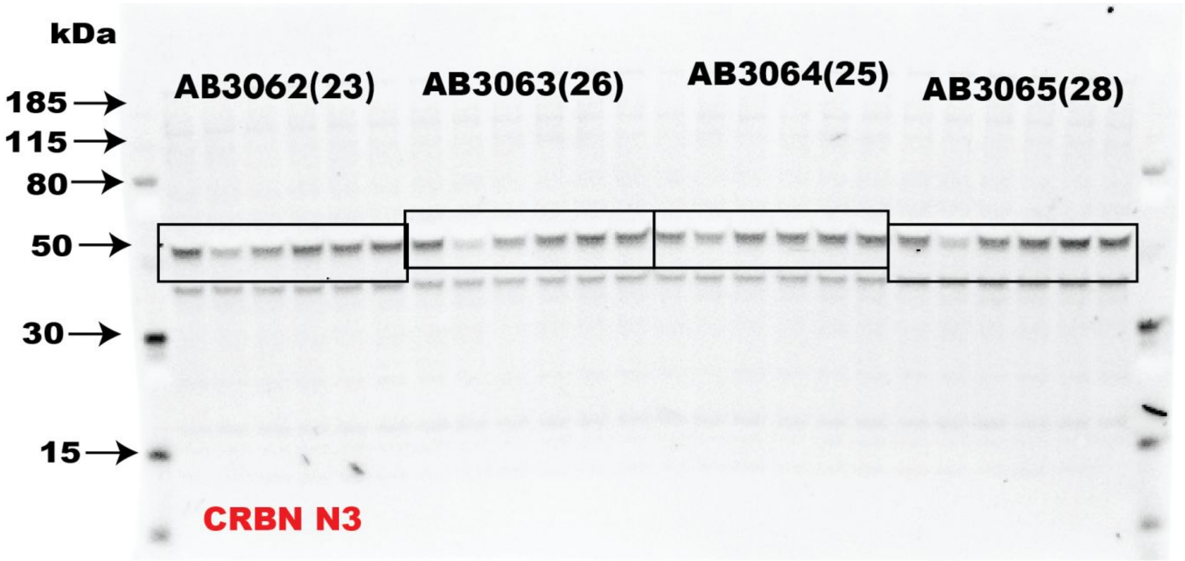
kDa
AB3062(23)
AB3063(26)
AB3064(25)
AB3065(28)
185
115
80
50
30
15
CRBN N3

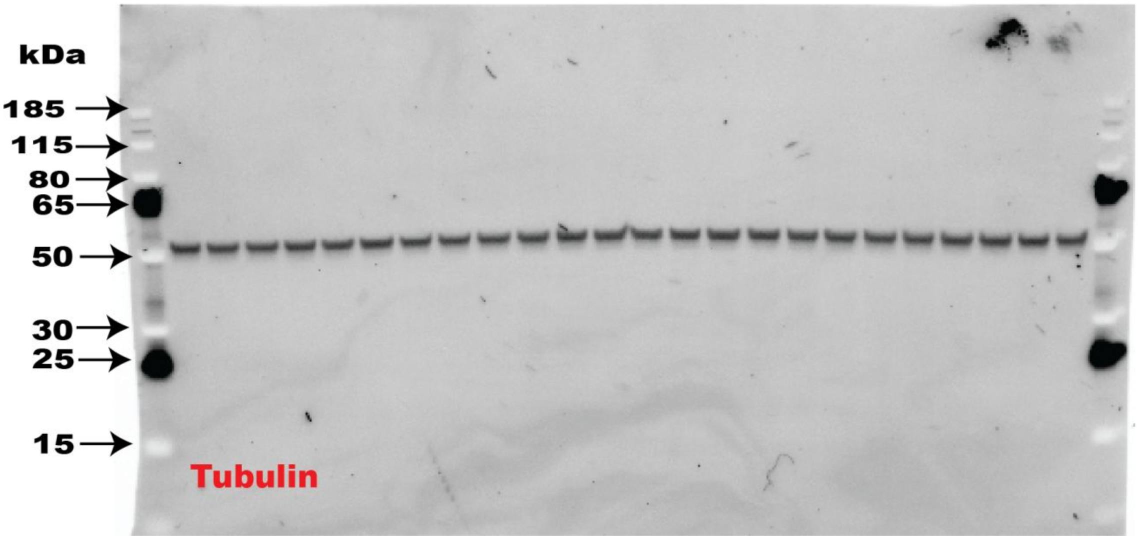
kDa
185
115
80
65
50
30
25
15
Tubulin

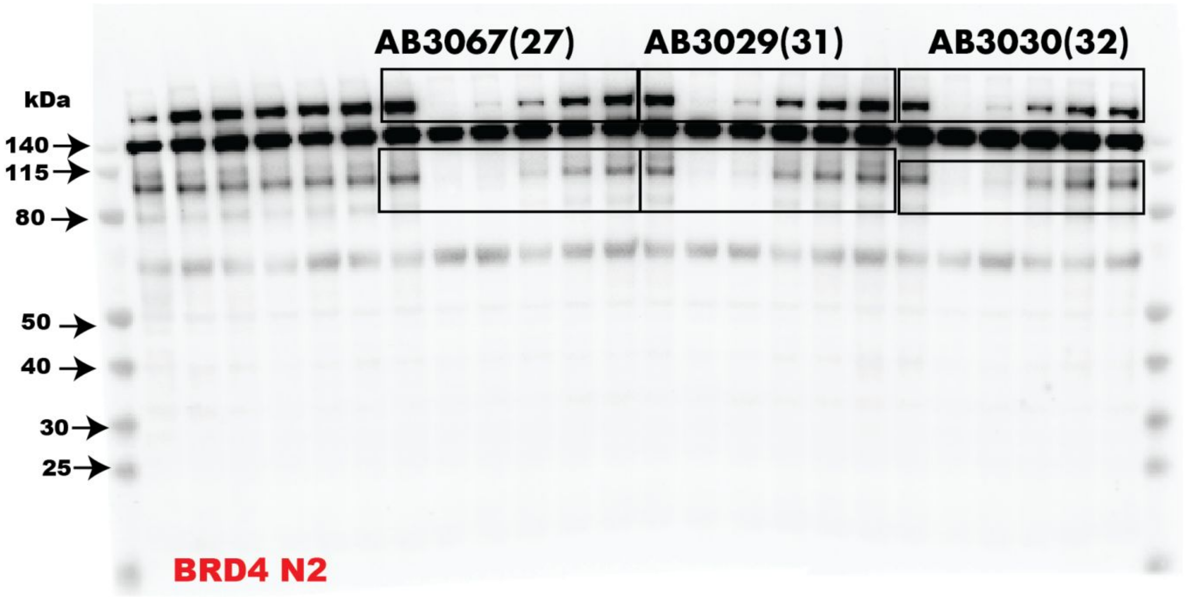
AB3067(27)
AB3029(31)
AB3030(32)
kDa
140
115
80
50
40
30
25
BRD4 N2

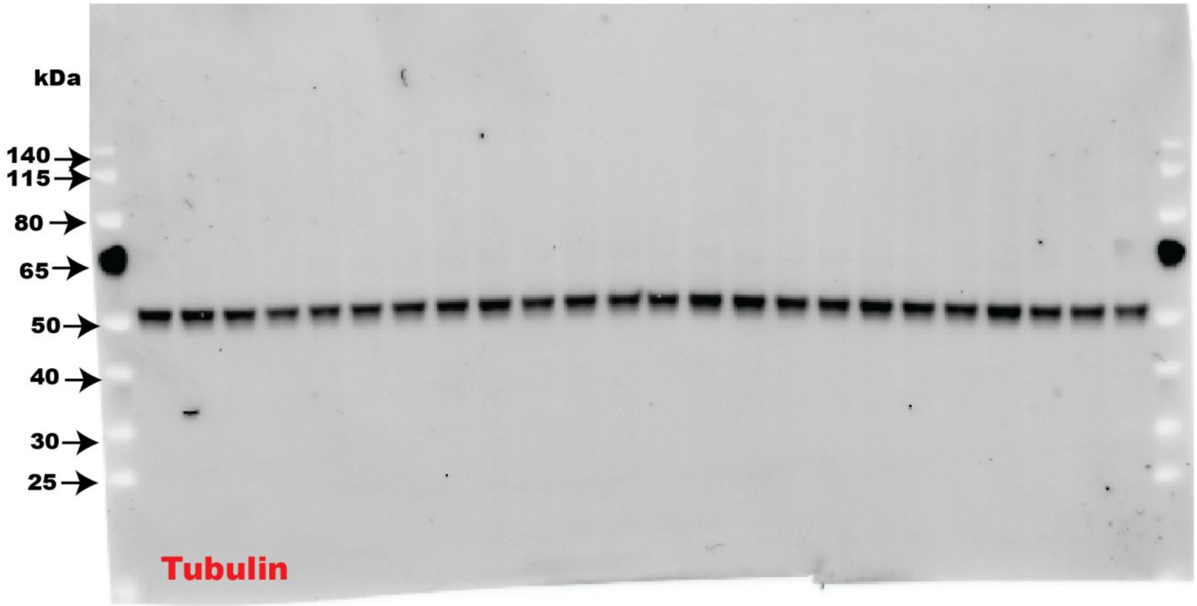
kDa
140
115
80
65
50
40
30
25
Tubulin

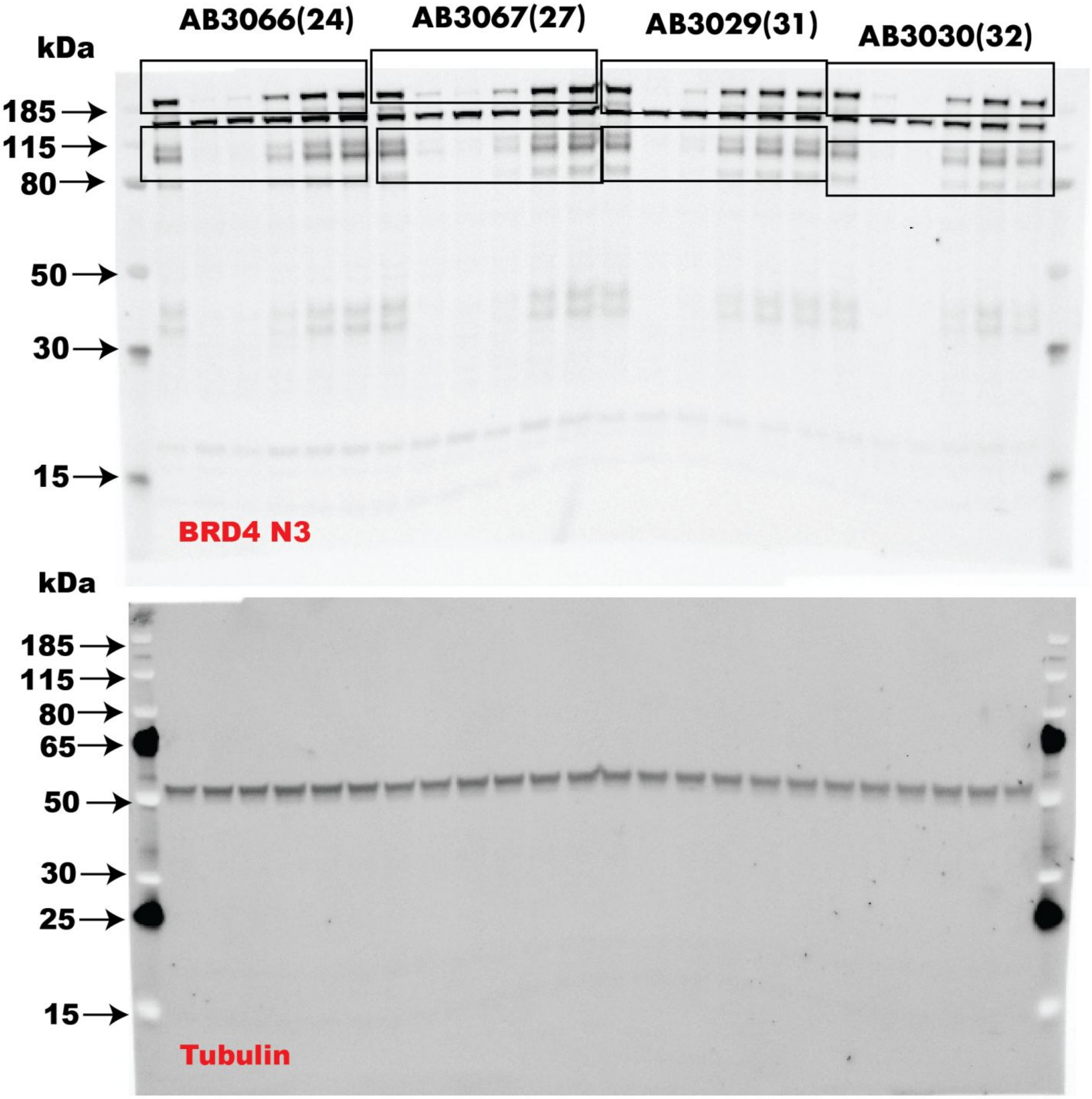
AB3066(24)
AB3067(27)
AB3029(31)
AB3030(32)
kDa
185
115
80
50
30
15
BRD4 N3
kDa
185
115
80
65
50
30
25
15
Tubulin

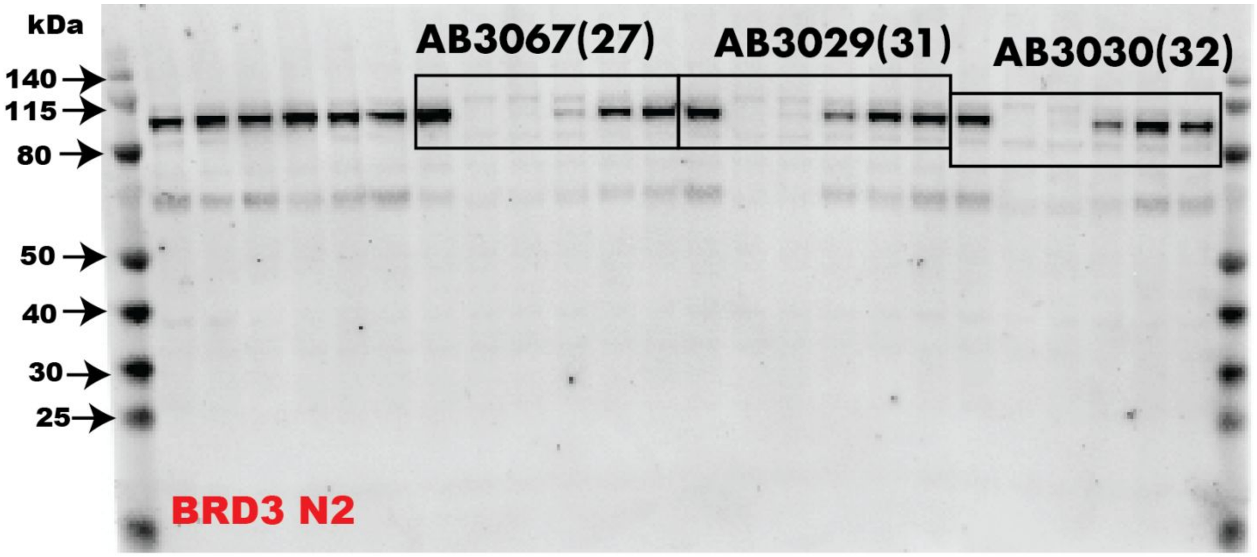
kDa
140
115
80
50
40
30
25
AB3067(27)
AB3029(31)
AB3030(32)
BRD3 N2

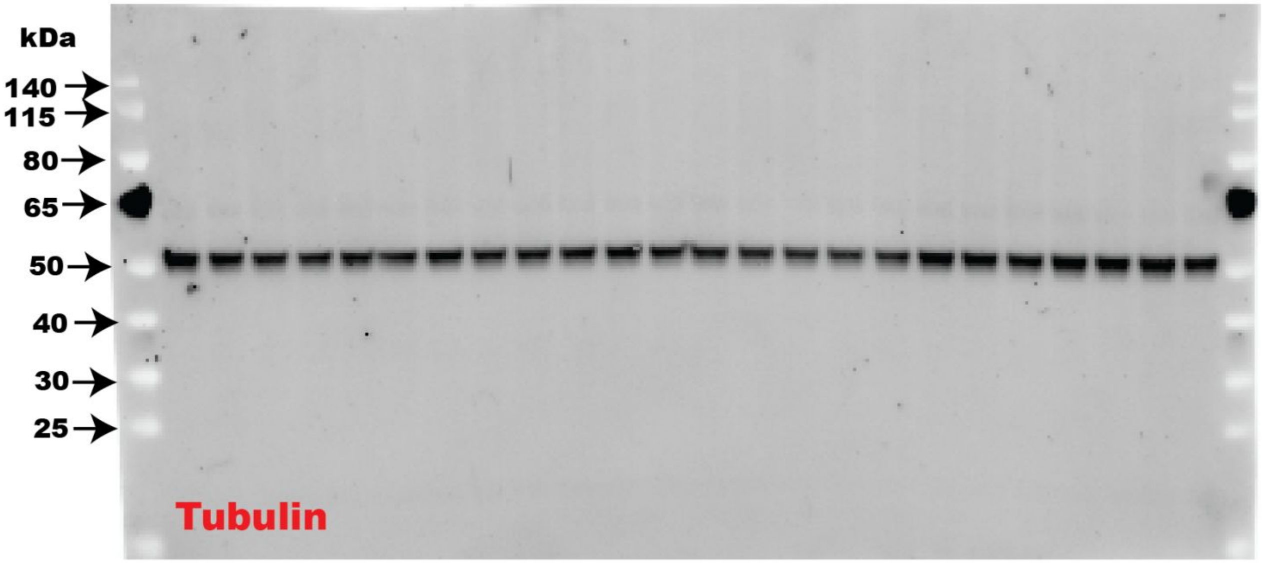
kDa
140
115
80
65
50
40
30
25
Tubulin

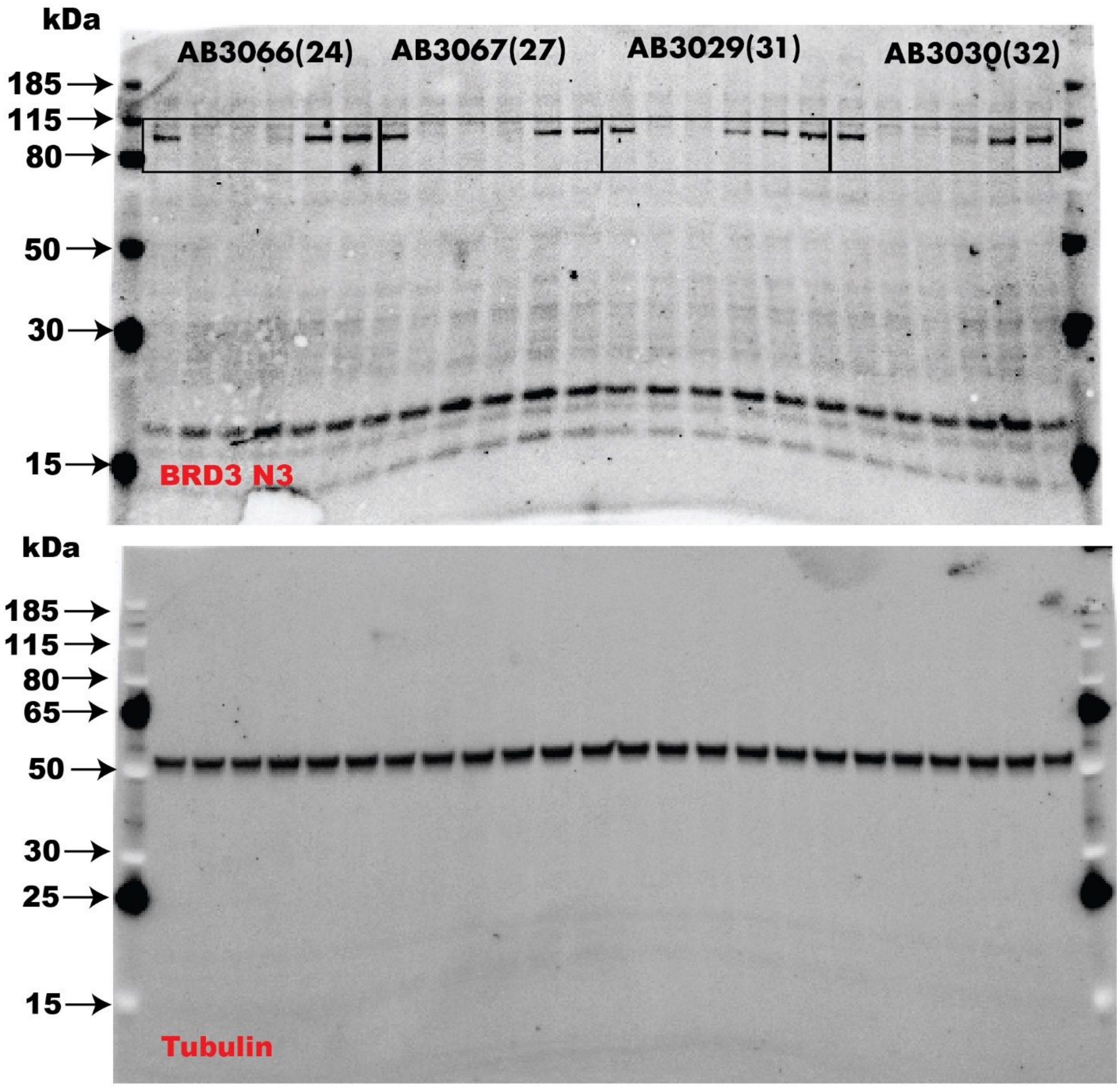
kDa
AB3066(24)
AB3067(27)
AB3029(31)
AB3030(32)
185
115
80
50
30
15
BRD3 N3
kDa
185
115
80
65
50
30
25
15
Tubulin

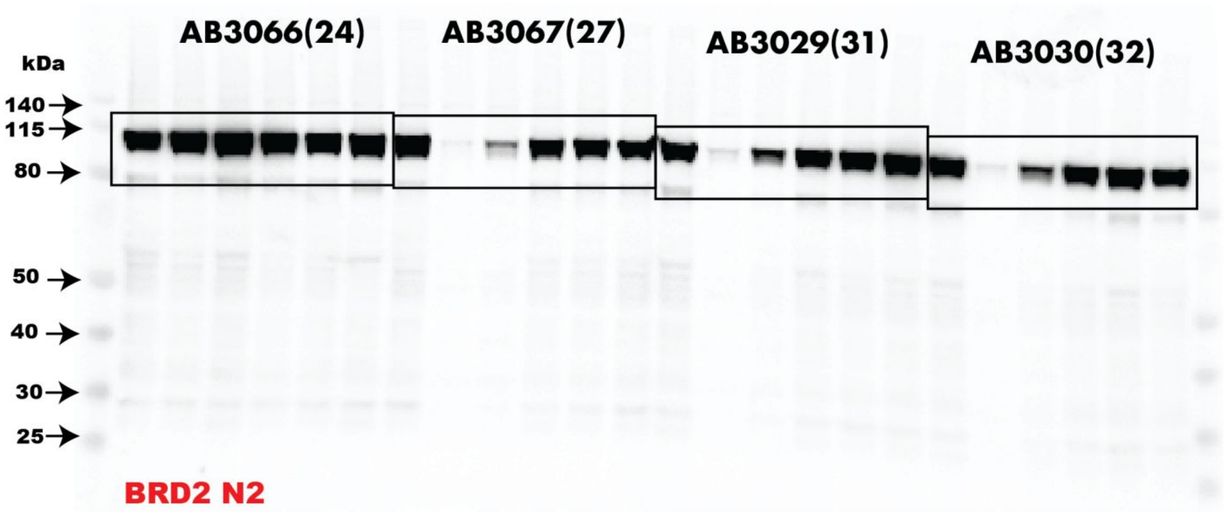
AB3066(24)
AB3067(27)
AB3029(31)
AB3030(32)
kDa
140
115
80
50
40
30
25
BRD2 N2

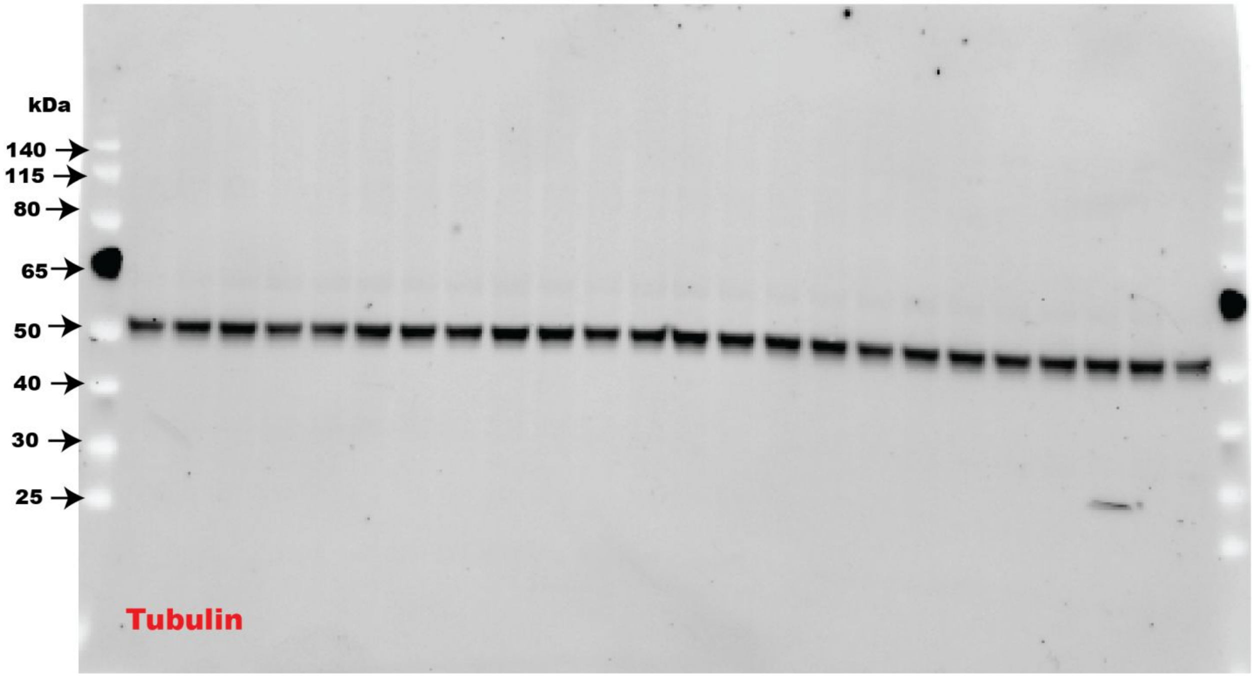
kDa
140
115
80
65
50
40
30
25
Tubulin

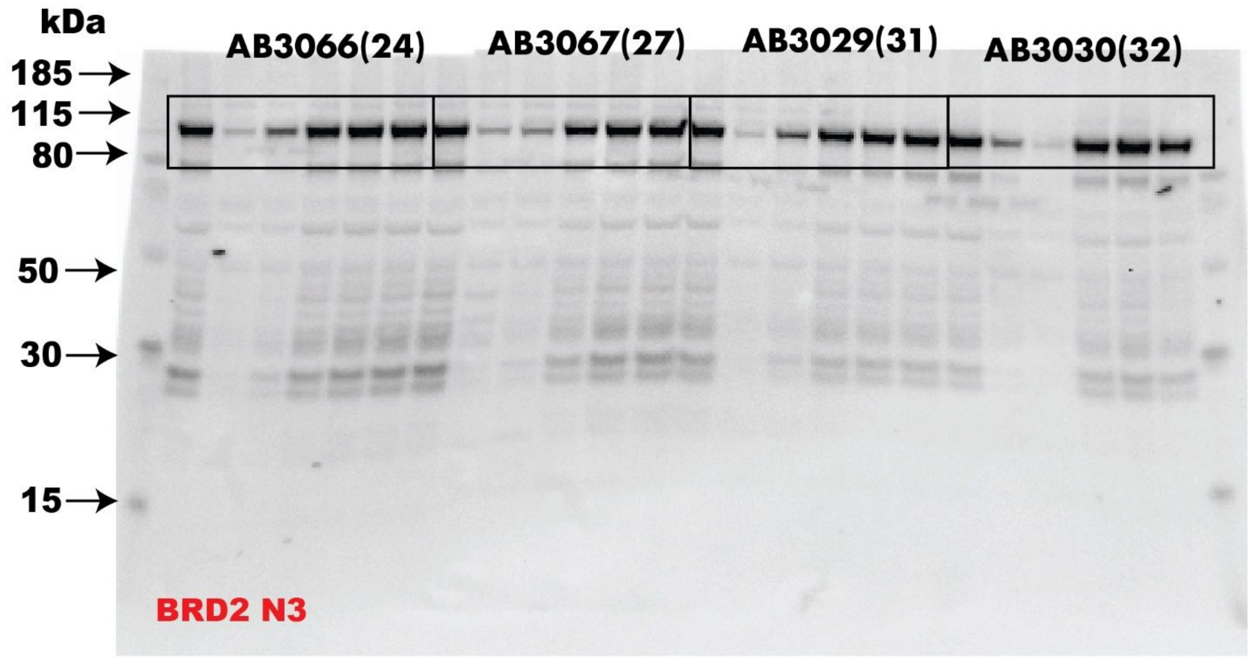
kDa
AB3066(24)
AB3067(27)
AB3029(31)
AB3030(32)
185
115
80
50
30
15
BRD2 N3

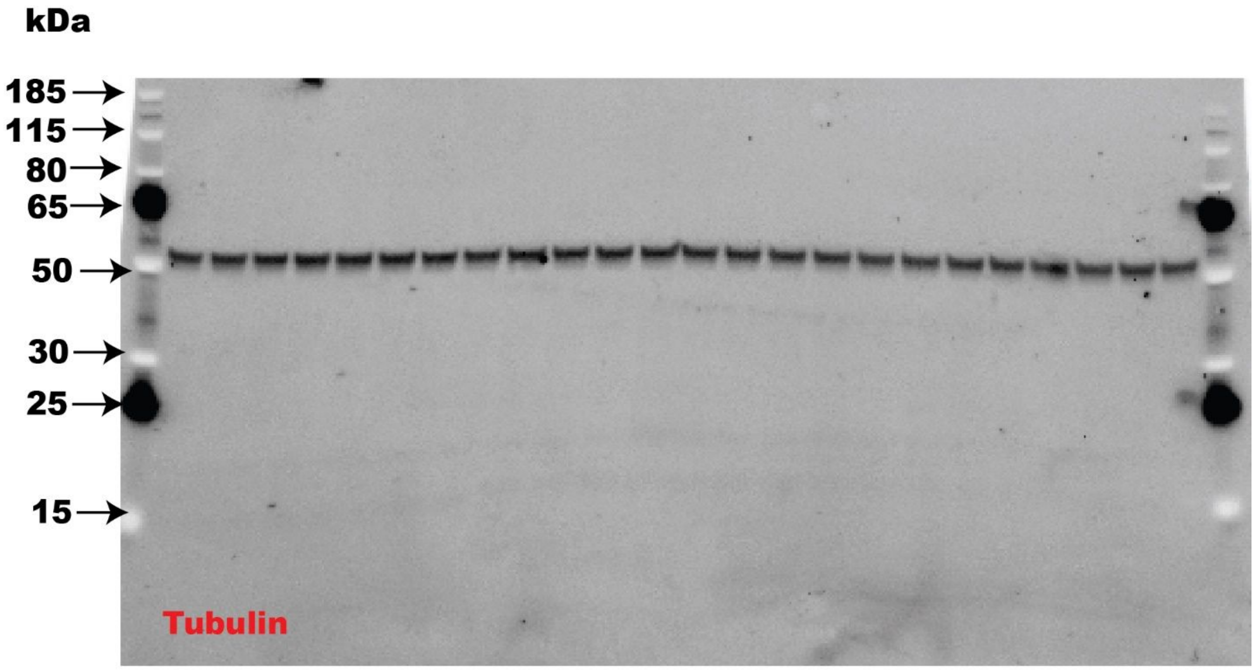
kDa
185
115
80
65
50
30
25
15
Tubulin

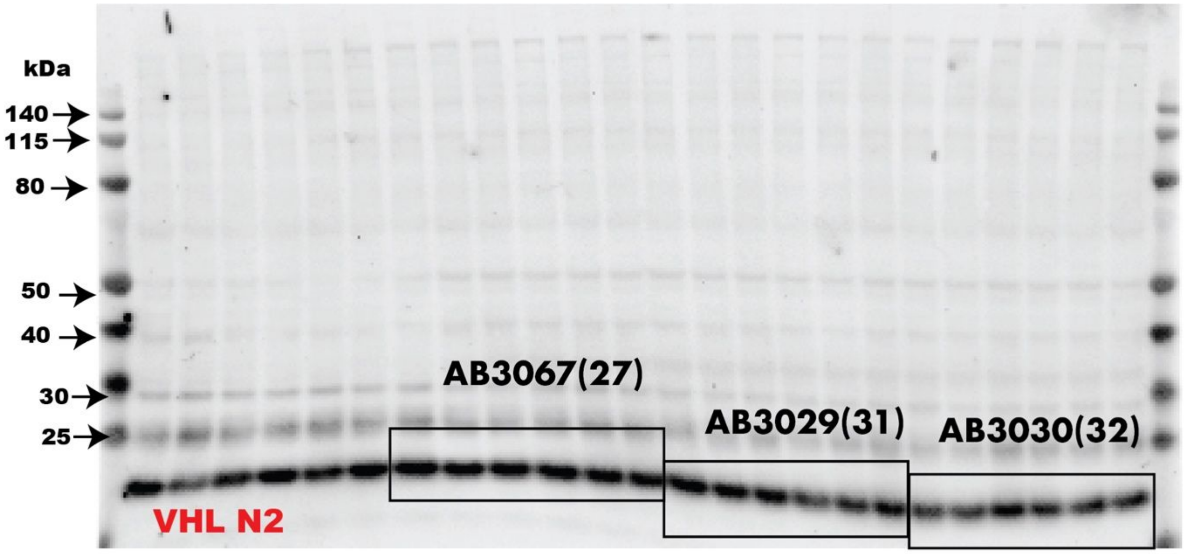
kDa
140
115
80
50
40
30
25
AB3067(27)
AB3029(31)
AB3030(32)
VHL N2

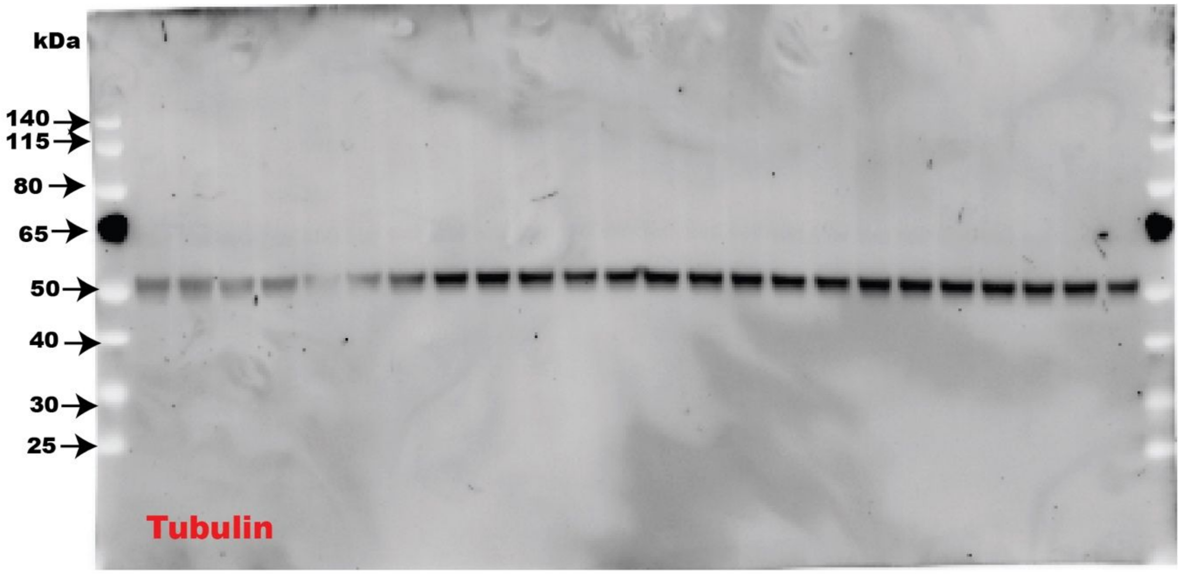
kDa
140
115
80
65
50
40
30
25
Tubulin

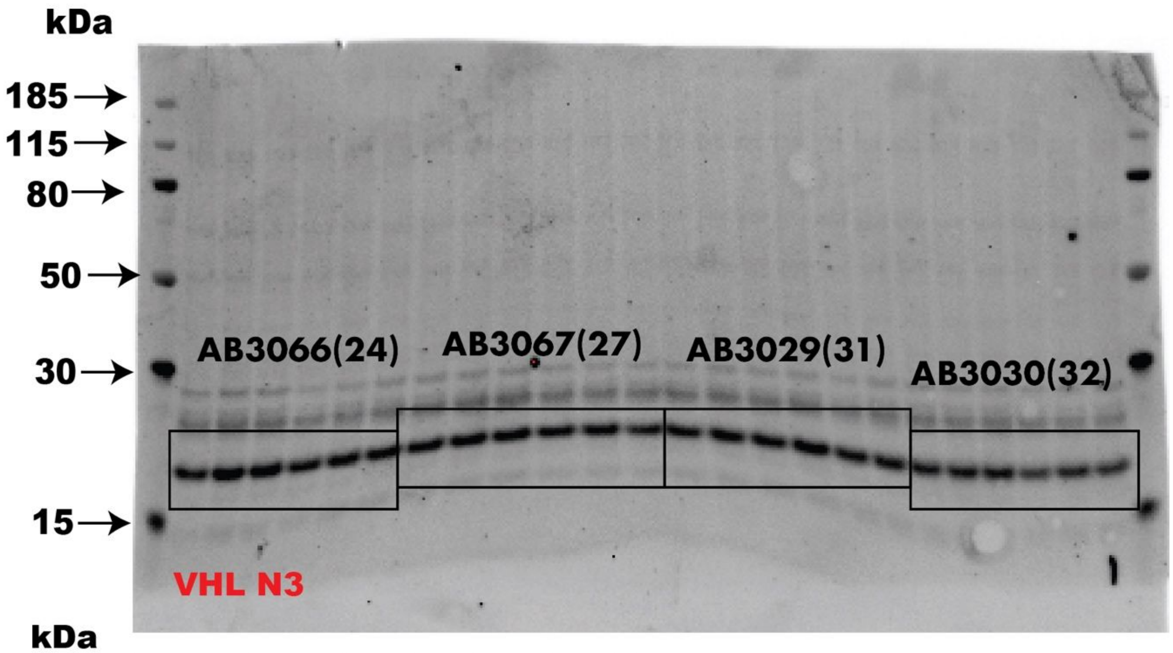
kDa
185
115
80
50
30
15
AB3066(24)
AB3067(27)
AB3029(31)
AB3030(32)
VHL N3

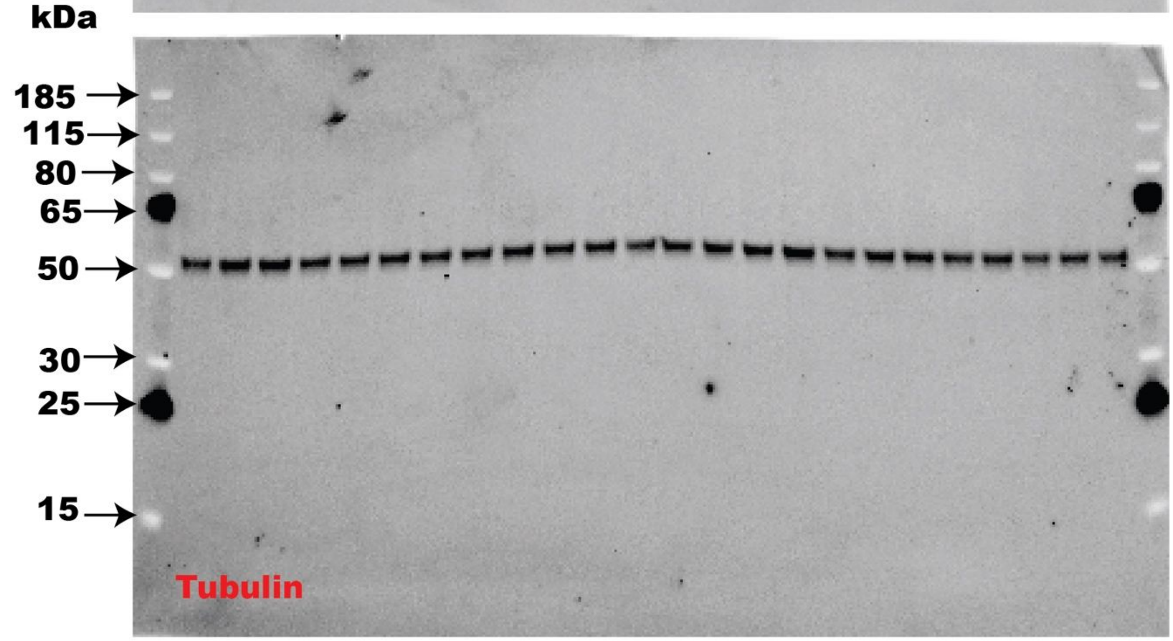
kDa
185
115
80
65
50
30
25
15
Tubulin

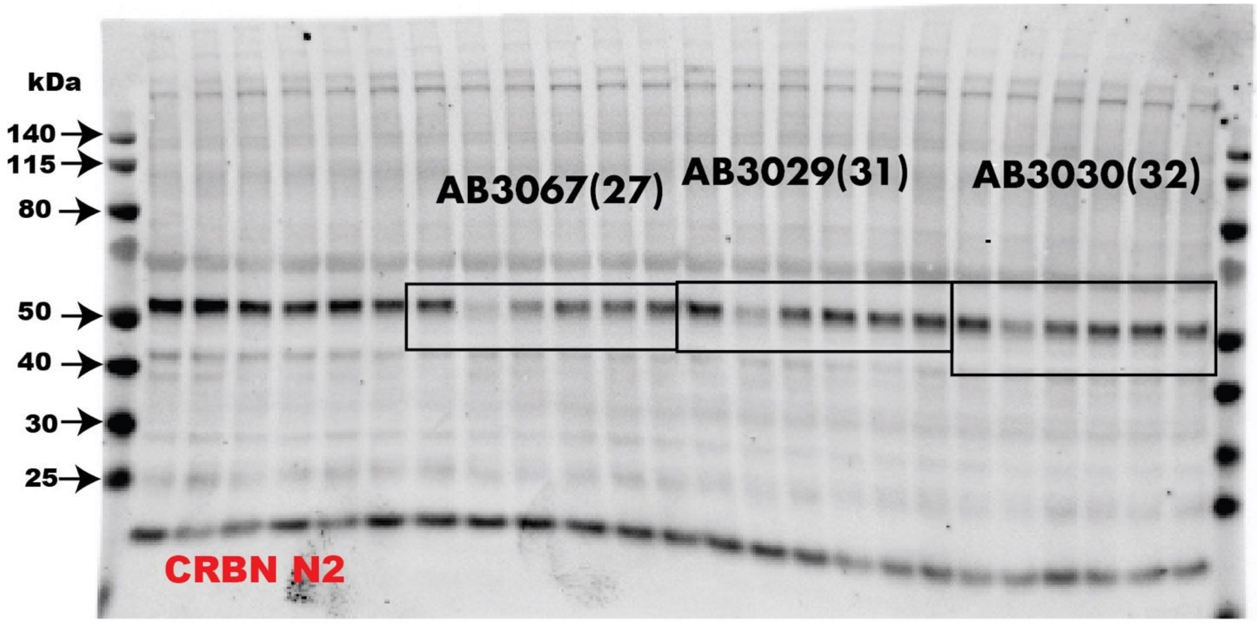

kDa
140
115
80
50
40
30
25
AB3067(27)
AB3029(31)
AB3030(32)
CRBN N2

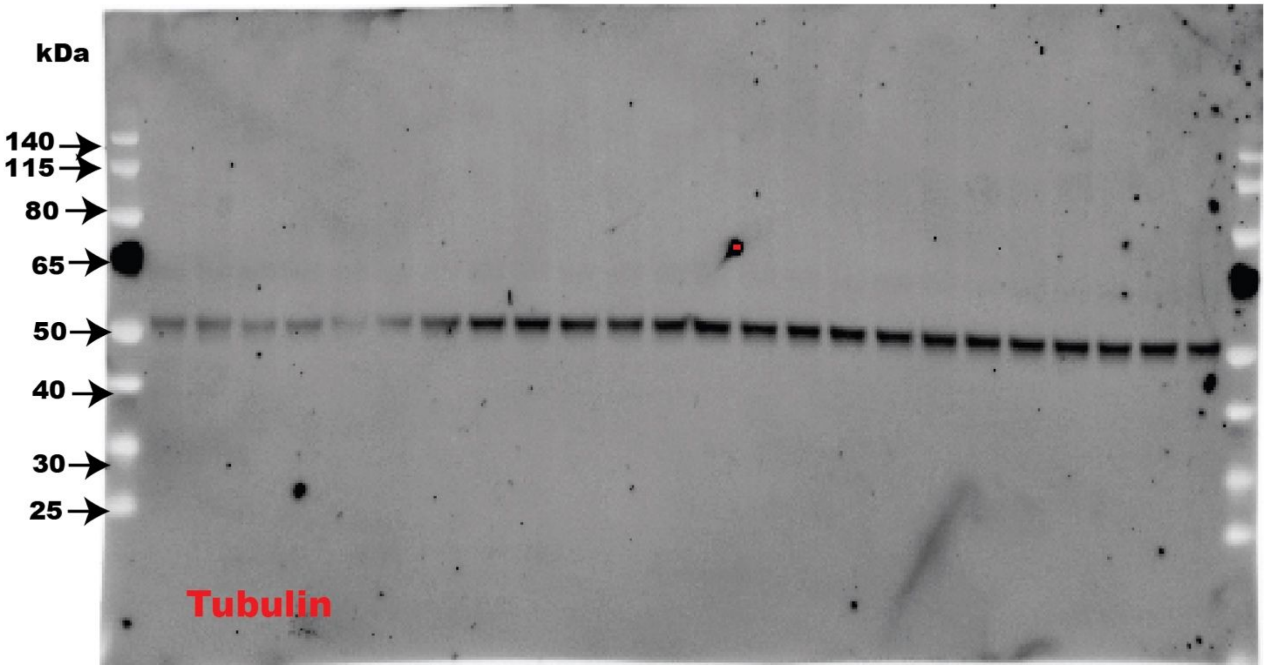

kDa
140
115
80
65
50
40
30
25
Tubulin

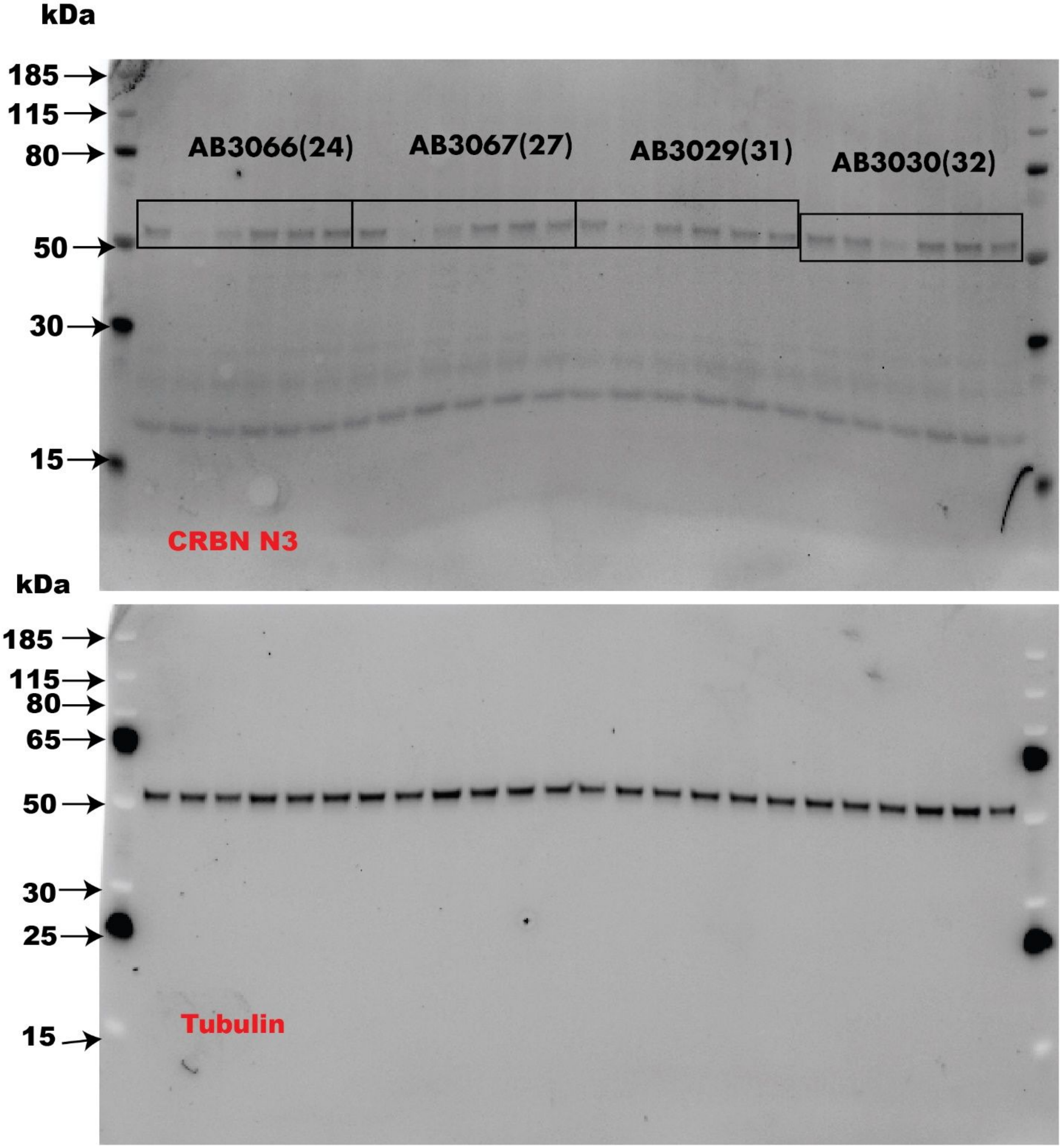
kDa
185
115
80
50
30
15
AB3066(24)
AB3067(27)
AB3029(31)
AB3030(32)
CRBN N3
kDa
185
115
80
65
50
30
25
15
Tubulin

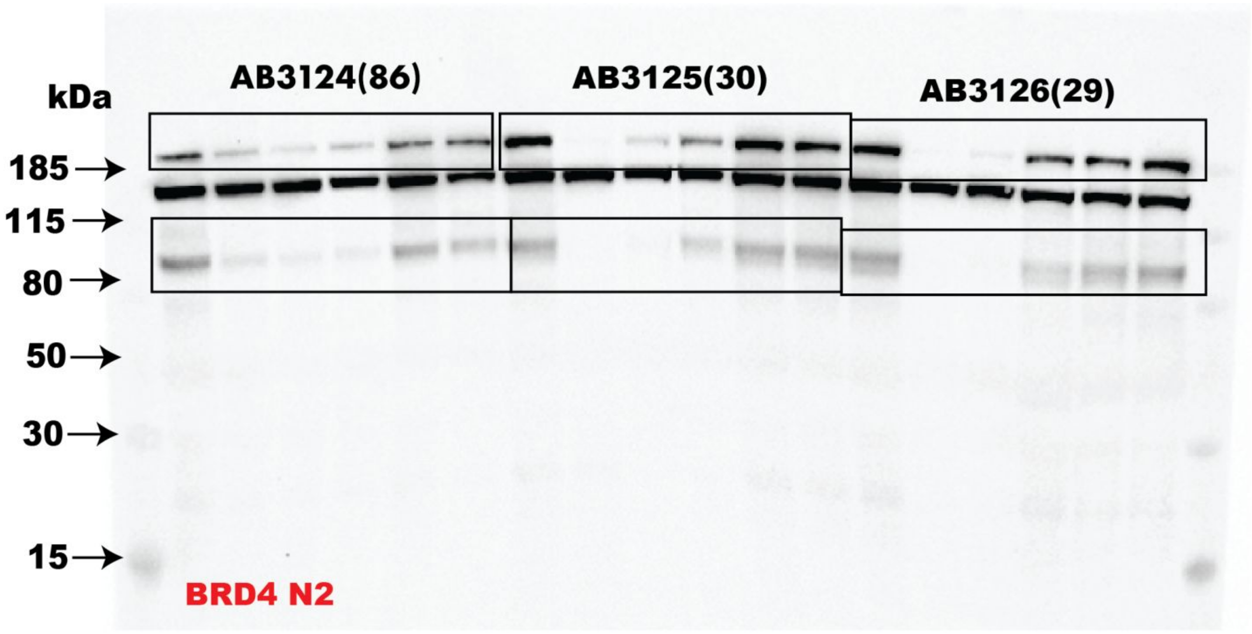
kDa
AB3124(86)
AB3125(30)
AB3126(29)
185
115
80
50
30
15
BRD4 N2

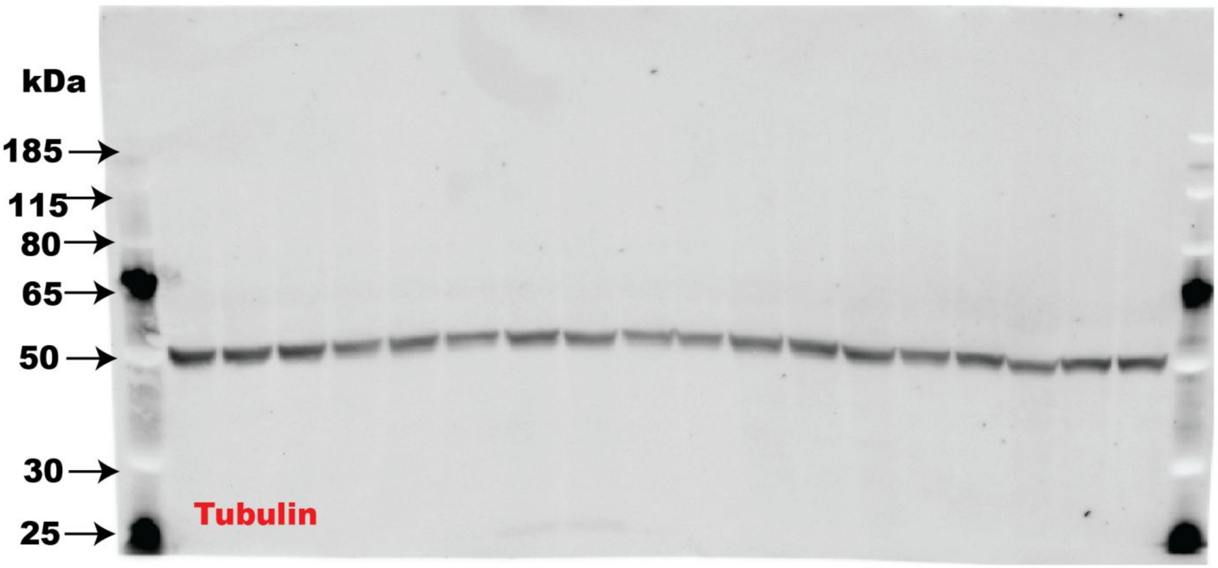
kDa
185
115
80
65
50
30
25
Tubulin

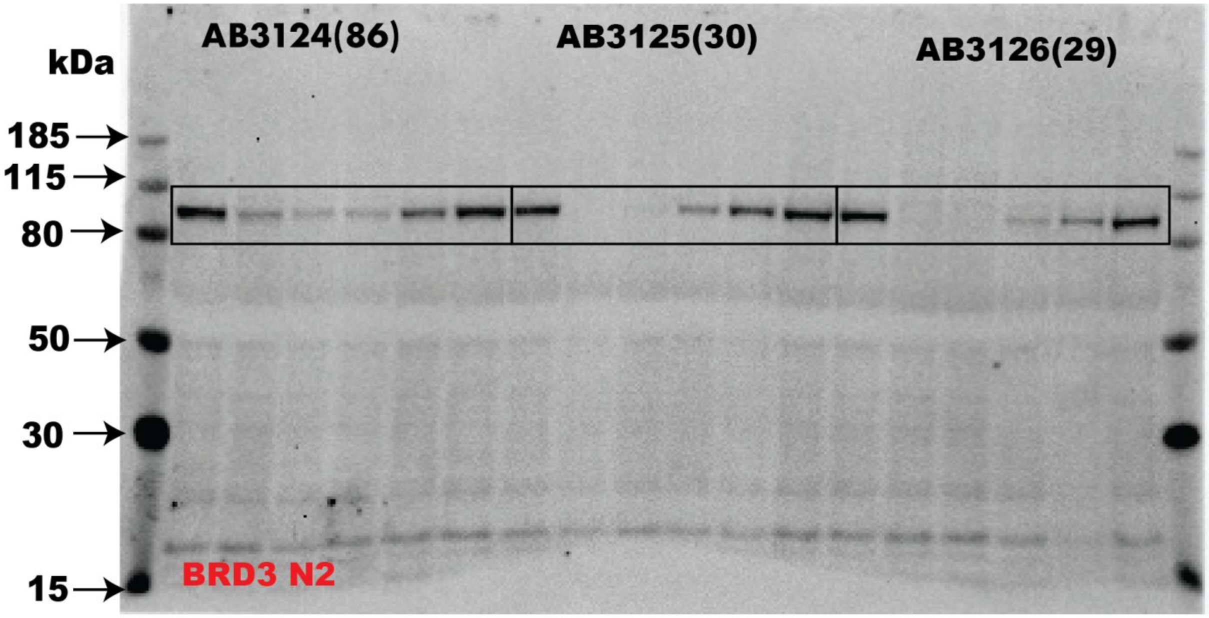
AB3124(86)
AB3125(30)
AB3126(29)
kDa
185
115
80
50
30
15
BRD3 N2

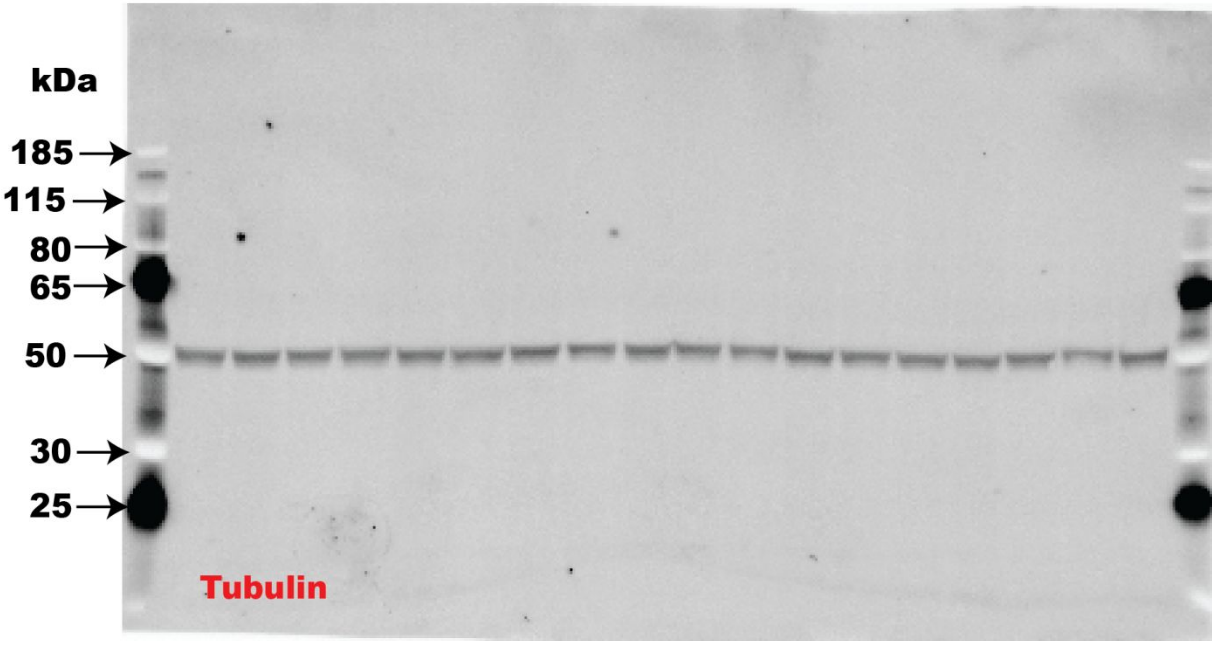
kDa
185
115
80
65
50
30
25
Tubulin

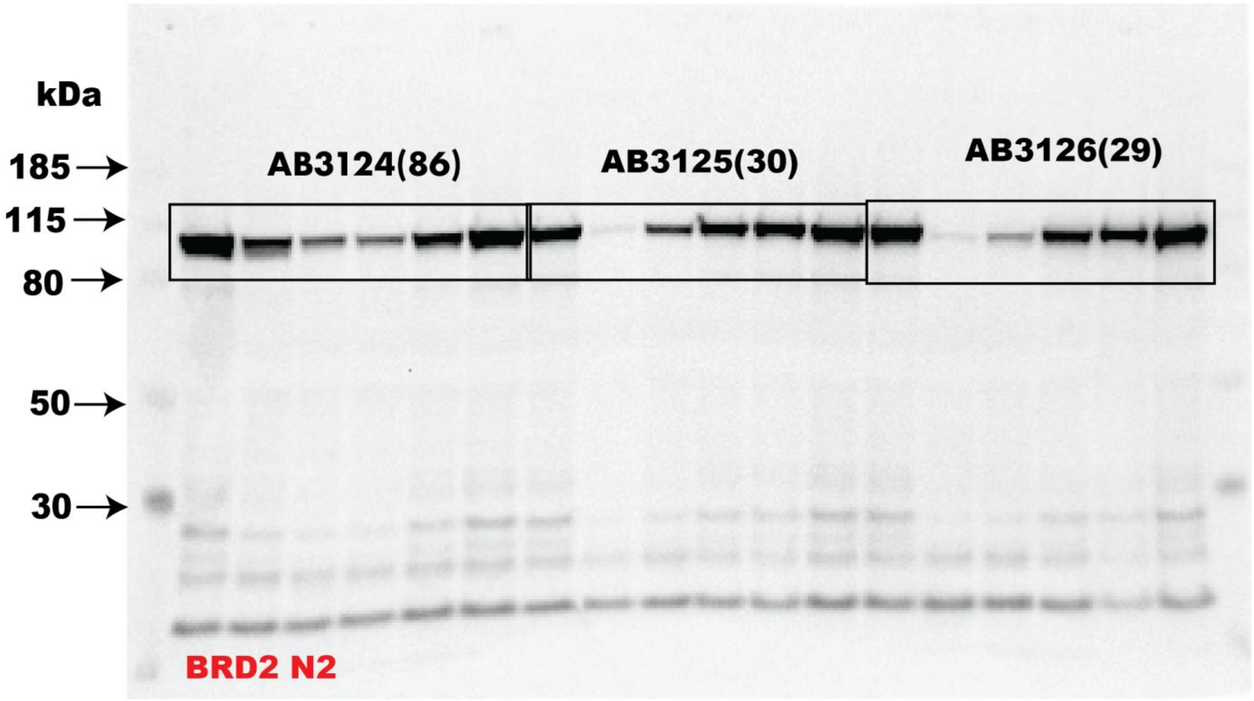
kDa
185
115
80
50
30
AB3124(86)
AB3125(30)
AB3126(29)
BRD2 N2

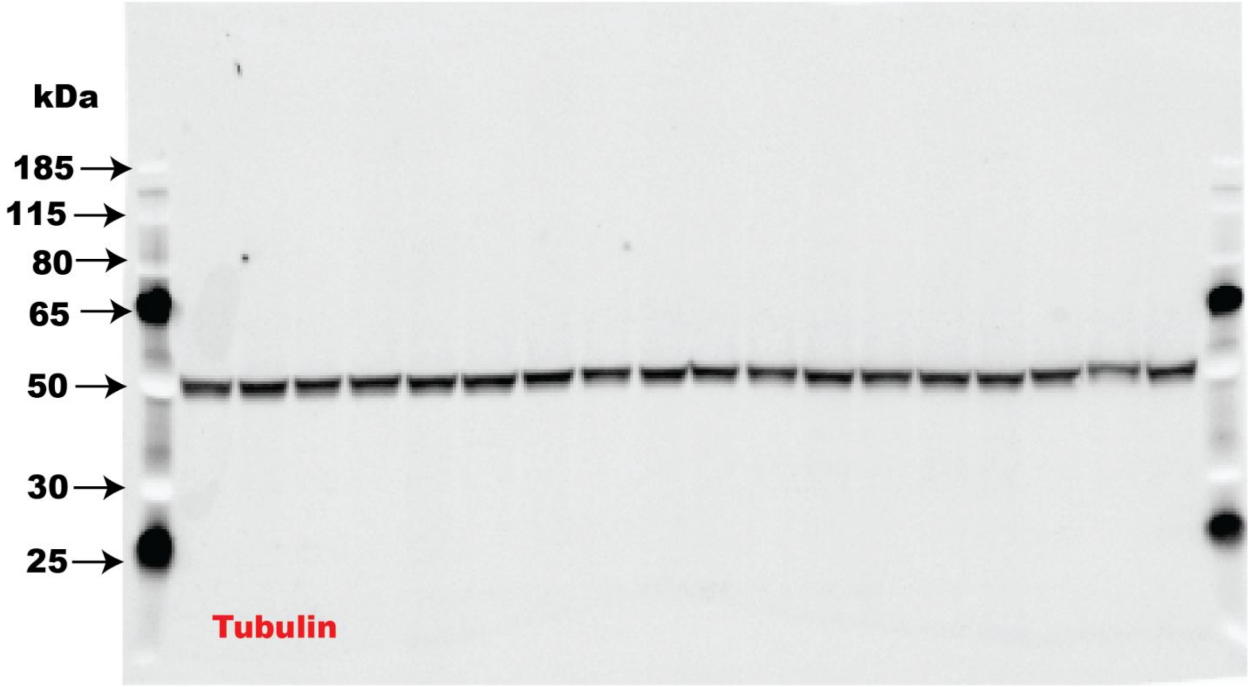
kDa
185
115
80
65
50
30
25
Tubulin

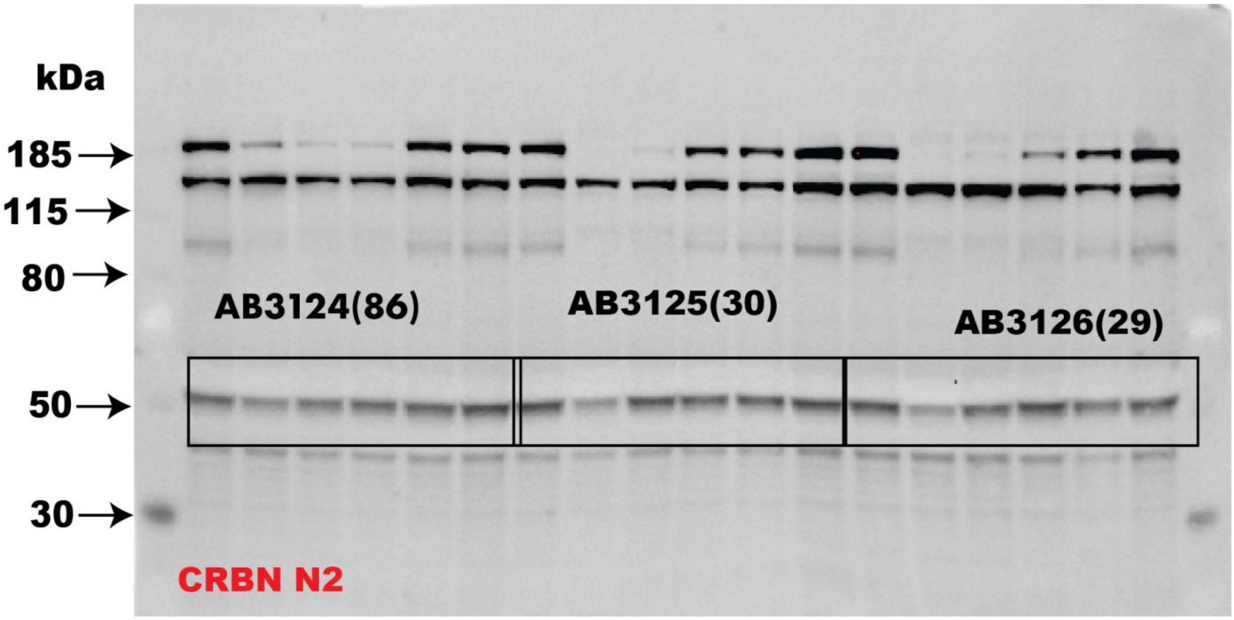

kDa
185
115
80
50
30
AB3124(86)
AB3125(30)
AB3126(29)
CRBN N2

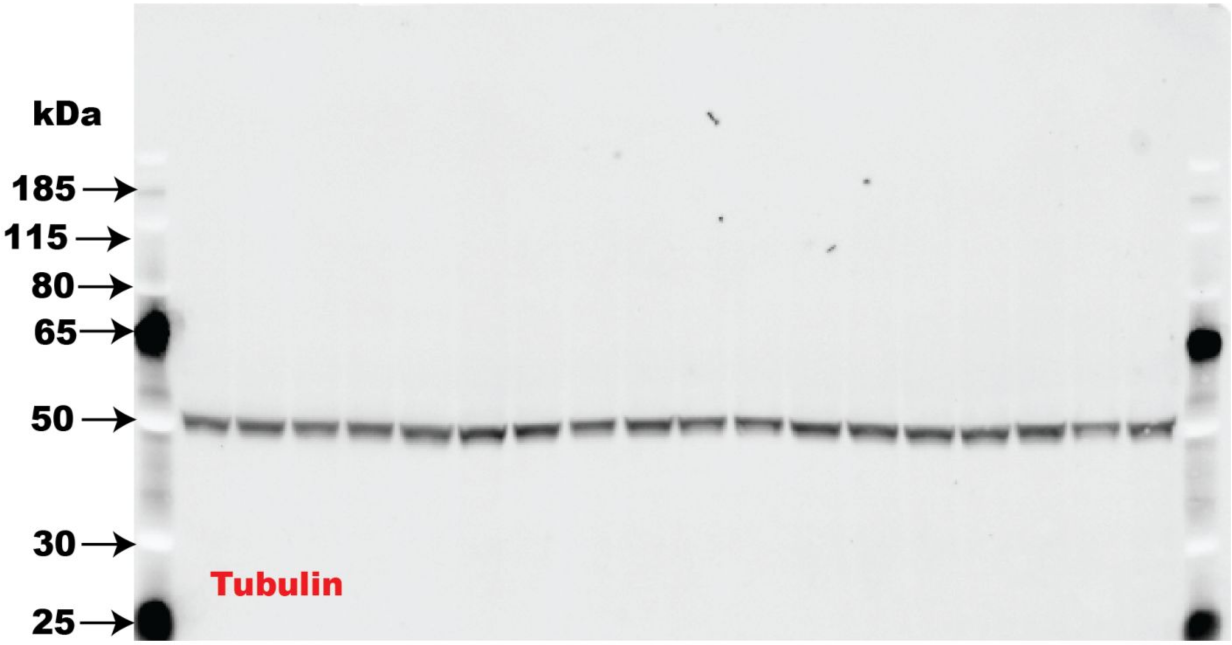

kDa
185
115
80
65
50
30
25
Tubulin

Supplementary Figure 10 (related to Figure 12). **Uncropped independent replicate western blotting images of the evaluation of heterotrivalent AB3145 (97) & AGB1 in homozygous BromoTag-BRD4 HEK293 cells.**

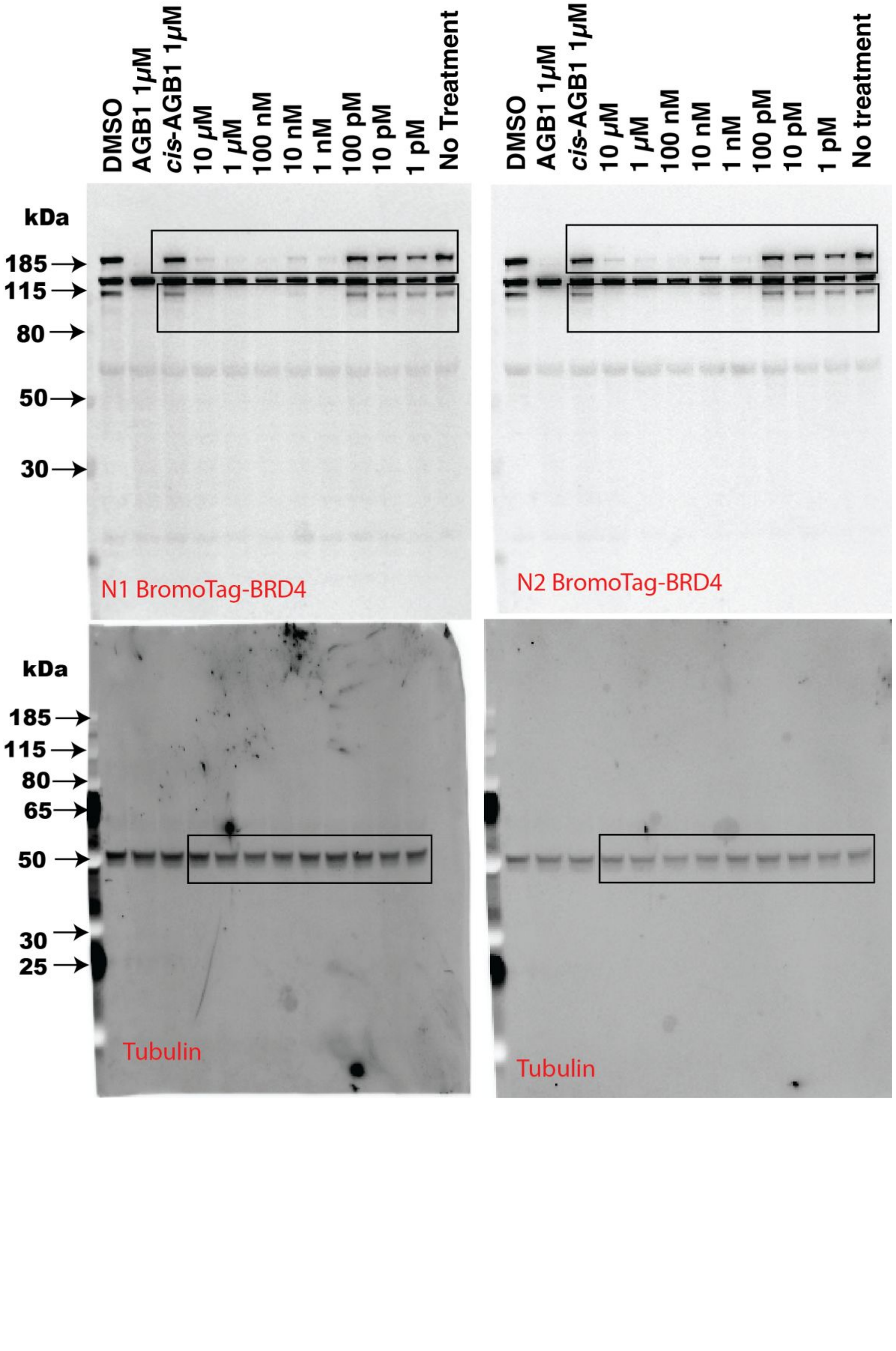

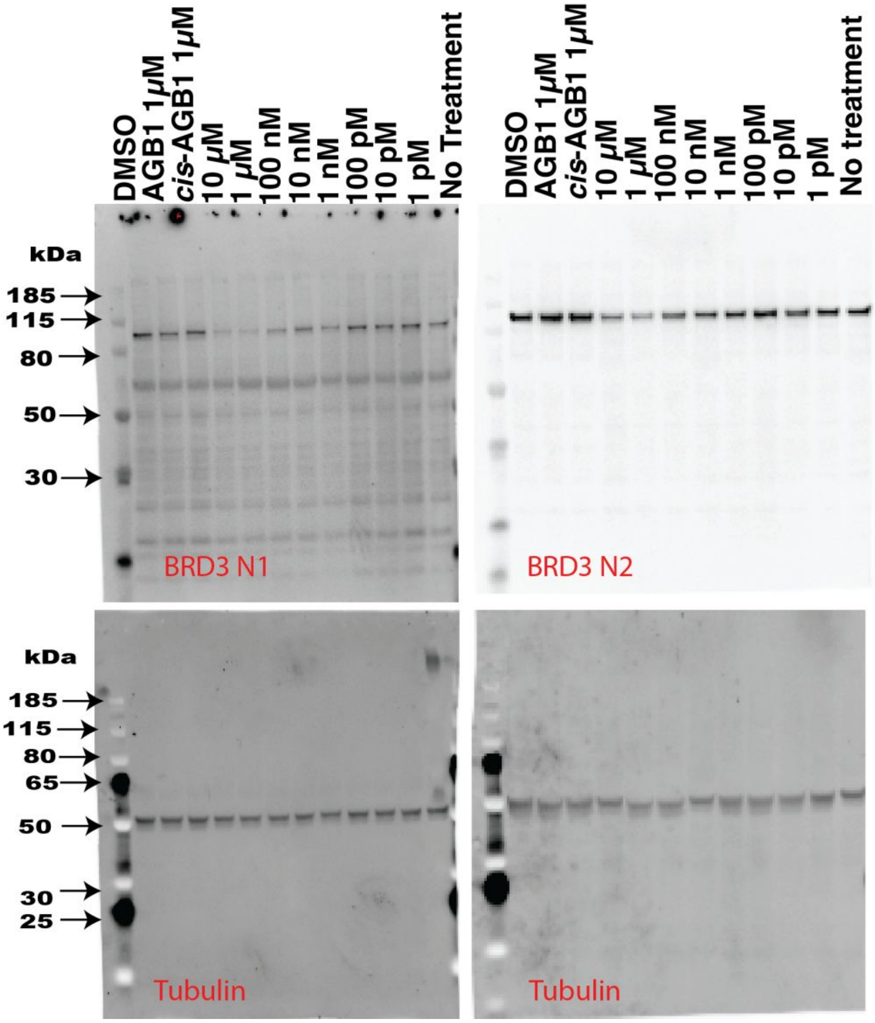
DMSO
AGB1 1μM
cis-AGB1 1μM
10 μM
1 μM
100 nM
10 nM
1 nM
100 pM
10 pM
1 pM
No Treatment
DMSO
AGB1 1μM
cis-AGB1 1μM
10 μM
1 μM
100 nM
10 nM
1 nM
100 pM
10 pM
1 pM
No treatment
kDa
185
115
80
50
30
BRD3 N1
BRD3 N2
kDa
185
115
80
65
50
30
25
Tubulin
Tubulin

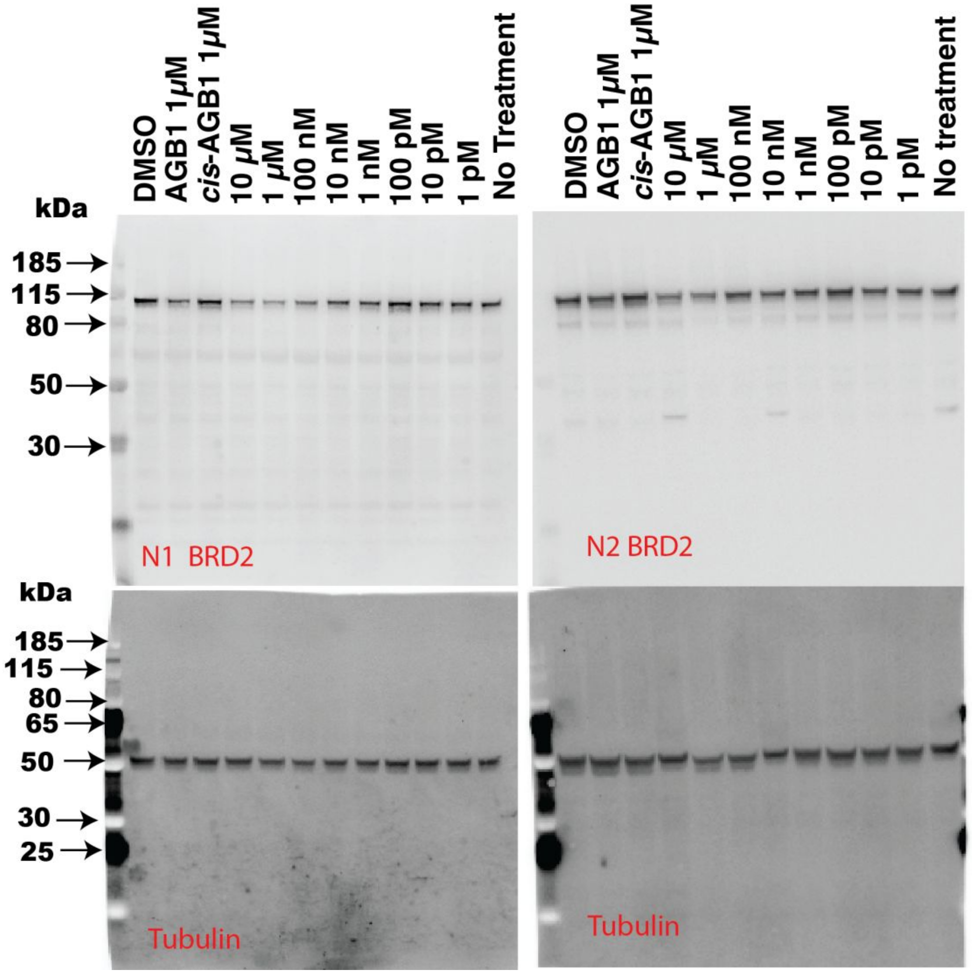
DMSO
AGB1 1μM
cis-AGB1 1μM
10 μM
1 μM
100 nM
10 nM
1 nM
100 pM
10 pM
1 pM
No Treatment
DMSO
AGB1 1μM
cis-AGB1 1μM
10 μM
1 μM
100 nM
10 nM
1 nM
100 pM
10 pM
1 pM
No treatment
kDa
185
115
80
50
30
N1 BRD2
N2 BRD2
kDa
185
115
80
65
50
30
25
Tubulin
Tubulin

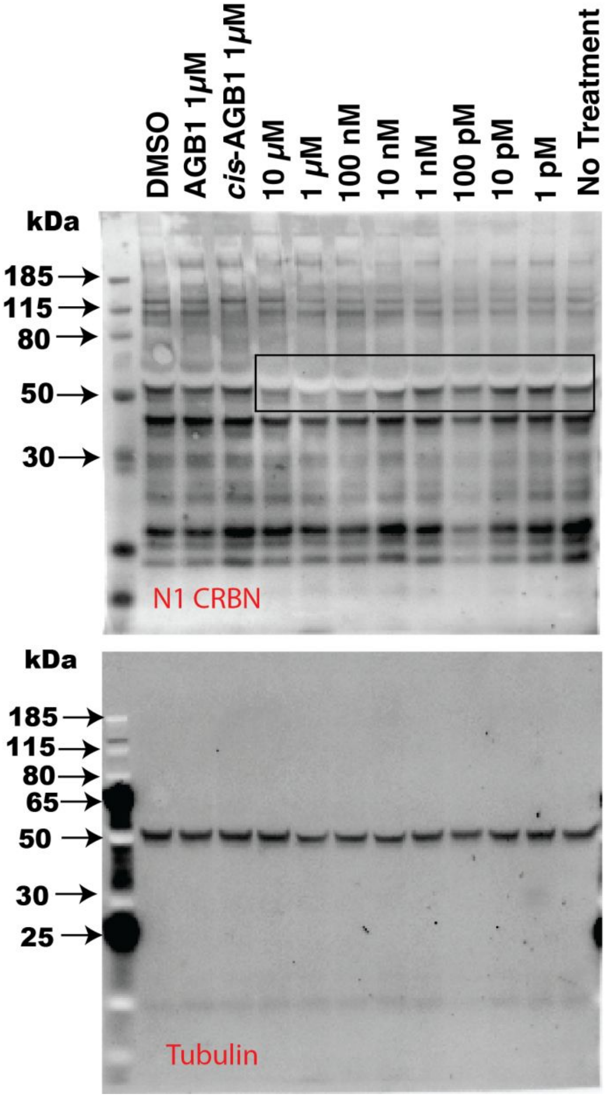
DMSO
AGB1 1μM
cis-AGB1 1μM
10 μM
1 μM
100 nM
10 nM
1 nM
100 pM
10 pM
1 pM
No Treatment
kDa
185
115
80
50
30
N1 CRBN
kDa
185
115
80
65
50
30
25
Tubulin

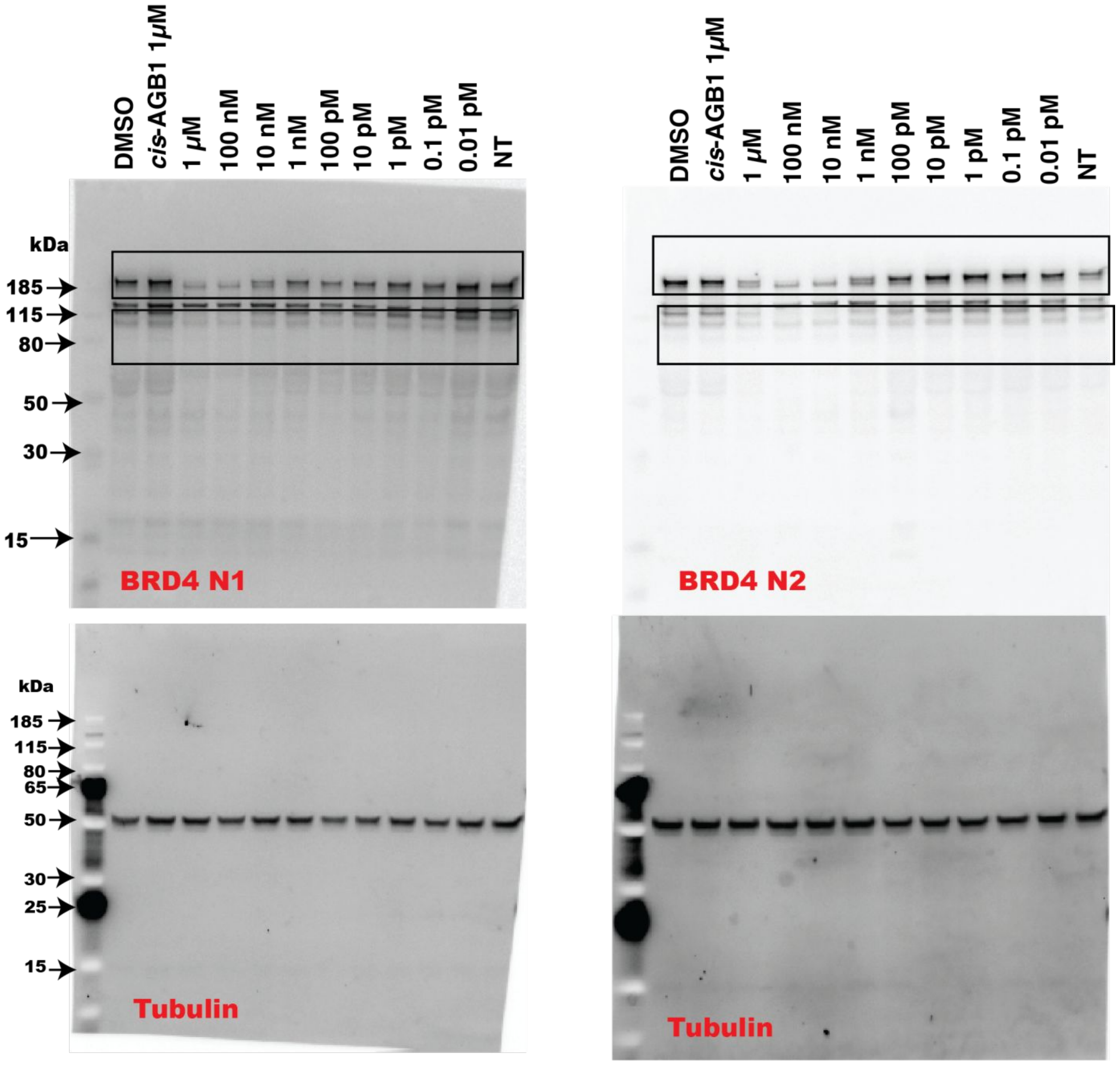
DMSO
cis-AGB1 1μM
1 μM
100 nM
10 nM
1 nM
100 pM
10 pM
1 pM
0.1 pM
0.01 pM
NT
kDa
185
115
80
50
30
15
BRD4 N1
BRD4 N2
kDa
185
115
80
65
50
30
25
15
Tubulin
Tubulin

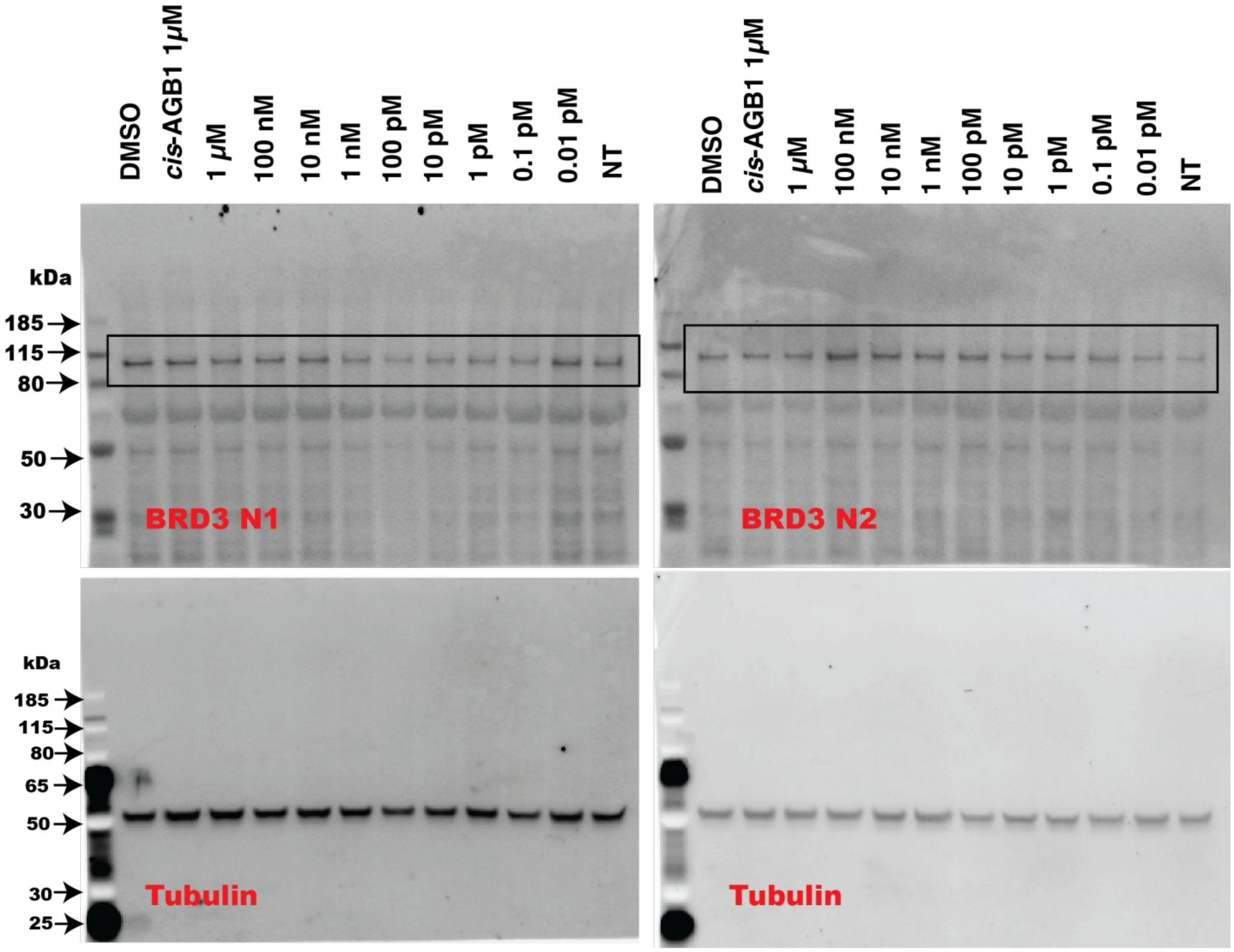
DMSO
cis-AGB1 1 μM
1 μM
100 nM
10 nM
1 nM
100 pM
10 pM
1 pM
0.1 pM
0.01 pM
NT
kDa
185
115
80
50
30
BRD3 N1
BRD3 N2
65
25
Tubulin

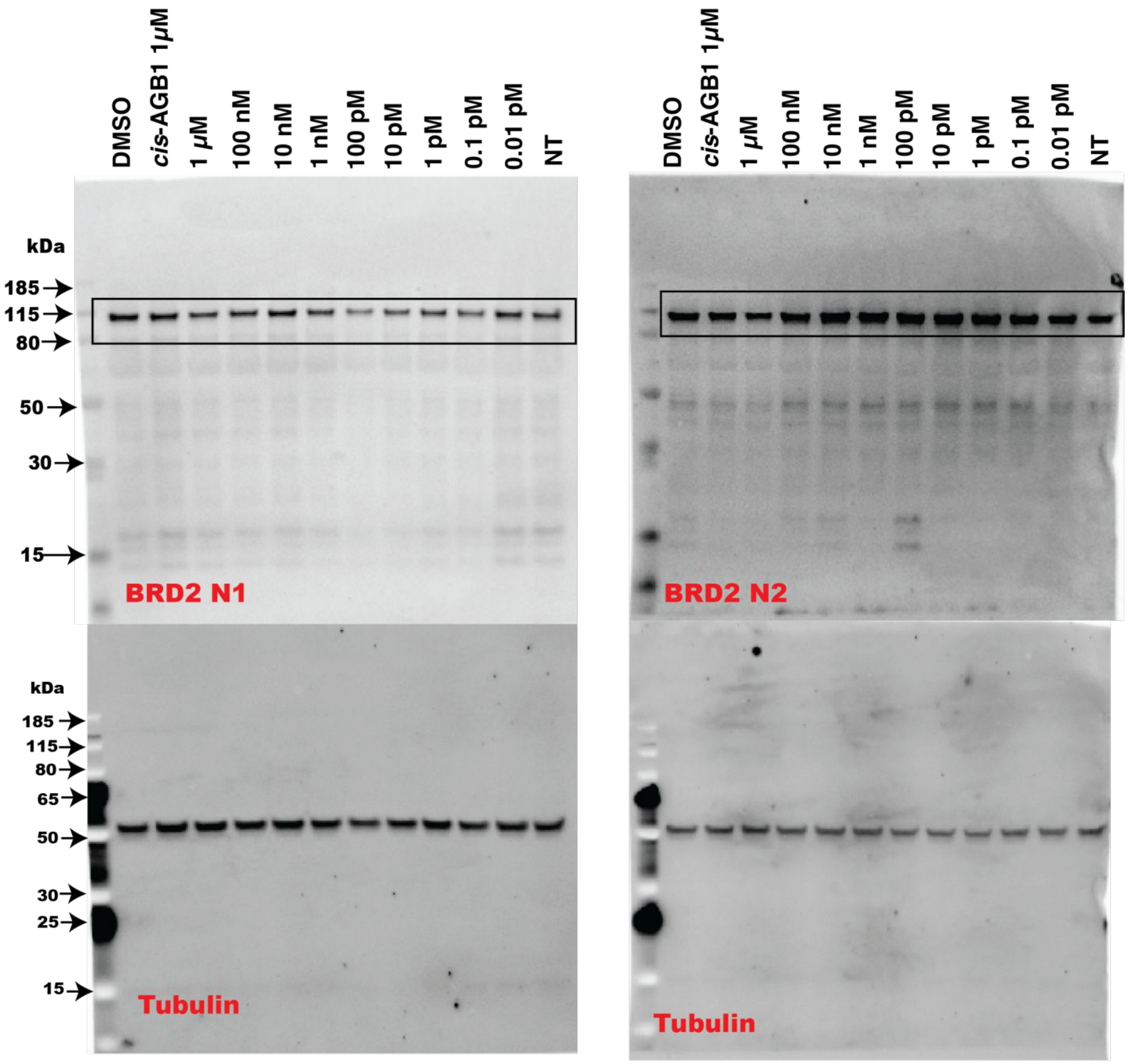
DMSO
cis-AGB1 1μM
1 μM
100 nM
10 nM
1 nM
100 pM
10 pM
1 pM
0.1 pM
0.01 pM
NT
kDa
185
115
80
50
30
15
BRD2 N1
DMSO
cis-AGB1 1μM
1 μM
100 nM
10 nM
1 nM
100 pM
10 pM
1 pM
0.1 pM
0.01 pM
NT
BRD2 N2
kDa
185
115
80
65
50
30
25
15
Tubulin
Tubulin

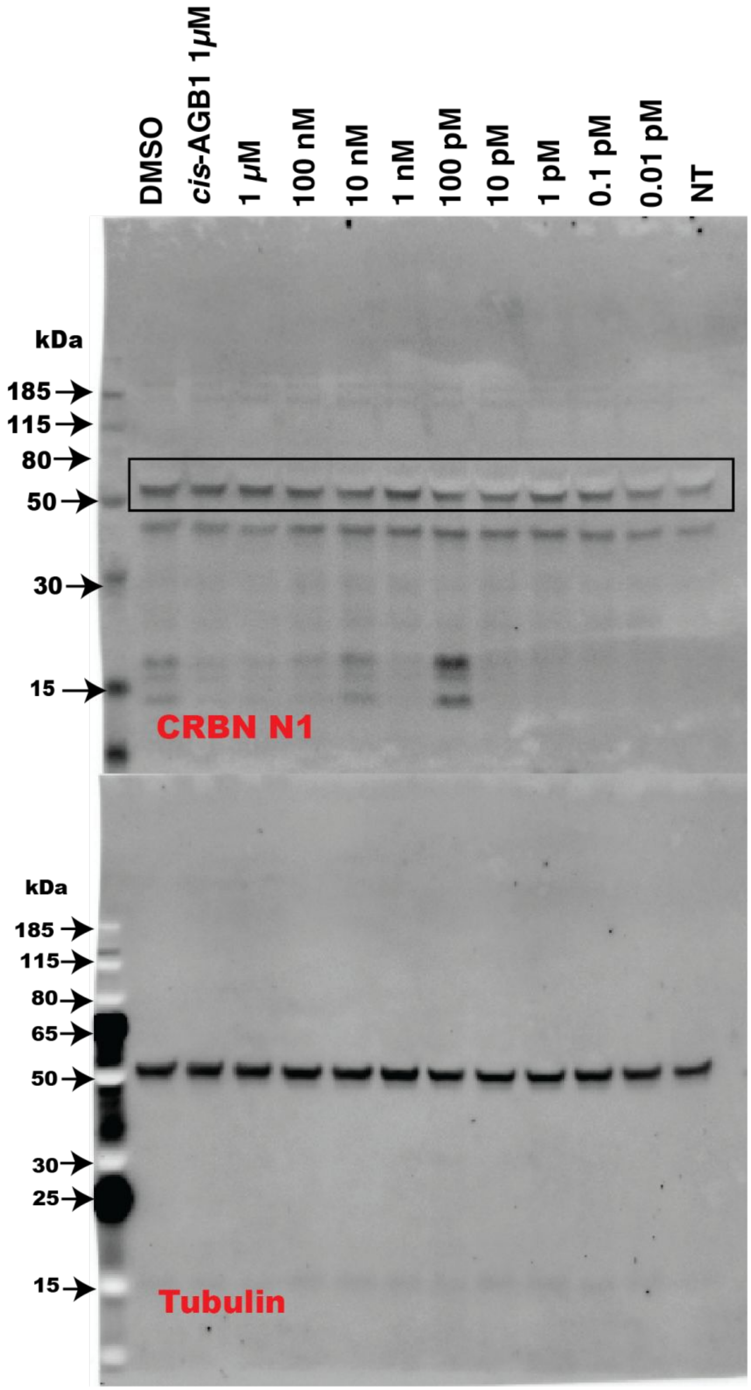
DMSO
cis-AGB1 1μM
1 μM
100 nM
10 nM
1 nM
100 pM
10 pM
1 pM
0.1 pM
0.01 pM
NT
kDa
185
115
80
50
30
15
CRBN N1
kDa
185
115
80
65
50
30
25
15
Tubulin

Supplementary Figure 11 (related to Figure 12)**. Cropped western blotting images of the evaluation of heterotrivalent AB3145 (97) and AGB1 in homozygous BromoTag-BRD4 HEK293 cells.**

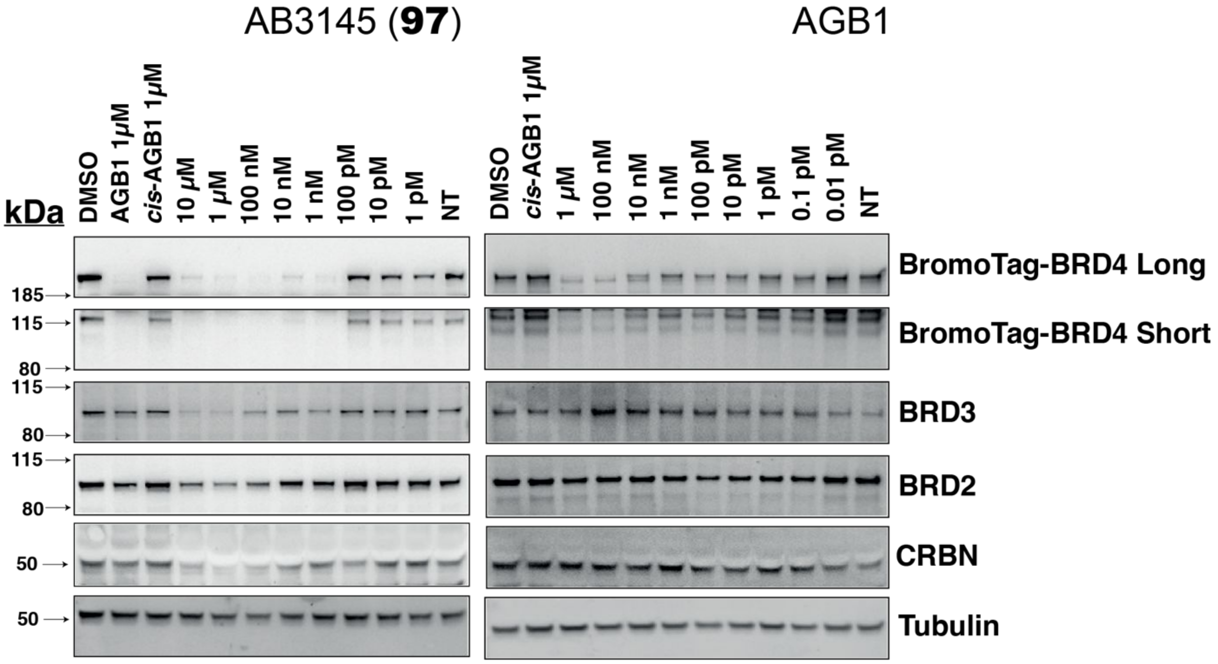

Supplementary Table 8 (related to Figure 12)**. DC_{50} values of AB3145 (97) and AGB1 in homozygous BromoTag-BRD4 HEK293 cells.**

	AB3145 (97)		AGB1	
**Protein**	**pDC_{50}**	**D_{max} %**	**pDC_{50}**	**D_{max} %**
**$BRD4^{Long}$**	**9.9 ± 0.6**	**14**	**8.0 ± 0.2**	**4**
**$BRD4^{Short}$**	**9.9 ± 0.1**	**14**	**8.6 ± 0.1**	**11**
**BRD3**	**7.5 ± 0.4**	**21**	**N.A.**	**N.A.**
**BRD2**	**6.5[a]**	**56**	**N.A.**	**N.A.**
**CRBN**	**7.7[b]**	**23**	**N.A.**	**N.A.**

[a] Unable to produce a meaningful S.E. due to an incomplete curve for one independent repeat. [b] S.E. unavailable due to N=1 repeat.

HPLC/HRMS Traces for Compounds 1, 2, 23 – 32, 86, 93 – 95 and 97.

MN666 (**1**)

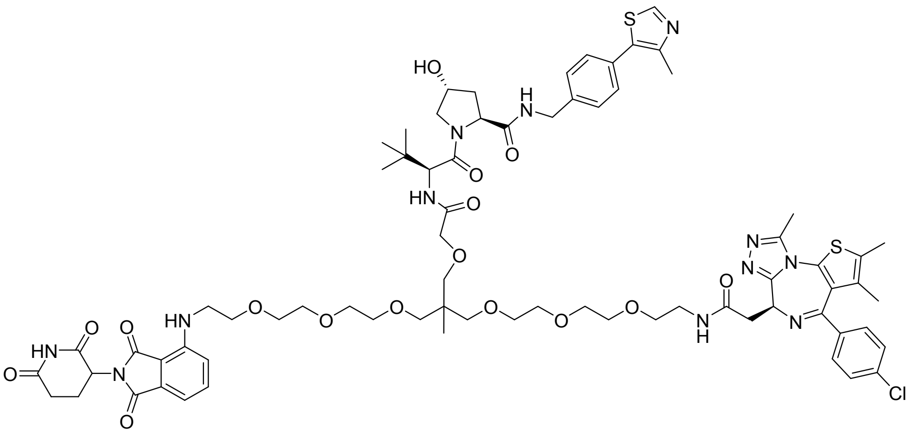

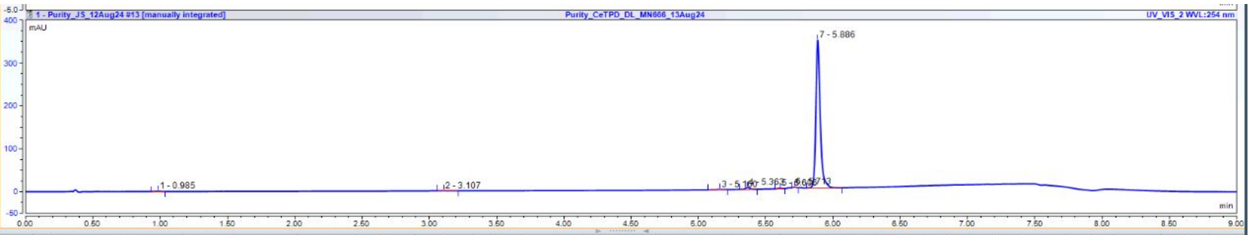

Peak No.	Peak Name	Ret.Time min	Amount n.a.	Rel.Area %	Area mAU*min	Height mAU	Type	Width (50%) min	Asym. EP	Resol. EP	Plates EP
1		0.985	n.a.	0.17	0.0231	0.47	BMB	0.047	1.09	28.23	2398
2		3.107	n.a.	0.14	0.0187	0.35	BMB	0.041	1.76	27.13	31304
3		5.160	n.a.	0.22	0.0286	0.52	BMB	0.048	0.77	2.94	64217
4		5.363	n.a.	1.21	0.1598	4.16	BMB	0.034	1.23	4.45	141009
5		5.606	n.a.	0.24	0.0318	0.98	BMB	0.031	1.06	2.33	184970
6		5.713	n.a.	0.33	0.0443	1.81	BMB	0.024	1.04	3.64	322919
7		5.886	n.a.	97.69	12.9518	345.26	BMB	0.032	1.35	n.a.	183976
Maximum			0.0000	97.69	12.9518	345.26		0.048	1.76	28.23	322919
Minimum			0.0000	0.14	0.0187	0.35		0.024	0.77	2.33	2398
Sum			0.0000	100.00	13.2580	353.56					

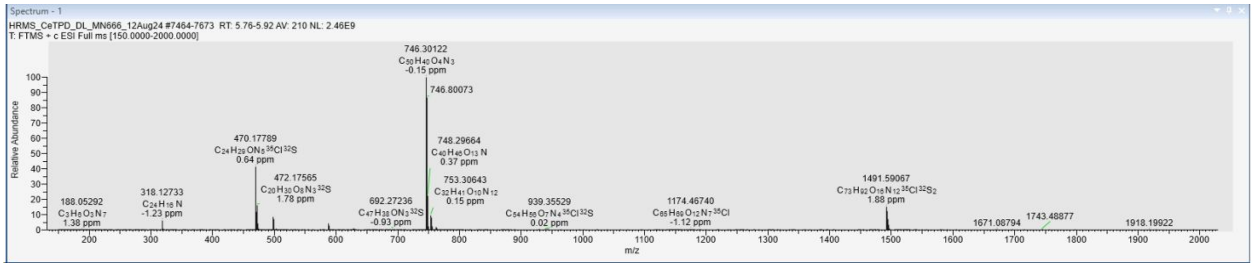

MN675 (**2**)

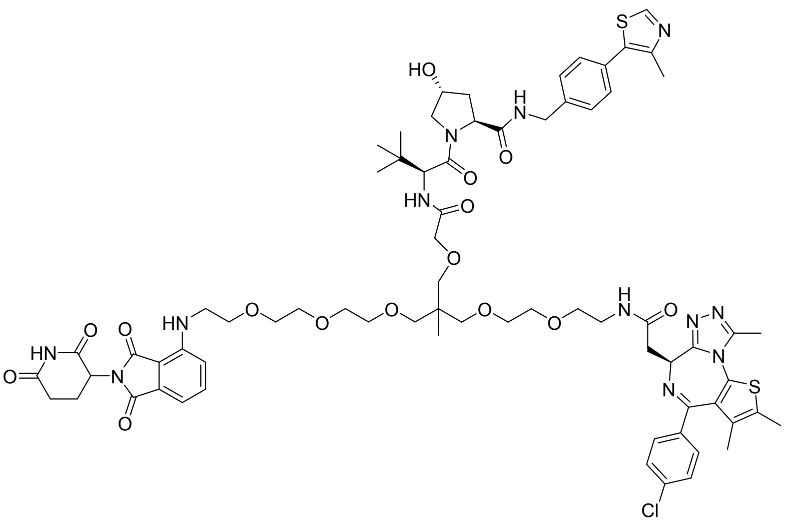

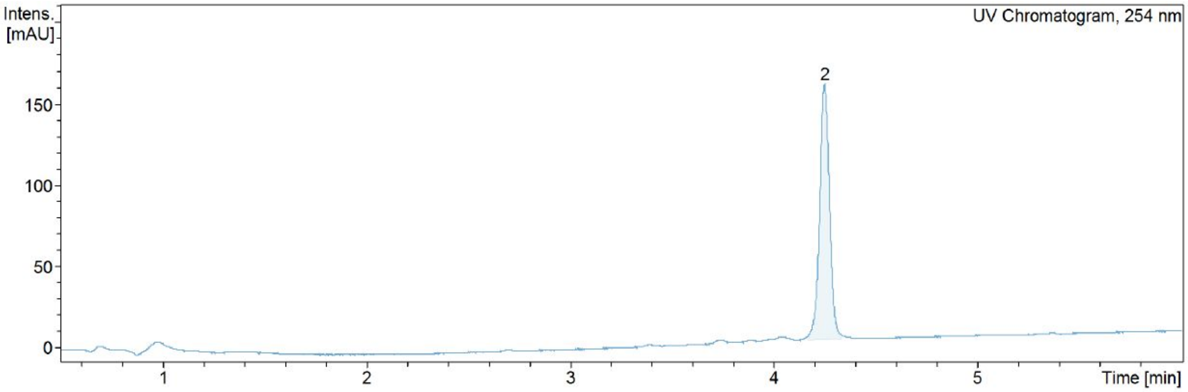

#	RT [min]	Area Frac. %	Chromatogram
1	4.1	9.80	BPC 149.0000-3000.0000 +, Masses excluded
2	4.2	100.00	UV Chromatogram, 254 nm
3	4.3	90.20	BPC 149.0000-3000.0000 +, Masses excluded

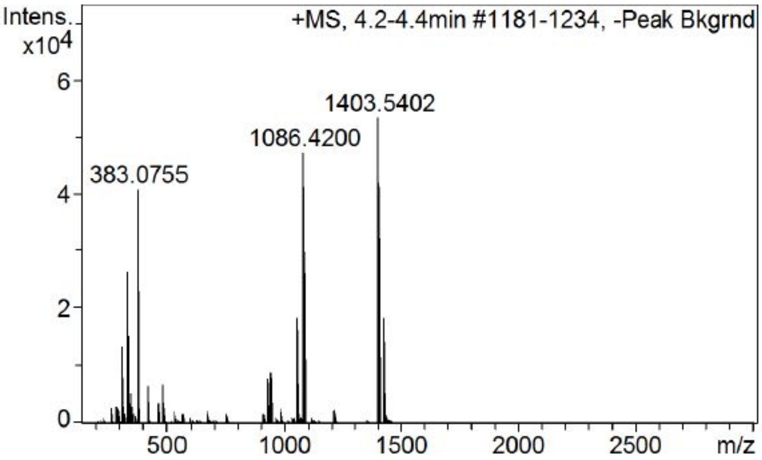

AB3062 (**23**)

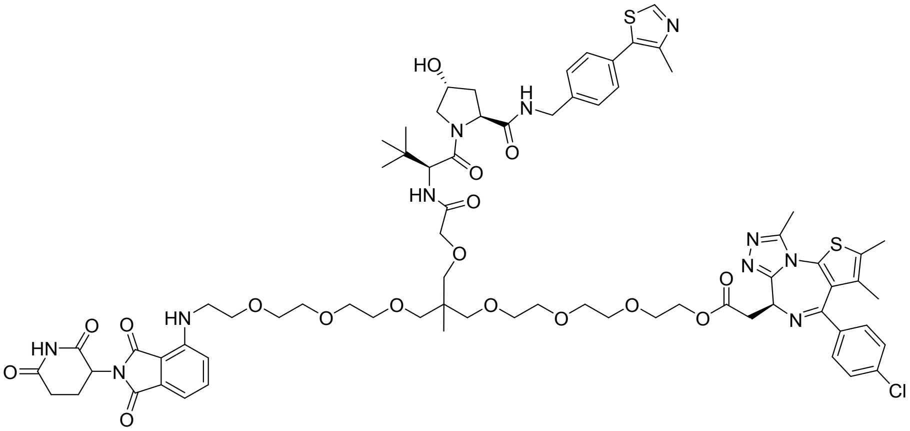

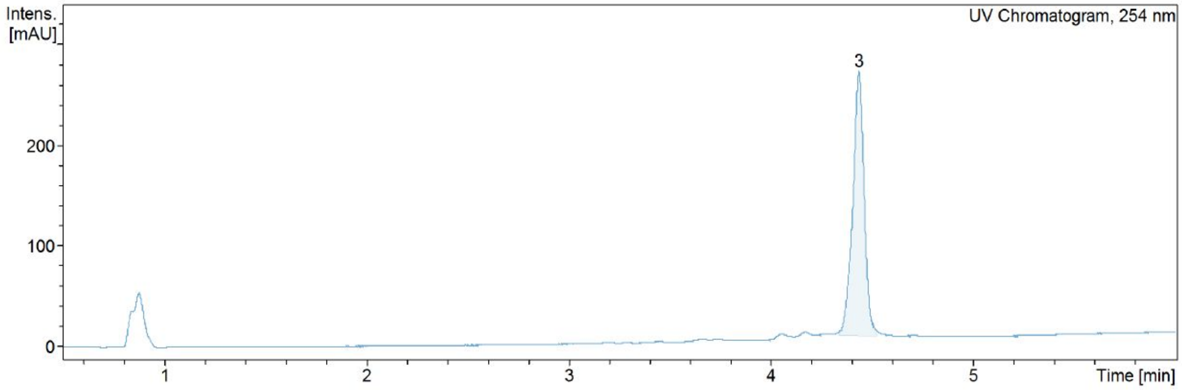

#	RT [min]	Area Frac. %	Chromatogram
1	4.1	10.39	BPC 149.0000-3001.0000 +, Masses excluded
2	4.2	12.77	BPC 149.0000-3001.0000 +, Masses excluded
3	4.4	100.00	UV Chromatogram, 254 nm
4	4.5	76.84	BPC 149.0000-3001.0000 +, Masses excluded

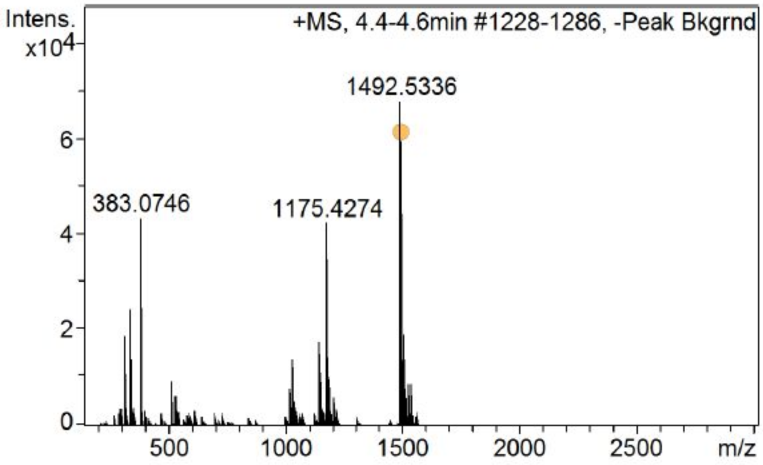

AB3066 (**24**)

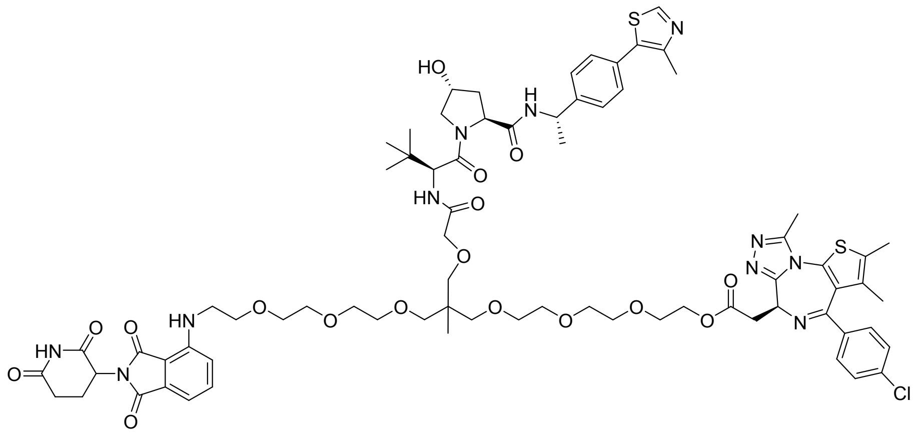

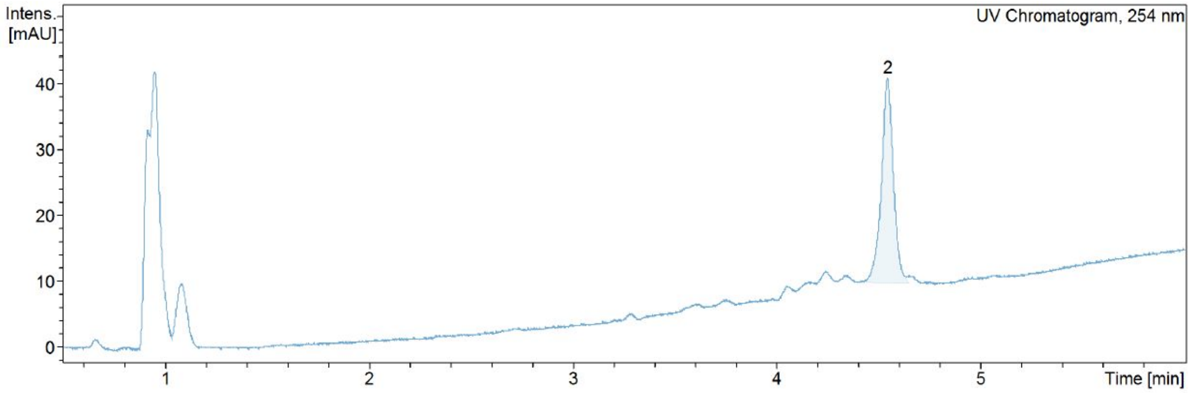

#	RT [min]	Area Frac. %	Chromatogram
1	4.1	12.52	BPC 149.0000-3001.0000 +, Masses excluded
2	4.5	100.00	UV Chromatogram, 254 nm
3	4.6	87.48	BPC 149.0000-3001.0000 +, Masses excluded

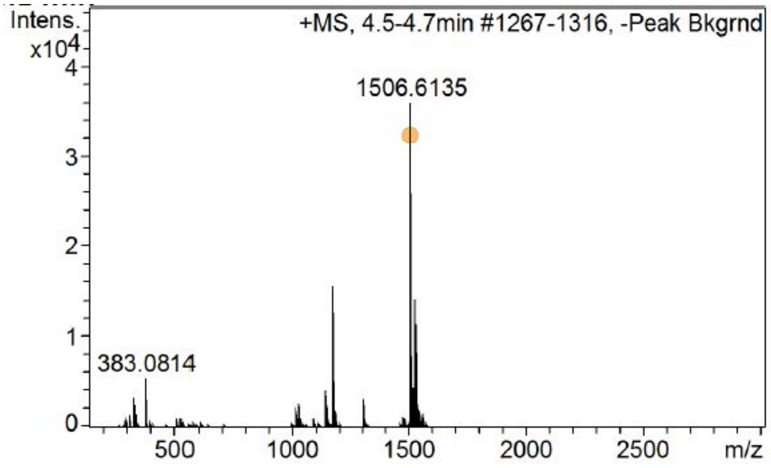

AB3064 (**25**)

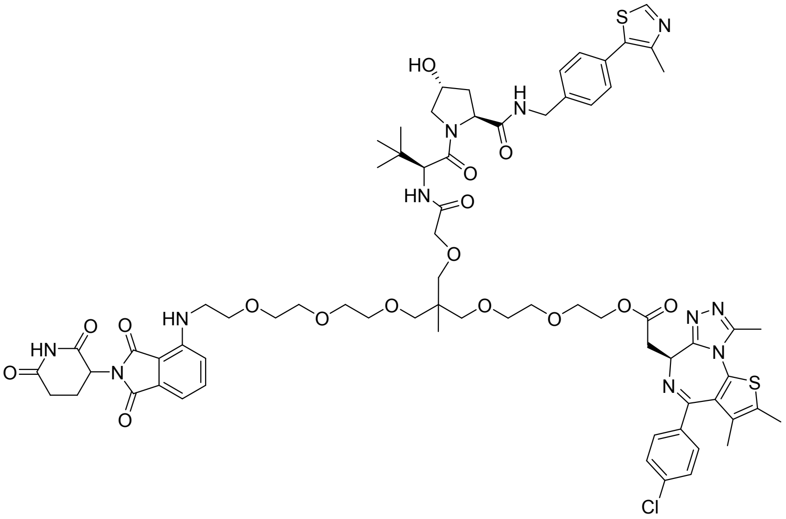

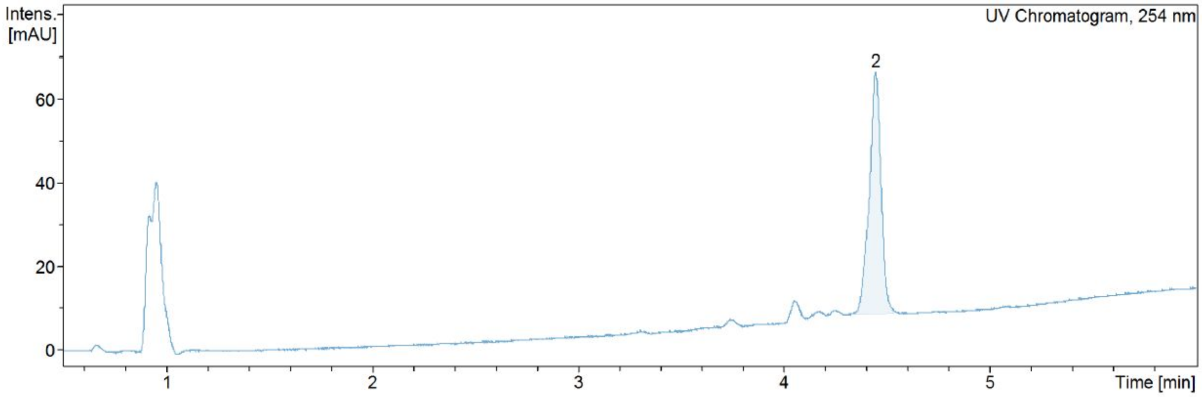

#	RT [min]	Area Frac. %	Chromatogram
1	4.1	27.74	BPC 149.0000-3001.0000 +, Masses excluded
2	4.4	100.00	UV Chromatogram, 254 nm
3	4.5	72.26	BPC 149.0000-3001.0000 +, Masses excluded

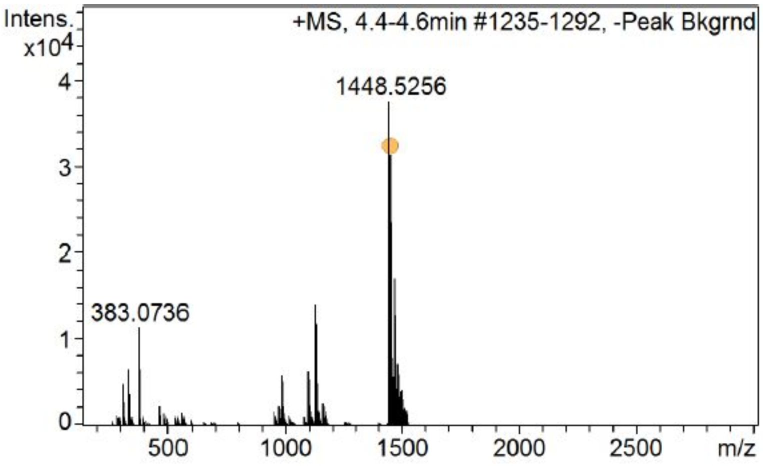

AB3063 (**26**)

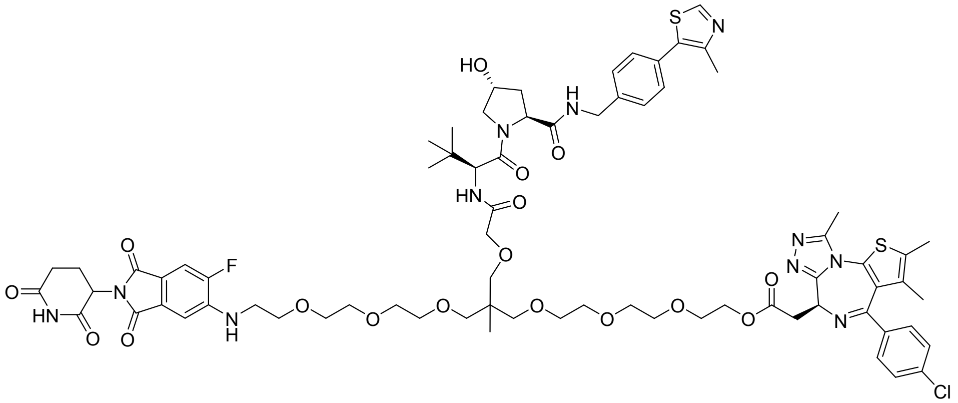

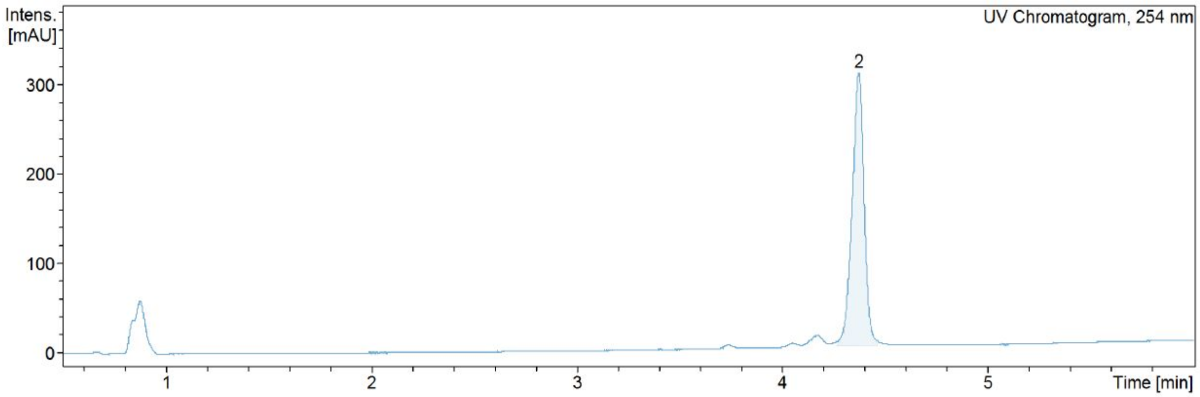

#	RT [min]	Area Frac. %	Chromatogram
1	4.1	10.77	BPC 149.0000-3001.0000 +, Masses excluded
2	4.4	100.00	UV Chromatogram, 254 nm
3	4.4	89.23	BPC 149.0000-3001.0000 +, Masses excluded

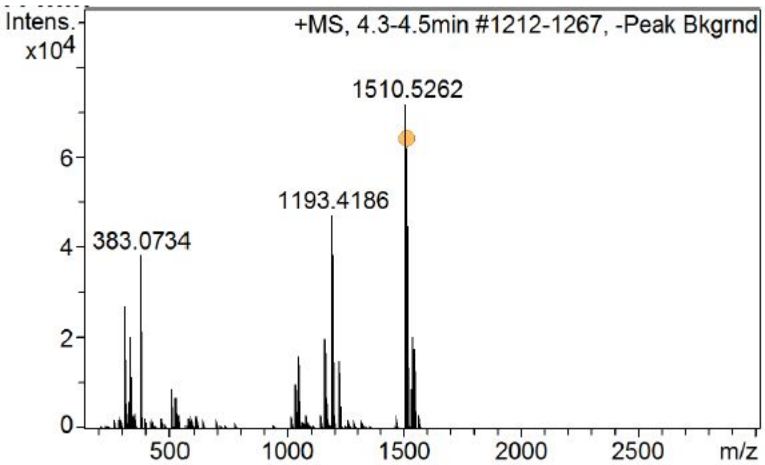

AB3067 (**27**)

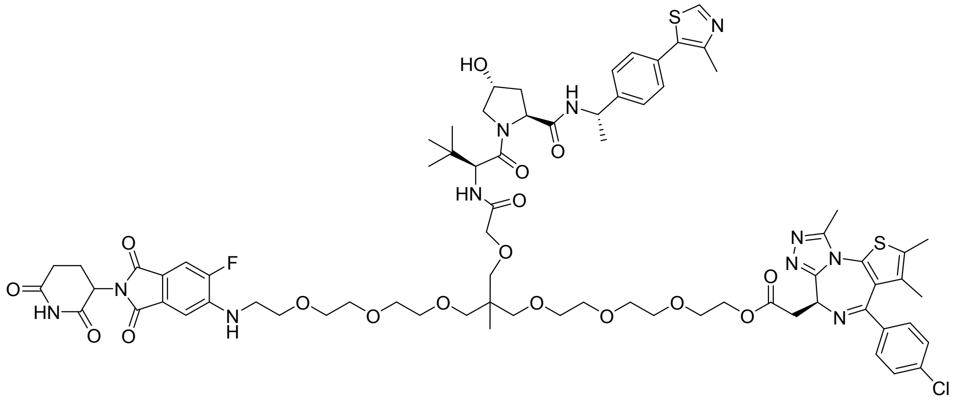

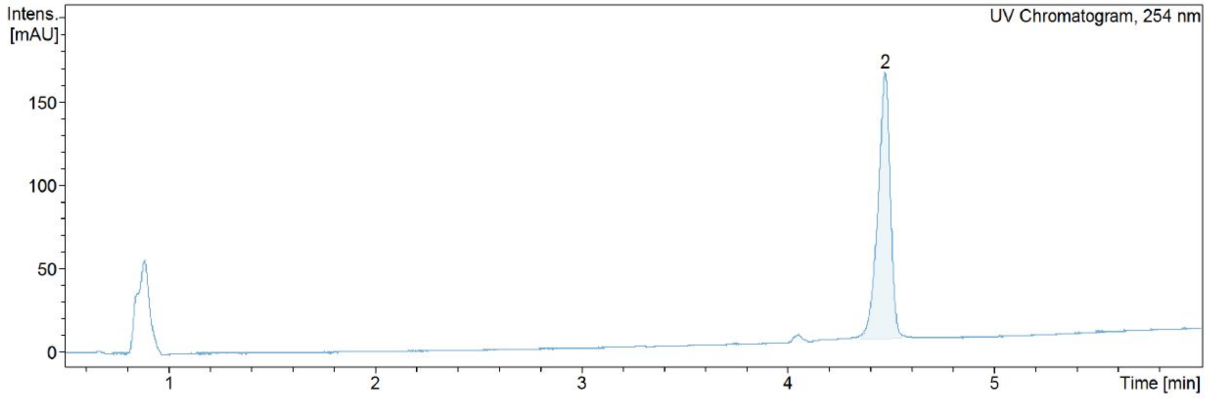

#	RT [min]	Area Frac. %	Chromatogram
1	4.1	16.07	BPC 149.0000-3001.0000 +, Masses excluded
2	4.5	100.00	UV Chromatogram, 254 nm
3	4.5	83.93	BPC 149.0000-3001.0000 +, Masses excluded

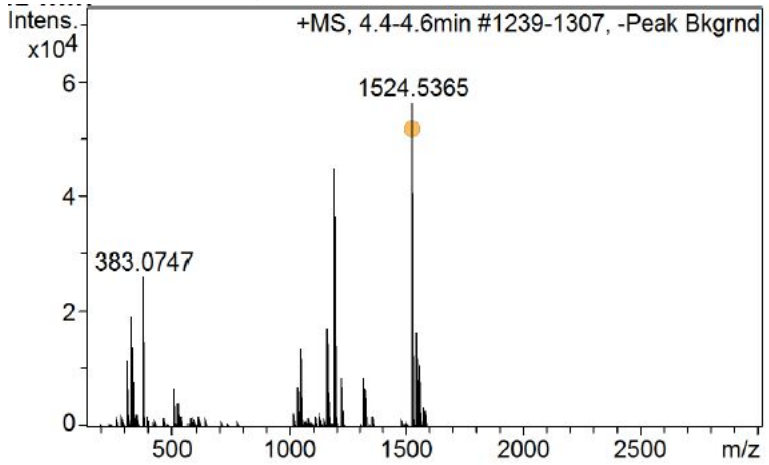

AB3065 (**28**)

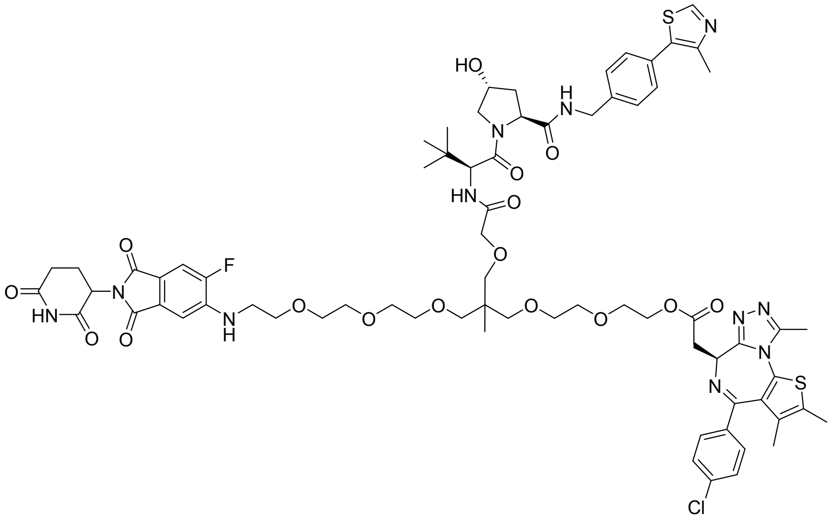

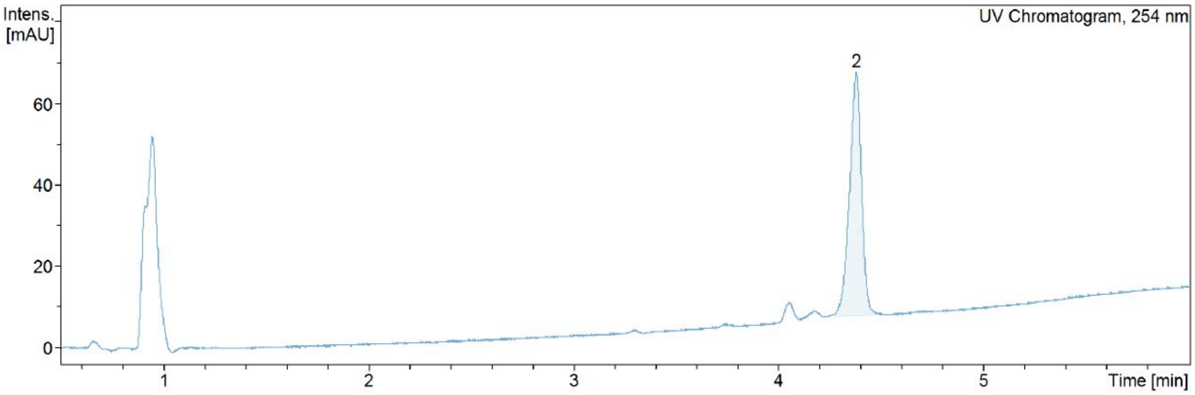

#	RT [min]	Area Frac. %	Chromatogram
1	4.1	29.94	BPC 149.0000-3001.0000 +, Masses excluded
2	4.4	100.00	UV Chromatogram, 254 nm
3	4.4	70.06	BPC 149.0000-3001.0000 +, Masses excluded

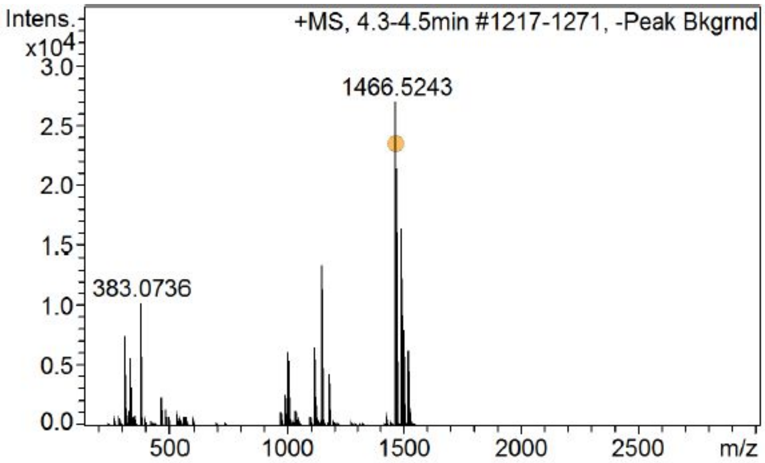

AB3126 (**29**)

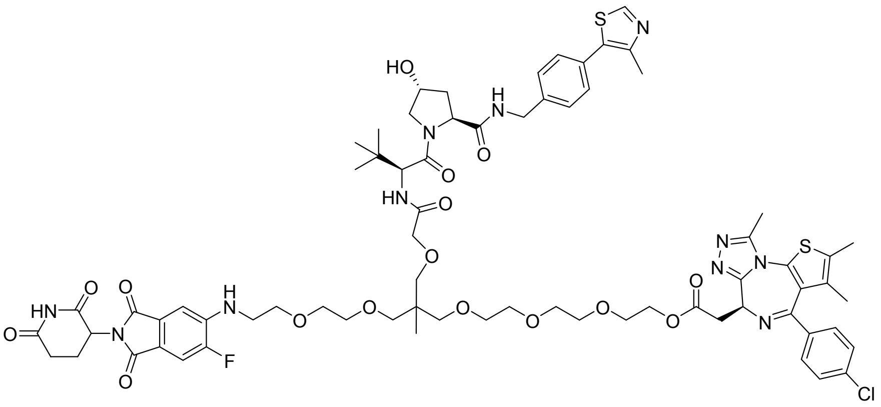

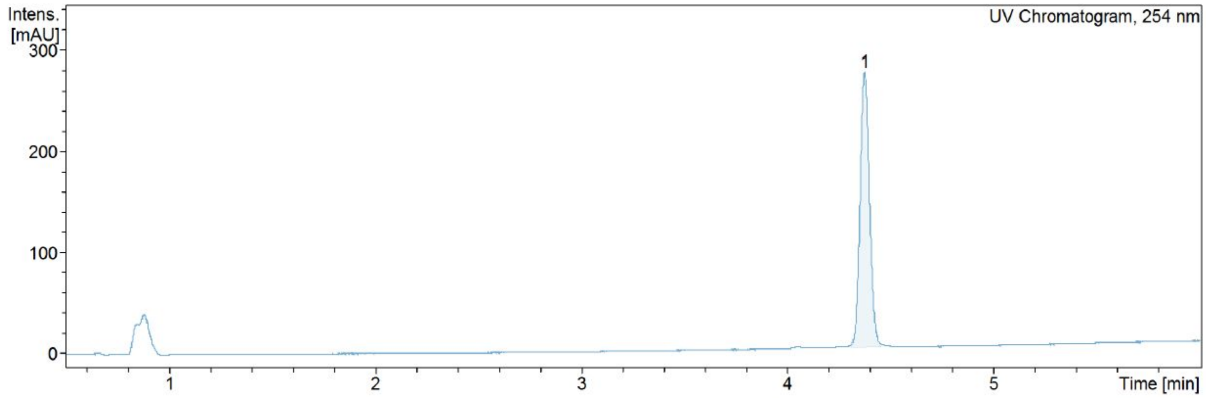

#	RT [min]	Area Frac. %	Chromatogram
1	4.4	100.00	UV Chromatogram, 254 nm
2	4.4	100.00	BPC 150.0000-3001.0000 +, Masses excluded

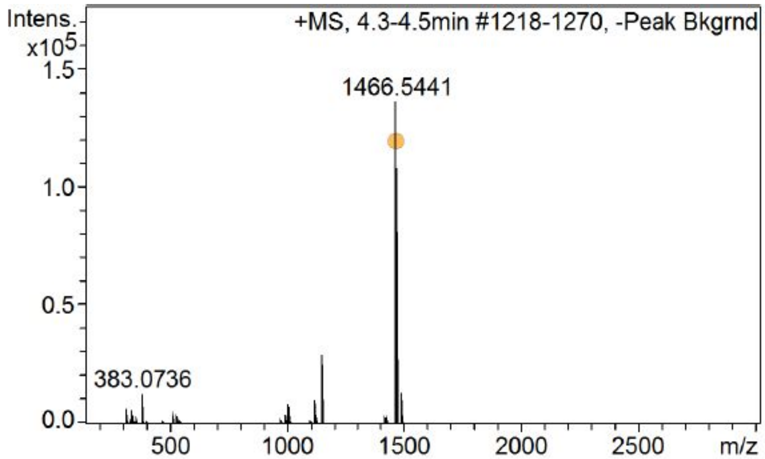

AB3125 (**30**)

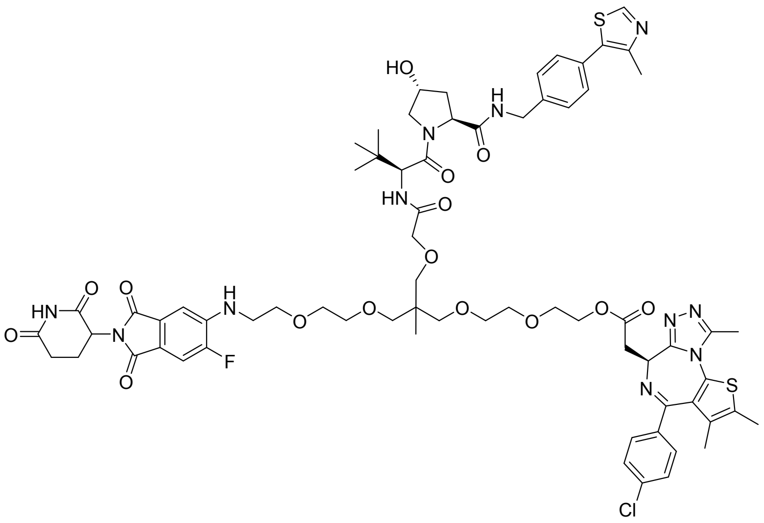

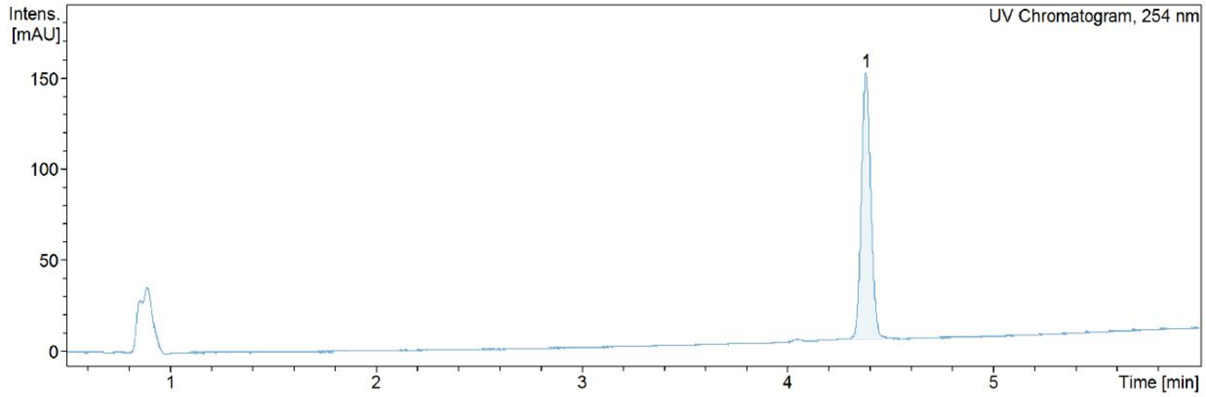

#	RT [min]	Area Frac. %	Chromatogram
1	4.4	100.00	UV Chromatogram, 254 nm
2	4.4	100.00	BPC 149.0000-3001.0000 +, Masses excluded

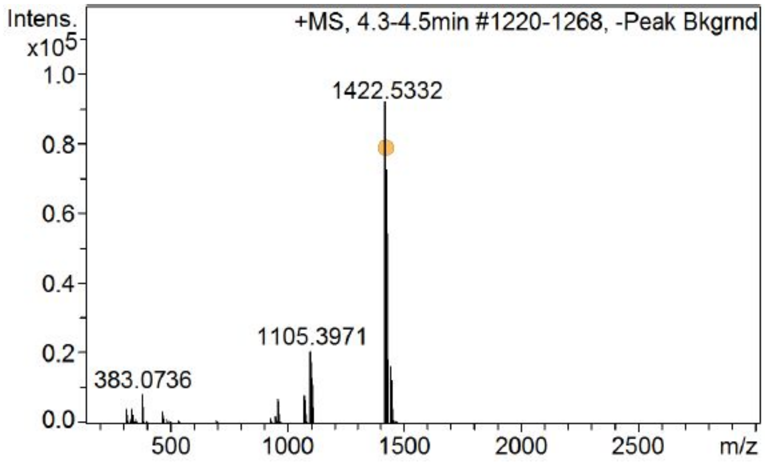

AB3029 (**31**)

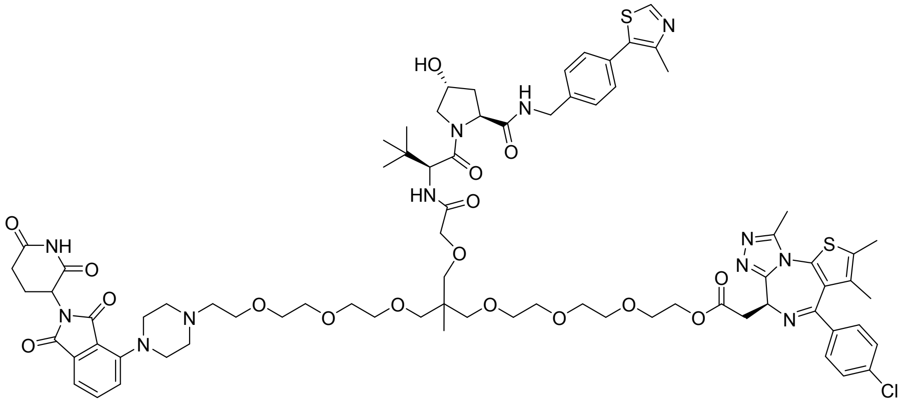

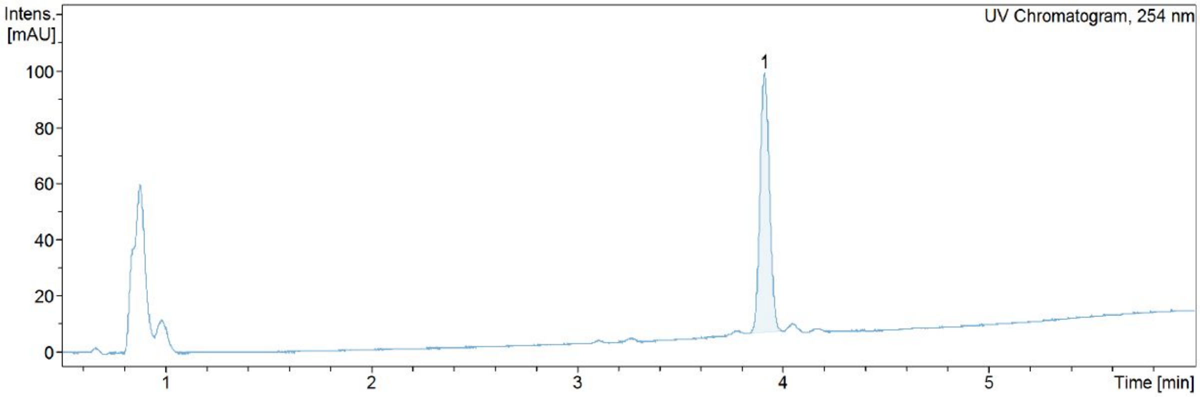

#	RT [min]	Area Frac. %	Chromatogram
1	3.9	100.00	UV Chromatogram, 254 nm

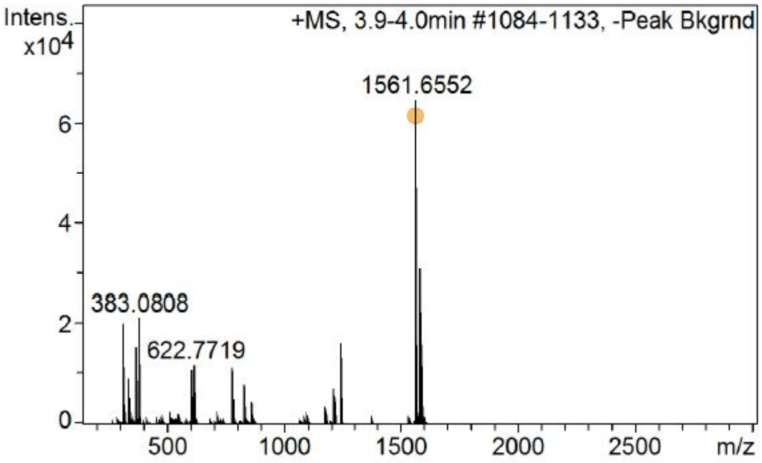

AB3030 (**32**)

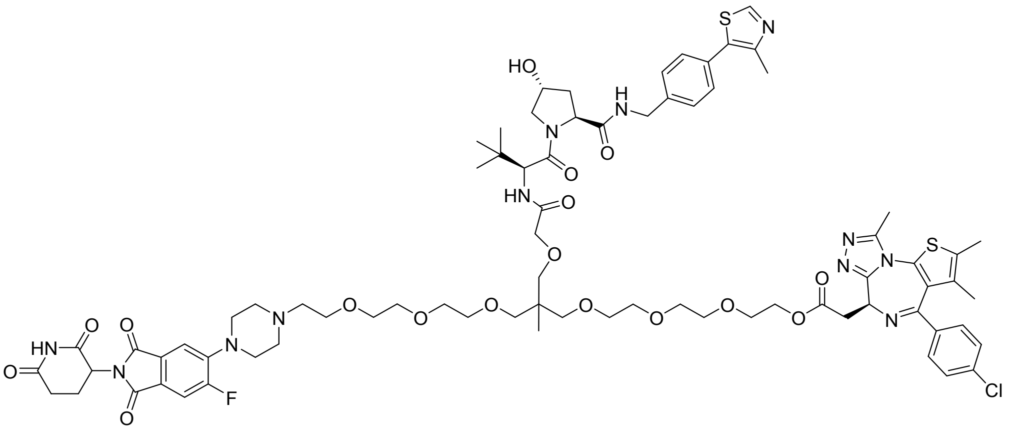

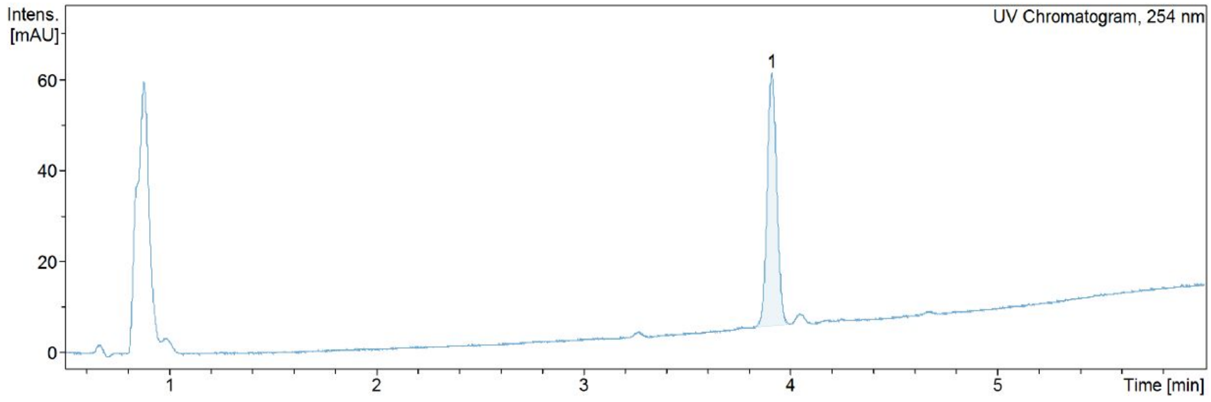

#	RT [min]	Area Frac. %	Chromatogram
1	3.9	100.00	UV Chromatogram, 254 nm

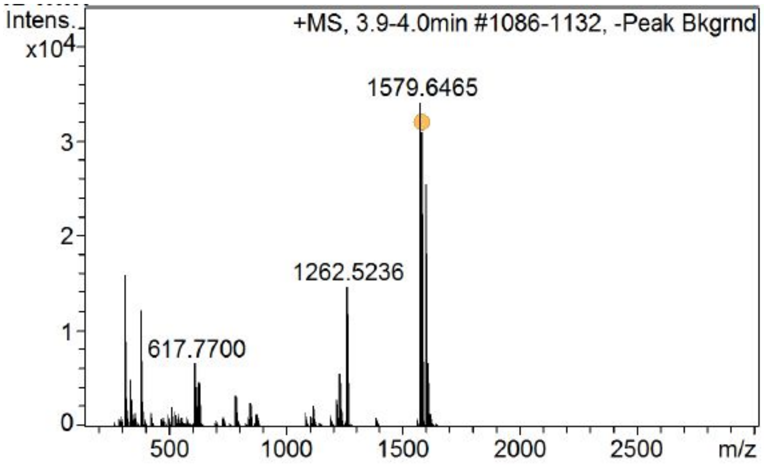

AB3124 (**86**)

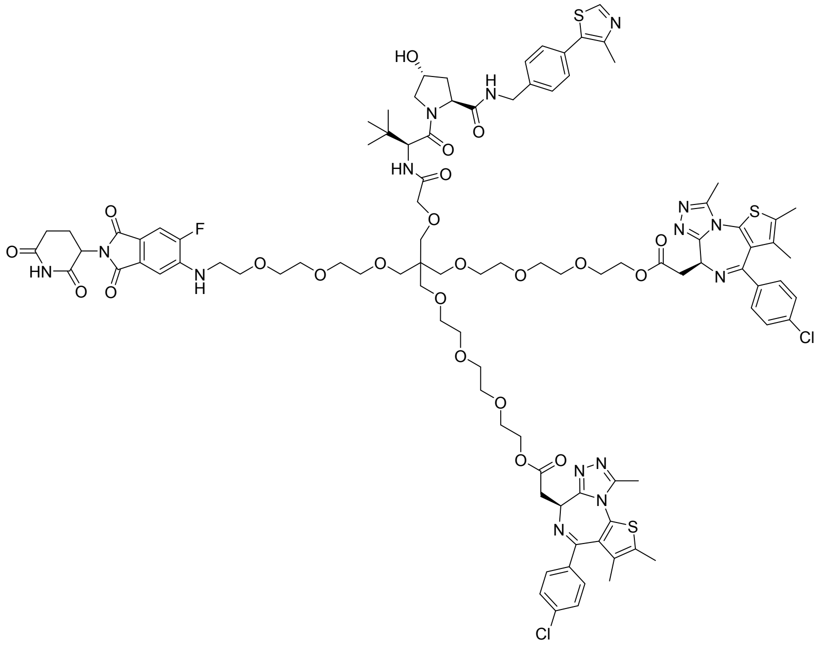

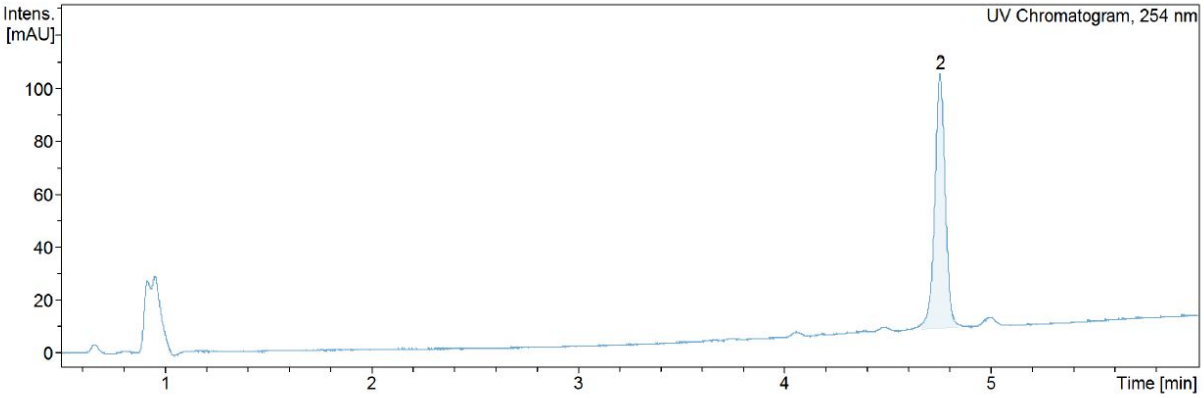

#	RT [min]	Area Frac. %	Chromatogram
1	4.1	17.02	BPC 150.0000-3001.0000 +, Masses excluded
2	4.8	100.00	UV Chromatogram, 254 nm
3	4.8	82.98	BPC 150.0000-3001.0000 +, Masses excluded

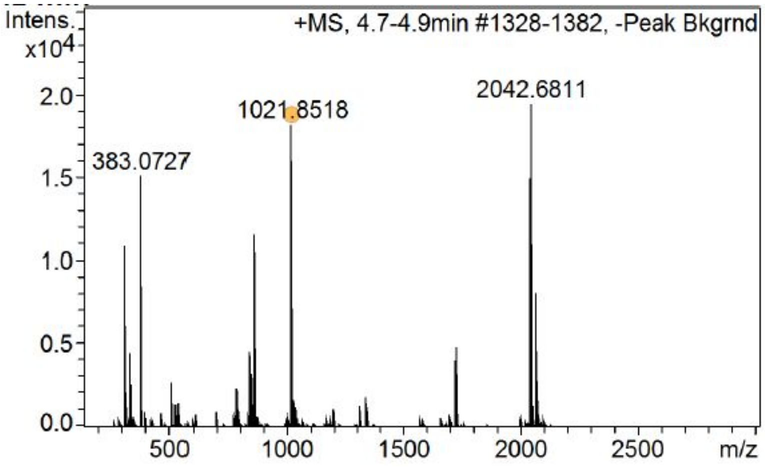

neg-AB3067 (**93**)

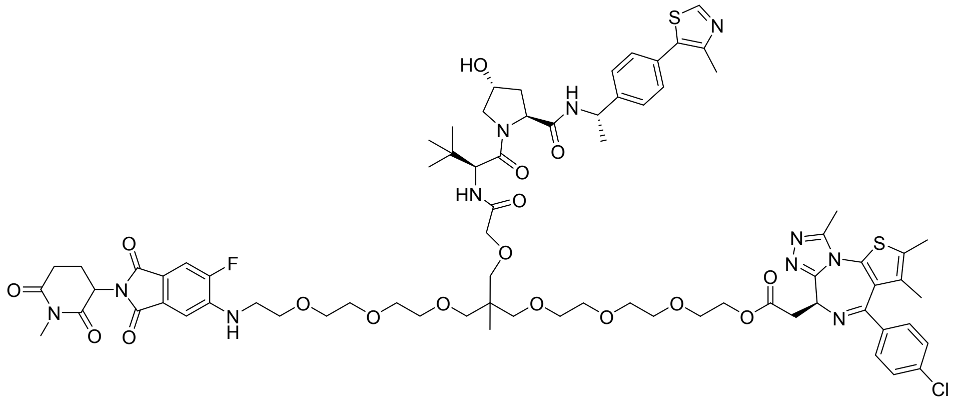

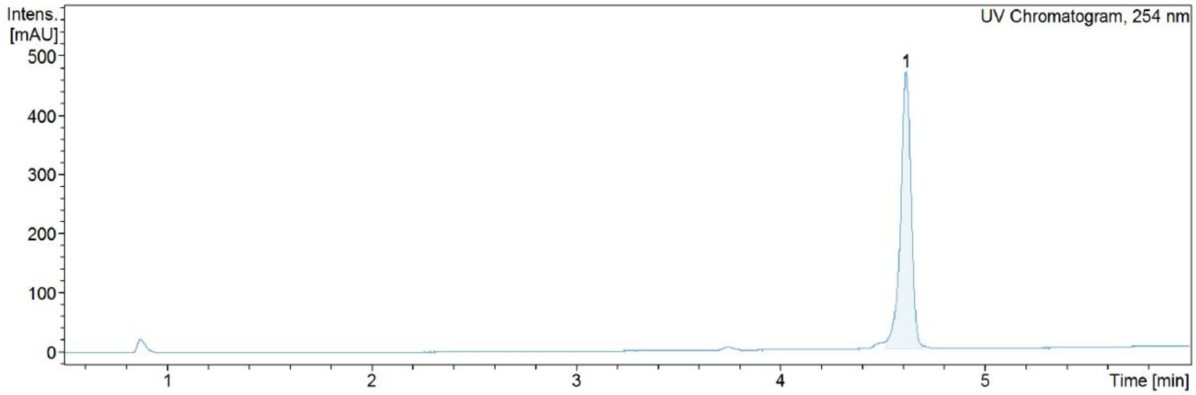

#	RT [min]	Area Frac. %	Chromatogram
1	4.6	100.00	UV Chromatogram, 254 nm

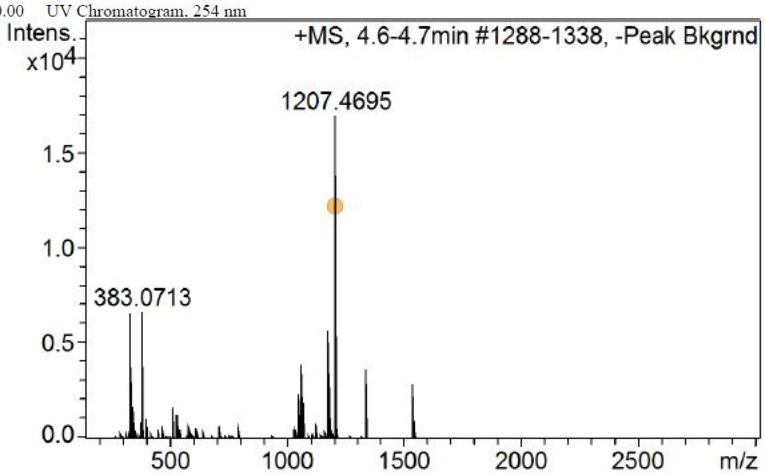

cis-AB3067 (**94**)

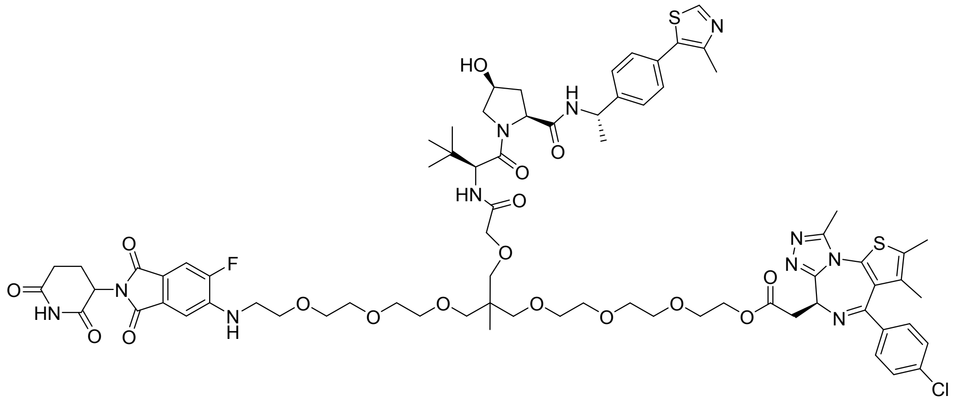

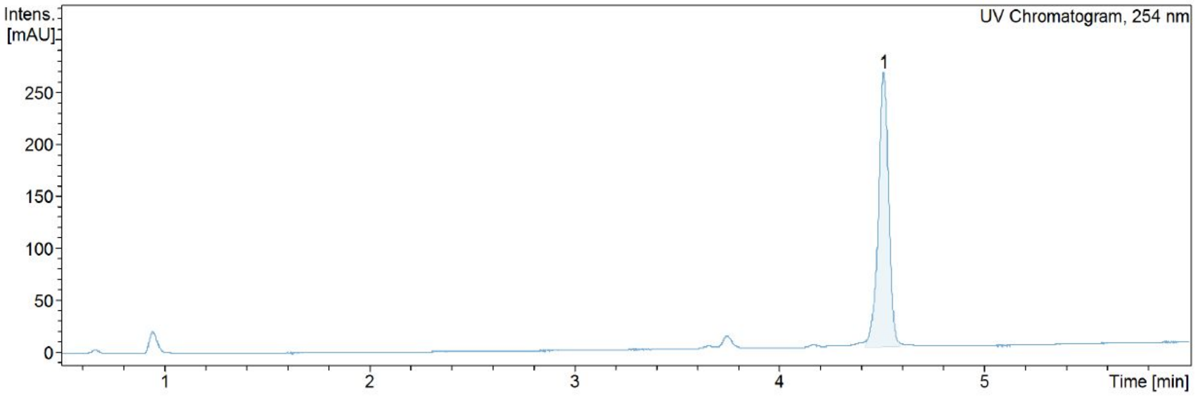

#	RT [min]	Area Frac. %	Chromatogram
1	4.5	100.00	UV Chromatogram, 254 nm

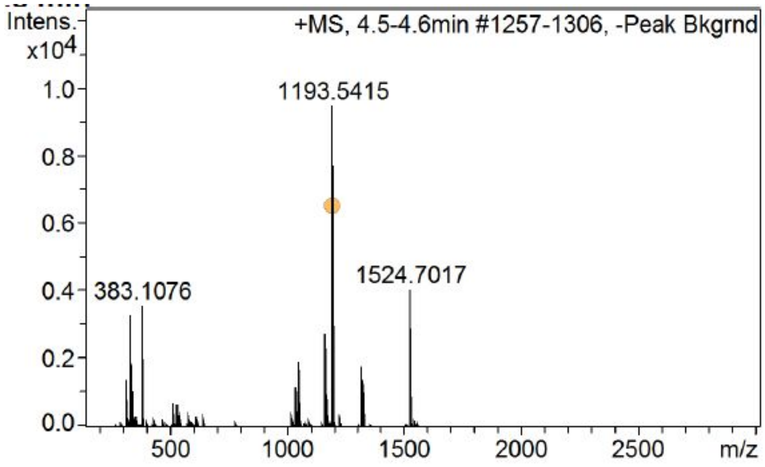

neg-cis-AB3067 (**95**)

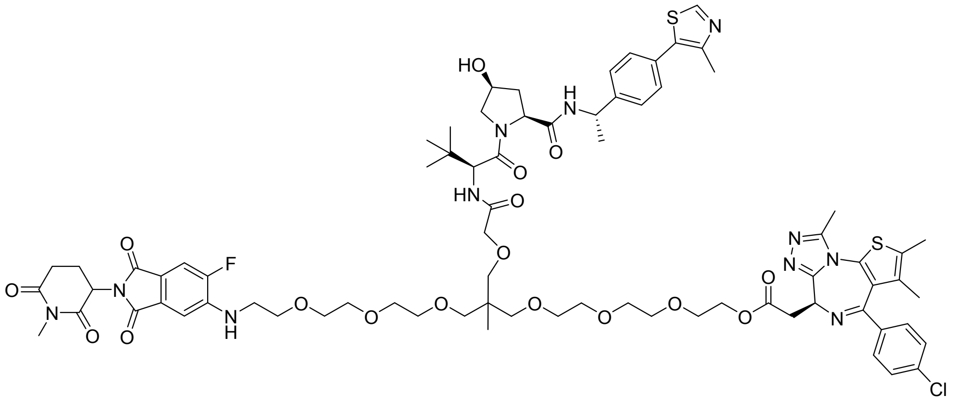

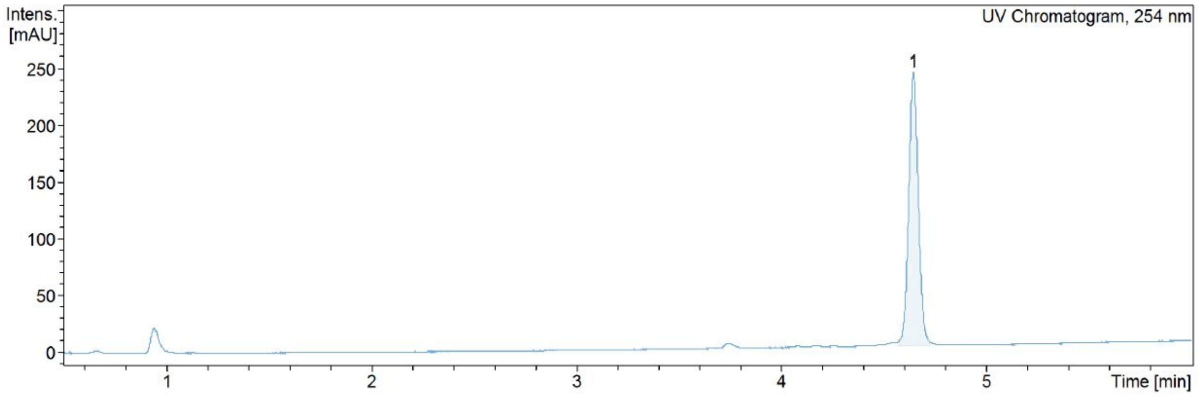

#	RT [min]	Area Frac. %	Chromatogram
1	4.6	100.00	UV Chromatogram, 254 nm

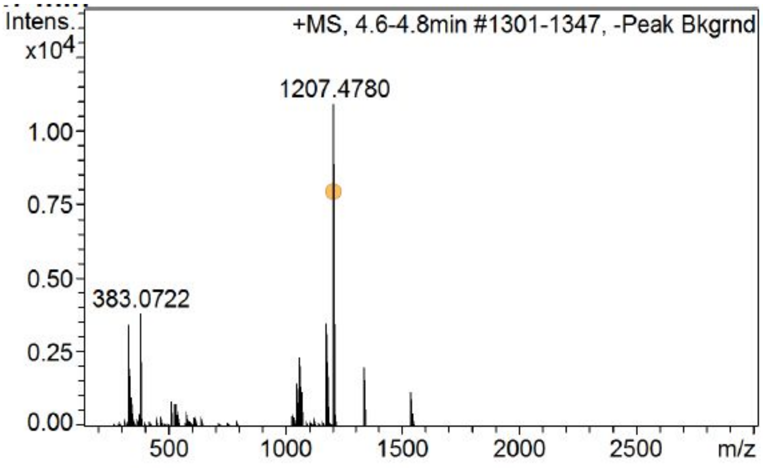

AB3145 (**97**)

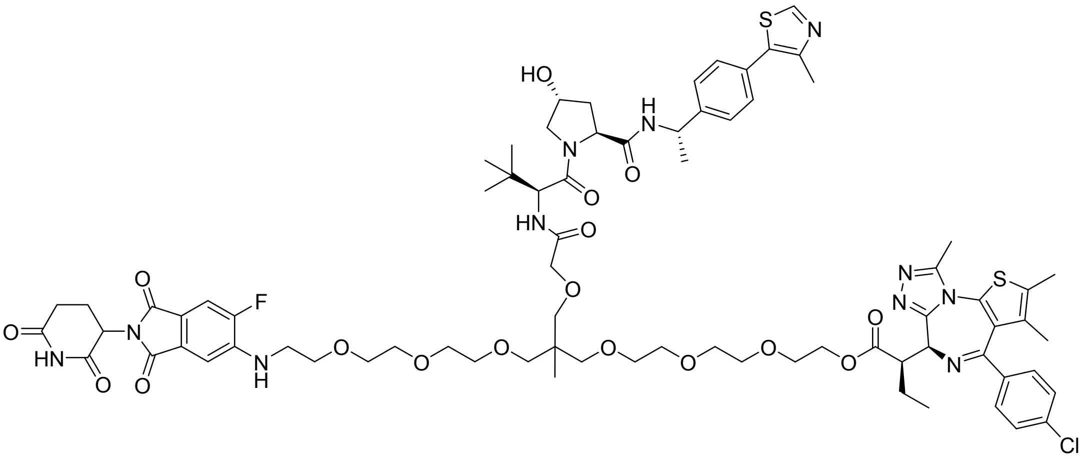

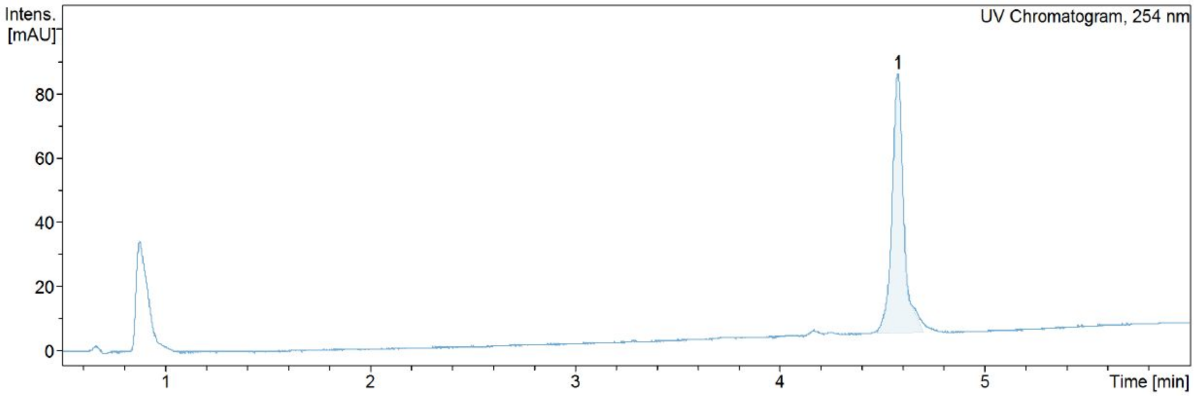

#	RT [min]	Area Frac. %	Chromatogram
1	4.6	100.00	UV Chromatogram, 254 nm

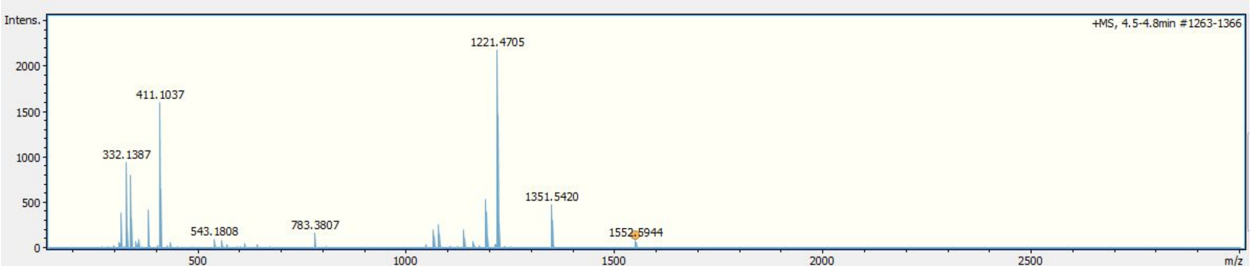

Supplement: Supplementary file 1 — ja4c11556_si_001.pdf [file ja4c11556_si_001.pdf]
